# TSG101 depletion dysregulates mitochondria and PML NBs, triggering MAD2-overexpressing interphase cell death (MOID) through AIFM1-PML-DAXX pathway

**DOI:** 10.1038/s41419-024-07229-w

**Published:** 2024-11-17

**Authors:** Yao Xi, Rui Xu, Shengnan Chen, Jiezhu Fang, Xiang Duan, Yidan Zhang, Guoli Zhong, Zhifei He, Yan Guo, Xinyu Li, Wenzhi Tao, Yang Li, Yan Li, Lei Fang, Yohei Niikura

**Affiliations:** 1 National Resource Center for Mutant Mice, MOE Key Laboratory of Model Animals for Disease Study, Model Animal Research Center, Medical School of Nanjing University, Nanjing, 210061 China; 2 Jiangsu Key Laboratory of Molecular Medicine, Medical School of Nanjing University, Nanjing, 210032 China

**Keywords:** Cell division, Cell death

## Abstract

Overexpression of mitotic arrest deficiency 2 (MAD2/MAD2L1), a pivotal component of the spindle assembly checkpoint (SAC), resulted in many types of cancer. Here we show that the depletion of tumor susceptibility gene 101 (TSG101), causes synthetic dosage lethality (SDL) in MAD2-overexpressing cells, and we term this cell death MAD2-overexpressing interphase cell death (MOID). The induction of MOID depends on PML and DAXX mediating mitochondrial AIFM1-release. MAD2, TSG101, and AIF-PML-DAXX axis regulate mitochondria, PML nuclear bodies (NBs), and autophagy with close inter-dependent protein stability in survival cells. Loss of C-terminal phosphorylation(s) of TSG101 and closed (C-)MAD2-overexpression contribute to induce MOID. In survival cells, both MAD2 and TSG101 localize at PML NBs in interphase, and TSG101 Y390 phosphorylation is required for localization of TSG101 to PML NBs. PML release from PML NBs through PML deSUMOylation contributes to induce MOID. The post-transcriptional/translational cell death machinery and the non-canonical transcriptional regulation are intricately linked to MOID, and ER-MAM, may serve as a crucial intersection for MOID signaling.

## Introduction

The spindle assembly checkpoint (SAC) is a surveillance mechanism and its activation is a fundamental step that ensures faithful chromosome segregation during mitosis. The mitotic arrest deficiency 2 (MAD2) gene was the first characterized gene of the SAC pathway [1, 2]. The mitotic checkpoint complex (MCC) is the effector of the SAC, which inhibits the anaphase-promoting complex (also called cyclosome or APC/C), the E3 ubiquitin ligase required for sister chromatid separation and mitotic exit. The MCC consists of CDC20, MAD2, and BUBR1:BUB3 [3]. Structural conversion from open (O-MAD2) to closed conformation (C-MAD2) is required for MAD2 incorporation in MCC in the SAC activation process [3–6].

Mice genetically engineered to overexpress MAD2 developed chromosome instability (CIN) and aneuploidy [7]. MAD2 overexpression also resulted in the formation of aggressive tumors in multiple organs [8]. Overexpression of MAD2 can be caused by loss of the tumor suppressor Rb or p53 [9, 10]. MAD2 is overexpressed in many cancers [8, 11]. The possible cell fate after Cisplatin and/or Paclitaxel treatment in regard to MAD2 protein level and the influence of MAD2 protein level in therapeutic efficacy were also discussed [12]. On the other hand, investigation of synthetic lethal interactions opens a way to exploit cancer-specific weaknesses and broaden the range of precision oncology, where the simultaneous loss of function of two genes/proteins leads to a lethal outcome [13]. Importantly, MAD2 overexpression could specifically make cancer cells susceptible to death by inducing a synthetic dosage lethality defect [11]. Nevertheless, there remains a dearth of scientific inquiry regarding the intricate functions and networks of MAD2 synthetic dosage lethlity, its intricate connection to SAC functionality, and its potential impact on obliterating cancer cells.

Tumor susceptibility gene 101 (TSG101), initially identified as a tumor suppressor gene in fibroblasts and a microtubule-binding protein, has been found to exhibit dual functionality in some epithelial tumor cells, serving both as a tumor suppressor and a oncogene/protein in subsequent studies [14–17]. TSG101 has also been implicated in multiple cellular functions, including mitosis [14–17] and endosomal sorting and trafficking as a bona fide component of the ESCRT1 [15]. Previous yeast synthetic genetic array (SGA) screen was performed and the 13 genes whose deletion kills Mad2-overexpressing yeast cells were identified [11]. Here we report that depletion TSG101 showed lethality in MAD2-overexpressing human cells. This cell death is triggered in interphase in a p53-independent but AIFM1-dependent and caspase-activated manner, proposing that the TSG101 can be a potential therapeutic target in MAD2-overexpressing tumors. We refer to this cell death as MOID (MAD2-overexpressing interphase cell death). MOID is regulated by the AIFM1-PML-DAXX pathway and PML nuclear bodies (PML NBs). Loss of C-terminal phosphorylations of TSG101 and C-MAD2-overexpression contribute to induce MOID. TSG101 Y390 phosphorylation in survival cells likely enables its localization to PML NBs. Furthermore, overexpressed O-MAD2 primarily binds to Y390-phosphorylated TSG101, while C-MAD2 favors to bind to Y390-non-phosphorylated TSG101. A part of MAD2 colocalizes with PML at PML NBs, and the PML release from PML NBs through PML deSUMOylation contributes to induce MOID. We also identified new genes regulated in MOID through RNA-seq analysis and found that MOID is concomitant with post-transcriptional/translational cell death machinery and non-canonical transcriptional regulation of oxidative stress and ATM/ATR-mediated DNA double-strand breaks (DSB) - DNA damage response (DDR). Our results revealed that ROS and autophagy are activated in MOID, and PML localized at ER-MAM can be a crucial intersection for MOID signaling.

## Results

### Depletion of TSG101 showed synthetic dosage lethality (SDL) in MAD2-overexpressing cells

The previous study used synthetic genetic array to screen for genes whose deletion kills Mad2-overexpressing yeast cells and identified 13 genes with putative human homologs [11]. Depletion of PPP2R1A significantly reduces the growth of MAD2-overexpressing human cells [11], but the other 12 genes were not studied further. This study reassessed mitotic abnormalities in these 12 other genes. We found that depletion of TSG101 significantly increases mitotic index as much as outer kinetochore protein CENP-F (Figs. S1A and S1B, #S1), and percentages of lagging chromosomes, misaligned chromosome, and abnormal number of microtubule organizing center in metaphase (Fig. S1C–S1E).

**Fig. 1:**
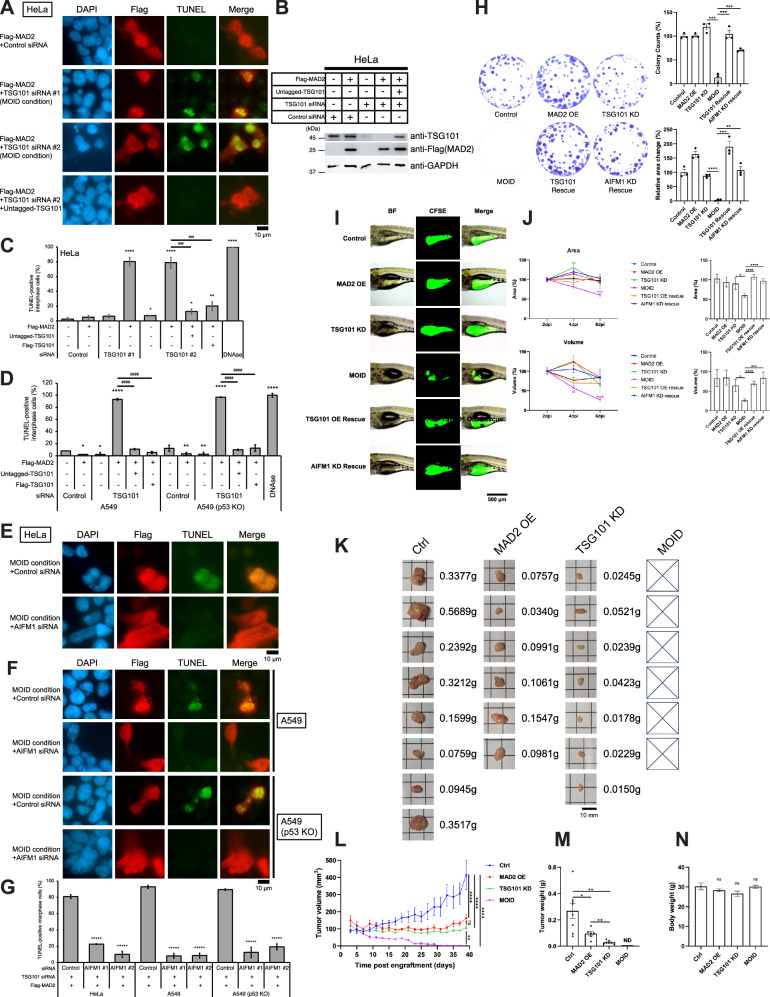
Depletion of TSG101 showed synthetic dosage lethality (SDL) in MAD2-overexpressing cells. **A** TUNEL assay. HeLa cells were transfected with the indicated siRNAs (Table S1) and/or constructs (Table S2). Cells were cultured for 96 h at 37°C. DNA fragmentation was detected by the TUNEL assay, and samples underwent indirect fluorescence microscopy using anti-Flag as a primary antibody to sort out Flag-MAD2 overexpressing cells (see MATERIALS AND METHODS). Scale bar, 10 μm. **B** Western blot analysis of total lysates of HeLa cells transfected with the indicated siRNAs and/or constructs. Cells were cultured for 96 h, collected, lysed, and immunoblotted with the indicated antibodies (Table S3). **C** A histogram summarizing TUNEL assay results of (**A**) (see MATERIALS AND METHODS). The mean percentages (±SD) of TUNEL-positive cells were shown. **D** A histogram summarizing TUNEL assay results (Images are shown in Fig. S2E and S2F). The mean percentages (±SD) of TUNEL-positive cells were shown. **E** TUNEL assay. Scale bar, 10 μm. (F) TUNEL assay. Scale bar, 10 μm. **G** A histogram summarizing TUNEL assay results of (**E**) and (**F**). The mean percentages (± SD) of TUNEL-positive cells were shown. (****) *P* < 0.0001 compared with 1st column from left in each cell line (Student’s t-test). **H** (Left) Colony outgrowth assay. MOID reduces the viability of HSkMCs. (Right) Colony counts and area (%) normalized to the control (1^st^ column from left) are shown summarizing the results of (Left). The mean percentages (± SEM) were shown. **I** Representative images of zebrafish xenografts of HSkMCs (Table S5). Scale bar, 500 μm. **J** HSkMCs-grown area and volume (%) are shown summarizing the results of (**I**) (Table S5). (Left) A line chart from 2-6 dpi. Data of each sample at 2 dpi was normalized to 100%. The mean percentages (± SEM) were shown. (****) *P* < 0.0001, (***) *P* < 0.001, and (**) P < 0.01 (one-way ANOVA). (Right) A histogram of 6 dpi. Data of each sample at 2 dpi was normalized to 100%. The mean percentages (± SEM) were shown. (K-N) Mouse xenografts implanting HeLa cells (see MATERIALS AND METHODS; Table S5). (K) Representative images with scale bar, 10 mm. **L** Tumor volume (mm^2^) at post engraftment (days). ⊠, invisible size of tumor. The mean percentages (± SEM) were shown. (****) *P* < 0.0001 and (**) *P* < 0.01 (one-way ANOVA). **M** Tumor weight. The mean percentages (± SEM) were shown. **N** Body weight. The mean percentages (± SEM) were shown.

After knockdown with the previously reported TSG101 siRNA target (Table S1, TSG101 #S1), the majority of TSG101 protein remained despite mitotic abnormalities (Fig. S1B–S1E, TSG101 #S1), leading us to reassess synthetic dosage lethality using a different siRNA pool (Table S1, TSG101 #1, 2) that more effectively reduced TSG101 protein level (Fig. S1B, TSG101 #1, 2). This new pool showed a high percentage of TUNEL-positive interphase cells under MAD2-overexpression in HeLa cells, and a similar SDL phenotype was observed in four different cell types (HeLa, 293 T, A549 cells, and human primary skeletal muscle cells [HSkMCs]) (Fig. 1A–D and S2A–S2H), although very minor cell line specific lethal effect appeared in A549 cells (Fig. 1D, either MAD2 overexpression or TSG101 depletion rather very slightly reduced the TUNEL positive background compared to the 1st sample from left in each WT and KO).

To test if other ESCRT components can be involved in this SDL, we performed siRNA depletion of other ESCRT component: SNF8 in the ESCRT II complex, CHMP2A in the ESCRT III complex, and ALIX (PDCD6IP) in the accessory complex (Fig. S1F). However, we did not detect the SDL phenotype with depletions of other ESCRT components (Fig. S1G), and depletions of other ESCRT components neither rescue (nor increase) the SDL phenotype caused by TSG101 depletion and MAD2-overexpression (Fig. S1H). These data suggest the independence from other ESCRT (II, II, and accessory) components’ pathways but the specific role of TSG101 in this SDL.

Consistent with the previous report [18] and our data, the mitotic index increased with either MAD2 overexpression or TSG101 depletion, and these 2 effects showed a synthetic tendency, although they are not statistically synergistic (Fig. S1I; we note this tendency was not observed in samples transiently sorted with hygromycin for 2 days as Fig. S9A and B). However, no TUNEL positivity was observed in mitotic cells under MOID condition (Fig. S1J). For convenience and simplicity, the following sentences refer to this cell death as MOID (MAD2-overexpressing interphase cell death; To clearly distinct from the cell death caused by MAD2 overexpression plus depletion of phosphatase PP2A, which was reported previously [11], and other mitotic cell death caused by (even partial) SAC dysfinction [19–21], the term “interphase” is included in “MOID”).

The results of our TUNEL assay are consistent with the results of our colony outgrowth assay and zebrafish xenograft implanted with human cells, including HSkMC, HeLa, and human rhabdomyosarcoma cell line Rh30, which shows a higher protein level of MAD2 (Fig. 1H–J and S3A–S3G; Table S5 for simplified sample indications). In Rh30-transplanted zebrafish xenografts, merely-TSG101-depleted cells showed severe lethality (Fig. S3D). Our results of mouse xenograft implanted with HeLa cells showed complete tumor eradication of MOID cells (Fig. 1K–N; ⊠, invisible size of tumor).

### MOID is p53-independent but AIFM1-dependent and caspase-activated cell death

**Fig. 2:**
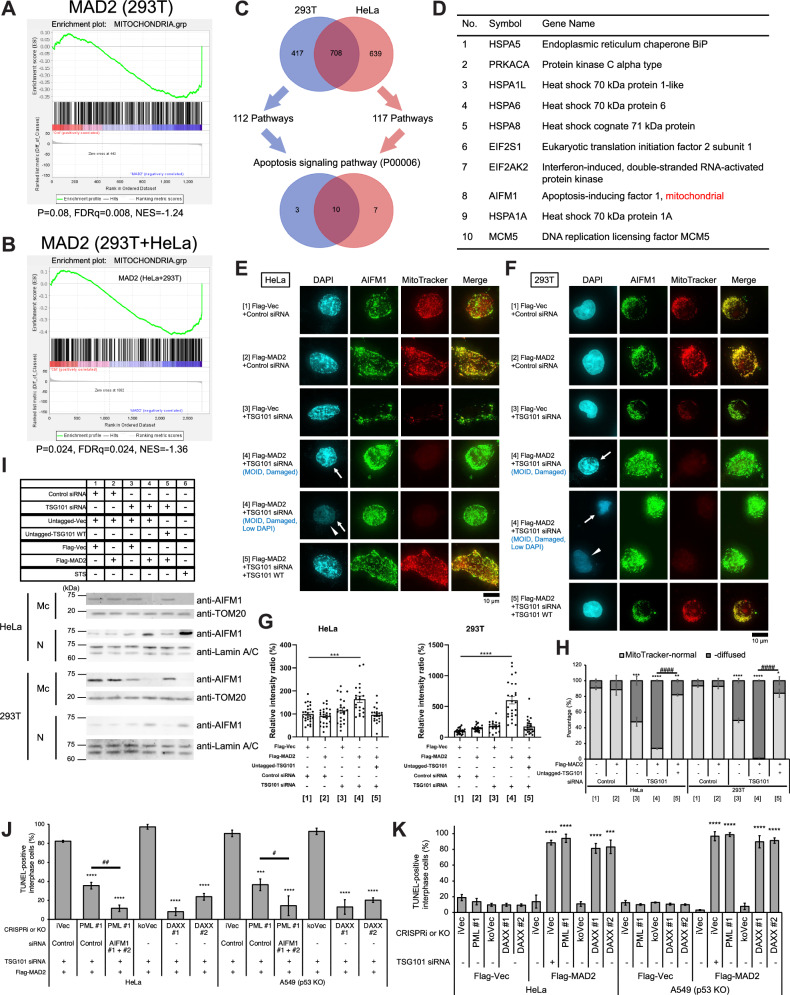
MOID induction depends on PML-DAXX function concomitant with mitochondrial AIFM1-release, but the inductive or suppressive role of PML-DAXX during MOID is TSG101-dependent. **A** and **B** Peptide set enrichment analysis (PSEA; see MATERIALS AND METHODS) of Flag-MAD2 interacting peptides was performed for 293 T (**C**) and HeLa+293 T (**D**) samples. P- and FDRq-values and NES were indicated. **C** Peptides interacting with MAD2 were identified from Mass-spec in both 293 T and HeLa, which were enriched in 112 and 117 pathways after PANTHER19.0 pathway analysis, respectively. 10 genes were identified from both 293 T and HeLa, which were annotated as Apoptosis signaling pathway (P00006) (MATERIALS AND METHODS). **D** A table showing that AIFM1 is found among 10 genes identified as **C**. **E** and **F** An anti-AIFM1 (green) rabbit polyclonal antibody (Table S3) was used to detect the release of AIFM1 from mitochondria marked with MitoTracker (red) during MOID. Arrows, damaged nuclei. Arrowheads, low DAPI nuclei. Scale bar, 10 μm. **G** Quantitated immunofluorescence signals of AIFM1 in nuclear shown in **E** and **F** (see MATERIALS AND METHODS). Signals were normalized to those of each Vector control (Ctrl), and the mean percentages (±SEM) are shown. **H** A histogram summarizing the pattern of MitoTracker signals shown in **E** and **F** (see MATERIALS AND METHODS). The mean percentages (± SD) of each pattern were shown. **I** Subcellular fractions isolated from 293 T and HeLa cells after the transfection with the indicated siRNAs and/or constructs. N, nuclear; Mc, crude mitochondria. STS, staurosporin (1.0 μM) control (see MATERIALS AND METHODS). Lamin A/C was used as a nuclear marker, TOM20 as a mitochondria marker. Note that we could not detect AIFM1 bands even with staurosporine (1.0–100 μM) control in cytosol fraction, presumably due to diluted conditions during sample preparation (data not shown). **J** A histogram summarizing TUNEL assay results as described in Fig. 1C (see MATERIALS AND METHODS). The mean percentages (± SD) of TUNEL-positive cells were shown. iVec indicates CRISPRi vector control. koVec indicates CRISPR KO vector control. **K** A histogram summarizing TUNEL assay results as described in Fig. 1C (see MATERIALS AND METHODS). The mean percentages (± SD) of TUNEL-positive cells were shown. iVec indicates CRISPRi vector control. koVec indicates CRISPR KO vector control.

To investigate the mechanism of MOID, MAD2 interactors were identified in Flag-MAD2-overexpressing HeLa and 293 T cells. Flag-MAD2 immunopurification was followed by LC-MS/MS, identifying Flag-MAD2 peptides and interactors (Table S4). Immunoprecipitates were analyzed, revealing a higher number of peptides for p53, p53BP1 of 293 T cells, and Apoptosis-inducing factor mitochondria associated 1 (AIFM1) of both 293 T and HeLa cells in Flag-MAD2 immunoprecipitates compared with Flag-vector control (Table S4). We performed peptide set enrichment analysis (PSEA) using peptide counts from our serial IP-mass spectrometry analyses. The enrichment plot of the Flag-MAD2 immunoprecipitants shows a higher score in “mitochondria”-related genes compared with the control (Fig. 2A and B). Then, we found AIFM1 appeared among 10 genes identified from both 293 T and HeLa, which were annotated as Apoptosis signaling pathway (P00006) (Fig. 2C and D; MATERIALS AND METHODS), further supporting our rationale to study AIFM1. Later, we found that both MAD2 and AIFM1 peptides appeared also in Flag-PML immunoprecipitants (Table S4; see also next section). Together, these data suggest that MAD2 functions in both PML NBs and mitochondria, and support our rationale to study AIFM1 as a key regulator of MOID.

Our IP-western blot analysis also revealed the specific interaction of Flag-MAD2 with endogenous p53 in 293 T cells (Fig. S4A; Table S4). However, because we observed MOID in HeLa cells with inactivated p53 gene by HPV E6 protein [22], we hypothesized that MOID is p53-independent cell death. We also confirmed that MOID occurred in cells lacking the p53 gene (Figs. 1D and S2E–S2G). AIFM1 regulates caspase-dependent and independent cell death [19, 20, 23]. We performed siRNA knockdown of p53 or AIFM1 in p53 gene-active cells (293 T and HSkMC) and found that p53 knockdown had no effect on MOID, but AIFM1 knockdown suppressed MOID regardless of p53 status (Figs. 1E–G and S4B–S4E). We observed interaction between Flag-MAD2 and TSG101, which decreased in MOID condition due to TSG101 depletion (Fig. S4F, samples 2-4). However, Flag-MAD2 and AIFM1 interaction increased in MOID condition (Fig. S4F, sample 4). Caspase activation was also observed in MOID cells (Fig. S4G and S4H), indicating caspase-activated cell death. Collectively, these data indicate that DNA double-strand breaks occur in four different cell types regardless of p53 status, suggesting that MOID is p53-independent but AIFM1-dependent.

### MOID induction depends on PML-DAXX function concomitant with mitochondrial AIFM1-release, but the inductive or suppressive role of PML-DAXX during MOID is TSG101-dependent

TSG101 interacts with DAXX and acts as a transcriptional repressor of nuclear glucocorticoid receptor [24], but DAXX can also function as a transcriptional coactivator [25]. DAXX is a centromere protein that acts as a histone H3 and/or CENP-A chaperon [26, 27] as well as PML interactor that acts as a major component of PML NBs [28, 29]. DAXX mediates apoptosis through Fas ligand stimulation [30], and recent evidence suggests that PML directly executes apoptotic caspases [25]. The precise apoptotic mechanism in PML-NBs remains hypothetical and needs further study [25]. These past studies intrigued us to assess the role of PML-DAXX in the MOID. Our IP-mass spectrometry analyses identified both MAD2 and AIFM1 peptides in Flag-PML immunoprecipitates (Table S4). Interestingly, a relatively higher number of peptides of mitochondrial proteins were also identified in Flag-PML, MAD2, TSG101, and DAXX immunoprecipitates, respectively, compared with other Flag-tagged proteins (EVI1, L1CAM, or DYX1C1) which are presumably not involved in the MOID pathway, but identified as interactor of p53, MAD2 and L1CAM, or AIFM1, respectively in our analyses (Fig. S5A and S5B; Table S4) and/or previous report [31]. Consistent with previous reports [32–34], our series of IP-mass spectrometry analysis revealed the interaction of PML and centromere-kinetochore proteins (e.g., MAD2, SUGT1, BUB3, BugZ, CENP-V; Table S4). MOID cells had heterogeneous nuclear morphologies, including normal DAPI morphology (early stage), damaged/condensed DNA (later stage), and low DAPI signals (later stage) (Fig. 2E and F; see also further results in Fig. 4H). Our results of immunofluorescence (Fig. 2E–H) and mitochondrial fractionation (Fig. 2I) showed that mitochondrial morphology was disrupted (Fig. 2H) and AIFM1 was released from mitochondria into nucleus during MOID (Fig. 2G and I).

These of our preliminary data encouraged us to assess the effect of single depletion of PML, DAXX, MAD2, and TSG101 on cellular events, including mitochondrial function, colocalization between mitochondria and LC3B (mitophagy-like status), PML NB formation, and colocalization between mitochondria and PML NBs which implies PML-MAMs (Mitochondria-associated endoplasmic reticulum membranes) interaction that modulates autophagy, tumor immune microenvironment, and cancer development [35, 36] (Fig. S5C–S5P; Supplemental Result 1). We summarized the close relationship between PML, DAXX, MAD2, and TSG101 in these cellular events, observed through immunofluorescence signaling (Fig. 6Q, left). We found that these proteins (PML, DAXX, MAD2, and TSG101) maintain a close dependence and contribute mutually to protein stability sustaining inter-organelle integrity, although DAXX might have an inhibitory effect on PML (Fig. S6A–S6C; Supplemental Result 1). Thus, we summarized the close relationship between PML, DAXX, MAD2, and TSG101 in protein stability (Fig. 6Q, left).

Single depletion of PML or DAXX (Figs. 2J and S6D) rescued the percentage of MOID, with AIFM1 depletion having a stronger effect than PML depletion. Double depletion of AIFM1 and PML further rescued the percentage of MOID induction (Figs. 2J and S6D; Supplemental Result 2), suggesting AIFM1 as a relatively upstream factor in the MOID induction pathway (Fig. 6Q, right; Supplemental Result 2). On the other hand, PML or DAXX single depletion induced MOID at a high percentage with MAD2 overexpression, like TSG101 single depletion, when MAD2 was overexpressed (Fig. 2K and S6E). These data suggest that PML/DAXX inductive or suppressive role depends on TSG101 protein level.

Together, MAD2, TSG101, and the components in AIF-PML-DAXX axis are interdependent in protein stability, and regulate mitochondria and PML NBs. MOID induction depends on PML-DAXX function concomitant with mitochondrial AIFM1-release, but the inductive or suppressive role of PML-DAXX during MOID is TSG101-dependent.

### Loss of C-terminal phosphorylations of TSG101 and C-MAD2-overexpression contribute to induce MOID

**Fig. 3:**
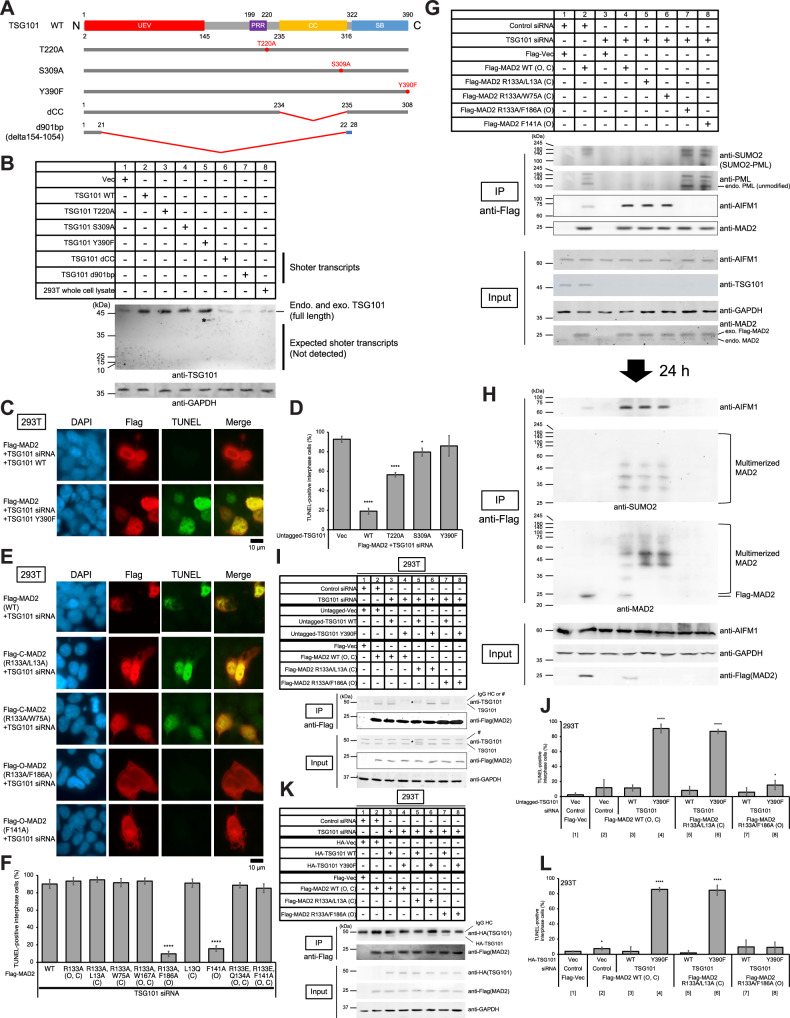
Loss of C-terminal phosphorylations of TSG101 and C-MAD2-overexpression contribute to induce MOID. **A** Schematic figures of each TSG101 construct used in this study. **B** Western blot analysis of total lysates of HeLa cells transfected with the indicated siRNAs and/or constructs. Cells were cultured for 48 h, collected, lysed, and immunoblotted with the indicated antibodies (Table S3). Asterisk indicates background signals. **C** Loss of C-terminal phosphorylations of TSG101 contributes to induce MOID. 293 T cells were transfected with the indicated siRNAs and/or constructs. Cells were cultured for 96 h at 37°C. DNA fragmentation was detected by the TUNEL assay (see MATERIALS AND METHODS). Scale bar, 10 μm. **D** A histogram summarizing TUNEL assay results shown in **C**. The mean percentages (± SD) of TUNEL-positive cells were shown. **E** C-MAD2-overexpression is required to induce MOID. HeLa cells were transfected with the indicated siRNAs and/or constructs. Cells were cultured for 96 h at 37°C. DNA fragmentation was detected by the TUNEL assay as described in Fig. 1A (see MATERIALS AND METHODS). Scale bar, 10 μm. **F** A histogram summarizing TUNEL assay results shown in **E**. The mean percentages (± SD) of TUNEL-positive cells were shown. **G** O-MAD2-overexpression does not induce MOID and maintains PML SUMOylation. 293 T cells were transfected with the indicated constructs, and pellets were collected 96 h after the transfection, which is the timing when TUNEL-positive MOID cells were observed. Proteins in 5% of the total cell lysates (Input) and immunoprecipitates (IP) were detected by western blot analysis using the indicated antibodies. **H** MOID induced by C-MAD2-overexpression promotes multimerization and SUMOylation of C-MAD2 at the later stage of MOID, while overexpressed O-MAD2 that blocks MOID is degraded at the same timing. 293 T cells were transfected with the indicated constructs, and pellets were collected 120 h after the transfection (24 h after the timing of (**G**) sample). Proteins in 5% of the total cell lysates (Input) and immunoprecipitates (IP) were detected by western blot analysis using the indicated antibodies. **I** Majority of overexpressed O-MAD2 binds Y390-intact TSG101 WT, while overexpressed C-MAD2 binds TSG101 Y390F mutant. Immunoprecipitation assay (see MATERIALS AND METHODS). 293 T cells were transfected with the indicated constructs, and pellets were collected 96 h after the transfection, which is the timing when TUNEL-positive MOID cells were observed. Proteins in 1.6% of the total cell lysates (Input) and immunoprecipitates (IP) were detected by western blot analysis using the indicated antibodies. Asterisk (*) indicates putative posttranslationally modified TSG101 band. Hash (#) indicates non-specific bands. **J** A histogram summarizing TUNEL assay results corresponding to the samples shown in **I** using untagged-TSG101. The mean percentages (± SD) of TUNEL-positive cells were shown. **K** Immunoprecipitation assay performed as **I** (see MATERIALS AND METHODS), but using HA-TSG101. Proteins in 3.0% of the total cell lysates (Input) and immunoprecipitates (IP) were detected by western blot analysis using the indicated antibodies. **L** A histogram summarizing TUNEL assay results corresponding to the samples shown in **K** using HA-TSG101. The mean percentages (± SD) of TUNEL-positive cells were shown.

Previously reported phosphorylation sites of TSG101 are T220 and Y390 with unknown function, and T220 as putative MST3 substrate [37]. Our LC-MS/MS identified novel phosphorylation site S309 (data not shown). We constructed mutants of these sites. We also constructed deletion mutants of the coiled-coil domain (TSG101 dCC), which was reported as the interaction domain with DAXX [24], and TSG101 d901bp (delta154-1054), which consists of only 28 amino acids’ peptides missing residues 204-1104 and is predominant in most cancer tissues [38] (Fig. 3A and B). However, protein expression of these two mutants, dCC and d901bp, cannot be confirmed by western blot due to protein instability or short peptide size, respectively, even in 2 independent bacterial plasmid clones of each (Fig. 3B, samples 6 and 7, and data not shown). TSG101 WT overexpression rescues MOID, but mutants of C-terminal phosphorylation sites abolish this rescue (Fig. 3C and D), indicating presumable contribution of loss of TSG101 C-terminal phosphorylations to MOID induction.

Using siRNAs targeting UTR of TSG101 (Table S1, TSG101 #2) combining untagged TSG101 overexpression as endogenous TSG101 protein level (Fig. S4F, compare samples 1 and 5) with transient hygromycin selection for 2 days, we could replace ca. 85% endogenous TSG101 with exogenous Flag-TSG101 (WT or Y390F; Figs. S1B, S4F, compare samples 1-2 and 5 of anti-TSG101 bands in input), then we performed immunofluorescnce using these cells. In survival cells without MAD2 overexpression, Flag-TSG101 Y390F showed the loss of colocalization with PML NBs (Fig. S6F and S6G; Supplemental Result 1) without affecting the integrity of PML NBs (Fig. S6H; Supplemental Result 1) compared to Flag-TSG101 WT. These Y390F mutant cells have a significantly higher population of abnormal nuclei (Fig. S6I; Supplemental Result 1), although most of them are TUNEL-negative cells (Fig. S6J; Supplemental Result 1). These data suggest that TSG101 Y390 phosphorylation is presumably required for TSG101 localization to PML NBs (or colocalization between TSG101 and PML NBs).

In SAC activation, MAD2 structural conversion from open to closed conformation is required for its incorporation in MCC. MAD2 binds to MAD1 or CDC20 through MAD2-interacting motif (MIM) [5]. The MAD1-closed MAD2 complex recruits open MAD2, which binds CDC20 to block APC/C^CDC20^ activity [4, 6]. This process remodels MAD2 from open (O-MAD2) to closed (C-MAD2) conformation with its structurally flexible C-terminal “safety belt”/“seatbelt” [5]. We were intrigued to determine the impact of these two conformations in MAD2-overexpressing cancer cells on MOID induction in interphase (Fig. S6K). Overexpression of O-MAD2 reduces MOID occurrence, but not C-MAD2 or coexisting form (O,C)-overexpressing cells (Fig. 3E and F). Overexpressed C-MAD2 enhances binding to endogenous AIFM1 and induces MOID as MAD2 WT (Fig. 3G, samples 4-6), while overexpressed O-MAD2 loses basal interaction with AIFM1 and suppresses MOID induction (Fig. 3G, samples 2, 7, and 8).

Notably, overexpressed Flag-MAD2 binds reducedly to endogenous PML and reduces SUMOylation of PML under MOID conditions (Fig. 3G, samples 2 and 4-6), while Flag-O-MAD2 binds excessively to PML and causes excessive SUMOylation when inhibiting MOID induction (Fig. 3G, samples 7 and 8). These changes were observed at 96 h post-transfection as shown in the TUNEL assay (Fig. 3E and F), suggesting that overexpressed Flag-MAD2 with C-MAD2 causes PML deSUMOylation and is released from PML NBs in MOID (see also next section). However, 24 hours later (120 h post-transfection), overexpressed Flag-C-MAD2 causes SUMOylation and multimerization in MOID conditions (Fig. 3H, samples 4-6; Flag-MAD2 monomers disappeared in MOID conditions due to the multimerization and SUMOylation both in Input and IP), but Flag-O-MAD2 overexpression promotes proteolysis when TSG101 is depleted (Fig. 3H, samples 7 and 8; Flag-O-MAD2 itself was not detected even in Input).

Because we detected the basal interaction between endogenous TSG101 and overexpressed MAD2 (Fig. S4F, sample 2), but that interaction is lost with TSG101 depletion or in MOID condition (Fig. S4F, samples 3 and 4) and rescued back with untagged TSG101 overexpression (Fig. S4F, sample 5), we tested the interactions among TSG101 WT, Y390F and MAD2 WT, C-MAD2, O-MAD2. Using siRNAs targeting UTR of TSG101 (Table S1, TSG101 #2) combining untagged-TSG101 overexpression as endogenous TSG101 protein level (Fig. S4F, compare samples 1-2 and 5 of anti-TSG101 bands in input) with transient hygromycin selection for 2 days, we could replace ca. 85% endogenous TSG101 with exogenous untagged-TSG101 (WT or Y390F; Figs. S1B and S4F), then we performed immunoprecipitation assay with these lysates. MAD2 WT that has presumably higher population of interphase O-MAD2 or O-MAD2 showed higher interaction with untagged-TSG101 WT, while C-MAD2 showed higher interaction with untagged-TSG101 Y390F (Fig. 3I and J; Note that samples 4 and 6 are MOID). Our TUNEL analysis also showed that C-MAD2 maintained the binding with untagged-TSG101 Y390F and induced the MOID (Fig. 3I and J; sample 6), suggesting that loss of Y390 phosphorylation is required for MOID induction rather than loss of MAD2-TSG101 binding itself (see Discussion). Usage of HA-TSG101 led to the similar results (Figs. 3K, L, and S7A–S7C). Together, these data suggest that C-MAD2 overexpression is required for MOID induction while O-MAD2 overexpression has a suppressive effect on the MOID induction. Overexpressed O-MAD2 primarily binds to Y390-phosphorylated TSG101, while C-MAD2 favors to bind to Y390-non-phosphorylated TSG101. Thus, conformational changes from O-MAD2 to C-MAD2 may play an important role on the MOID induction (see Discussion).

### MAD2 colocalizes with PML at PML NBs, and PML release from PML NBs through PML deSUMOylation contributes to induce MOID

Our results showed that endogenous PML is excessively SUMOylated under conditions where O-MAD2 overexpression suppresses MOID induction (Fig. 3E and F). Therefore, we studied how the SUMOylation of PML is functionally related to the mechanism of MOID. PML NBs formation is mainly regulated by PML SUMOylation [39]. Previously, 3 SUMOylation sites (K65, K160, and K490) of PML were reported [40–42], and major SUMOylation sites among these 3 sites is K160 [42]. PML coiled-coil domain is also important for PML dimerization-multimerization, and subsequent PML SUMOylation [42].

**Fig. 4:**
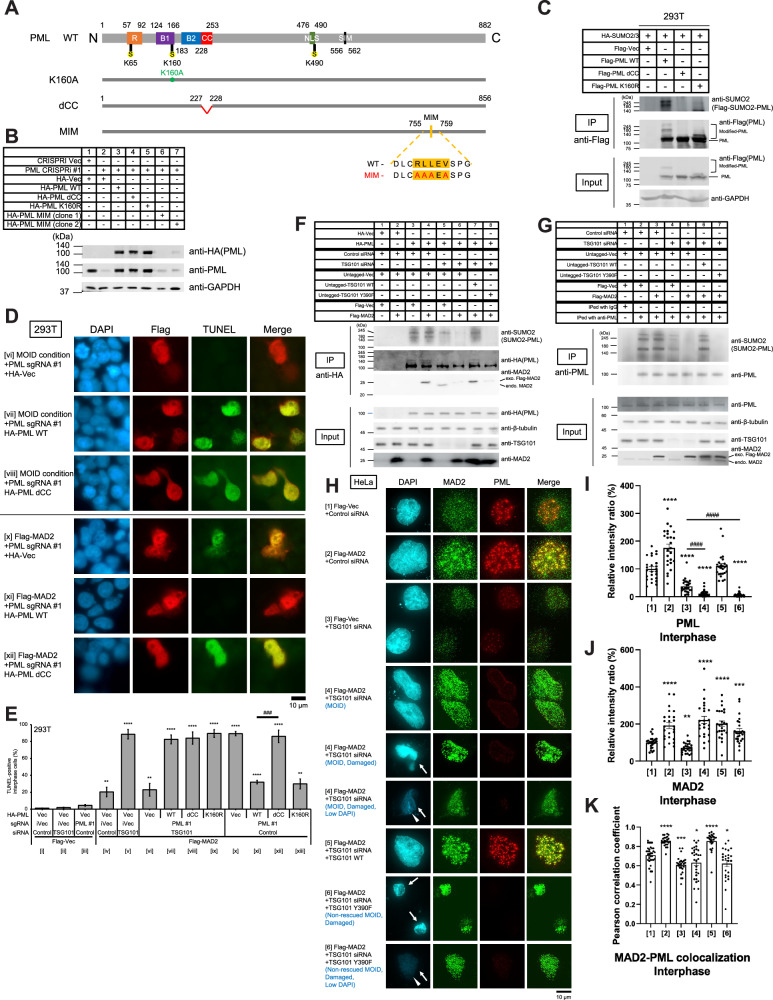
MAD2 colocalizes with PML at PML NBs, and PML release from PML NBs through PML deSUMOylation contributes to induce MOID. **A** PML constructs used in this study. All the PML constructs were fused to an N-terminal HA tag and RNAi-resistant. **B** Western blot analysis using 293 T total cell lysates. Cells were cultured and harvested 96 h after transfection with the indicated constructs. **C** Representative images of the in vivo SUMOylation assay with the HA-PML constructs (WT and mutants) using 293 T cells (see MATERIALS AND METHODS). **D** DeSUMOylated PML delta coiled-coil (dCC) mutant did not suppress MOID induction with Flag-MAD2 overexpression and single PML depletion, and constitutively induces MOID. TUNEL assay of 293 T cells was performed as described in Fig. 1A (see MATERIALS AND METHODS).Scale bar, 10 μm. **E** A histogram summarizing TUNEL assay results shown in **D**. The mean percentages (± SD) of TUNEL-positive cells were shown. **F** In vivo SUMOylation assay of HA-PML using 293 T cells (see Figure S8A**;** MATERIALS AND METHODS). **G** In vivo SUMOylation assay of endogenous PML using 293 T cells (see MATERIALS AND METHODS). **H** Immunofluorescence images of cells transfected with the indicated siRNA and/or constructs are shown (see MATERIALS AND METHODS). Arrows, damaged nuclei. Arrowheads, low DAPI nuclei. **I** The formation of PML NB is induced by only MAD2 overexpression, and significantly reduced by TSG101 depletion, but is further abolished under MOID conditions. A histogram summarizing the quantitation of PML signals in interphase of **H**. Sample numbers correspond to the ones shown in Fig. 4H and Tabe S5. Signals were normalized with Ctrl cells (1st column from left), and the mean percentages (±SEM) are shown. **J** A histogram summarizing the quantitation of nuclear MAD2 signals including interphase foci of **H**. Sample numbers correspond to the ones shown in Fig. 4H and Tabe S5. Signals were normalized with Ctrl cells (1st column from left), and the mean percentages (±SEM) are shown. **K** A histogram summarizing the Pearson correlation coefficient of the colocalization between MAD2 nuclear foci and PML signals of **H**. Sample numbers correspond to the ones shown in Fig. 4H and Tabe S5. The mean values (±SEM) are shown.

Thus, we constructed a major SUMOylation mutant (K160R) and coiled-coil domain deletion mutant (dCC) of PML isoform 1, and evaluate the effect of PML SUMOylation of these mutants (Fig. 4A and B). Both K160R and dCC showed the loss of SUMOylation, but the K160R left a small amount of SUMOylation and/or other types of post-translational modification(s) (Figs. 4C, S7D, and S7E), which can be presumably SUMOylation(s) of other site(s). Consistent with the previous reports and our data, dCC showed the loss of the formation of PML NBs and diffused localization of PML in whole nuclear (Fig. S7F and S7G**)**. The MAD2-interacting motif (MIM) mutant of PML isoform 1 (Fig. 4A) was unstable and not detected in western blot (Fig. 4B, samples 6 and 7, 2 independent bacterial plasmid clones are shown), indicating that MAD2-PML interaction may be required for PML protein stability, consistent with our data showing that MAD2 depletion results in protein destabilization of PML (Fig. S6C; Supplemental Result 1).

We found that two types of HA-PML mutants (dCC or K160R) were able to induce MOID effectively when TSG101 and endogenous PML were depleted simultaneously (Figs. 4D, E, S7H, and S7I, samples [vi]-[ix]; Table S5 for simplified sample indications). Overexpression of Flag-MAD2 and single depletion of PML also induced MOID consistently (Figs. 4D, E, S7H, and S7I, sample [x]). However, only the dCC mutant did not suppress the induction of MOID, and constitutively induced MOID (Figs. 4D, E, S7H, and S7I, samples [xi]-[xiii]). We also excluded the possibility of a dominant effect on cell death of PML dCC overexpression (Fig. S7J–S7O; Table S8; Supplemental Result 3). These data suggest that the mutant abrogated SUMOylation is able to consistently induce MOID regardless of TSG101 status, and PML deSUMOylation contributes to induce MOID.

SUMOylation status of PML protein was observed using SUMOylation assay with exogenous HA-PML immunoprecipitation (Figs. 4F and S8A). Loss of TSG101 reduced SUMOylation of HA-PML precipitants (Figs. 4F and S8A, samples 3 and 5), which is consistent with the aforementioned loss of PML (PML NBs) immunofluorescence signals and other phenotypes (Fig. S5J–S5P; Supplemental Result 1). TSG101 depletion also increased interaction between HA-PML and endogenous MAD2 (Figs. 4F and S8A, samples 3 and 5; Supplemental Result 4). These data suggests that TSG101 is necessary to maintain PML NB formation and can inhibit interaction between PML and MAD2 (Supplemental Result 4). However, in MOID condition, the binding between overexpressed Flag-MAD2 and HA-PML is reduced, and HA-PML SUMOylation is also reduced (Figs. 4F and S8A, samples 4-6; consistent with Fig. 3G), but these are rescued by TSG101 WT but not Y390F mutant (Figs. 4F and S8A, samples 7-8). Conceptually similar results were obtained from the SUMOylation assay using endogenous PML with anti-PML immunoprecipitation (Figs. 4G and S8B, and S8C).

Our immunofluorescence analysis showed heterogeneous DAPI images in MOID cells (Figs. 2E, F, and 4H, MOID; Table S5 for simplified sample indications), including normal and two types of abnormal DNA morphology: (i) damaged or condensed DNA or (ii) low DAPI signals and both often appear in the same transfectant, presumably indicating later stage of cell death (Figs. 2E, F, and 4H, arrows and arrowheads). MOID cells have lower PML signals and some lack PML nuclear bodies (Fig. S8D). MOID cells with low DAPI signals and PML loss suggest late stage of MOID (Figs. 2E, F, and 4H, and S8E). TSG101 depletion reduces PML NB formation (Fig. 4H, I, sample [3], S5J, S5N, and S8F; Supplemental Result 1), which is further abolished under MOID conditions with additional Flag-MAD2 overexpression (Fig. 4H and I, sample [4]). Loss of PML NB formation in MOID condition is rescued by exogenous TSG101 WT overexpression, but not by Y390F mutant (Fig. 4H and I, samples [5] and [6]).

An anti-MAD2 antibody (Table S3, A207) showed nuclear MAD2 foci colocalizing with PML in PML nuclear bodies (Fig. 4H[1], S8F, and S8G, control siRNA), which is enhanced by Flag-MAD2 overexpression (Fig. 4H–J, samples [1] and [2]) but decreased by TSG101 depletion (Fig. 4H and K, samples [3] and [4], S8F, and S8G), mainly because of the loss of PML NBs by TSG101-depletion as aforementioned. TSG101 depletion causes milder decrease in MAD2-PML colocalization in Fig. 4K compared with Fig. S8G, presumably due to additional vector transfection and/or transient hygromycin treatment for 2 days in Fig. 4K, considering that PML is stress response protein. However, total signals of MAD2 nuclear foci, including ones outside PML NBs, remain unchanged regardless of TSG101 status (Figs. 4H, J, S8F, and S8H), suggesting that most of the MAD2 proteins involved in cell death and survival signaling are not confined only in PML NB.

Together, MAD2 colocalizes with PML at PML NBs of which formation depends on TSG101 in normal (survival) conditions. Loss of TSG101 combined with MAD2 overexpression causes PML to be released from PML NBs through deSUMOylation, leading to MOID induction. The process of PML deSUMOylation and subsequent loss of PML NB formation play an important role in MOID induction.

### Chromosome instability correlates with MOID

Previous reports have linked the loss of function of spindle assembly checkpoint proteins and/or microtubule inhibitor treatment to programmed cell death and chromosome instability [11, 20, 21,43–45]. We therefore characterized MOID cells for mitotic cell population, interphase nuclei abnormalities, and abnormal mitotic cells, as previously described [27, 46, 47]. The mitotic cell population is elevated in viable controls with MAD2 overexpression and/or TSG101 depletion (Figs. S9A and S9B**;** we note the mitotic indexes of samples [2]–[4] are lower than in Figure S1I, and the synthetic effect of MAD2 overexpression or TSG101 depletion isn’t observed, likely due to extra pcDNA5 transfection and/or transient hygromycin treatment for 2 days). Interphase micronuclei and cytokinesis (chromosome bridge-type) cells are especially increased in MOID cells inducing chromosome instability, which is rescued by TSG101 WT overexpression but not by Y390F mutant (Figs. S9C–S9H). These data suggest that chromosome instability significantly correlates with MOID.

### MOID is concomitant with post-transcriptional/translational cell death machinery and non-canonical transcriptional regulation of oxidative stress and ATM/ATR-mediated DNA double-strand breaks (DSB) - DNA damage response (DDR)

**Fig. 5:**
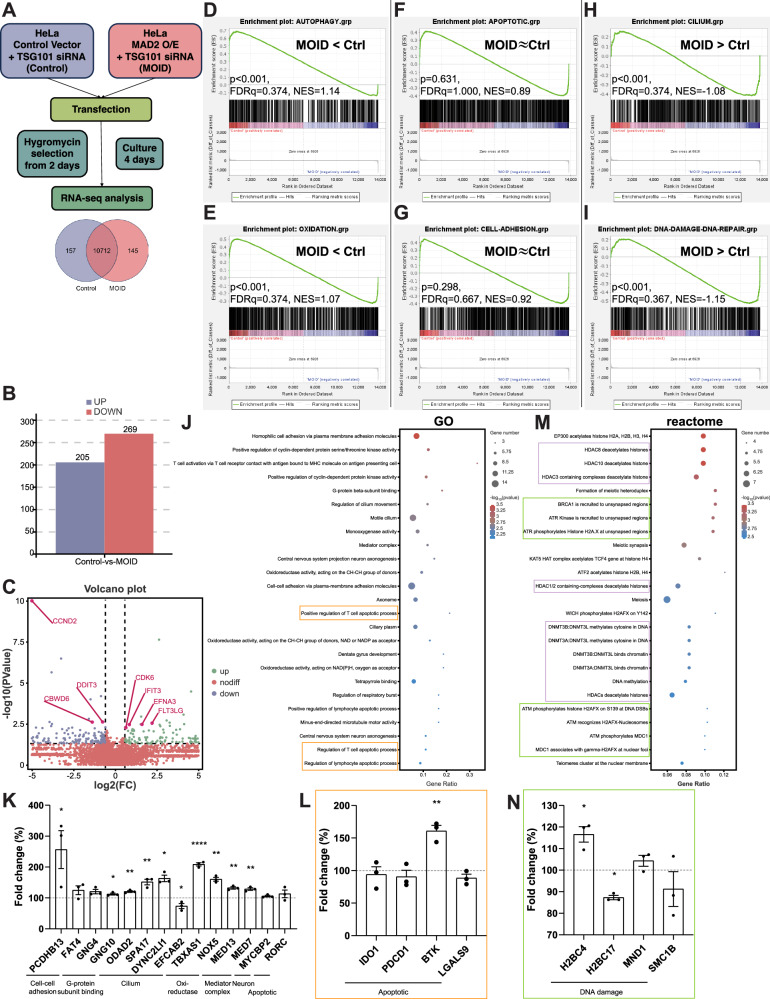
Transcriptome RNA-seq analysis of MOID. **A** Scheme of cell treatments and HeLa samples’ collection for RNA-seq. Venn diagram shows the gene number of shared or unique expressed genes in each group (submitted sample number = 2). **B** Histogram of the gene number of both up or downregulated genes in the matched MOID group compared with the control group. **C** Volcano plot of differentially expressed genes in the MOID group compared with the control group. Cut-off values, *p* ≤ 0.05, FC ≥ 1.5. **D–I** Gene set enrichment analysis (GSEA) of genes’ set that hit the following keywords: **D** autophagy, **E** oxidation, **F** apoptotic, **G** cell-adhesion, **H** cilium, **I** DNA damage and/or DNA repair. P- and FDRq-values and NES were indicated. **J** Gene Ontology (GO) enrichment analysis showing top 25 biological processes of 76 genes affected in the MOID group. Orange rectangle indicates apoptotic process. **K** Evaluation of differentially expressed genes (with most significant P value/each process and reads counts ≥ 10) of top 25 biological processes in GO enrichment analysis by RT-qPCR (*n* ≥ 3 replicates). The mean ± SEM. is shown. **L** Evaluation of differentially expressed genes (with most significant *P* value and reads counts ≥ 10) in apoptotic related gene sets as shown in (**J**, orange rectangle) by RT-qPCR (*n* ≥ 3 replicates). The mean ± SEM. is shown. **M** Reactome analysis showing 25 pathways of 11 genes affected in the MOID group. Violet rectangle indicates HDACs/DNMT3s-related pathway. Green rectangle indicates ATM/ATR/BRCA1-related pathway. **N** Evaluation of differentially expressed genes (with most significant *P* value and reads counts ≥ 10) in ATM/ATR/BRCA1 related gene sets as shown in (M, green rectangle) by RT-qPCR (*n* ≥ 3 replicates). The mean ± SEM. is shown.

We conducted transcriptome RNA-seq analysis of coding mRNA expression levels to investigate how transcription of genes are regulated in MOID. For simplicity, we compared the coding mRNA levels between the MOID sample (Table S5, sample [4]; Fig. 5A and S10A–S10E) and the sample depleted with TSG101 siRNA as the control (Table S5, sample [3]). Because these samples showed similar features of the pattern of MitoTracker signals (Fig. 2H) and MAD2-PML colocalization in interphase (Fig. 4K) except MAD2 mRNA level and MOID phenotypes (i.e., cell fate). We hypothesize that the difference between these two samples manifests the most difference in the cell fate, and find out which gene’s coding mRNA is responsible for the difference in the cell fate.

We identified a relatively higher number of downregulated genes in the MOID sample (Fig. 5B; 205 upregulated vs. 269 downregulated genes with fold difference > 1.5 and *P* value < 0.05). We performed further RT-qPCR analysis for the top 7 genes with the lowest P values in the total individual genes (Fig. 5C and S10E), then 3 genes (FLT3LG, EFNA3, and CDK6) and 1 gene (CCND2) were verified as genes significantly up and downregulated genes in the MOID sample than the control, respectively (Figures S10F and S10G; Table S7). In gene set enrichment analysis (GSEA), the enrichment plot of the MOID sample shows a lower score in autophagy and oxidation-related genes (Fig. 5D and E), while not a large difference in apoptotic and cell-adhesion-related genes compared with the control (Fig. 5F and G). We obtained higher enrichment scores of cilium and DNA damage and/or DNA repair-related genes of the MOID sample compared with the control (Fig. 5H and I). These data suggest that autophagy, oxidation, cilium, and DNA damage-DNA repair are transcriptionally impaired in MOID.

In the gene ontology (GO) analysis (Fig. 5J), the mRNA of total 76 genes showed a change in fold difference of more than 1.5 in top 25 ontologies (based on P value rank) (Fig. 5J; data not shown). We focused on these top 25 ontologies and performed further RT-qPCR analysis for at least one gene with the lowest P value and a read count of 10 or more in each ontology (Fig. 5K; Table S7). Genes that function in Cell-cell adhesion (PCDHB13), G-protein subunit binding (GNG10), Cilium, Oxi-reductase (TBXAS1 and NOX5), and Mediator complex (MED13 and MED7) are significantly dysregulated (Fig. 5K). Our results of GO analysis also demonstrated that 3 apoptotic processes ranked in top 25 ontologies (based on P value rank) (Fig. 5J, orange rectangle). Our results of RT-qPCR analysis showed upregulation of BTK mRNA, which was previously reported to be involved in apoptotic dual function [48] (Fig. 5L; Table S7).

We found many pathways related to HDACs and DNMT3s in the top 25 reactome pathways (based on P value rank) (Fig. 5M, violet rectangle). HDACs and DNMT3s are known protein interactors of PML and DAXX, and these interactions are important for the transcriptional repression of target genes involved in apoptotic regulations [49, 50]. These data might suggest that MOID can be regulated through the repression of anti-apoptotic genes by HDACs and/or DNMT3s with PML and/or DAXX co-repressors. However, we did not observe significant up and downregulations of canonical apoptotic and anti-apoptotic genes, respectively, of previously reported or suggested target genes of DAXX/PML/AIFM1/HDACs and autophagy (Table S6; data not shown). These data suggest that the MOID can also be under post-transcriptional/translational machinery (see Discussion). On the other hand, we have found many pathways related to the phosphorylation of Histone H2As by ATM/ATR/BRCA1 (Fig. 5M, green rectangle), supporting that the MOID is concomitant with the transcriptional regulation of DNA double-strand breaks (DSBs) and/or DNA damage response (DDR). In total, the mRNA of 11 genes showed a change in fold difference of more than 1.5 in top 25 reactome pathways (Fig. 5M and data not shown). Consistently, the dysregulations of two genes that function in DNA damage (H2BC4 and H2BC17) were verified by RT-qPCR (Fig. 5N; Table S7). Together, we identified coding mRNA regulated in MOID induction, supporting that MOID is concomitant with post-transcriptional/translational cell death machinery and non-canonical transcriptional regulation of oxidative stress and ATM/ATR-mediated DSB and/or DDR.

### ROS and autophagy are activated in MOID, and mitochondrial-associated membrane (MAM) and PML localized at ER-MAM can be a crucial intersection for MOID signaling

**Fig. 6:**
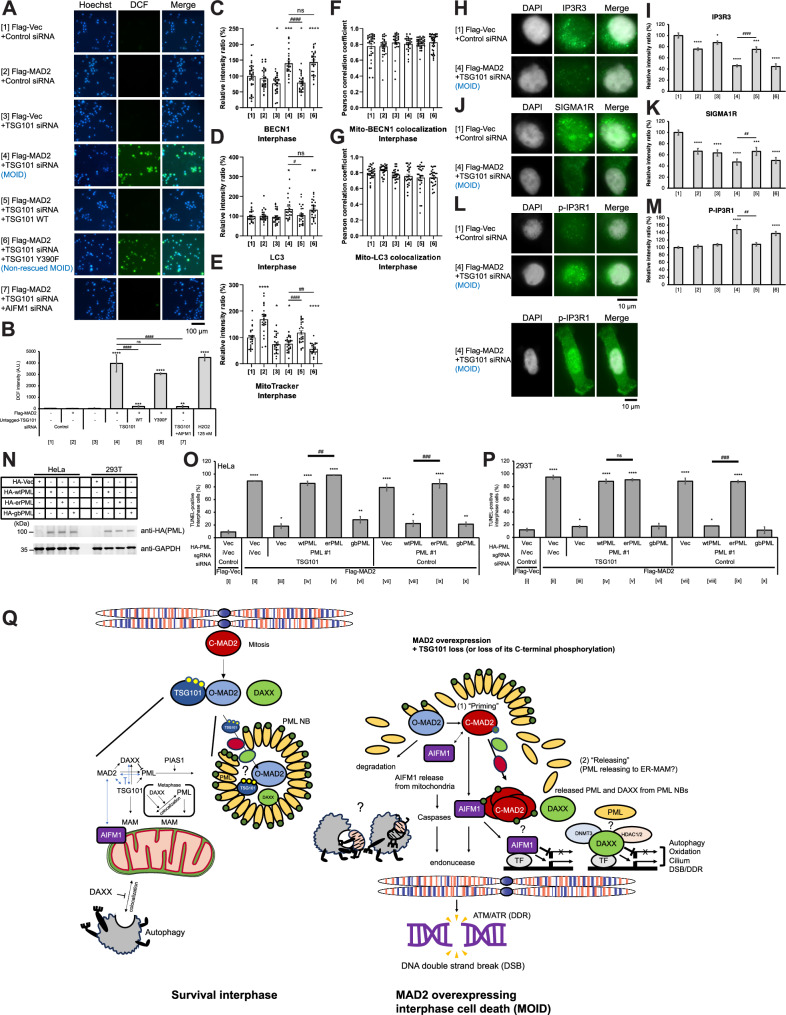
ROS and autophagy are activated in MOID, and mitochondrial associated membrane (MAM) and PML localized at ER-MAM can be a crucial intersection for MOID signaling. **A** ROS detection through fluorescent DCF using DCFH-DA probe (see MATERIALS AND METHODS). **B** A histogram summarizing ROS detection (fluorescence DCF intensity) as shown in **A**. HeLa cells were transfected with the indicated constructs, and pellets were collected 96 h after the transfection as TUNEL-positive MOID cells were observed. The mean percentages (± SD) were shown (see MATERIALS AND METHODS, Statistical Analysis). **C** Quantitation of interphase intracellular signals of anti-BECLIN1 (BECN1) in interphase. HeLa cells were transfected as samples [1]-[6] in **A** and **B** and cultured at 37 °C for 96 hours, which is the timing when TUNEL-positive MOID cells were observed. Samples were observed with an indirect fluorescence microscope using an anti-BECN1 antibody (Tabe S3). **D** Quantitation of interphase intracellular signals of anti-LC3B in interphase. HeLa cells were transfected as samples [1]-[6] in (**A** and **B**) and cultured at 37 °C for 96 hours, which is the timing when TUNEL-positive MOID cells were observed. Samples were observed with an indirect fluorescence microscope using an anti-LC3B antibody (Tabe S3). **E** Quantitation of interphase intracellular signals of MitoTracker in interphase (see MATERIALS AND METHODS). HeLa cells were transfected as samples [1]-[6] in (**A** and **B**) and cultured at 37 °C for 96 hours, which is the timing when TUNEL-positive MOID cells were observed. **F** Pearson correlation coefficient between fluorescence signals of MitoTracker and anti-BECN1 in interphase (see MATERIALS AND METHODS). **G** Pearson correlation coefficient between fluorescence signals of MitoTracker and anti-LC3B in interphase (see MATERIALS AND METHODS). **H-M** Immunofluorescence signals of ER-MAM markers are associated with the MOID. Representative immunofluorescence images and histograms summarizing quantitation of interphase intracellular signals of (**H** and **I**) anti-IP3R3, (**J** and **K**) anti-SIGMA1R, and (**L** and **M**) anti-phospho-IP3R1 (Ser 1756) (see MATERIALS AND METHODS). **N** Western blot analysis of HA-PML WT and mutants (erPML and gbPML). **O** and **P** erPML mutant failed to rescue this MOID induction with Flag-MAD2 overexpression and single PML depletion. A histogram summarizing TUNEL assay of (**O)** HeLa and (**P**) 293 T cells performed as described in Fig. 1C (see MATERIALS AND METHODS). The mean percentages (± SD) of TUNEL-positive cells were shown. **Q** A hypothetical model depicting that PML-MAD2 interaction at PML NBs and formation of PML NBs regulates MOID. (Left) In survival interphase, PML, DAXX, MAD2, and TSG101 maintain a close dependence and contribute mutually to protein stability sustaining inter-organelle integrity. Note that previous reports suggested that PML can be auto-SUMOylated [40, 41, 68] and/or SUMOylated by PIAS1 SUMO E3 ligase [69]. O-MAD2-specific binding of TSG101 and TSG101 function inside PMN NB are hypothetical. Black arrows indicate activatory or inhibitory direction based on our results of immunofluorescence and protein stability assay or other reports. Black double-head arrows indicate colocalization based on our immunofluorescence results. Blue arrows indicate inhibitory or interactive relationships based on our immunoprecipitation and/or in vivo SUMOylation assays. (Right) The series of our results propose at least 2 steps of MOID induction process (1): “Priming” and (2) “Releasing”. Mechanisms and roles of MAD2 SUMOylation/multimerization and autophagy are open questions. Transcriptional roles of AIFM1 and PML-DAXX collaboration are unknown. See text for other details.

Based on our results of RNA-seq analysis, we hypothesized that ROS and autophagy are activated in MOID. Indeed, we detected ROS induction through fluorescence signals of 2’- 7’dichlorofluorescein (DCF) using 2’-7’dichlorofluorescin diacetate (DCFH-DA) probe specifically in MOID samples (Fig. 6A and B). We also detected increased anti-BECLIN1 (BECN1: a regulator of autophagic cell death) and anti-LC3 immunofluorescence signals under the MOID condition that consistently showed the decreased (and diffused) MitoTracker signals (Fig. 6C–E; see also Fig. 2H), although no significant changes of the colocalization was observed between MitoTracker and anti-BECN1 signals, neither between MitoTracker and anti-LC3 signals (Fig. 6F and G). These data suggest that ROS and autophagy are activated in MOID, but apparently, we did not detect canonical mitophagy (i.e., colocalization of mitochondria and autophagy proteins; see Discussion).

Giorgi et al. reported that PML is specifically enriched at the endoplasmic reticulum (ER) and at the mitochondria-associated membranes (MAM), i.e., ER-MAM, signaling domains involved in ER-to-mitochondria calcium ion (Ca^2+^) transport and in induction of apoptosis [51]. They revealed that PML associated with large molecular complexes containing IP3R, suggesting that PML plays a crucial role in regulating IP3R phosphorylation and Ca^2+^ release from ER. Thus, we are intrigued to test if the ER-MAM protein, including calcium ion (Ca2 + ) channel IP3Rs, involved in MOID, and study how PML at ER-MAM functions in MOID. Our analysis revealed that MOID is highly associated with changes of immunofluorescence signals of ER-MAM proteins: while the intensities of IP3R3 and SIGMA1R are negatively correlated (Fig. 6H–K), the intensity of phospho-IP3R1 (Ser 1756) is positively correlated with MOID induction (Fig. 6L and M). Unexpectedly, nuclear signals, rather than ER-MAM signals, of phospho-IP3R1 (Ser 1756) is more intensified in MOID condition (Fig. 6L, and data not shown). Next, we also constructed chimeric HA-PML mutants: one localizes at ER-MAM (erPML), and another localizes at Golgi body (gbPML) as previous reported [51, 52] (Fig. 6N; Table S2). We overexpressed these constructs under the depletion of endogenous PML with CRISPRi, and performed TUNEL assay as aforementioned PML dCC mutant (see also Figs. 4D, E, S7H, and S7I). We found that HA-erPML mutant induced MOID more effectively than PML WT (wtPML) when TSG101 and endogenous PML were depleted simultaneously in HeLa cells (Fig. 6O, samples [iv] and [v]; Table S5 for simplified sample indications), while HA-gbPML mutant did not (Fig. 6O and P, samples [iv] and [vi]). Overexpression of Flag-MAD2 and single depletion of PML also induced MOID consistently (Fig. 6O and P, samples [vii]). However, only the erPML mutant failed to suppress the induction of MOID, and constitutively induces MOID (Fig. 6O and P, samples [viii]-[x]), which is mimical phenotypic pattern as aforementioned PML dCC mutant. These data suggest that PML localized at ER-MAM can be a crucial intersection for MOID signaling.

## Discussion

Consistent with our results, several studies previously suggest that MAD2 functions as an oncogene or a modelator of DNA damage response in nonmitotic phase [12, 53]. Other reports suggest that MAD2 functions in the apoptosis or senescence signaling pathway [12, 53]. In this study, we have clearly shown that the depletion of TSG101 showed synthetic dosage lethality (SDL) in MAD2-overexpressing interphase cells, and we refer to this cell death as MOID (MAD2-overexpressing interphase cell death) revealing at least 22 different characteristics of MOID (Table S8). We observed a similar SDL phenotype in MAD2-overexpressing and TSG101-depleted interphase cells in four different types of cells (HeLa, 293 T, A549 cells, and human primary skeletal muscle cells [HSkMCs]), suggesting that MOID occurs commonly in MAD2-overexpressing human cells. MAD2 overexpression rate required for MOID induction varies among different cell lines and different our experiments, but exogenous Flag-MAD2 level was within ca. 1-22 fold of endogneous MAD2 protein level (i.e., total 2-23 fold of total MAD2 protein level with TSG101 depletion induced MOID) (Figs. 3G, 4G, S2D, S2G, S4F, S8A, and S8C). Although the results of our IP-mass spectroscopy and IP-western blot analysis demonstrated the basal interaction of Flag-MAD2 with endogenous p53 in p53-activated cells, MOID occurs regardless of activation of the p53 gene. The significance and functions of MAD2-p53 interaction in p53-activated cells remain to be studied. These MAD2-p53 functions could not be limited in the MOID-activation pathway but could be extended to comprehensive cellular pathways, such as SAC activation, senescence, or other tumor-suppressing functions. Our results also revealed that MOID is AIFM1-dependent and caspase-activated cell death. In our IP-mass spectrometry analysis and PSEA, the enrichment plot of the Flag-MAD2 immunoprecipitants shows a higher score in mitochondria-related genes compared with the control (Fig. 2C and D). We also identified both MAD2 and AIFM1 peptides in Flag-PML immunoprecipitates (Table S4), and relatively higher number of peptides derived from mitochondrial proteins interact with Flag-PML, DAXX, TSG101, and MAD2 proteins compared with other control proteins (Figures S5A and S5B), suggesting that MAD2 functions in both PML NBs and mitochondria. PML, DAXX, or TSG101-depleted interphase cells showed consistently impaired fluorescence signals of MitoTracker and PML NBs. In neuronal axons, TSG101 regulates mitochondrial biogenesis in a noncanonical, ESCRT-independent but PGC-1ɑ/Nrf2-dependent manner [54]. We detected that TSG101 and the components in the AIF-PML-DAXX axis are closely interdependent in protein stability, and they also regulate mitochondria in survival interphase (Fig. 6Q, left). These data suggest the regulation and collaboration between mitochondria and PML NBs through the AIFM1-PML-DAXX axis for MOID induction.

MOID induction depends on PML-DAXX function concomitant with mitochondrial AIFM1-release, but the inductive or suppressive role of PML-DAXX during MOID is TSG101-dependent. However, an in-depth study remains to clarify the mechanism of how TSG101 determines the cell fate through of PML and DAXX. We found that loss of C-terminal phosphorylations of TSG101 and C-MAD2-overexpression contribute to induce MOID. Currently, there are no reports about the functional roles of TSG101 phosphorylations and the effect of an open (O-MAD2) and a closed (C-MAD2) conformations in MAD2-overexpressing cancer cells. The result of our TUNEL assay suggests that C-MAD2-overexpression is required to induce MOID. Previous studies suggest that a dramatic conformational rearrangement of the MAD2 protein regulates the function of MAD2 in the spindle assembly checkpoint (SAC) [3–6,55]. A stable kinetochore-anchored complex of MAD1 heterodimerizes with C-MAD2. This C-MAD2 serves as a template that recruits an O-MAD2 from the cytoplasm via asymmetric dimerization, which is subsequently bound to Cdc20 to inhibit APC/C^CDC20^^4,6^. However, abnormal overexpression of C-MAD2 conformation is not yet confirmed in cancer patients’ samples. Even if it was confirmed, finding the endogenous regulatory mechanism that induces C-MAD2 overexpression during cancer development is still unclear. These studies would be an intriguing future direction to eradicate MAD2-overexpressing cancer. We also observed the colocalization of MAD2 with PML NBs, and the series of our results propose at least 2 steps of MOID induction process: (1) priming with PML deSUMOylation in which O-MAD2 is converted to C-MAD2 and binding of C-MAD2-AIFM1 occurs, (2), releasing of PML-DAXX from PML NBs with MAD2 SUMOyaltion/multimerization (Fig. 6Q, right). Our results using HA-tagged and/or untagged TSG101 WT vs. Y390F replaced with ca. 85% endogenous TSG101 (Fig. 3I–L, S6F–S6J, and S7A–S7C) also suggest that TSG101 localizes at PML NBs in interphase, and TSG101 Y390 phosphorylation is presumably required for localization of TSG101 to PML NBs in survival cells. Furthermore, overexpressed O-MAD2 primarily binds to phosphorylated TSG101, while C-MAD2 favors non-phosphorylated TSG101. A part of MAD2 colocalizes with PML at PML NBs (Figs. 4H, K, S8F, and S8G), and the PML release from PML NBs through PML deSUMOylation contributes to induce MOID (Fig. 4D–G, S7H, S7I, and S8A–S8C). Because overexpression of phosphorylation-deficient mutant (e.g., Y390F) does not rescue the MOID (Fig. 3C, D, etc.), we also speculate that phosphorylated TSG101 may block the priming conversion of O-MAD2 to C-MAD2 for the MOID induction (Fig. 6Q (1), “Priming”) interacting with O-MAD2. Since sample 6 of Figs. 3I, K, and S7A showed the interaction of overexpressed Flag-C-MAD2 and TSG101 Y390F in MOID condition, it could be plausible to describe that TSG101 release from overexpressed C-MAD2 could not be necessary for MOID induction, rather loss of Y390 phosphorylation combined with overexpressed C-MAD2 could be required for MOID induction (Fig. 6Q, right). However, we could not exclude the possibility that in MOID condition C-MAD2 is not able to revert back to O-MAD2 after proper SAC silencing at G1 entry, instead of the conversion at PML NBs during homeostatic G1 phase. Because chromosome instability correlates with MOID (Figure S9), despite no synergistic elevation of mitotic index or mitotic TUNEL positive cells with MAD2 overexpression and TSG101 depletion is observed (Figures S1I, S1J, S9A, and S9B), we could not exclude neither the possibility that mitotic abnormalities or SAC defects impact post-mitotic cell survival. How TSG101 contributes to the conversion between O-MAD2 and C-MAD2 and how it is localized and functions in PML NBs and the cell cycle is an interesting future research direction.

We identified coding mRNA regulated in MOID induction through our transcriptome RNA-seq analysis and verification with RT-qPCR. The role of PML NBs in MOID may contradict ones in Fas-induced apoptosis previously reported where the formation of PML NBs is anti-apoptotic [56] but partially consistent with one in B cell apoptosis where PML gene loss suppresses apoptosis [57]. In fact, we did not detect any mRNA of Fas ligand/receptor in our RNA-seq using HeLa cells **(**Table S6; data not shown), therefore, MOID is presumably a different type from Fas-induced apoptosis. Some reports suggest that DAXX represses gene transcription of anti-apoptotic genes but such suppressive activity is reduced when DAXX is sequestered into PML NBs [58, 59], consistent with our model of MOID induction (Fig. 6, right). Other examples show that one protein works in opposite ways in different types of apoptosis (e.g., Bruton’s tyrosine kinase [BTK] [48]), or that different types of apoptosis and/or cell death (e.g., necroptosis, pyroptosis, etc.) compete and inhibit each other. Solving more complex cell death mechanisms, including such competitions and balancing of different cell death pathways, will be a challenge in the future.

The transcriptional regulation of DSB and/or DDR mediated by ATM/ATR is impaired in MOID, suggestive of concomitant DNA damage. However, we did not observe significant up and downregulation of canonical apoptotic and anti-apoptotic genes, respectively, of previously reported or suggested target genes of DAXX/PML/AIFM1/HDACs and autophagy (Table S6). However, our results of gene set enrichment analysis (GSEA) suggest that autophagy, oxidation, cilium, and DNA damage-DNA repair are impaired in MOID. These data suggest that MOID is concomitant with post-transcriptional/translational cell death machinery and non-canonical transcriptional regulation of oxidative stress and ATM/ATR-mediated DSB and/or DDR. Indeed, we demonstrated that ROS and autophagy were activated in MOID, although we did not detect canonical mitophagy (i.e., colocalization of mitochondria and autophagy proteins) (Fig. 6A–G). It is an open question whether MOID is classified as mitophagy or a non-canonical type of mitophagy that does not involve the colocalization between LC3 (or BECN1) and mitochondria (Fig. 6Q, right).

Our analysis also revealed that MOID is highly associated with changes of immunofluorescence signals of ER-MAM proteins and PML localized at ER-MAM can be a crucial intersection for MOID signaling (Fig. 6H–P). As for post-transcriptional/translational cell death machinery, it is a formidable challenge to determine the functional linkage of many catastrophic events in a short period of time, such as MAD2 SUMOylation/multimerization and ER-MAM signaling that we detected in MOID. Nevertheless, together our results provide new insights into the mechanisms underlying MOID and also sugget that future development of TSG101 as well as MAD2 inhibitors can be an effective therapy for killing MAD2-overexpressing tumor cells. In the future, verification of MOID using actual human patient samples and the development of clinical research, including accurate drug screening, are strongly expected. The clinical application of MOID can be also useful for the treatment of non-dividing (e.g., senescent) cancer cells with low MAD2 expression that is resistant to microtubule inhibitors (e.g., paclitaxel and vincristine) as well as other cytotoxic and DNA damaging agents (cisplatin and γ-irradiation) [53].

## Materials and Methods

### Lead contact and materials availability

Further information and requests for resources and reagents should be directed to and will be fulfilled by the Lead Contact, Yohei Niikura (niikura@nju.edu.cn). There are restrictions to the availability of cell lines and recombinant DNA (plasmids) due to material transfer agreement (MTA) at the Model Animal Research Center, Medical School of Nanjing University.

### Experimental model and subject details

#### Statistical analysis

In this study, all statistical tests are justified and all data assumptions are met as normal distribution. For line charts of zebrafish and mouse xenografts, we used the one-way ANOVA. For all the other tests, we used the Student’s t-test. For both tests, we note *P* values with these indications within histograms in each figure: (****) *P* < 0.0001, (***) *P* < 0.001, (**) *P* < 0.01, and (*) *P* < 0.05; (####) *P* < 0.0001, (###) *P* < 0.001, (##) *P* < 0.01, and (#) *P* < 0.05. Asterisks (*) are used to compare with 1st column from left (e.g., Control siRNA, iVec, plus Vector[s]-transfectant or just Vector[s]-transfectant) in each subgroup (e.g., same cell line or transfectant) within a histogram. Hashtags (#) are used to compare with MOID sample (Table S5, MOID or sample [4]) in each subgroup (e.g., same cell line or transfectant) within a histogram. We note that ± SD values between MitoTracker-normal and -diffused cells are identical for the histogram summarizing the pattern of MitoTracker (MitoTracker™ Orange CMTMRos, ThermoFisher Scientific, M7510) signals (Fig. 2H).

In all experiments involving any cells in this study, cells were randomly spread in each cell line before transfection, and randomly processed/treated in each transfectant group after transfection, unless otherwise noted. In all experiments with any cells in this study, unless otherwise noted, the phenotypic analysis after transfection was conducted in a blinded manner by the analyst. Specifically, each group of transfectants was assigned a number unrelated to the sample, and the analyst removed the blinding only after completing the analysis.

#### Cell culture and transfection

Cell lines used in this study are listed in Table S9. HeLa, 293 T, human skeletal muscle primary cells (HSkMC), Rh30 (generously gifted from Yue Shen, Nanjing Medical University), A549, and A549 p53 KO cells (generously gifted from Jianghuai Liu, Model Animal Research Center, Medical School of Nanjing University) were cultured in high-glucose Dulbecco’s modified Eagle’s medium (Biological Industries or HyClone, China) with 10% fetal bovine serum (FBS) (Gibco, Australia or ExCell Bio, China). Cells were grown at 37°C in 5% CO_2_ in a humidified incubator. Cells were transfected with annealed double-stranded siRNAs (Table S1) and/or constructs (Table S2) by using Lipofectamine 3000 (Invitrogen), Lipofectamine RNAiMAX (Invitrogen), and/or linear polyethyleneimine (PEI)(YEASEN, China) [60]. For PML knockdown (KD) and DAXX KO, puromycin (0.5 μg/ml) was added to the culture medium at 4 days after transfection. For MOID induction, TSG101 #2 (Table S1) was consistently used unless otherwise indicated. For the colony outgrowth assay, the zebrafish xenograft, and the pattern analysis of MitoTracker (MitoTracker™ Orange CMTMRos, ThermoFisher Scientific, M7510) signals, PML NB-negative cells, and low DAPI/PML-negative cells, cells were selected with hygromycin (100 μg/ml) (YEASEN, China) 48-96 hr after transfection of siRNA and/or constructs. For all siRNA transfections, final concentration of siRNA was 50 nM. Only for SNF8 and CHMP2A siRNAs, 100 nM and 250 nM was used, respectively. For MitoTracker analysis, MitoTracker™ Orange CMTMRos (ThermoFisher Scientific, M7510; 100 nM) was incubated for 45 min before the sample fixation.

#### Immunoblotting

Antibodies used in this study are listed in Table S3. The method of immunoblotting has been described in detail previously [21, 27, 47, 61]. The Odyssey CLx Infrared Imaging System (LI-COR Biosciences) and/or Amersham Imager 600 (GE Healthcare Life Sciences) were used for the detection of coimmunoblotting. For MOID samples, cell pellets were collected primarily at 96 hours after transfection (when TUNEL positivity is observed) unless otherwise indicated. Cells were suspended in denaturing buffer A1 (20 mM Tris-HCl pH 7.4, 50 mM NaCl, 0.5% Nonidet P-40, 0.5% deoxycholate, 0.5% SDS, 1 mM EDTA, and InStabTM protease inhibitor cocktail [YEASEN, China]) [21, 27, 47, 61] or buffer used in the immunoprecipitation assay. The cell suspension was sonicated, frozen in liquid nitrogen, and thawed (freeze-thaw pro- cess). Before electrophoresis, cell lysates were mixed with SDS sample buffer [21, 27, 47, 61]. Proteins were subjected to western blot analysis [21, 27, 47, 61] with the indicated antibodies (Table S3). GAPDH protein was used as a loading control. The intensity of band signals were quantitated by Image Studio Version 5.2.5 Software (LI-COR Biosciences, Lincoln, NE), and/or ImageJ (NIH, Bethesda, MD).

### TUNEL assay

TUNEL assay was performed as described previously with the following minor modifications [20, 61]. TUNEL assay was performed by using a TUNEL BrightGreen Apoptosis Detection Kit (Vazyme). For DAXX KO, the cells were infected with DAXX KO lenti-virus, and 48 h later siRNAs and/or constructs of other genes were co-transfected. For MOID induction, TSG101 #2 (Table S1) was consistently used. Cells were cultured with puromycin (0.5 μg/ml) between the 4th day and the 6th day after the DAXX KO lenti-virus infection. TUNEL assay was performed 6 days after the DAXX KO lenti-virus infection. For PML CRISPRi, PML sgRNA vector, siRNAs, and/or constructs of other genes were co-transfected. Cells were cultured with puromycin (0.5 μg/ml) between the 4th day and the 6th day after the co-transfection. TUNEL assay was performed 6 days after the DAXX KO lenti-virus infection as aforestated. For the siRNA of the other targets, siRNAs and/or constructs of other genes were co-transfected. TUNEL assay was performed 96 h after the co-transfection as aforestated. Cells treated with DNAse (50 μg/ml [SIGMA] for 10 min as indicated in figure legends) after fixation is shown as a control to verify TUNEL positivity.

Before applying the recombinant TdT enzyme of the TUNEL BrightGreen Apoptosis Detection Kit (Vazyme, China), cells were fixed, permeabilized, and incubated with 1/1000 diluted anti-Flag rat monoclonal primary antibody (Table S3, A128; Invitrogen, MA1-142) for 1 h at 37°C as previously described [62] After the cells were washed 3 times with KB (10 mM Tris HCl pH 7.5, 150 mM NaCl, 0.5% bovine serum albumin), they were incubated with the recombinant TdT enzyme in 1 × Equilibration Buffer (Vazyme, China) mixed with 1/200 diluted fluorescent secondary antibodies (Table S3; Jackson ImmunoResearch Laboratories, 112-165-167) for 1 h at 37°C. Slides were washed 3 times with KB and then incubated in KB containing 0.5 μg/ml DAPI (Sangon Biotech, China).

Samples were examined under a fluorescent microscope (see Image Acquisition, Processing, and Quantitation). For Flag-MAD2 overexpressing cells, more than 100 interphase Flag+TUNEL+ (double positive) cells in 3 independent experiments were counted. For Flag-vector (Flag-Vec) overexpressing or DNAse-treated cells, more than 300 interphase TUNEL-positive cells in 3 independent experiments were counted considering their Flag-negative signals and approximate transfection efficiency (ca. more than 50%) of overexpression vector.

### Colony outgrowth assay

Colony outgrowth assay was performed as described previously with the following minor modifications [46]. Colony outgrowth assay was performed as described previously with the following minor modifications [46]. Cells (1.8 × 10^5^) were plated in a 6-well plate. 18 h later, plasmids and/or siRNA are transfected as descript in Cell Culture and Transfection. The cells were selected with 100 µg/mL hygromycin B (YEASEN, China) for 2 days from 48 h post transfection. After the treatment, cells were collected and seeded in a 6-well plate with density 1.0 × 10^3^ cells/well of 6-well culture plate. Cells were grown 10 days after plating, and colonies were fixed for 10 min in methanol and stained for 10 min in a crystal violet solution (2.3% crystal violet, 0.1% ammonium oxylate, 20% ethanol; Sangon, China). The number of colonies was counted with the ImageJ software (NIH, Bethesda, MD).

### Zebrafish xenograft

Zebrafish (Danio reiro) and embryos were raised in accordance with standard procedures [63]. Adult fishes undergo a light-dark circle of 14 h (light)/10 hours (dark). Adult fishes aged 2 months to 1 year were crossed to produce embryos and larvae. Wild-type (WT) TU (Tübingen) zebrafish, with a random mix of males and females at 0-8 dpf, were used in this study. Embryos and larvae were cultured in E3 medium (5 mM NaCl, 0.17 mM KCl, 0.33 mM CaCl_2_, 0.33 mM MgSO_4_; Sangon, China) at 28°C, and 0.2 mM N-phenylthiourea (PTU; Rhawn, China) was applied to prevent pigment formation from 1-day post-fertilization (dpf) in order not to interfere with imaging. After the transfection and MOID-induction on culture plate, human cell lines were selected with selected with 100 µg/mL hygromycin B (YEASEN, China) for 2 days from 48 h post transfection, and labeled with CFDA-SE (Shyuanye, China) at a concentration of 5 µM according to the manufacturer’s instructions.

Zebrafish embryos at indicated developmental stages in each experiment were anesthetized with 0.003% tricaine (D&B, China) and placed on a 6 cm Petri dish coated with 3% agarose. Before transplanation, all fishes with same genomic background are randomly spread in different groups.

Human cell transfectants were marked with [1]-[4]/[1]-[6] (Table S5, Fig. 1H–J and S3D–G) and transplanted to all animals at approximately same time (less than 2 hours difference). The single-cell suspension, formulated into the certain density of 5 × 10^7^ cells/ml was injected into perivitelline space by microinjection. Each embryo bore about 300-500 cells. Suitable injected (survival) embryos were selected after 24 h post-injection (hpi). Selected embryos were placed at 32°C for subsequent experiments. Images were taken at 2 days post injection (dpi), 4 dpi, and 6 dpi. Dead fishes before 6 dpi were excluded for the final statistical analysis. The area of the xenografts was measured with the ImageJ software (NIH, Bethesda, MD), and the volume of the xenografts was calculated by the following formula (V = 1/2 × L × W^2^; V, Volume; L, Length; W, Width). No blinding was performed during the xenograft.

### Mouse xenograft

NCG mice (Mus musculus) are all obtained from GemPharmaTech. Corp. and cultured in specific pathogen-free (SPF) grade mice facility until sacrifice. Only male mice of a strain: NCG (NOD/ShiLtJGpt-Prkdc^em26Cd52^Il2rg^em26Cd22^/Gpt) with an age of 3-4 months were used in this study. We applied 8-10 weeks mice as the recipients of HeLa cell derived xenografts (CDX). HeLa cells were obtained after 48 h post transfection and another 48 h of hygromycin (100 μg/ml) (YEASEN, China) treatment. Before transplanation, all mice with same genomic background are randomly spreaded in different groups. Transfected HeLa cells were then washed with PBS and resuspended in 5.0 mg/mL MatriGel (Mogengel) and placed on ice before transplantation. HeLa cell transfectants were marked with [1]-[4] (Table S5, Fig. 1K–N) and transplanted to all animals at approximately same time (less than 2 hours difference). 3 × 10^6^ cell in around 100 μl of MatriGel has been injected subcutaneously to the lower back of each recipients mice. No animal exclusion was performed for the analysis. Tumor size has been measured every 2 days from the 3rd day post transplantaion. All mice are sacificed at 39 days post transplantation. Tumors have been extracted to measure the weight and photographed on the graph paper (10 × 10 mm). No blinding was performed during the xenograft.

The Ethics Committee at Model Animal Research Center of Nanjing University approved the animal protocols used in this study.

### Caspase assay

FAM-FLICA caspase assay was performed as described previously with the following minor modifications [20]. HeLa cells were transfected with siRNAs and/or constructs. Cells were cultured 95 h at 37°C, then incubated with the FAM-VAD-FMK FLICA (fluorochrome inhibitor of caspases) solution (FAM-FLICA poly caspase assay #91, Immunochemistry Technologies, LLC) for 60 min at 37°C to detect activated caspases 1, and 3–9.

After incubation with the FAM-VAD-FMK FLICA solution, cells were fixed, permeabilized, and incubated with 1/1000 diluted anti-Flag rat monoclonal primary antibody (Table S3, A128; Invitrogen, MA1-142) for 1 h at 37°C as previously described [62]. After the cells were washed 3 times with KB (10 mM Tris HCl pH 7.5, 150 mM NaCl, 0.5% bovine serum albumin), they were incubated in KB containing 1/200 diluted fluorescent secondary antibodies (Table S3; Jackson ImmunoResearch Laboratories, 112-165-167) for 1 h at 37°C. Slides were washed 3 times with KB and then incubated in KB containing 0.5 μg/ml DAPI (Sangon Biotech, China).

Samples were examined under a fluorescent microscope (see Image Acquisition, Processing, and Quantitation). For Flag-MAD2 overexpressing cells, more than 100 interphase Flag+FLICA+ (double positive) cells in three independent experiments were counted. For Flag-vector (Flag-Vec) overexpressing or DNAse-treated cells, more than 300 interphase FLICA-positive cells in three independent experiments were counted considering their Flag-negative signals and approximate transfection efficiency (ca. more than 50%) of overexpression vector.

### Protein stability assay

Protein stability assay was performed as described previously with the following minor modifications [47]. HeLa cells were transfected with PML CRISPRi vector, DAXX CRISPR KO, or MAD2 siRNA and cultured until 6 days, 6 days, or 2 days after transfection, respectively. For PML knockdown (KD) and DAXX KO, protein synthesis was inhibited by cycloheximide (CHX; 100 μg/ml) addition at 5 days after transfection (this time point is shown as 0 h). For MAD2 siRNA, protein synthesis was inhibited by cycloheximide (CHX; 100 μg/ml) addition at 1 day after transfection (this time point is shown as 0 h). For PML knockdown (KD) and DAXX KO, puromycin (0.5 μg/ml) was added to the culture medium at 4 days after transfection.

### Subcellular fractionation

Subcellular fractionations (Fig. 2I) were performed as described previously with the following minor modifications [64]. Alternatively, 0.5 ml Kimble^TM^ Kontes^TM^ pellet pestle (Fisher Scientific) with 1.5 ml RNAse/DNAse free microcentrifuge tube (Nest Biotechnology) or 2.0 ml glass dounce homogenizer (Nantong Supin Experimental Equipment Corp., China) was used for the homogenizing process, and similar results were obtained. Cells treated with staurosporin (STS, 1.0 μM), a known inducer of apoptosis, for 6 h was used as a control.

### Immunoprecipitation Assay

Antibodies used in this study are listed in Table S3. Immunoprecipitation assay was performed as described previously with the following minor modifications [21, 27, 47, 61]. To study the interaction of exogenous Flag-MAD2 with endogenous p53 (Figure S4A), 293 T cells were transfected with the indicated constructs (Table S2). Cells were cultured 48 h, collected, and lysed in denaturing buffer A1 (20 mM Tris-HCl pH 7.4, 50 mM NaCl, 0.5% Nonidet P-40, 0.5% deoxycholate, 0.5% SDS, 1 mM EDTA, and InStabTM protease inhibitor cocktail [YEASEN, China]) [21, 27, 47, 61] by a sonication and freeze-thaw process. Lysates were diluted 10 times with PBS and proteins were immunoprecipitated with the indicated antibodies (Table S3) overnight at 4°C in buffer A2 (i.e., PBS that contains 10% of buffer A1). Immunoprecipitates were washed 4 times with buffer A2, and proteins were eluted with SDS sample buffer [21, 27, 47, 61] and subjected to western blot analysis [21, 27, 47, 61] with the indicated antibodies (Table S3).

To study the interaction of exogenous Flag-MAD2 with untagged- or HA-TSG101 replacing ca. 85% endogenous TSG101 with exogenous untagged- or HA-TSG101, the immunoprecipitation was performed as described above, but cells were cultured 96 h. Cells were selected with hygromycin (100 μg/ml) (YEASEN, China) 48-96 hr after transfection of siRNA and/or constructs. Lysates were diluted 5 times with PBS and proteins were immunoprecipitated with the indicated antibodies (Table S3) overnight at 4°C in buffer A3 (i.e., PBS that contains 20% of buffer A1). Immunoprecipitates were washed 4 times with PBS, and a western blot was performed.

### In vivo SUMOylation assay

Cells were transfected with the indicated siRNA (Table S1) and/or constructs (Table S2). Cells were cultured 96 h after the transfection, which is the timing when TUNEL-positive MOID cells were observed. Cells were collected and lysed in denaturing buffer A1 (20 mM Tris-HCl pH 7.4, 50 mM NaCl, 0.5% Nonidet P-40, 0.5% deoxycholate, 0.5% SDS, 1 mM EDTA, and InStabTM protease inhibitor cocktail [YEASEN, China]) [21, 27, 47, 61] by a sonication and freeze-thaw process. Lysates were diluted 10 times with PBS and proteins were immunoprecipitated with the indicated antibodies (Table S3) overnight at 4°C in buffer A2 (i.e., PBS that contains 10% of buffer A1). Immunoprecipitates were washed 4 times with buffer A2, and proteins were eluted with SDS sample buffer [21, 27, 47, 61] and proteins in 5% of the total cell lysates (Input) and immunoprecipitates (IP) were detected by western blot analysis [21, 27, 47, 61] using the indicated antibodies. (Table S3).

### Mass spectrometry

To identify the ubiquitylation site of the Flag-tagged proteins, 293 T or HeLa cells were transfected with the indicated constructs (Table S2). Cells were collected at 48 h after transfection and lysed in denaturing buffer A1 (20 mM Tris-HCl pH 7.4, 50 mM NaCl, 0.5% Nonidet P-40, 0.5% deoxycholate, 0.5% SDS, 1 mM EDTA, and InStabTM protease inhibitor cocktail [YEASEN, China]) [21, 27, 47, 61] by a sonication and freeze-thaw process. Proteins were immunoprecipitated overnight at 4°C in buffer A1; for immunoprecipitation we used with anti-Flag (DYKDDDDK) Affinity Beads (Smart-Lifesciences, China; Table S3, A151) in buffer A1. The range of protein amount in total cell lysates was 5.7-22.0 mg and net beads amount was 25.0-62.5 μl for one sample of immunoprecipitation in our total analyses. Immunoprecipitates were washed 4 times with buffer A1, and proteins were eluted with SDS sample buffer [65], electrophoresed on an SDS-PAGE gel, and stained with Coomassie brilliant blue R-250 (Sangon Biotech, China, A610037-0005). The gel region covering total protein sizes was excised and cut out.

MS data acquisition was performed by LC- MS/MS using a nanoLC.2D (Eksigent Technologies) coupled with a TripleTOF 5600+ System (AB SCIEX, Concord, ON). First, samples were chromatographed using a 60 min gradient from 5-80% (mobile phase A: 0.1% (v/v) formic acid, 2% (v/v) acetonitrile; mobile phase B: 0.1% (v/v) formic acid, 98% (v/v) acetonitrile) after direct injection onto a nanoLC Column, 3C18-CL, 75 μm*15 cm (Eksigent Technologies). The gradient was comprised of an increase from 2% to 22% mobile phase B over 40 min, 22% to 35% B in 12 min and climbing to 80% B in 4 min, then holding at 80% B for the last 4 min, all at a constant flow rate of 300 nl/min on an Ekisgent NanoLC system. MS1 spectra were collected in the range 350–1,500 m/z for 250 ms. The 50 most intense precursors with charge state 2–5 were selected for fragmentation, and MS2 spectra were collected in the range 100–2,000 m/z for 100 ms; precursor ions were excluded from reselection for 15 s.

The original MS data were submitted to ProteinPilot Software (version 4.5, AB Sciex) for database searching against UniProt Homo Sapiens database (April 9, 2016, containing 160,566 sequences, http://www.uniprot.org/proteomes/UP000005640) concatenated with reverse decoy database. Trypsin/P was specified as a cleavage enzyme allowing up to 3 missing cleavages, 4 modifications per peptide, and 2-5 charges. Mass error was set to 20 ppm for the first search, 5 ppm for the main search, and 0.02 Da for fragmented ions. False discovery rate (FDR) thresholds for protein, peptide, and modification sites were specified at 1%. The minimum peptide length was set at 7. All the other parameters in ProteinPilot were set to default values. In addition, ProteinProspector (v 5.20.0) was used for search comparisons and protein quantitation, as described previously [46, 66].

As a control, other Flag-tagged proteins which are presumably not involved in the MOID pathway, but precipitated with p53 peptides (with Flag-EVI1 [isoform b]), endogenous MAD2 and p53 proteins (with Flag-L1CAM), or AIFM1 peptides (with Flag-DYX1C1) in our analyses (Table S4; data not shown) or previous report [31] are shown (Figures S5A and B). Note that both MAD2 and PML peptides were also precipitated with Flag-L1CAM in our IP-mass spectroscopy analysis (Table S4; data not shown). EVI1 d324 indicates the EVI1 isoform b that lacks the 191-514 a.a. (324 amino acids) and keeps Ser instead of Ala at 191^th^ a.a. (counted from 1^st^ Met) as an additional control. “1st batch” and “2nd batch” indicate that the Flag-immunoprecipitation and the LC-MS/MS were repeated for these Flag-tagged proteins.

### Peptide set enrichment analysis (PSEA)

The peptides from mass-spec results have been mapped to the related genes respectively. The PSEA analysis was performed by GSEA software v4.3.2. For Flag-MAD2 immunoprecipitants (Figures S5A and B), gene sets with the name “Mitochondria” were summarized for any genes with the keyword in their description.

To find AIFM1 among 10 genes identified from both 293 T and HeLa, which were annotated as Apoptosis signaling pathway (P00006) (Fig. 2C and D**)**, all MAD2 interacting proteins in 293T-1^st^ batch and 293T-2^nd^ batch (Table S4) are summarized as 293 T, MAD2 interacting proteins in HeLa-1^st^ batch and HeLa-2nd batch (Table S4) are summarized as HeLa (Fig. 2C). These peptides corresponding genes are further run PANTHER19.0 pathway analysis for each cell line data, respectively. Genes from Apoptosis signaling pathway (P00006) are further analyzed by Venn diagram and 10 genes are sorted out as shown in the table (Fig. 2D).

### Immunofluorescence

Antibodies used in this study are listed in Table S3. The indirect immunofluorescence staining was performed as described previously [20, 21, 27, 46, 47, 49, 50] with the following minor modifications. For the quantitation of anti-LC3B immunofluorescence signals, cells were cultured with NH_4_Cl (20 mM) at 54–72 h after transfection (for 18 h before the fixation). For the immunofluorescence of MAD2 and PML double staining (Fig. 4H) and pattern analysis of MitoTracker signals, PML NB-negative cells, and low DAPI/PML-negative cells, cells were selected with hygromycin (100 μg/ml) (YEASEN, China) 48-96 hr after transfection of siRNA and/or constructs. More than 200 interphase cells in 3 independent experiments were counted. Details about other counting were described in Figure legends and Supplemental Figure legends.

### Image acquisition, processing, quantitation, and colocalization analysis

Cells were observed cells through the Leica TCS SP5II confocal laser scanning microscope (Leica) co-equipped with Leica EL6000 external light source (Leica). Image acquisition was performed by Leica LAS AF software Ver. 4.0 (Leica). Image processing, including deconvolution and signal quantitation, were performed by Image J (NIH).

Alternatively, we observed cells through the GE DeltaVision Elite (DV) microscope system (GE). Image acquisition and image deconvolution were performed by SoftWoRx software Ver. 6.5.2 (GE). Image processing and signal quantitation were performed by Image J (NIH).

The images of TUNEL-positive cells and FLICA-positive cells, including other 2 color images were acquired through a SDPTOP ICX41 fluorescence microscope (Sunny Optical Technology Co.) equipped with a MG-120 LED light source (Mshot). Image acquisition was performed by ImageView software Ver. 3.7.6454 (ToupCam Co.). Image processing, including deconvolution and signal quantitation, were performed by Image J (NIH).

Quantitation and colocalization analysis of immunofluorescence signal intensity in cells was performed manually as described [27, 46, 47] or similar procedures were applied by Image J (NIH) to obtain the Pearson correlation coefficient. The integrated signal intensity in the nucleus was calculated by subtracting the fluorescence intensity of the background (measured outside the nucleus). For quantitation and colocalization analysis of MitoTracker and anti-PML immunofluorescence signals in metaphase cells, we contoured the whole cell area. For quantitation in metaphase cells, we subtracted the fluorescence intensity of the background (measured outside the cell). For quantitation and colocalization analysis of MitoTracker, anti-BECLIN1 (BECN1), anti-LC3, anti-IP3R3, anti-SIGMA1R, and antiphospho-IP3R1 (Ser 1756) immunofluorescence signals in interphase cells (Fig. 6I, K, and M), we contoured the whole cell area. More detailed processes are available upon request.

The population of mitotic cells, abnormal interphase nuclei, and abnormal mitotic cells were examined as previously described [27, 46, 47]. For quantitation of immunofluorescence signals of AIFM1 in nuclear (Fig. 2G and S7N), we used only non-deconvoluted images from the in-focus maximized plane with the most captured fluorescence signals to quantitate the signal diffused into the nucleus.

### RNA-seq and data processing

Transcriptome analysis was performed by using RNA-seq as described previously with the following minor modifications (1). Total RNA was extracted from TSG101-depleted HeLa by siRNA (Control) and TSG101-depleted plus MAD2 overexpressing HeLa (MOID) cells using RNA isolater Total RNA Extraction Reagent (Vazyme) according to the manufacturer’s protocol. RNA quality verification, mRNA enrichment, fragmentation, library cDNA reverse transcription, and the following cDNA library sequencing using Illumina Novaseq6000 are performed by Gene Denovo Biotechnology Co. (Guangzhou, China). Two independent pooled samples per group were analyzed. Paired-end, 150 nt reads were obtained from the same sequence lane. Transcriptome sequencing libraries averaged 40 million paired reads per sample, with 94% alignment to the human genome (GRCh38.p14). The paired-end clean reads were mapped to the reference genome using HISAT2.2.4 and other parameters set as a default. FPKM (fragment per kilobase of transcript per million mapped reads) value was calculated to quantitate its expression abundance and variations, by using the RSEM software. The criteria for a regulated gene were a fold change greater than 1.5 (either direction) and a significant p-value (< 0.05) versus the control group. GO Enrichment Analysis and Reactome Analysis are performed by using online tools from https://www.omicsmart.com/, which is generated by Gene Denovo Biotechnology Co. (Guangzhou, China). GSEA analysis was performed by GSEA software v4.3.2. Gene sets with the name “apoptotic”, “autophagy”, “DNA damage and DNA repair”, “cell-cell adhesion”, “cilium” and “oxidation” were summarized for any genes with these keywords in their description, respectively.

### RNA analyses

Quantitative RT-qPCR was performed as described previously with minor modifications [67]. Total RNA was extracted from transfected cells using RNA isolater (Vazyme, China). Isolated total RNA integrity was verified by ethidium bromide staining and electrophoresis. 1 μg total RNA of each sample were then reverse transcribed with the HiScript III 1st Strand cDNA Synthesis Kit (Vazyme, China) using random hexamer primers according to the manufacturer’s instructions. Real-time quantitative qPCR was performed using the LightCycler® 96 instrument (Roche, Switzerland) with ChamQ SYBR qPCR Master Mix (Vazyme, China). Specific oligonucleotide primers for target gene sequences are listed in Table S7. The expression of β-actin (ACTB) is used to calibrate the targeted mRNA between samples.

### ROS detection

The cell-permeable free radical sensor, DCFH-DA (Heowns, China) was used to measure intracellular ROS levels following the manufacture’s protocol. Briefly, cells were pre-incubated with Hoechst (100 ng/ml) for 30 min at 37 °C in 5% CO_2_, trypsinized, and washed with PBS. 5 × 10^4^ cells or each sample were incubated with DCFH-DA probe (10 μM) in PBS at 37 °C for 45 min. As positive control, cells were co-incubated with H_2_O_2_ (125 nM) during the probe incubation for 45 min. After the probe incubation, cells were washed with PBS twice to remove all non-permeabilized (nonuptaken) probes. After cell uptake, DCFH-DA is deacetylated by cellular esterases to a non-fluorescent compound, which is later oxidized by ROS into 2’- 7’dichlorofluorescein (DCF). Fluorescence signals of DCF (Ex/Em: ∼492–495/517–527 nm) of triplicates or more plate were measured in 96 well by SYNERGY H1 (BioTek).

## Supplementary information

SUPPLEMENTAL MATERIAL

Original western blots in Main Figures

Original western blots in Supplemental Figures
