# Correction: TSG101 depletion dysregulates mitochondria and PML NBs, triggering MAD2-overexpressing interphase cell death (MOID) through AIFM1-PML-DAXX pathway

**DOI:** 10.1038/s41419-024-07294-1

**Published:** 2024-12-16

**Authors:** Yao Xi, Rui Xu, Shengnan Chen, Jiezhu Fang, Xiang Duan, Yidan Zhang, Guoli Zhong, Zhifei He, Yan Guo, Xinyu Li, Wenzhi Tao, Yang Li, Yan Li, Lei Fang, Yohei Niikura

**Affiliations:** 1 National Resource Center for Mutant Mice, MOE Key Laboratory of Model Animals for Disease Study, Model Animal Research Center, Medical School of Nanjing University, Nanjing, 210061 China; 2 Jiangsu Key Laboratory of Molecular Medicine, Medical School of Nanjing University, Nanjing, 210032 China

**Keywords:** Cell division, Cell death

Correction to**:** *Cell Death and Disease* (2024) 15:838 10.1038/s41419-024-07229-w; Article published online 17 November 2024.

In this article a wrong ESM file has been included. This has been corrected.

## Supplementary information

Supplemental text + Figures S1-S10

