# Supplementary material for: TSG101 depletion dysregulates mitochondria and PML NBs, triggering MAD2-overexpressing interphase cell death (MOID) through AIFM1-PML-DAXX pathway
Source: Cell Death Dis. 2024 Nov 17;15(11):838. doi: 10.1038/s41419-024-07229-w (PMC11570632; doi:10.1038/s41419-024-07229-w)
Supplement: Supplementary file 1 — SUPPLEMENTAL MATERIAL [file 41419_2024_7229_MOESM1_ESM.pdf]

Supplemental Information

TSG101 depletion dysregulates mitochondria and PML NBs, triggering MAD2-overexpressing interphase cell death (MOID) through AIFM1-PML-DAXX pathway

Yao Xi,[1,2] Rui Xu,[1,2] Shengnan Chen,[1,2] Jiezhu Fang,[1,2] Xiang Duan,[1,2] Yidan Zhang,[1,2] Guoli Zhong,[1,2] Zhifei He,[1,2] Yan Guo,[2] Xinyu Li,[2] Wenzhi Tao,[1,2] Yang Li,[1,2] Yan Li, [1,2] Lei Fang, [2,#] and Yohei Niikura[1,2,#]

[1] National Resource Center for Mutant Mice, MOE Key Laboratory of Model Animals for Disease Study, Model Animal Research Center, Medical School of Nanjing University, Nanjing 210061, China.

[2] Jiangsu Key Laboratory of Molecular Medicine, Medical School of Nanjing University, Nanjing 210032, China.

[#]Correspondence: njfanglei@nju.edu.cn; niikura@nju.edu.cn

This PDF file includes:

Supplemental Results 1-4

Supplemental References

Tables S1 to S9

Supplemental Figure Legends

Figures S1 to S10

Supplemental Results

Supplemental Result 1. The effect of single depletion of PML, DAXX, MAD2, and TSG101 on cellular events (of mitochondria, autophagy, and PML NBs) and mutual protein stability (related to Figure 6Q, left; Figures S5 and S6). First, we assessed intracellular signals of MitoTracker in interphase cells after the depletion of PML, DAXX, or TSG101 (**Figures S5C-S5E**; **Table S1)**. We note that the majority of PML signals are originated from PML NBs in interphase cells, and mitotic accumulation of PML proteins (MAPP) in metaphase (1). MitoTracker signals were decreased by PML or TSG101 depletion (**Figures S5F** and **S5J-S5L**), suggesting that these proteins are required to maintain mitochondrial integrity, which is consistent with our immunofluorescence analysis (**Figure 2H**). PML signals were decreased by TSG101 depletion specifically in interphase (**Figures S5J** and **S5N-S5P**), while increased by DAXX depletion (**Figure S5H)**. We note that these data are consistent with the elevation of PML level in the western blot after the DAXX depletion (**Figure S6B**). DAXX depletion leads to higher mitochondria (Mito)-LC3B colocalization like mitophagy (**Figure S5G**), while DAXX or TSG101 depletion leads to lower mitochondria (Mito)-PML colocalization (**Figures S5I** and **S5M**) implying unknown dysregulation of inter-organelle signaling. We summarized the close relationship between PML, DAXX, MAD2, and TSG101 in these cellular events, observed through immunofluorescence signaling (**Figure 6Q**, left).

Second, we monitored the protein stability of 3 endogenous proteins (PML, DAXX, and MAD2) after cycloheximide (CHX) treatment in PML, DAXX, or MAD2-depleted cells (**Figures S6A-S6C**). We note that CHX treatment itself still fairly maintained or rather increased PML and DAXX protein levels without any protein

depletion (in RNAi control) with unknown mechanism (**Figures S6A-S6C**). In PML-depleted cells, DAXX protein level was significantly decreased 6 days after the transfection (i.e., 24 h post CHX treatment), and DAXX protein stability (or half-life) was also decreased after the CHX treatment compared with the control (**Figure S6A**). On the other hand, in DAXX-depleted cells, PML protein level was significantly increased 6 days after the transfection (i.e., 24 h post CHX treatment), and elevated PML protein level was also observed after the CHX treatment compared with the control (**Figure S6B**). In MAD2-depleted cells, both PML and DAXX protein levels were significantly decreased 2 days after the transfection (i.e., 24 h post CHX treatment), and these protein stabilities (or half-life) were also decreased after the CHX treatment compared with the control (**Figure S6C**). In addition to the depletion of these 3 proteins, downregulation of DAXX protein was also observed in TSG101-depleted cells (**Figure S5E**). Therefore, these proteins (PML, DAXX, MAD2, and TSG101) maintain a close dependence and contribute mutually to protein stability, although DAXX might have an inhibitory effect on PML. Thus, we summarized the close relationship between PML, DAXX, MAD2, and TSG101 in protein stability (**Figure 6Q**, left).

Supplemental Result 2. The explanation for the weaker effect of the single PML depletion on the reduction of MOID (related to Figures 2J, 6, and S6D)

The double depletion of AIFM1 and PML rescued synthetically the percentage of MOID (**Figure 2J** and **S6D**: ca. 15% MOID), suggesting that AIFM1 is a relatively upstream factor than PML involved in the MOID induction pathway (**Figure 6**, right). Another explanation for the weaker effect of the single PML depletion on the reduction of MOID is, that the PML itself is the constitute of PML nuclear bodies

(NBs), and PML, which constitutes “proper” PML NBs (non-free PML), may rather inhibit the MOID. In this hypothesis, only free (non-SUMOylated) PML released from PML NBs is required to induce the MOID, suggesting a model in which TSG101 prevents PML from being released from PML NBs. In other words, TSG101 is presumably required to maintain the integrity of PML NBs (**Figure 6**, left). In addition, since the TSG101 depletion causes the release of AIFM1 from mitochondria to induce the MOID, TSG101 can be also required to maintain the integrity of mitochondria for cellular viability **(Figure 6**, left). Indeed, the result of our immunofluorescence analysis strongly supports this model: in interphase, TSG101 depletion induces the loss of the signals of MitoTracker and total nuclear PML (**Figures 2H**, **4H**, **4I**, **4K**, and **S5J-N**; Note that the majority of total nuclear signals of PML in interphase derives from PML NBs).

Supplemental Result 3. PML dCC + MAD2 overexpression likely shows MOID characteristics, excluding the possibility of dominant effect on cell death of PML dCC overexpression (related to Figures 4D, 4E, and S7H-S7O).

Our results showed that PML dCC + Flag-Vec overexpression (under the condition that majority of endogenous PML was replaced with PML dCC by PML sgRNA) did not generate TUNEL positive cells, while PML dCC + Flag-MAD2 overexpression did (**Figure S7J**, compare samples [2] and [3] of HeLa or 293T), suggesting that single PML dCC overexpression dose not sufficiently cause dominant effect on cell death. We also selected 5 assays (**Table S8**; ROS detection and immunofluorescence of interphase MAD2, MitoTracker, AIFM, and BECN1) and examined if PML dCC + Flag-Vec overexpression shows MOID characteristics (**Figures S7K-S7O**).

Consistenly, these assays revealed MOID characteristics in PML dCC + Flag-MAD2 overexpression, but not in PML dCC + Flag-Vec overexpression (**Figures S7K-S7O**). Thus, PML dCC + MAD2 overexpression likely shows MOID characteristics, excluding the possibility of dominant effect on cell death of PML dCC overexpression.

Supplemental Result 4. The explanation of the result of SUMOylation assay through HA-PML immunoprecipitation (related to Figure 4F and **S8A).**

We observed the specific interaction between HA-PML and Flag-MAD2 (**Figure 4F** and **S8A**, samples 2 and 4). In addition, the interaction between HA-PML and endogenous MAD2 is increased with TSG101 depletion (**Figure 4F** and **S8A**, samples 3 and 5**),** suggesting that TSG101 can inhibit the interaction between HA-PML and endogenous MAD2, presumably because TSG101 competes with PML to bind MAD2 where MAD2 is not overexpressed (**Figure 6**, left). However, once the Flag-MAD2 is overexpressed regardless of TSG101 depletion, the main interactor of HA-PML switches to overexpressed MAD2 from endogenous MAD2 (**Figure 4F** and **S8A**, samples 4-8). The mechanism and rationale that HA-PML binds Flag-MAD2 more than endogenous MAD2 when Flag-MAD2 is overexpressed are unknown, and we can not avoid the possibility of a slight difference of conformation between exogenous and endogenous MAD2 and stoichiometry of PML-MAD2 interaction. However, we note that the interaction of HA-PML and Flag-MAD2 is significantly decreased in MOID conditions compared to non-MOID conditions (**Figure 4F** and **S8A**, samples 4 and 6-8), suggesting that overexpressed Flag-MAD2 is released from PML NBs during MOID. These results are consistent with the result of SUMOylation assay through Flag-MAD2 immunoprecipitation, suggesting that

overexpressed Flag-MAD2 which contains C-MAD2 is released from PML NB in MOID, concomitant with PML deSUMOylation (**Figure 3G**, samples 2 and 4-6).

Supplemental References

1. Lang A, Lang E, Boe SO. PML Bodies in Mitosis. Cells. 2019;8(8).
2. Giorgi C, Ito K, Lin HK, Santangelo C, Wieckowski MR, Lebiedzinska M, et al. PML regulates apoptosis at endoplasmic reticulum by modulating calcium release. Science. 2010;330(6008):1247-51.
3. Yang M, Ellenberg J, Bonifacino JS, Weissman AM. The transmembrane domain of a carboxyl-terminal anchored protein determines localization to the endoplasmic reticulum. J Biol Chem. 1997;272(3):1970-5.
4. Tarkar A, Loges NT, Slagle CE, Francis R, Dougherty GW, Tamayo JV, et al. DYX1C1 is required for axonemal dynein assembly and ciliary motility. Nat Genet. 2013;45(9):995-1003.
5. Croxton R, Puto LA, de Belle I, Thomas M, Torii S, Hanaii F, et al. Daxx represses expression of a subset of antiapoptotic genes regulated by nuclear factor-kappaB. Cancer Res. 2006;66(18):9026-35.
6. Das TP, Suman S, Papu John AM, Pal D, Edwards A, Alatassi H, et al. Activation of AKT negatively regulates the pro-apoptotic function of death-associated protein kinase 3 (DAPK3) in prostate cancer. Cancer Lett. 2016;377(2):134-9.
7. Boosen M, Vetterkind S, Kubicek J, Scheidtmann KH, Illenberger S, Preuss U. Par-4 is an essential downstream target of DAP-like kinase (Dlk) in Dlk/Par-4-mediated apoptosis. Mol Biol Cell. 2009;20(18):4010-20.
8. Li GM, Li L, Li MQ, Chen X, Su Q, Deng ZJ, et al. DAPK3 inhibits gastric cancer progression via activation of ULK1-dependent autophagy. Cell Death Differ. 2021;28(3):952-67.
9. Shih HM, Chang CC, Kuo HY, Lin DY. Daxx mediates SUMO-dependent transcriptional control and subnuclear compartmentalization. Biochem Soc Trans. 2007;35(Pt 6):1397-400.
10. Park J, Lee JH, La M, Jang MJ, Chae GW, Kim SB, et al. Inhibition of NF-kappaB acetylation and its transcriptional activity by Daxx. J Mol Biol. 2007;368(2):388-97.
11. Santiago A, Godsey AC, Hossain J, Zhao LY, Liao D. Identification of two independent SUMO-interacting motifs in Daxx: evolutionary conservation from Drosophila to humans and their biochemical functions. Cell Cycle. 2009;8(1):76-87.
12. Chen KC, Chang LS. Notexin upregulates Fas and FasL protein expression of human neuroblastoma SK-N-SH cells through p38 MAPK/ATF-2 and JNK/c-Jun pathways. Toxicon. 2010;55(4):754-61.
13. Boellmann F, Guettouche T, Guo Y, Fenna M, Mnayer L, Voellmy R. DAXX interacts with heat shock factor 1 during stress activation and enhances its transcriptional activity. Proc Natl Acad Sci U S A. 2004;101(12):4100-5.
14. Michod D, Bartesaghi S, Khelifi A, Bellodi C, Berliocchi L, Nicotera P, et al. Calcium-dependent dephosphorylation of the histone chaperone DAXX regulates H3.3 loading and transcription upon neuronal activation. Neuron. 2012;74(1):122-35.
15. Puto LA, Reed JC. Daxx represses RelB target promoters via DNA methyltransferase recruitment and DNA hypermethylation. Genes Dev. 2008;22(8):998-1010.
16. Chen HY, Lee YR, Chen RH. The functions and regulations of DAPK in cancer metastasis. Apoptosis. 2014;19(2):364-70.
17. Padmaja Gade DVK. Death-Associated Protein Kinase 1 Suppresses Tumor Growth and Metastasis via Autophagy and Apoptosis. 1: Molecular Mechanisms: Academic Press; 2014. p. 277-92.

18. Lai MC, Liu WY, Liou SS, Liu IM. The protective effects of moscatilin against methylglyoxal-induced neurotoxicity via the regulation of p38/JNK MAPK pathways in PC12 neuron-like cells. Food Chem Toxicol. 2020;140:111369.
19. Choi YH, Bernardi R, Pandolfi PP, Benveniste EN. The promyelocytic leukemia protein functions as a negative regulator of IFN-gamma signaling. Proc Natl Acad Sci U S A. 2006;103(49):18715-20.
20. Wu WS, Vallian S, Seto E, Yang WM, Edmondson D, Roth S, et al. The growth suppressor PML represses transcription by functionally and physically interacting with histone deacetylases. Mol Cell Biol. 2001;21(7):2259-68.
21. Yi TZ, Li J, Han X, Guo J, Qu Q, Guo L, et al. DNMT inhibitors and HDAC inhibitors regulate E-cadherin and Bcl-2 expression in endometrial carcinoma in vitro and in vivo. Chemotherapy. 2012;58(1):19-29.
22. Liu D, Liu M, Wang W, Pang L, Wang Z, Yuan C, et al. Overexpression of apoptosis-inducing factor mitochondrion-associated 1 (AIFM1) induces apoptosis by promoting the transcription of caspase3 and DRAM in hepatoma cells. Biochem Biophys Res Commun. 2018;498(3):453-7.
23. Wang Y, Zhang G, Meng Q, Huang S, Guo P, Leng Q, et al. Precise tumor immune rewiring via synthetic CRISPRa circuits gated by concurrent gain/loss of transcription factors. Nat Commun. 2022;13(1):1454.

Table S1. siRNAs, CRISPRi (sgRNA), and CRISPR KO vectors used in this study

Type	Target protein	Indication in present study	Relevant characteristic(s)	Database no.	Target sequence (Forward sequence)	Source / Reference	Catalog / ID number
siRNA	Luciferase	Luc	1 target	Si68	CUUACGCUGAGUACUUCGAdTdT	Genescript	N.A.
siRNA	N.A.	Control or Control siRNA	1 target	Si20	UUCUCCGAACGUGUCACGUdTdT	GenePharma	N.A.
siRNA	N.A.	Control or Control siRNA	1 target	Si78	UUCUCCGAACGUGUCACGUdTdT	Sangon	N.A.
siRNA	TSG101	#S1 (in Figure S1)	1 target	Si79	CCUCCAGUCUUCUCUCGUCdTdT	Sangon	N.A.
siRNA	TSG101	#1	siRNA pool (2 targets mixture)	Si79	CCUCCAGUCUUCUCUCGUCdTdT	Sangon	N.A.
				S99	GUUCUCAGUACCCUUCUCAdTdT	Sangon	N.A.
siRNA	TSG101	#2 or TSG101 siRNA (except in Figure S1)	siRNA pool (3 targets mixture), UTR targets	Si80	UGCACUUUCUAUCCUCUGUdTdT	Sangon	N.A.
				Si100	GAGGUUGAGCUCUUCUUAAdTdT	Sangon	N.A.
				Si101	GCCCAGCAGCGGCUGACCCdTdT	Sangon	N.A.
siRNA	p53	#1	siRNA pool (3 targets mixture)	Si26	GAGGGATGTTTGGGAGATGTA	GenePharma	N.A.
				Si27	CCCUUGCUUGCAAUAGGUGdTdT	GenePharma	N.A.
				Si28	GUUGGCCUGCACUGGUGUUdTdT	GenePharma	N.A.
siRNA	p53	#2	siRNA pool (2 targets mixture)	Si29	CGGCGCACAGAGGAAGAGAAU	GenePharma	N.A.
				Si30	UCAGACCUAUGGAAACUACUU	GenePharma	N.A.
siRNA	AIFM1	#1	1 target	Si53	CUUGUUCCAGCGAUGGCAU	GenePharma	N.A.
		#2	1 target	Si54	CUUGUUCCAGCGAUGGCAUdTdT	GenePharma	N.A.
siRNA	CHMP7/hSNF7	#1	siRNA pool (4 targets mixture)	Si110	CAAGGUAGAUGAACUGAUG	Thermo/Dharmacon	N.A.
				Si111	UAAGAGAGCUGCCCUACAG	Thermo/Dharmacon	N.A.
				Si112	GGAAUUGGCCCAGGAGUUG	Thermo/Dharmacon	N.A.
				Si113	GAAGCAAUACAGAAACUGA	Thermo/Dharmacon	N.A.
siRNA	ALIX (PDCD6IP)	#1	1 target	Si114	GCCGCUGGUGAAGUUCAUCdTdT	This study (1)	N.A.
		#2	1 target	Si115	GAAGGAUGCUUUCGAUAAAdTdT	This study (1)	N.A.
		#3	siRNA pool (4 targets mixture)	Si126	CCAGAACAAAUGCAGUGAUAUdTdT	Sangon	N.A.
				Si129	GACUGAAUGCAGUGUAUAAUU	Sangon	N.A.

				Si131	GAUGCUUUCGAUAAAGGUUCA	Sangon	N.A.
				Si132	CAUUUGAAACGAUUAUACUUU	Sangon	N.A.
siRNA	PTPN23	#1	1 target	Si116	GUGCACAGGUGGUAGAUUAdTdT	This study (1)	N.A.
		#2	1 target	Si117	GCAAACAGCGGAUGAGCAAdTdT	This study (1)	N.A.
siRNA	CENP-F	#1	1 target	Si118	GAAUCUUAGUAGUCAAGUAdTdT	This study (1)	N.A.
		#2	1 target	Si119	GGUUAUUGUCUGCCUUGAAdTdT	This study (1)	N.A.
siRNA	RNF20	#1	siRNA pool (4 targets mixture)	Si120	CCAAUGAAAUCAAGUCUAA	Thermo/Dharmacon	N.A.
				Si121	UAAGGAAACUCCAGAAUAU	Thermo/Dharmacon	N.A.
				Si122	GCAAAUGUCCCAAGUGUAA	Thermo/Dharmacon	N.A.
				Si123	AGAAGAAGCUACAUGAUUU	Thermo/Dharmacon	N.A.
siRNA	MED31	#1	siRNA pool (4 targets mixture)	Si124	GAAUAUGCCAAGUAUCUAA	Thermo/Dharmacon	N.A.
				Si125	GUUUAGCCAACCCAAAUUA	Thermo/Dharmacon	N.A.
				Si126	UAACACAUCGGGAAAAUGA	Thermo/Dharmacon	N.A.
				Si127	GCGGAUGCGCCUUCAGCAA	Thermo/Dharmacon	N.A.
siRNA	VPS25	#1	siRNA pool (4 targets mixture)	Si128	CAGAACAACUCCGUCUUUA	Thermo/Dharmacon	N.A.
				Si129	GCACAAGGCCGAGAUCAUC	Thermo/Dharmacon	N.A.
				Si130	GGGAAACUCAUCUAUCAGU	Thermo/Dharmacon	N.A.
				Si131	GUCGAUCCAGAUUGUAUUA	Thermo/Dharmacon	N.A.
siRNA	RAB7	#1	siRNA pool (4 targets mixture)	Si132	CUAGAUAGCUGGAGAGAUG	Thermo/Dharmacon	N.A.
				Si133	GUACAAAGCCACAAUAGGA	Thermo/Dharmacon	N.A.
				Si134	AAACGGAGGUGGAGCUGUA	Thermo/Dharmacon	N.A.
				Si135	CGAAUUUCCUGAACCUAUC	Thermo/Dharmacon	N.A.
siRNA	WDR5	#1	siRNA pool (4 targets mixture)	Si136	GAGAGUGGCUGGCAAGUUC	Thermo/Dharmacon	N.A.

				Si137	GACGAAAGCGUGAGGAUAU	Thermo/Dharmacon	N.A.
				Si138	CAGAGGAUAACCUUGUUUA	Thermo/Dharmacon	N.A.
				Si139	GCACAUGACUCUCCCGUUU	Thermo/Dharmacon	N.A.
siRNA	VBP1	#1	siRNA pool (4 targets mixture)	Si140	GGAGGAAGACCUUGACUUU	Thermo/Dharmacon	N.A.
				Si141	GAACAGUACCAGAAGUAUA	Thermo/Dharmacon	N.A.
				Si142	GAACUCAACCUUGCUCAAA	Thermo/Dharmacon	N.A.
				Si143	GGGCUAAUGUAAUGCUUGA	Thermo/Dharmacon	N.A.
siRNA	SUPT4H1	#1	siRNA pool (4 targets mixture)	Si144	ACCGAGAGAUGGUAUAUGA	Thermo/Dharmacon	N.A.
				Si145	CAGCGAGUCAGUAACUUUA	Thermo/Dharmacon	N.A.
				Si146	UCAAGACUAUAGACCAGUU	Thermo/Dharmacon	N.A.
				Si147	UGACAAUUGUGAUGCAUAU	Thermo/Dharmacon	N.A.
siRNA	SNF8	#1	1 target	Si127	GUAAGCAUAAUCUGAUUAACA	Sangon	N.A.
		#2	siRNA pool (2 targets mixture)	Si124	CUGAUAACUUUGGAGGAACUAdTdT	Sangon	N.A.
				Si130	CGAACUAGGUGUCCAAAUUAU	Sangon	N.A.
siRNA	CHMP2A	#1	1 target	Si125	CAUGGAUAUGAAGGAGGAGAUdTdT	Sangon	N.A.
		#2	1 target	Si128	CCAUCAUGGACCUAUUGUUCG	Sangon	N.A.
CRISPRi (sgRNA) vector	N.A.	Ctrl or iVec	p62988-sgRNA-dCas9-KRAB(ZNF10K)-2	D248	N.A.	This study	N.A.
CRISPRi (sgRNA) vector	PML	#1	CRISPRi-sgRNA-PML-T1 (same background as D248)	Cv20	caccgccgagaatcgaaactaagct	This study	N.A.
CRISPR KO vector	N.A.	Ctrl or koVec	LentiCRISPRi-v2-dCas9	D374	N.A.	This study	N.A.

CRISPR KO vector	DAXX	#1	LentiCRISPR-sgRNA-DAXX-T1-G (same background as D374)	Cv73	gaatgttgcaagacaaaagtg	This study	N.A.
CRISPR KO vector	DAXX	#2	LentiCRISPR-sgRNA-DAXX-T2 (same background as D374)	Cv74	gatctggaacagatgcagga	This study	N.A.

(1) Synthesized by the Hartwell Center for Bioinformatics and Biotechnology, St. Jude Children's Research Hospital.

Table S2. Plasmid vector constructs used in this study

D number	Relevant characteristic(s)	Used for	Source / Reference / Note
D208	pQCXIP-Flag	IP-mass spectrometry and/or other assays.	This study.
D196	pQCXIP-Flag-human EVI1 isoform b	IP-mass spectrometry and/or other assays.	This study.
D185	pQCXIP-Flag-human EVI1 isoform b d324	IP-mass spectrometry and/or other assays.	This study. This is EVI1 isoform b that lacks the 190-513 a.a. (324 amino acids) and keeps Ser instead of Ala at 190th a.a..
D177	pcDNA5-Flag (Vector control; 1st clone)	IP-mass spectrometry and/or other assays.	This study.
D347	pcDNA5-Flag (Vector control; 2nd clone)	IP-mass spectrometry, TUNEL, IP, Western blot, and/or other assays.	This study.
D348	pcDNA5-Flag-human MAD2	IP-mass spectrometry, TUNEL, IP, Western blot, and/or other assays.	This study.
D416	pcDNA5-Flag-MAD2 R133A (O, C)	TUNEL, Western blot, and/or other assays.	This study.
D417	pcDNA5-Flag-MAD2 R133A/L13A (C)	IP, TUNEL, Western blot, and/or other assays.	This study.
D418	pcDNA5-Flag-MAD2 R133A/W75A (C)	IP, TUNEL, Western blot, and/or other assays.	This study.
D419	pcDNA5-Flag-MAD2 R133A/W167A (O, C)	TUNEL, Western blot, and/or other assays.	This study.
D420	pcDNA5-Flag-MAD2 R133A/F186A (O)	IP, TUNEL, Western blot, and/or other assays.	This study.
D421	pcDNA5-Flag-MAD2 L13Q (C)	TUNEL, Western blot, and/or other assays.	This study.
D422	pcDNA5-Flag-MAD2 F141A (O)	IP, TUNEL, Western blot, and/or other assays.	This study.
D425	pcDNA5-Flag-MAD2 R133E/Q134A (O, C)	TUNEL, Western blot, and/or other assays.	This study.
D426	pcDNA5-Flag-MAD2 R133A/F141A (O, C)	TUNEL, Western blot, and/or other assays.	This study.
D351	pcDNA5-Flag-human TSG101	TUNEL, IP-mass spectrometry, and/or other assays.	This study.
D495	pcDNA5-Flag-human TSG101 Y390F	TUNEL, Immunofluorescence, and/or other assays.	This study.

D375	pcDNA5-untagged (Vector control)	TUNEL, IP, and/or other assays.	This study.
D381	pcDNA5-untagged-human TSG101 WT (1st clone)	TUNEL, IP, and/or other assays.	This study.
D450	pcDNA5-untagged-human TSG101 WT (2nd cone)	Western blot	This study.
D440	pcDNA5-untagged-human TSG101 T220A	TUNEL, IP, and/or other assays.	This study.
D441	pcDNA5-untagged-human TSG101 S309A	TUNEL, IP, and/or other assays.	This study.
D442	pcDNA5-untagged-human TSG101 Y390F	TUNEL, IP, and/or other assays.	This study.
D408	pQCXIP-Flag (Vector control)	Immunofluorescence	This study.
D414	pQCXIP-Flag-TSG101 WT	Immunofluorescence	This study.
D434	pQCXIP-Flag-TSG101 Y390F	Immunofluorescence	This study.
D162	pcDNA5-HA (Vector control)	TUNEL, IP, and/or other assays.	This study.
D496	pcDNA5-HA-human TSG101 WT	IP, and/or other assays.	This study.
D497	pcDNA5-HA-human TSG101 Y390F	IP, and/or other assays.	This study.
D431	pcDNA5-HA-human PML WT (wtPML)	TUNEL, IP, and/or other assays.	This study.
D445	pcDNA5-HA-human PML dCC	TUNEL, IP, and/or other assays.	This study.
D446	pcDNA5-HA-human PML K160R	TUNEL, IP, and/or other assays.	This study.
D447	pcDNA5-HA-human PML MIM (1st clone)	Western blot	This study.
D452	pcDNA5-HA-human PML MIM (2nd clone)	Western blot	This study.
D486	pcDNA5-HA-human erPML (PML-MVYIGIAIFLFLVGLFMK)	TUNEL, IP, and/or other assays.	This study and Giorgi at al. (2).
D487	pcDNA5-HA-human gbPML (PML-MVYIGIAIFLFLVGLFMVAVAK)	TUNEL, IP, and/or other assays.	This study and Yang at al. (3).
D187	pcDNA5-Flag-human DAXX	IP-mass spectrometry and/or other assays.	This study.
D263	pLKO.1-CMV-3xFlag	IP-mass spectrometry, IP, and/or other assays.	This study.
D267	pLKO.1-CMV-3xFlag-PML	IP-mass spectrometry, IP, and/or other assays.	This study.
D269	pLKO.1-CMV-3xFlag-PML dCC	IP-mass spectrometry, IP, and/or other assays.	This study.
D391	pLKO.1-CMV-3xFlag-PML K160R	IP, and/or other assays.	This study.
D292	pLKO.1-cmv-3xFlag-human L1CAM	IP-mass spectrometry and/or other assays.	This study.
D275	pLKO.1-cmv-3xFlag-human DYX1C1	IP-mass spectrometry and/or other assays.	This study.

D248	p62988-sgRNA-dCas9-KRAB(ZNF10K)-2	CRISPRi (sgRNA) vector (iVec)	This study. Other CRISPRi (sgRNA) vectors are show in Table S2.
D374	p62988-sgRNA-dCas9-KRAB(ZNF10K)-2	CRISPR KO vector	This study. CRISPR KO vectors are show in Table S2.

Table S3. Antibodies used in this study

Database number	Antibody	Antibody type	Source	Catalog/Database number
A19	Anti-MAD2	Rabbit polyclonal	ABclonal Technology	A1699
A122	Anti-MAD2	Rabbit monoclonal	ABclonal Technology	A11469
A146	Anti-MAD2	Rabbit polyclonal	Biogot technology	BS5787
A207	Anti-MAD2	Mouse monoclonal	Santa Cruz Biotechnology	sc-374131
A75	Anti-PML	Mouse monoclonal	Santa Cruz Biotechnology	sc-966
A29	Anti-PML	Rabbit polyclonal	ABclonal Technology	A1184
A31	Anti-DAXX	Rabbit polyclonal	Proteintech	20489-1-AP
A132	Anti-TSG101/VPS23	Rabbit monoclonal	ABclonal Technology	A5789
A39	Anti-p53	Rabbit polyclonal	Santa Cruz Biotechnology	SC-126
A72	Anti-AIFM1	Rabbit monoclonal	ABclonal Technology	A19536
A228	Anti-BECN1	Rabbit polyclonal	Proteintech	11306-1-AP
A147	Anti-LC3B	Rabbit monoclonal	ABclonal Technology	A19665
A192	Anti-IP3R3 (ITPR3)	Rabbit polyclonal	ABclonal Technology	A12794
A193	Anti-SIGMA1R	Rabbit polyclonal	ABclonal Technology	A14837
A229	Anti-phospho-IP3R1 (Ser 1756)	Rabbit polyclonal	ABclonal Technology	AP1224
A6	Anti-GAPDH	Mouse monoclonal	ABclonal Technology	AC033
A2	β-actin	Rabbit monoclonal	ABclonal Technology	AC026
A65	Anti-SUMO2	Rabbit polyclonal	ABclonal Technology	A2571
A7	Anti-Flag	Mouse monoclonal	ABclonal Technology	AE005
A8	Anti-Flag	Rabbit polyclonal	ABclonal Technology	AE004
A91	Anti-Flag	Mouse monoclonal	Abbkine	ABB-A02010
A128	Anti-Flag	Rat monoclonal	Invitrogen	MA1-142
A151	Anti-Flag (DYKDDDDK) Affinity Beads	Mouse monoclonal	Smart-Lifesciences	SA042005
A37	Anti-HA	Mouse monoclonal	ABclonal Technology	AE008
A38	Anti-HA	Rabbit monoclonal	Cell Signaling Technology	3724T

A171	Anti-HA	Rabbit monoclonal	Biogot technology	AP0005M
A194	Anti-HA Affinity Beads	Mouse monoclonal	Cell Signaling Technology	SA068005
A235	Anti-HA	Rabbit monoclonal	Proteintech	51064-2-AP
A35	Rabbit IgG control	Non-specific control	R&D Systems	AB-105-C
ANTI #65	Anti-phospho-Histone H3 (Ser 10), Mitosis marker	Mouse monoclonal	abcam	7031100
ANTI #82	Anti-phospho-Histone H3 (Ser 10), Mitosis marker	Rabbit polyclonal	Upstate	6570
SA7	IRDye® 800CW Goat anti-Mouse IgG (H + L), 0.1 mg	Affinity-purified secondary antibody	LI-COR Biosciences	P/N 925-32210
SA8	IRDye® 800CW Goat anti-Rabbit IgG (H + L), 0.1 mg	Affinity-purified secondary antibody	LI-COR Biosciences	P/N 925-32211
SA10	IRDye® 680RD Goat anti-Mouse IgG (H + L), 0.1 mg	Affinity-purified secondary antibody	LI-COR Biosciences	P/N 925-68070
SA11	IRDye® 680RD Goat anti-Rabbit IgG (H + L), 0.1 mg	Affinity-purified secondary antibody	LI-COR Biosciences	P/N 925-68071
SA13	Peroxidase AffiniPure Goat Anti-Mouse IgG (H+L)	Affinity-purified secondary antibody	Jackson ImmunoResearch	115-035-003
SA14	Peroxidase AffiniPure Goat Anti-Rabbit IgG (H+L)	Affinity-purified secondary antibody	Jackson ImmunoResearch	111-035-003
SA22	HRP Goat Anti-Rabbit IgG (H+L)	Affinity-purified secondary antibody	ABclonal Technology	AS014
SA1	Affinity-purified secondary antibody Alexa Fluor® 488 Goat Anti-Mouse IgG (H+L)	Affinity-purified secondary antibody	Jackson ImmunoResearch	115-545-003

SA2	Affinity-purified secondary antibody Alexa Fluor® 488 Goat Anti-Rabbit IgG (H+L)	Affinity-purified secondary antibody	Jackson ImmunoResearch	111-545-003
SA3	Affinity-purified secondary antibody Cy™3 Goat Anti-Mouse IgG (H+L)	Affinity-purified secondary antibody	Jackson ImmunoResearch	115-165-003
SA4	Affinity-purified secondary antibody Cy™3 Goat Anti-Rabbit IgG (H+L)	Affinity-purified secondary antibody	Jackson ImmunoResearch	111-165-003

Table S4. Number of peptides interacted with Flag-proteins in IP-mass spectrometry

Cell line	Immunoprecipitaed Flag-protein	PML peptide	MAD2 peptide	TSG101 peptide	DAXX peptide	EVI1 peptide	L1CAM peptide	DYX1C1 peptide	AIFM1 peptide	p53 peptide	p53BP1 peptide	SUGT1 peptide	BUB3 peptide	BugZ (ZNF207) peptide	CENP-T peptide	CENP-V peptide
293T	PML	76 (1)	3 (1)						5 (0)	2 (1)		3 (1)	5 (3)			2 (0)
	MAD2, 1st batch		264 (66)						15 (13)	3 (0)		6 (1)	9 (7)	0 (2)		1 (0)
	MAD2, 2nd batch		295 (12)						20 (13)	1 (0)			7 (15)	0 (2)		1 (0)
	TSG101			46 (1)					1 (5)		0 (1)	0 (2)	0 (1)	0 (1)		
	DAXX				826 (4)				3 (0)				0 (2)	1 (0)		
	EVI1		1 (3)			72 (0)			4 (8)	2 (1)		6 (6)	12 (8)			
	EVI1 d324		1 (3)			26 (0)			3 (8)	1 (0)		3 (6)	9 (8)			1 (0)
	L1CAM						8 (0)						3 (3)			
	DYX1C1							210 (0)	(4)				7 (3)		1 (0)	
HeLa	PML, 1st batch	46 (0)					5 (1)		12 (5)			4 (1)	6 (10)	1 (0)		
	PML, 2nd batch	30 (2)							3 (4)			2 (1)	5 (4)	0 (1)		
	PML dCC, 1st batch	89 (0)					4 (1)		16 (5)			7 (1)	8 (10)			1 (0)
	PML dCC, 2nd batch	30 (2)							1 (4)				5 (4)	0 (1)		1 (0)
	MAD2, 1st batch		3 (0)						20 (13)		1 (0)		2 (6)	1 (1)		
	MAD2, 2nd batch		88 (15)				3 (0)		15 (16)			5 (6)	10 (18)	2 (0)		2 (0)
	TSG101		3 (17)	38 (0)			0 (1)		4 (6)				3 (1)	1 (1)		1 (1)
	DAXX				66 (0)											

Note: Yellow indicates the cases where no Vector-interacting peptide background was observed or where the peptides' number in Flag-proteins' precipitants were higher more than 2-fold in the Vectors' precipitants. Green indicates the peptides of the immunoprecipitated Flag-proteins. Grey indicates the cases where peptides' number in the Vectors' precipitants (shown in the parenthesis) were higher than in Flag-proteins' precipitants.

Table S5. Simplified sample indications used in this study

Figure number, etc.	Indication in fugure	Transfected vector(s) and siRNA(s)	Notes
Figures 1H-1N and S3	[1] Control	Flag-Vec (D347) + Control siRNA (si20 or Si78)	Conceptually identical to Figures 4H-4K, [1].
	[2] MAD2 O/E	Flag-MAD2 (D348) + Control siRNA (si20 or Si78)	Conceptually identical to Figures 4H-4K, [2].
	[3] TSG101 KD	Flag-Vec (D347) + TSG101 siRNA #2 (Si80/100/101)	Conceptually identical to Figures 4H-4K, [3].
	[4] MOID	Flag-MAD2 (D348) + TSG101 siRNA #2 (Si80/100/101)	Conceptually identical to Figures 4H-4K, [4].
	[5] TSG101 O/E Rescue	Flag-MAD2 (D348) + TSG101 siRNA #2 (Si80/100/101) + Untagged-TSG101 WT (D381)	Conceptually identical to Figures 4H-4K, [5].
	[6] AIFM1 KD Rescue	Flag-MAD2 (D348) + TSG101 siRNA #2 (Si80/100/101) + AIFM1 siRNA (#1 + #2)	
Figures 4D, 4E, S7H, and S7I	[i]	HA-Vec (D162) + iVec sgRNA (D248) + Control siRNA (si20 or Si78) + Flag-Vec (D347)	Conceptually identical to Figures 6O and 6P, [i].
	[ii]	HA-Vec (D162) + iVec sgRNA (D248) + TSG101 siRNA #2 (Si80/100/101) + Flag-Vec (D347)	
	[iii]	HA-Vec (D162) + PML sgRNA #1 (Cv20) + Control siRNA (si20 or Si78) + Flag-Vec (D347)	
	[iv]	HA-Vec (D162) + iVec sgRNA (D248) + Control siRNA (si20 or Si78) + Flag-MAD2 (D348)	
	[v]	HA-Vec (D162) + iVec sgRNA (D248) + TSG101 siRNA #2 (Si80/100/101) + Flag-MAD2 (D348)	Conceptually identical to Figures 6O and 6P, [ii].
	[vi]	HA-Vec (D162) + PML sgRNA #1 (Cv20) + TSG101 siRNA #2 (Si80/100/101) + Flag-MAD2 (D348)	Conceptually identical to Figures 6O and 6P, [iii].
	[vii]	HA-PML WT (D431) + PML sgRNA #1 (Cv20) + TSG101 siRNA #2 (Si80/100/101) + Flag-MAD2 (D348)	Conceptually identical to Figures 6O and 6P, [iv].
	[viii]	HA-PML dCC (D445) + PML sgRNA #1 (Cv20) + TSG101 siRNA #2 (Si80/100/101) + Flag-MAD2 (D348)	
	[ix]	HA-PML K160R (D446) + PML sgRNA #1 (Cv20) + TSG101	

		siRNA #2 (Si80/100/101) + Flag-MAD2 (D348)	
	[x]	HA-Vec (D162) + PML sgRNA #1 (Cv20) + Control siRNA (si20 or Si78) + Flag-MAD2 (D348)	Conceptually identical to Figures 6O and 6P, [vii].
	[xi]	HA-PML WT (D431) + PML sgRNA #1 (Cv20) + Control siRNA (si20 or Si78) + Flag-MAD2 (D348)	Conceptually identical to Figures 6O and 6P, [viii].
	[xii]	HA-PML dCC (D445) + PML sgRNA #1 (Cv20) + Control siRNA (si20 or Si78) + Flag-MAD2 (D348)	
	[xiii]	HA-PML K160R (D446) + PML sgRNA #1 (Cv20) + Control siRNA (si20 or Si78) + Flag-MAD2 (D348)	
Figures 4H-4K, S1I, S1J, and S9	[1]	Flag-Vec (D347) + Control siRNA (si20 or Si78)	Conceptually identical to Figures 1H-1J and S3, Control.
	[2]	Flag-MAD2 (D348) + Control siRNA (si20 or Si78)	Conceptually identical to Figures 1H-1J and S3, MAD2 O/E.
	[3]	Flag-Vec (D347) + TSG101 siRNA #2 (Si80/100/101)	Conceptually identical to Figures 1H-1J and S3, TSG101 KD.
	[4]	Flag-MAD2 (D348) + TSG101 siRNA #2 (Si80/100/101)	Conceptually identical to Figures 1H-1J and S3, MOID.
	[5]	Flag-MAD2 (D348) + TSG101 siRNA #2 (Si80/100/101) + Untagged-TSG101 WT (D381)	Conceptually identical to Figures 1H-1J and S3, TSG101 O/E Rescue.
	[6]	Flag-MAD2 (D348) + TSG101 siRNA #2 (Si80/100/101) + Untagged-TSG101 Y390F (D442)	
Figures 6O and 6P	[i]	HA-Vec (D162) + iVec sgRNA (D248) + Control siRNA (Si78) + Flag-Vec (D347)	Conceptually identical to Figures 4D, 4E, S7N, and S7O, [i].
	[ii]	HA-Vec (D162) + iVec sgRNA (D248) + TSG101 siRNA #2 (Si80/100/101) + Flag-MAD2 (D348)	Conceptually identical to Figures 4D, 4E, S7N, and S7O, [v].
	[iii]	HA-Vec (D162) + PML sgRNA #1 (Cv20) + TSG101 siRNA #2 (Si80/100/101) + Flag-MAD2 (D348)	Conceptually identical to Figures 4D, 4E, S7N, and S7O, [vi].

	[iv]	HA-wtPML (D431) + PML sgRNA #1 (Cv20) + TSG101 siRNA #2 (Si80/100/101) + Flag-MAD2 (D348)	Conceptually identical to Figures 4D, 4E, S7N, and S7O, [vii].
	[v]	HA-erPML (D486) + PML sgRNA #1 (Cv20) + TSG101 siRNA #2 (Si80/100/101) + Flag-MAD2 (D348)	
	[vi]	HA-gbPML (D487) + PML sgRNA #1 (Cv20) + TSG101 siRNA #2 (Si80/100/101) + Flag-MAD2 (D348)	
	[vii]	HA-Vec (D162) + PML sgRNA #1 (Cv20) + Control siRNA (Si78) + Flag-MAD2 (D348)	Conceptually identical to Figures 4D, 4E, S7N, and S7O, [x].
	[viii]	HA-wtPML (D431) + PML sgRNA #1 (Cv20) + Control siRNA (Si78) + Flag-MAD2 (D348)	Conceptually identical to Figures 4D, 4E, S7N, and S7O, [xi].
	[ix]	HA-erPML (D486) + PML sgRNA #1 (Cv20) + Control siRNA (Si78) + Flag-MAD2 (D348)	
	[x]	HA-gbPML (D487) + PML sgRNA #1 (Cv20) + Control siRNA (Si78) + Flag-MAD2 (D348)	

Note 1: See Table S1, 2 for details of plasmid vectors and siRNAs.
Note 2: To adjust the total amount of plasmid vectors and siRNAs identical among different samples, we added Control siRNA (si20 or Si78) and Untagged-Vec (D375) upon the necessity.
Note 3: For Figures 1H-1K and S3, sample numbers were omitted in the figures for simplicity.

Table S6. Reported target genes transcriptionally regulated by DAXX/PML/AIFM1/HDACs

Gene name commonly used [species used in the report]	Gene name (NCBI Official Symbol)	RNA-seq result (Up or Down)	Apoptotic of anti-apoptotic (or others)?	Transcriptional activator/repressor	Transcriptional co-activator/co-repressor	Reference
cIAP2 [human]	BIRC3	Not significantly changed	Anti-apoptotic	RelB (activator)	DAXX (co-repressor)	(5)
cFLIP [human]	CFLAR	Not significantly changed	Anti-apoptotic	RelB (activator)	DAXX (co-repressor)	(5)
Survivin [human]	BIRC5	Not significantly changed	Anti-apoptotic	RelB (activator)	DAXX (co-repressor)	(5)
ZipK [human]	DAPK3	Not significantly changed	Dual function	RelB (activator)	DAXX (co-repressor, if not major repressor)	(For anti-apoptotic function) (5); (For apoptotic function) (6, 7); (For autophagic cell death function) (8)
MAIL [human]	NFKBIZ	Not significantly changed	Apoptotic	Unknown	DAXX (co-activator, if not major activator)	(5)
Bok [human]	BOK	Not significantly changed	Apoptotic	Unknown	DAXX (co-activator, if not major activator)	(5)
PAI-1 [human]	SERPINE1	Not significantly changed	Apoptotic	Smad4 (activator)	DAXX (co-activator)	(9)
IkB-alpha [human]	NFKBIA	Not significantly changed	Contex/condition-dependent (generallly pro-apoptotic) or unknown	NF-kB (repressor)	DAXX (co-repressor)	(10)
IL8 [human]	CXCL8	Not significantly changed	Contex/condition-dependent or unknown	NF-kB (repressor)	DAXX (co-repressor)	(10)
Bcl-2 [human]	BCL2	Not significantly changed	Anti-poptotic	ETS-1 (activator)	DAXX (co-repressor)	(10)
Fas ligand (FASLG/CD95/Apo-1) [human]; Unclear if this is target gene of DAXX.	FASLG	Expression was not detetced	Apoptotic	c-Jun (activator)	DAXX (co-activator)	(11, 12)
HSP70 [human]	HSPA4	Not significantly changed	Contex/condition-dependent or unknown	HSF1 (activator)	DAXX (co-activator)	(13)
Bdnf [mouse]	BDNF	Not significantly changed	Contex/condition-dependent (immediate early genes [IEG] of neuron) or unknown	DAXX (if not co-activator)	DAXX (if not main activator)	(14)
c-Fos [mouse]	FOS	Not significantly changed	Contex/condition-dependent (immediate early genes [IEG] of neuron) or unknown	DAXX (if not co-activator)	DAXX (if not main activator)	(14)
dapk1 (mouse)	DAPK1	No change, redundant with below	Dual function	RelB (activator)	DAXX, DNMT1/3 (co-repressor)	(15); (For apoptotic function) (16, 17); (For anti-necroptotic fucntion) (18)
dapk3 (mouse)	DAPK3	No change, redundant with above	Dual function	RelB (activator)	DAXX, DNMT1/3 (co-repressor)	(For anti-apoptotic function) (5); (For apoptotic function) (6, 7); (For autophagic cell death function) (8)
c-flip (mouse)	CFLAR	Up (but p > 0.05), redundant with above	Anti-apoptotic	RelB (activator)	DAXX, DNMT1/3 (co-repressor)	(15)

birc3 (ciap2) (mouse)	BIRC3	No change, redundant with above	Anti-apoptotic	RelB (activator)	DAXX, DNMT1/3 (co-repressor)	(15)
Ifi202B [mouse]	PYHIN1	Expression was not detetced	Contex/condition-dependent or unknown	STAT-1alpha (activator)	PML (inhibitor of STAT-1alpha to bind DNA)	(19)
Ifi203 [mouse]	No human homolog	No human homolog	Contex/condition-dependent or unknown	STAT-1alpha (activator)	PML (inhibitor of STAT-1alpha to bind DNA)	(19)
Ifi204 [mouse]	IFI16	Up	Contex/condition-dependent or unknown	STAT-1alpha (activator)	PML (inhibitor of STAT-1alpha to bind DNA)	(19)
Ifi205 [mouse]	No human homolog	No human homolog	Contex/condition-dependent or unknown	STAT-1alpha (activator)	PML (inhibitor of STAT-1alpha to bind DNA)	(19)
Ifi10 (IP-10) [mouse]	CXCL10	Expression was not detetced	Contex/condition-dependent or unknown	STAT-1alpha (activator)	PML (inhibitor of STAT-1alpha to bind DNA)	(19)
Clcn3 [mouse]	CLCN3	Not significantly changed	Contex/condition-dependent or unknown	STAT-1alpha (activator)	PML (inhibitor of STAT-1alpha to bind DNA)	(19)
Rgs2 [mouse]	RGS2	Expression was not detetced	Contex/condition-dependent or unknown	STAT-1alpha (activator)	PML (inhibitor of STAT-1alpha to bind DNA)	(19)
Gnbp3 [mouse]	MUL1	Not significantly changed	Contex/condition-dependent or unknown	STAT-1alpha (activator)	PML (inhibitor of STAT-1alpha to bind DNA)	(19)
TNFaip2 [mouse]	TNFAIP2	Not significantly changed	Contex/condition-dependent or unknown	STAT-1alpha (activator)	PML (inhibitor of STAT-1alpha to bind DNA)	(19)
Bcl-2 [human] ; Unclear if this is target gene of PML.	BCL2	Not significantly changed	Anti-poptotic	HDAC (repressor)	PML (co-repressor)	(20, 21)
Caspase 3 [human]; Limited in reporter gene assay.	CASP3	Not significantly changed	Apoptotic	AIFM1 (if not co-activator)	AIFM1 (if not main activator)	(22)
DRAM [human]; Limited in reporter gene assay.	DRAM1	Not significantly changed	Autophagic cell death/mitophagy	AIFM1 (if not co-activator)	AIFM1 (if not main activator)	(22)

Note 1: “Unclear if this is target gene.” indicates that mainly because the only reporter assay using major activator/repressor was performed, thus direct link between DAXX/PML/AIFM1/HDACs and the listed gene is unclear.
Note 2: Except for the cases of Note 1, other genes are also omitted in this table for simplicity, if it is unclear whether the target gene of either of DAXX/PML/AIFM1/HDACs or not.
Note 3: “Limited in reporter assay.” indicates that this gene is likely to be the target gene, but the data is limited in reporter assay.

Table S7. RT-qPCR primers used in this study

O number	ON or ONYX number	Gene symbol	Forward (5' - 3')	Reverse (5' - 3')
O 464/465	*ON 464/465*	*ACTB*	*CACCATTGGCAATGAGCGGTTC*	*AGGTCTTTGCGGATGTCCACGT*
O 1255/1256	*ONYX 842/843*	*BTK*	*GTTCAGCAGCAAATCTGAC*	*CCAGGGAGTAAATTTCCCAC*
O 1195/1160	*ONYX 782/783*	*CCND2*	*TCATTGAGCACATCTTGCG*	*AACTTAAAGTCGGTGGCAC*
O 1211/1212	*ONYX 798/799*	*CDK6*	*ACCTACTTCTGAAGTGTTTGAC*	*TCCTGGAAGTATGGGTGAG*
O 1199/1200	*ONYX 786/787*	*DDIT3*	*CATGTTAAAGATGAGCGGGTG*	*CACTTCCTTCTTGAACACTCTC*
O 1249/1250	*ONYX 836/837*	*DNMT3L*	*TACGACCGAGAGTCGGAGAAT*	*GCCCAAACTCGTCAGCTCTTT*
O 1225/1226	*ONYX 812/813*	*DYNC2LI1*	*TGATGGAGCTGAAATTGCA*	*GGTTCATCTCTGTCAAGACAC*
O 1227/1228	*ONYX 814/815*	*EFCAB2*	*CTGATTGCAGAGGTAGAGGA*	*TAGTATTTCTGTCATCACCGGA*
O 1205/1206	*ONYX 792/793*	*EFNA3*	*ATGAAGGTGTTCGTCTGCT*	*TCAAAGTCTTCCAGCACGT*
O 1215/1216	*ONYX 802/803*	*FAT4*	*TGTCAATGACAATCCACCA*	*ACAAAGGAACCTGGAAGAG*
O 1203/1204	*ONYX 790/791*	*FLT3LG*	*GTGAGCTGTCTGACTACCTG*	*CAAGCCTTGCATCTTGGAC*
O 1219/1220	*ONYX 806/807*	*GNG10*	*GTGGAGAGGATCAAGGTCTC*	*TACTTCCAGCTGGAACACC*
O 1217/1218	*ONYX 804/805*	*GNG4*	*TATGGACAGGGTCAAGGTC*	*ACGTGAGCTTCACAGTAGG*
O 1243/1244	*ONYX 830/831*	*H2BC17*	*ATCTCATCGAAGGCCATGG*	*CGATGCGCTCAAAGATGTC*
O 1241/1242	*ONYX 828/829*	*H2BC4*	*AAAGCGCAGAAGAAAGATGG*	*ACACAGAGTAACTCTCCTTGC*
O 1251/1252	*ONYX 838/839*	*IDO1*	*TGACTGGATGTTCATTGCT*	*GCTGAGCATGTTTAACTTCTC*
O 1207/1208	*ONYX 794/795*	*IFIT3*	*AACAGCCATCATGAGTGAG*	*AAGTTCCAGGTGAAATGGC*
O 1257/1258	*ONYX 844/845*	*LGALS9*	*ATTTCAAGGTGATGGTGAACG*	*ATTGACGGAGATGGTGTCC*
O 1231/1232	*ONYX 818/819*	*MED13*	*CAGAGCACAGAACAAGAGG*	*CAGGATGCCACTTTAGCAG*
O 1233/1234	*ONYX 820/821*	*MED7*	*AGCTGAGGTTCTTGATAGTC*	*GCCAAACATCATGTAACTGTC*
O 1245/1246	*ONYX 832/833*	*MND1*	*AGAGAACTCGCATGATGGA*	*GGAGCAATCTTCTCCAAGTC*
O 1235/1236	*ONYX 822/823*	*MYCBP2*	*GAGTGATGAAGGAGAGGCA*	*AGAGCTTGTTCATCTTCATGTC*
O 1237/1238	*ONYX 824/825*	*NOX5*	*AGTATCATGTACAGGCACCA*	*GTTGTCTTGGACACCTTCG*
O 1221/1222	*ONYX 808/809*	*ODAD2*	*AGCACAATTACTTCGGAAGAG*	*TCTTCATCTTCCTCACTTTCTG*
O 1213/1214	*ONYX 800/801*	*PCDHB13*	*CTCAGGTCTACATCGAAGTC*	*AAAGGCTGCTCAAATTCAGG*
O 1253/1254	*ONYX 840/841*	*PDCD1*	*AGGATGGTTCTTAGACTCCC*	*GATGTGTTGGAGAAGCTGC*
O 1239/1240	*ONYX 826/827*	*RORC*	*CAAGACTCATCGCCAAAGC*	*AAGATCTGCAGCCTTTCCA*
O 1247/1248	*ONYX 834/835*	*SMC1B*	*GCTGTTGATAGGGAAGTGG*	*CTTCTCTAATCGTTTCTGTTCC*
O 1223/1224	*ONYX 810/811*	*SPA17*	*CATTCGAGGAGCAAGAACC*	*ATGTCTCTTCCTCCTTCCC*
O 1229/1230	*ONYX 816/817*	*TBXAS1*	*AGCTGAACGAGATGGTTCC*	*CTGGATGTCAAATGCGTCC*
O 1201/1202	*ONYX 788/789*	*ZNG1F*	*CATAAGGCTGAAGGGATTGG*	*AGATCACAGAGCTCATGGAC*
O 1289/1290	*ONYX 863/864*	*SNF8*	*TCGCCCAGGATGTCAGTCA*	*CACGGTGTGATCCATATTGAGC*
O 1291/1292	*ONYX 865/866*	*CHMP2A*	*CGCGAGCGACAGAAACTAGAG*	*CCCGCATCAATACAAACTTGC*
O 1293/1294	*ONYX 867/868*	*ALIX (PDCD6IP)*	*ATGGCGACATTCATCTCGGTG*	*CGCTTGGGTAAGTCTGCTGG*

Table S8. Major characteristics of MOID

No.	Cellular characteristics of MOID	Assay name	Figure number (only firstly shown figure; other figures are omitted)
1	DNA double-strand break	TUNEL assay	Figures 1A-1C
2	p53-independent cell death	TUNEL assay	Figures 1A-1D and S2E-S2G
3	AIFM1-dependent cell death	TUNEL assay	Figures 1F and 1G
4	Long-term cell death (growth inhibition)	Colony outgrowth assay	Figure 1H
5	Cell death In vivo environment (microenvironment)	Zebrafish and mouse xenografts	Figures 1I-1N
6	AIFM1-nuclear translocation	Immunofluorescence and subcellular fractionation	Figures 2E-2G and 2I
7	Diffused/reduced MitoTracker pattern	MitoTracker immunofluorescence	Figures 2H and 6E
8	Contribution of dephosphorylation of C-terminal TSG101 including Y390	TUNEL assay	Figures 3C and 3D
9	Requirement of C-MAD2 overexpression	SUMOylation (immunoprecipitation) and TUNEL assays	Figures 3E-3G
10	Multimerization and SUMOyaltion of overexpressed C-MAD2 at late stage (120 h post-transfection)	SUMOylation (immunoprecipitation) assay	Figure 3H
11	Contribution of dephosphorylation of TSG101 Y390 and its interaction with C-MAD2	SUMOylation (immunoprecipitation) and TUNEL assays	Figures 3I-3L
12	Contribution of DeSUMOyaltion of PML	SUMOylation (immunoprecipitation) and TUNEL assays	Figures 3G and 4D-4G
13	Reduced PML (NBs)signals and MAD2-PML colocalization	Immunofluorescence	Figures 4H-4K
14	205 upregulated and 269 downregulated mRNAs compared with TSG101 siRNA sample (e.g., Upregulated BTK and H2BC4 and downregulated H2BC17)	Bulk RNA-seq (transcriptome) analysis	Figure 5 (e.g., Figures 5L and 5N)
15	ROS induction through DCFH-DA probe	ROS detection through DCFH-DA probe	Figures 6A and 6B
16	Increased BECN1 and LC3 signals	Immunofluorescence	Figures 6C and 6D

17	Association with changes of immunofluorescence signals of ER-MAM proteins	Immunofluorescence	Figures 6H-6M
18	ESCRT I-III-independent cell death pathway	TUNEL assay	Figure S1F-S1H
19	TSG101 release from MAD2-TSG101 interaction	Immunoprecipitation assay	Figure S4F
20	Caspase activation	FAM-FLICA caspase assay (FAM-VAD-FMK FLICA, poly caspase assay)	Figures S4G and S4H
21	PML-NB-negative and Low DAPI+PML-NB-negative cells are increased	Immunofluorescence	Figures S8D and S8E
22	Chromsome instability correlated (interphase micronuclei and cytokinesis [chromosome bridge-type] cells are especially increased)	Immunofluorescence	Figures S9

Table S9. Cell lines used in this study

Database number	Cell line name	Purchased/Gifted year (including STR/micoplasma test)	Purchased organization	Catalog number	Reference
CS49	HeLa	2021	National Collection of Authenticated Cell Cutures, China	TCHu187	This study
CS51	293T	2021	National Collection of Authenticated Cell Cutures, China	GNHu17	This study
CS19	HSkMC	2019	Shanghai Jining Bio, China	JN27282	This study
CS54	A549	2022	Gifted by Jianghuai Liu, Model Animal Research Center, Medical School of Nanjing University; Originally purchased from ATCC	CCL-185	(23)
CS53	A549 p53 KO	2022	Gifted by Jianghuai Liu, Model Animal Research Center, Medical School of Nanjing University; Established by Jianghuai Liu lab from ATCC, CCL-185.	N.A.	This study

Supplemental Figure Legends

Figure S1. Abnormal mitotic progression in TSG101-depleted HeLa cells, and depletion of other ESCRT components did not show MOID, neither rescued MOID in MAD2-overexpressing cells (related to Figure 1).

(A) Depletion of TSG101 significantly increased Mitotic index (MI). HeLa cells were transfected with indicated siRNAs (**Table S1**) and cells were cultured for 72 h at 37°C. Mitotic cells were observed by DAPI stain, and more than 200 interphase cells were counted per experiment ($n \geq 3$ experiments), and the mean percentages (±SD) are shown. (**) $P < 0.01$ compared with Luc siRNA-treated cells (Student's t test).

(B) Western blot analysis of total lysates of HeLa cells transfected with indicated siRNAs (**Table S1**). Cells were cultured for 72 h, collected, lysed, and immunoblotted with indicated antibodies (**Table S3**). GAPDH protein was used as a loading control. Percentage (%) of anti-TSG101 western blot signals compared with the signal of the Luc siRNA sample is indicated (normalized to anti-GAPDH signals).

(C) Abnormal metaphase was observed by DAPI stain in TSG101 siRNA (TSG101 #S1; **Table S1**)-treated Hela cells 72 h after the transfection. Arrows indicate misaligned metaphase chromosomes. Scale bar, 10 µm.

(D) Abnormal anaphase B or telophase was observed by DAPI stain in TSG101 siRNA (TSG101 #S1; **Table S1**)-treated Hela cells. Arrows indicate lagging chromosomes. Scale bar, 10 µm.

(E) Histogram summarizing mitotic index, anaphase B or telophase cells with lagging chromosome, misaligned metaphase cells, abnormal metaphase cells shown in (C) and (D). Mean percentages of followings are shown. Mitotic index (MI) (±SD; $n \geq 3$ experiments of more than 200 cells per experiment). Misaligned, misaligned

metaphase cell (arrows in [C]) (±SD; n ≥ 5 experiments of more than 50 metaphase cells per experiment). 3 MTOC, cell with 3 microtubule-organizing centers (MTOCs) (±SEM; n ≥ 5 experiments of more than 50 metaphase cells per experiment). ≥4 MTOC, cell with four or more microtubule-organizing centers (MTOCs) (±SEM; n ≥ 5 experiments of more than 50 metaphase cells per experiment). Lagging Chr., anaphase B or telophase cell with lagging chromosome (arrows in [D] (±SEM; n ≥ 3 experiments of more than 50 anaphase B or telophase cells per experiment).

(F) RT-qPCR analysis to verify mRNA depletion of different ESCRT components: TSG101 (ESCRT-I), SNF8 (ESCRT-II), CHMP2A (ESCRT-III), and ALIX (PDCD6IP) in the accessory complex. The optimized time length of cell culture is 48 h for SNF8 and CHMP2A, and 72 h for ALIX (PDCD6IP). (****) $P < 0.0001$ and (***) $P < 0.001$ compared with each control (Ctrl) (Student's t-test).

(G) A histogram summarizing TUNEL assay results of HeLa cells transfected with the indicated siRNAs and/or constructs. sRNAs of different ESCRT components were tested with Flag-MAD2 overexpression: TSG101 (ESCRT-I), SNF8 (ESCRT-II), CHMP2A (ESCRT-III), and ALIX (PDCD6IP) in the accessory complex. The optimized time length of cell culture after the 2nd siRNA transfection of each ESCRT component is 48 h for SNF8 and CHMP2A, and 72 h for ALIX (PDCD6IP). The mean percentages (± SD) of TUNEL-positive cells were shown. (****) $P < 0.0001$ compared with 3rd column from right and not significant (ns) compared to each control in the same set of 2nd transfection (Student's t-test).

(H) A histogram summarizing TUNEL assay results of HeLa cells transfected with the indicated siRNAs and/or constructs. sRNAs of different ESCRT components were tested with Flag-MAD2 overexpression: TSG101 (ESCRT-I), SNF8 (ESCRT-II), CHMP2A (ESCRT-III), and ALIX (PDCD6IP) in the accessory complex. The

optimized time length of cell culture after the 2nd siRNA transfection of each ESCRT component is 48 h for SNF8 and CHMP2A, and 72 h for ALIX (PDCD6IP). The mean percentages (± SD) of TUNEL-positive cells were shown. (****) $P < 0.0001$ compared with 3rd column from right (Student's t-test).

(I) Mitotic index increased with either MAD2 overexpression or TSG101 depletion, and these 2 effects showed an additive (synthetic) tendency. HeLa or 273T cells were transfected with indicated siRNAs (**Table S1**) and/or constructs (**Table S2**), and cells were cultured for 96 h at 37°C. Mitotic cells were observed by DAPI stain. For Flag-MAD2 overexpressing cells, more than 200 interphase Flag+TUNEL+ (double positive) cells in were counted per experiment (n ≥ 3 experiments). For Flag-vector (Flag-Vec) overexpressing or DNAse-treated cells, more than 400 interphase TUNEL-positive cells were counted per experiment (n ≥ 3 experiments) considering their Flag-negative signals and approximate transfection efficiency (ca. more than 50%) of overexpression vector. The mean percentages (±SD) are shown. Asterisks (*) and hashtags (#) are put as described in **MATERIALS AND METHOD** (Statistical Analysis) (Student's t test).

(J) A histogram summarizing TUNEL assay results of the samples shown in (I). More than 50 mitotic cells were counted per experiment (n ≥ 3 experiments). Cells treated with DNAse (50 μg/ml, 10 min) after fixation is shown as a control to verify TUNEL positivity. The mean percentages (±SD) are shown. Asterisks (*) are put as described in **MATERIALS AND METHOD** (Statistical Analysis) (Student's t test).

Figure S2. MOID occurs commonly in MAD2-overexpressing human cells (related to Figure 1).

(A) Fluorescence images of TUNEL assay. 293T cells were transfected with indicated siRNAs and/or constructs. TUNEL assay was performed as **Figure 1A**. Scale bar, 10 μm.

(B) Western blot analysis of total lysates of 293T cells transfected with indicated siRNAs and/or constructs. Cells were cultured for 96 h, collected, lysed, and immunoblotted with indicated antibodies (**Table S3**). GAPDH protein was used as a loading control.

(C) Fluorescence images of TUNEL assay. Human primary skeletal muscle cells (HSkMCs) were transfected with indicated siRNAs and/or constructs. TUNEL assay was performed as **Figure 1A**. Scale bar, 10 μm.

(D) (Left and Right) Western blot analysis of total lysates of HSkMC cells transfected with indicated siRNAs and/or constructs. Cells were cultured for 96 h, collected, lysed, and immunoblotted with indicated antibodies (**Table S3**). GAPDH protein was used as a loading control.

(E) Fluorescence images of TUNEL assay. A549 cells were transfected with indicated indicated siRNAs and/or constructs. TUNEL assay was performed as **Figure 1A**. Scale bar, 10 μm.

(F) Fluorescence images of TUNEL assay. A549 p53 KO cells were transfected with indicated siRNAs and/or constructs. TUNEL assay was performed as **Figure 1A**. Scale bar, 10 μm.

(G) Western blot analysis of total lysates of A549 and A549 (p53 KO) cells transfected with indicated siRNAs and/or constructs. Cells were cultured for 96 h, collected, lysed, and immunoblotted with indicated antibodies (**Table S3**). GAPDH protein was used as a loading control.

(H) A histogram summarizing TUNEL assay results of (A) and (C). 293T or HSkMC cells were transfected with indicated siRNAs and/or constructs. Cells were cultured for 96 h at 37°C. DNA fragmentation was detected by the TUNEL assay as described in **Figure 1A** (see **MATERIALS AND METHODS**). Cells treated with DNAse (50 μg/ml [SIGMA] for 10 min) after fixation is shown as a control to verify TUNEL positivity. The mean percentages (± SD) of TUNEL-positive cells were shown. (****) $P < 0.0001$ compared with 1st column from left in each cell (Student's t-test).

Figure S3. MOID reduces the viability of HSkMCs and Rh30 cells in zebrafish xenografts (see Table S5 for simplified sample indication) (related to Figure 1).
(A) (Left) Colony outgrowth assay. MOID reduces the viability of HSkMCs. HSkMCs were spread with 500 cells/well (see **MATERIALS AND METHODS**). (Right) Colony counts and area (%) normalized to the control (1st column from left) are shown summarizing the results of (Left). The mean percentages (± SEM) were shown. (**) $P < 0.01$ and (*) $P < 0.05$ (Student's t-test).
(B) (Left) Colony outgrowth assay. MOID reduces the viability of HeLa cells. HeLa cells were spread with 1000 cells/well (see **MATERIALS AND METHODS).** (Right) Colony counts and area (%) normalized to the control (1st column from left) are shown summarizing the results of (Left). The mean percentages (± SEM) were shown. (***) $P < 0.001$ and (**) $P < 0.01$ (Student's t-test).
(C) Western blot analysis to detect endogenous MAD2 protein expression level in HSkMC and Rh30 cell lysates. β-actin (ACTB) protein was used as loading control.
(D) Representative images of zebrafish xenograft after the transplantation of Rh30 cells with indicated variations. Scale bar, 500 μm.

(E) Rh30 cells-grown area and volume (%) are shown summarizing the results of (D). (Left) A line chart from 2-6 dpi. Data of each sample at 2 dpi was normalized to 100 %. (**) $P < 0.01$ and (*) $P < 0.05$ (one-way ANOVA t-test). (Right) A histogram of 6 dpi. Data of each sample at 2 dpi was normalized to 100 %. The mean percentages (± SEM) were shown. (****) $P < 0.0001$, (***) $P < 0.001$, (**) $P < 0.01$, and (*) $P < 0.05$ (Student's t-test).

(F) Representative images of zebrafish xenograft after the transplantation of HeLa cells with indicated variations. Scale bar, 500 μm.

(G) HeLa cells-grown area and volume (%) are shown summarizing the results of (F). (Left) A line chart from 2-6 dpi. Data of each sample at 2 dpi was normalized to 100 %. (****) $P < 0.0001$ and (***) $P < 0.001$ (one-way ANOVA t-test). (Right) A histogram of 6 dpi. Data of each sample at 2 dpi was normalized to 100 %. The mean percentages (± SEM) were shown. (****) $P < 0.0001$, (***) $P < 0.001$, (**) $P < 0.01$ and (*) $P < 0.05$ (Student's t-test).

Figure S4. MOID is p53-independent but AIFM1-dependent cell death (related to Figure1).

(A) Flag-MAD2 interacts with endogenous p53. Immunoblot analysis of Flag-MAD2 immunoprecipitates. 293T cells were transfected with indicated constructs. Cells were cultured 48 h, collected, lysed, and proteins in the total cell lysate (Input) and precipitate (IP) with anti-Flag (DYKDDDDK) Affinity Beads (Smart-Lifesciences, China; **Table S3**, A151) were detected with indicated antibodies (**Table S3**). The volume of the total cell lysate was 5% of that of the starting material.

(B) Western blot analysis of total HSkMC cell lysates harvested 96 h after transfection with indicated siRNA (**Table S1**) revealed depletion of p53. GAPDH protein was the loading control.

(C) Western blot analysis of total HSkMC cell lysates harvested 72 h after transfection with indicated siRNA (**Table S1**) revealed depletion of AIFM1. GAPDH protein was the loading control.

(D) Western blot analysis of total A549 cell lysates harvested 72 h after transfection with indicated siRNA (**Table S1**) revealed depletion of AIFM1 and TSG101. GAPDH protein was the loading control.

(E) A histogram summarizing TUNEL assay results. 293T or HSkMC cells were transfected with indicated siRNAs and/or constructs. Cells were cultured for 96 h at 37°C. DNA fragmentation was detected by the TUNEL assay as described in **Figure 1A** (see **MATERIALS AND METHODS**). The mean percentages (± SD) of TUNEL-positive cells were shown. (****) $P < 0.0001$ compared with 1st column from left (Student's t-test).

(F) Overexpressed Flag-MAD2 interacts with both AIFM1 and TSG101 in non-MOID cells, and increases affinity with AIFM1 during MOID. 293T cells were transfected with the indicated constructs, and pellets were collected 96 h after the transfection, which is the timing when TUNEL-positive MOID cells were observed. Proteins in 5% of the total cell lysates (Input) and immunoprecipitates (IP) were detected by western blot analysis using the indicated antibodies (**Table S3**).

(G) MOID is caspase-activated cell death. HeLa cells were transfected with indicated siRNAs and/or constructs. Cells were cultured 95 h at 37°C, then incubated with the FAM-VAD-FMK FLICA (fluorochrome inhibitor of caspases) solution (Immunochemistry Technologies, LLC) for 60 min at 37°C, and samples underwent

indirect fluorescence microscopy using anti-Flag as a primary antibody to sort out Flag-MAD2 overexpressing cells (see **MATERIALS AND METHODS**). As a positive control, HeLa cells were treated with 1 μM staurosporine (STS) for 6 h, and HSkMC and A549 p53 KO cells were treated with 100 μM staurosporine (STS) for 6 h. Scale bar, 10 μm.

(H) A histogram summarizing FAM-FLICA caspase assay results of (G). The mean percentages (± SD) of interphase FLICA-positive cells were shown. (****) $P < 0.0001$ compared with 1st column from left in each cell (Student's t-test).

Figure S5. MAD2, TSG101, and AIF-PML-DAXX axis regulate mitochondria, PML NBs, and autophagy (related to Figure 2).

(A) The percentage of mitochondrial proteins out of total proteins identified in the liquid chromatography-tandem mass spectrometry (LC-MS/MS). Grey bars indicate the cases where no Vector-interacting peptide background was observed, and white bars indicate the cases where the peptides' number in Flag-proteins' precipitants were higher more than 2-fold in the Vector's precipitants (see **MATERIALS AND METHODS)**.

(B) The percentage of mitochondrial proteins out of total proteins identified in the liquid chromatography-tandem mass spectrometry (LC-MS/MS). The analysis was performed as (A).

(C) Western blot analysis of total HeLa cell lysates harvested 6 days after transfection with PML CRISPRi vector (**Table S1**) revealed depletion of PML. GAPDH protein was the loading control (see **MATERIALS AND METHODS**).

(D) Western blot analysis of total HeLa cell lysates harvested 6 days after transfection with DAXX CRISPR KO (**Table S1**) revealed depletion of DAXX. GAPDH protein was the loading control (see **MATERIALS AND METHODS**).

(E) Western blot analysis of total HeLa cell lysates harvested 4 days after transfection with TSG101 siRNA (**Table S1**) revealed depletion of TSG101. GAPDH protein was the loading control (see **MATERIALS AND METHODS**).

(F) PML depletion reduced the MitoTracker signals in metaphase. HeLa cells were transfected for 72 hr with PML CRISPRi or control (Ctrl) vector (**Table S1**). MitoTracker signals were quantitated. Signals were normalized with Ctrl cells, and the mean percentages (±SEM) are shown. P value is shown compared with Ctrl cells (Student's t test).

(G) DAXX depletion increased the colocalization between MitoTracker and LC3B signals in interphase. HeLa cells were transfected for 72 hr with DAXX CRISPR KO or control (Ctrl) vector **(Table S1**). Cells were cultured with NH_4Cl (20 mM) at 54-72 h after transfection (for 18 h before the fixation; see **MATERIALS AND METHODS**). Pearson correlation coefficient was obtained. The mean values (±SEM) are shown. P value is shown compared with Ctrl cells (Student's t test).

(H) DAXX depletion increased the PML signals in interphase. HeLa cells were transfected for 72 hr with DAXX CRISPR KO or control (Ctrl) vector (**Table S1**). PML signals were quantitated. Signals were normalized with Ctrl cells, and the mean percentages (±SEM) are shown. P value is shown compared with Ctrl cells (Student's t test).

(I) DAXX depletion decreased the colocalization between MitoTracker and PML signals in metaphase. HeLa cells were transfected for 72 hr with DAXX CRISPR KO or control (Ctrl) vector (**Table S1**). Pearson correlation coefficient was obtained. The

mean values (±SEM) are shown. P value is shown compared with Ctrl cells (Student’s t test).

(J) TSG101 depletion reduced the MitoTracker signals in interphase and metaphase, and the PML signals in interphase. Immunofluorescence images of cells transfected with the indicated siRNA (**Table S1**) are shown. Cells were cultured for 96 h at 37°C, and samples underwent indirect fluorescence microscopy using the indicated antibodies (see **MATERIALS AND METHODS**).

(K) TSG101 depletion reduced the MitoTracker signals in interphase. HeLa cells were transfected for 96 hr with TSG101 siRNA or control (Ctrl) vector (**Table S1**). MitoTracker signals were quantitated. Signals were normalized with Ctrl cells, and the mean percentages (±SEM) are shown. P value is shown compared with Ctrl cells (Student’s t test).

(L) TSG101 depletion reduced the MitoTracker signals in metaphase. HeLa cells were transfected for 96 hr with TSG101 siRNA or control (Ctrl) vector (**Table S1**). MitoTracker signals were quantitated. Signals were normalized with Ctrl cells, and the mean percentages (±SEM) are shown. P value is shown compared with Ctrl cells (Student’s t test).

(M) TSG101 depletion decreased the colocalization between MitoTracker and PML signals in metaphase. HeLa cells were transfected for 96 hr with TSG101 siRNA or control (Ctrl) vector (**Table S1**). Pearson correlation coefficient was obtained. The mean values (±SEM) are shown. P value is shown compared with Ctrl cells (Student’s t test).

(N-P) TSG101 depletion decreased the PML signals in (N) interphase, but did not affect the PML signals in (O) pro/prometaphase and (P) metaphase. HeLa cells were transfected for 96 hr with TSG101 or control (Ctrl) siRNA (**Table S1**). PML signals

were quantitated. Signals were normalized with Ctrl cells, and the mean percentages (±SEM) are shown. P value is shown compared with Ctrl cells (Student's t test). ns, not significant.

Figure S6. MAD2, TSG101, and AIF-PML-DAXX axis are closely inter-dependent in protein stability, and apoptotic functions of PML and DAXX during MOID are TSG101-dependent (related to Figures 2 and 3).

(A-C) (Left) Protein stability assay was performed (see **MATERIALS AND METHODS)**. Total cell lysates were analyzed for endogenous protein levels with indicated antibodies at the indicated time points. GAPDH protein was used as a loading control. (Right) Histograms summarizing quantitated protein levels. Each protein level is normalized with the GAPDH. The mean percentages (± SEM) were shown.

(D) A histogram summarizing TUNEL assay results. 293T or HSkMC cells were transfected with indicated siRNAs and/or constructs. Cells were cultured for 96 h at 37°C. DNA fragmentation was detected by the TUNEL assay as described in **Figure 1A** (see **MATERIALS AND METHODS**). The mean percentages (± SD) of TUNEL-positive cells were shown. (****) *P* < 0.0001 compared with 1st column from left in each cell (Student's t-test). (##) *P* < 0.01 (Student's t-test).

(E) A histogram summarizing TUNEL assay results. 293T or HSkMC cells were transfected with indicated siRNAs and/or constructs. Cells were cultured for 96 h at 37°C. DNA fragmentation was detected by the TUNEL assay as described in **Figure 1A** (see **MATERIALS AND METHODS**). The mean percentages (± SD) of TUNEL-positive cells were shown. iVEC indicates CRISPRi vector control. (****) *P* < 0.0001 compared with 1st column from left in each cell (Student's t-test).

(F) TSG101 Y390F mutant loses the colocalization with PML NBs without affecting the integrity of PML NBs. Immunofluorescence images of cells transfected with the indicated siRNA (**Table S1**) are shown. Cells were cultured for 96 h at 37°C, and samples underwent indirect fluorescence microscopy using the indicated antibodies (see **MATERIALS AND METHODS**).

(G) Pearson correlation coefficient between anti-Flag(TSG101) and anti-PML in the interphase nuclear shown in (F) was obtained. The mean values (±SEM) are shown. Asterisks (*) of P value are shown compared with WT (Student's t test; **MATERIALS AND METHODS**).

(H) PML signals in the interphase nuclear shown in (F) were quantitated. Signals were normalized with WT, and the mean percentages (±SEM) are shown. ns, not significant (Student's t-test).

(I) Histogram summarizing normal and abnormal nuclei cells shown in (F). Type 1, apparently normal DAPI signals but Flag(TSG101) signals are diffused; Type 2, condensed DAPI signals; Type 3, weakened DAPI signals; Type 4, plural nuclear (≥ 2N). The mean values (±SD) of each type are shown. Only asterisks (*) of P value of normal nuclei are shown compared with WT for the simplicity (Student's t test; **MATERIALS AND METHODS**).

(J) A histogram summarizing TUNEL assay results of samples transfected and cultured as (F) (see **MATERIALS AND METHODS**). The mean percentages (± SD) of TUNEL-positive cells were shown. Asterisks (*) of P value are shown compared with WT (Student's t test; **MATERIALS AND METHODS**). ns, not significant.

(K) Western blot analysis of 293Tcell total lysates collected 48 h after transfection with indicated constructs (**Table S2**) and immunoblotted with indicated antibodies (**Table S3**). GAPDH protein was the loading control.

Figure S7. TSG101 Y390 phosphorylation is required for TSG101 localization to PML NBs, and loss of TSG101 Y390 phosphorylation and C-MAD2-overexpression contribute to induce MOID, while deSUMOylated PML delta coiled-coil (dCC) mutant constitutively induces MOID (related to Figures 3 and 4).

(A) Majority of overexpressed O-MAD2 binds Y390-intact TSG101 WT, while overexpressed C-MAD2 binds TSG101 Y390F mutant. Immunoprecipitation assay (see **MATERIALS AND METHODS**). HeLa cells were transfected with the indicated constructs, and pellets were collected 96 h after the transfection, which is the timing when TUNEL-positive MOID cells were observed. Proteins in 10.7% of the total cell lysates (Input) and immunoprecipitates (IP) were detected by western blot analysis using the indicated antibodies.

(B) A histogram summarizing TUNEL assay results corresponding to the samples shown in (A), but using untagged-TSG101. The mean percentages (± SD) of TUNEL-positive cells were shown.

(C) A histogram summarizing TUNEL assay results corresponding to the samples shown in (A) using HA-TSG101. Proteins in 3.0% of the total cell lysates (Input) and immunoprecipitates (IP) were detected by western blot analysis using the indicated antibodies.

(D) Western blot analysis of 293T cell total lysates collected 48 h after transfection with indicated constructs (**Table S2**) and immunoblotted with indicated antibodies (**Table S3**). GAPDH protein was the loading control.

(E) Representative images of the in vivo SUMOylation assay with the HA-PML constructs (WT and mutants) using HeLa cells (see **MATERIALS AND METHODS**). For input, GAPDH protein was used as a loading control.

(F) PML coiled-coil domain is required for PML NBs formation. Immunofluorescence images of cells transfected with the indicated constructs (**Table S2**) are shown (see **MATERIALS AND METHODS**).

(G) A histogram summarizing the localization patterns shown in (F). HeLa More than 200 interphase cells were counted per experiment ($n \geq 3$ experiments), and the mean percentages (±SD) are shown. ‘‘Others (Non-PML NB)’’ indicates mostly damaged cells, dead cells, or cells with nucleolar localization (in interphase) presumaby due to transfection or other treatments. **** $P < 0.0001$ and ** $P < 0.01$ compared with 3xFlag-PML WT (Student’s t test).

(H) DeSUMOylated PML delta coiled-coil (dCC) mutant failed to suppress MOID induction with Flag-MAD2 overexpression and single PML depletion, and constitutively induces MOID. 293T cells were transfected with the indicated sgRNAs, siRNA, and/or constructs. Cells were cultured for 96 h at 37°C. DNA fragmentation was detected by the TUNEL assay as described in **Figure 1A** (see **MATERIALS AND METHODS**). Scale bar, 10 μm.

(I) A histogram summarizing TUNEL assay results shown in (H). HeLa cells were transfected with indicated siRNAs and/or constructs. Cells were cultured for 96 h at 37°C. DNA fragmentation was detected by the TUNEL assay as described in **Figure 1A** (see **MATERIALS AND METHODS**). The mean percentages (± SD) of TUNEL-positive cells were shown. Asterisks (*) and hashtags (#) are put as described in **MATERIALS AND METHOD** (Statistical Analysis) (Student’s t test).

(J) A histogram summarizing TUNEL assay results of indicated samples detected as (I). The mean percentages (± SD) of TUNEL-positive cells were shown. Asterisks (*) and hashtags (#) are put as described in **MATERIALS AND METHOD** (Statistical Analysis) (Student's t test).

(K) A histogram summarizing ROS detection (fluorescence DCF intensity). HeLa cells were transfected with the indicated sgRNA, siRNA, and/or, constructs, and pellets were collected 96 h after the transfection as TUNEL-positive MOID cells were observed in (J). The mean percentages (± SD) were shown. Asterisks (*) are put as described in **MATERIALS AND METHOD** (Statistical Analysis) (Student's t test).

(L-O) histograms summarizing indicated fluorescence signals. Samples were collected as (K). For MAD2 and AIFM1 quantitations, nuclear regions were contoured, while for MitoTracker and BECN1 quantitations, whole cell regions were contoured. The mean percentages (± SEM) were shown. Asterisks (*) and hashtags (#) are put as described in **MATERIALS AND METHOD** (Statistical Analysis) (Student's t test).

Figure S8. PML release from PML NBs through PML deSUMOylation is required to induce MOID (related to Figure 4).

(A) In vivo SUMOylation assay of HA-PML using HeLa cells. Cells were transfected with the indicated constructs, and pellets were collected 96 h after the transfection, which is the timing when TUNEL-positive MOID cells were observed. Proteins in 5% of the total cell lysates (Input) and immunoprecipitates (IP) with anti-HA Affinity Beads (Smart-Lifesciences, China; **Table S3**, A194) were detected by western blot analysis using the indicated antibodies (see **Figure 4F** and **MATERIALS AND METHODS**).

(B and C) In vivo SUMOylation assay of endogenous PML using HeLa cells. Cells were transfected with the indicated constructs, and pellets were collected 96 h after the transfection, which is the timing when TUNEL-positive MOID cells were observed. Proteins in 5% of the total cell lysates (Input) and immunoprecipitates (IP) with anti-PML were detected by western blot analysis using the indicated antibodies. Note that the sample with TSG101 depleted without MAD2-overexpression is shown in (B, sample 4) (but not shown in [C]), and the sample where MOID is not rescued by TSG101 Y390F is shown in (C, sample 6) (but not shown in [B]).

(D) PML NB-negative cells are observed during MOID. A histogram summarizing the population of PML NB-negative cells in interphase of (Left) HeLa and (Right) 293T (see **Figure 4H** and **MATERIALS AND METHODS**). See **Figure 4H** for corresponding immunofluorescence images and sample numbers. The mean percentages (± SD) were shown. (***) $P < 0.0001$, (***) $P < 0.001$, (**) $P < 0.01$, and (*) $P < 0.05$ compared with sample [1] (**Table S5**) in each cell line (Student's t-test). (####) $P < 0.0001$, (###) $P < 0.001$, and (##) $P < 0.01$ compared with sample [4] (MOID condition; **Table S5**) in each cell line (Student's t-test). ns, not significant.

(E) Unique nuclear morphology with 'low DAPI/PML NB-negative' staining is observed in MOID. A histogram summarizing the population of 'weak DAPI and PML NB-negative' cells in interphase of (Left) HeLa and (Right) 293T (see **Figure 4H** and **MATERIALS AND METHODS**). See **Figure 4H** for corresponding immunofluorescence images and sample numbers. The mean percentages (± SD) were shown. (****) $P < 0.0001$, (***) $P < 0.001$, and (**) $P < 0.01$ compared with sample [1] (**Table S5**) in each cell line (Student's t-test). (####) $P < 0.0001$, (###) $P < 0.001$, and (##) $P < 0.01$ compared with sample [4] (MOID condition; **Table S5**) in each cell line (Student's t-test). ns, not significant.

(F) TSG101 depletion reduced the colocalization between MAD2 nuclear foci and PML signal in interphase but maintained the total signals of MAD2 nuclear foci in interphase. Immunofluorescence images of cells transfected with the indicated siRNA (**Table S1**) are shown. Cells were cultured for 96 h at 37°C, and samples underwent indirect fluorescence microscopy using the indicated antibodies (see **MATERIALS AND METHODS**).

(G) TSG101 depletion decreased the colocalization between MAD2 (interphase nuclear foci) and PML signals in interphase. (Left) HeLa cells or (Right) 293T were transfected for 96 hr with TSG101 siRNA or control (Ctrl) vector (**Table S1**). Pearson correlation coefficient was obtained. The mean percentages (±SEM) are shown. P value is shown compared with Ctrl cells (Student’s t test).

(H) TSG101 depletion did not affect the signals of MAD2 nuclear foci in interphase. (Left) HeLa cells or (Right) 293T were transfected for 96 hr with TSG101 siRNA or control (Ctrl) vector (**Table S1**). MAD2 interphase nuclear foci were quantitated. Signals were normalized with Ctrl cells, and the mean percentages (±SEM) are shown. ns, not significant.

Figure S9. Chromosome instability correlates with MOID (related to Figure 4).

(A and B) Histogram summarizing mitotic cells (from late G2 to Cytokinesis) of MOID samples ([4] is MOID; see **Table S5** for the sample variation) and other samples of (A) HeLa and (B) 293T cells. Mean percentages of mitotic cells (from late G2 to Cytokinesis; i.e., Phospho-histone H3 positive plus cytokinesis cells) out of total cells (±SD; $n \geq 3$ experiments of more than 200 total cells per experiment) are shown. The mitotic indexes of samples [2]-[4] in (A and B) are lower than in **Figure S1I**, and the synthetic effect of MAD2 overexpression or TSG101 depletion wasn't observed,

likely due to extra pcDNA5 transfection and/or transient hygromycin treatment for 2 days.

(C and D) Histogram summarizing micronuclei cells of control and MOID samples ([4] is MOID; see **Table S5** for the sample variation) of (C) HeLa and (D) 293T cells. Mean percentages of micronuclei cells out of total interphase cells (excluding DAPI-lost or damaged cells) (±SD; $n \geq 3$ experiments of more than 200 total cells per experiment) are shown. (****) $P < 0.0001$, (***) $P < 0.001$, and (**) $P < 0.01$ compared with sample 1 (**Table S5**) in each cell line (Student's t-test). (##) $P < 0.01$ compared with sample [4] (MOID condition; **Table S5**) in each cell line (Student's t-test). ns, not significant.

(E and F) Histogram summarizing cytokinesis (chromosome bridge-type) cells of control and MOID samples ([4] is MOID; see **Table S5** for the sample variation) of (E) HeLa and (F) 293T cells. Note that slight pro/prometaphase arrest was observed in single MAD2-overexpressing cells (samples [2] and [5] practically) that might have led to the reduction of cytokinesis population. Mean percentages of cytokinesis (chromosome bridge-type) cells out of mitotic cells (±SD; $n \geq 3$ experiments of more than 50 mitotic cells) are shown. (***) $P < 0.001$, (**) $P < 0.01$, and (*) $P < 0.05$, compared with sample 1 (**Table S5**) in each cell line (Student's t-test). (###) $P < 0.001$ and (##) $P < 0.01$ compared with sample [4] (MOID condition; **Table S5**) in each cell line (Student's t-test). ns, not significant.

(G and H) Representative images of micronuclei and cytokinesis (chromosome bridge-type) cells of (G) HeLa and (H) 293T cells. The sample transfected with vector plus control siRNA (sample [1]; see **Table S5** for the sample variation) is used as control that shows normal interphase nuclear. Arrows indicate micronuclei. Arrowheads indicate chromosome bridges. Scale bar, 10 μm.

Figure S10. Transcriptome RNA-seq analysis of MOID (related to Figure 5).

(A and B) Sample correlation between 2 individual RNA-seq samples. (A) Control samples; (B) MOID samples. Pearson Correlations are shown in the top.

(C and D) An overview of Gene Ontology (GO) enrichment analysis showing biological process (C), molecular function and cellular component (D) affected by up or downregulation of genes expression in MOID samples.

(E) Heatmaps depicting the relative expression levels of high-confidence top 50 genes in HeLa either Control or MOID group. Each row corresponds to a gene, and signal intensity is normalized across the row. Genes are ranked by P value with the lowest at the top.

(F) Relative gene expression status (down or up regulation compared with Control group) and description of top 7 differentially expressed genes (with most significant p value).

(G) Evaluation of top 7 differentially expressed genes by RT-qPCR in HeLa sample. All qPCR experiments were repeated ($n \geq 3$ replicates), and the mean ± SEM. is shown.

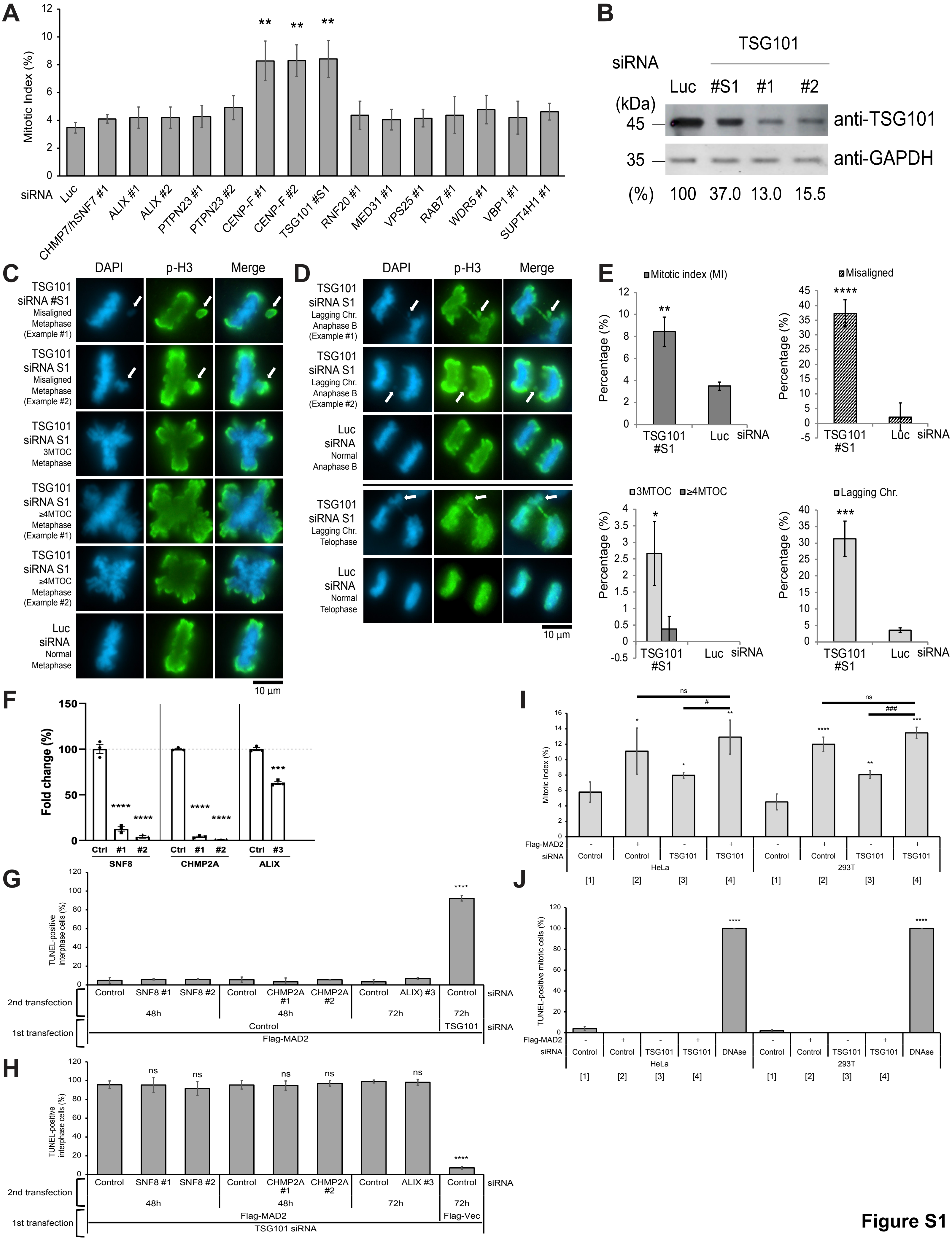
A
Mitotic Index (%)
siRNA
Luc
CHMP7/hSNF7 #1
ALIX #1
ALIX #2
PTPN23 #1
PTPN23 #2
CENP-F #1
CENP-F #2
TSG101 #S1
RNF20 #1
MED31 #1
VPS25 #1
RAB7 #1
WDR5 #1
VBP1 #1
SUPT4H1 #1
B
siRNA
TSG101
Luc #S1 #1 #2
(kDa)
45
35
anti-TSG101
anti-GAPDH
(%) 100 37.0 13.0 15.5
C
DAPI p-H3 Merge
TSG101 siRNA #S1 Misaligned Metaphase (Example #1)
TSG101 siRNA S1 Misaligned Metaphase (Example #2)
TSG101 siRNA S1 3MTOC Metaphase
TSG101 siRNA S1 ≥4MTOC Metaphase (Example #1)
TSG101 siRNA S1 ≥4MTOC Metaphase (Example #2)
Luc siRNA Normal Metaphase
10 µm
D
DAPI p-H3 Merge
TSG101 siRNA S1 Lagging Chr. Anaphase B (Example #1)
TSG101 siRNA S1 Lagging Chr. Anaphase B (Example #2)
Luc siRNA Normal Anaphase B
TSG101 siRNA S1 Lagging Chr. Telophase
Luc siRNA Normal Telophase
10 µm
E
Mitotic index (MI)
Misaligned
3MTOC ≥4MTOC
Lagging Chr.
Percentage (%)
TSG101 #S1
Luc
siRNA
F
Fold change (%)
Ctrl #1 #2
SNF8
Ctrl #1 #2
CHMP2A
Ctrl #3
ALIX
G
TUNEL-positive interphase cells (%)
Control SNF8 #1 SNF8 #2 Control CHMP2A #1 CHMP2A #2 Control ALIX) #3 Control siRNA
2nd transfection
48h 48h 72h 72h
1st transfection
Control TSG101 siRNA
Flag-MAD2
H
TUNEL-positive interphase cells (%)
Control SNF8 #1 SNF8 #2 Control CHMP2A #1 CHMP2A #2 Control ALIX #3 Control siRNA
2nd transfection
48h 48h 72h 72h
1st transfection
Flag-MAD2 Flag-Vec
TSG101 siRNA
I
Mitotic Index (%)
Flag-MAD2
siRNA
Control TSG101
HeLa
293T
[1] [2] [3] [4]
J
TUNEL-positive mitotic cells (%)
Flag-MAD2
siRNA
Control TSG101 DNAse
HeLa
293T
[1] [2] [3] [4]

Figure S1

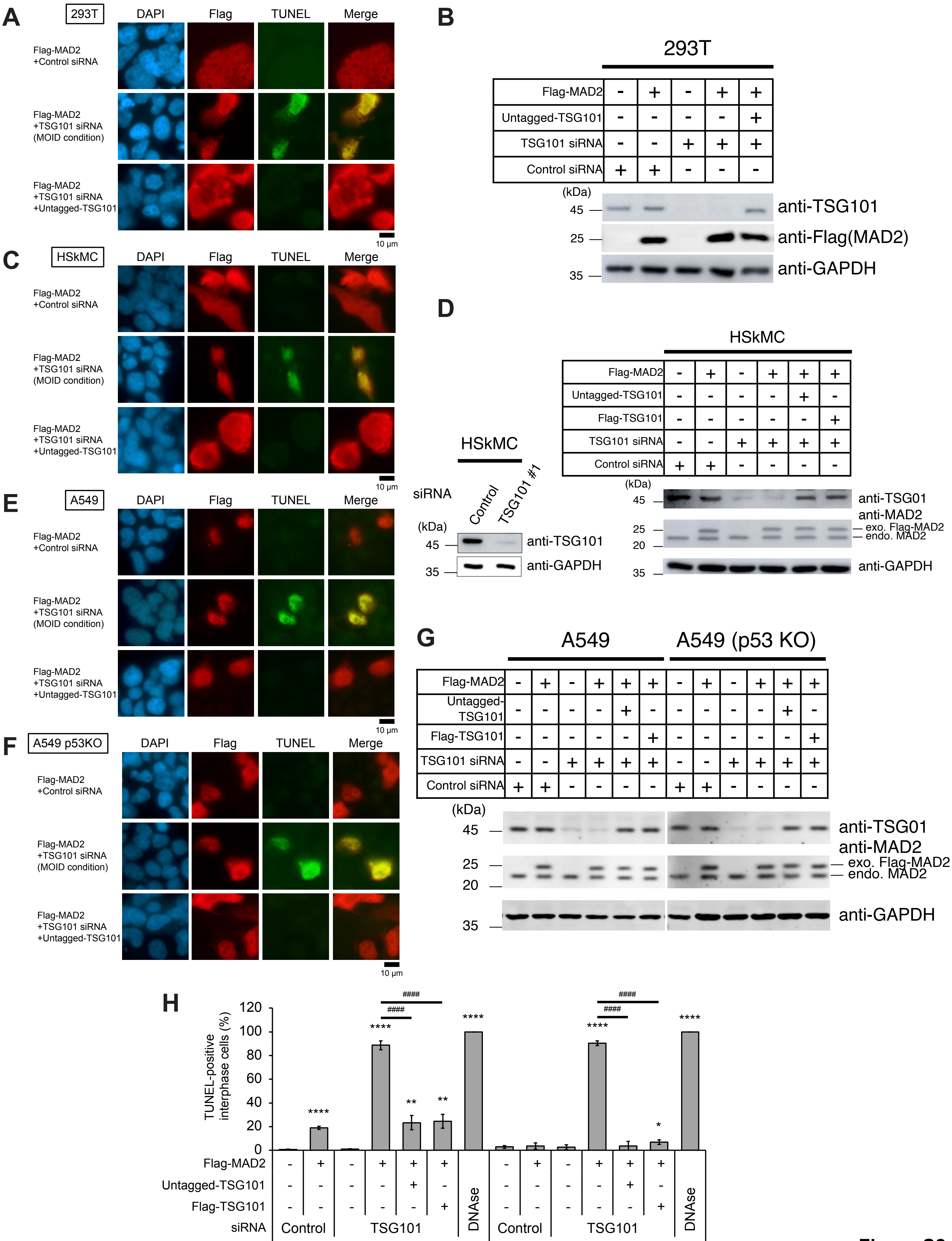

A
293T
DAPI
Flag
TUNEL
Merge
Flag-MAD2 +Control siRNA
Flag-MAD2 +TSG101 siRNA (MOID condition)
Flag-MAD2 +TSG101 siRNA +Untagged-TSG101
10 μm
B
293T
Flag-MAD2
Untagged-TSG101
TSG101 siRNA
Control siRNA
(kDa)
anti-TSG101
anti-Flag(MAD2)
anti-GAPDH
C
HSkMC
D
HSkMC
siRNA
Control
TSG101 #1
anti-TSG101
anti-GAPDH
Flag-TSG101
anti-TSG01
anti-MAD2
exo. Flag-MAD2
endo. MAD2
E
A549
F
A549 p53KO
G
A549
A549 (p53 KO)
H
TUNEL-positive interphase cells (%)
Flag-MAD2
Untagged-TSG101
Flag-TSG101
siRNA
Control
TSG101
DNAse
293T
HSkMC

Figure S2

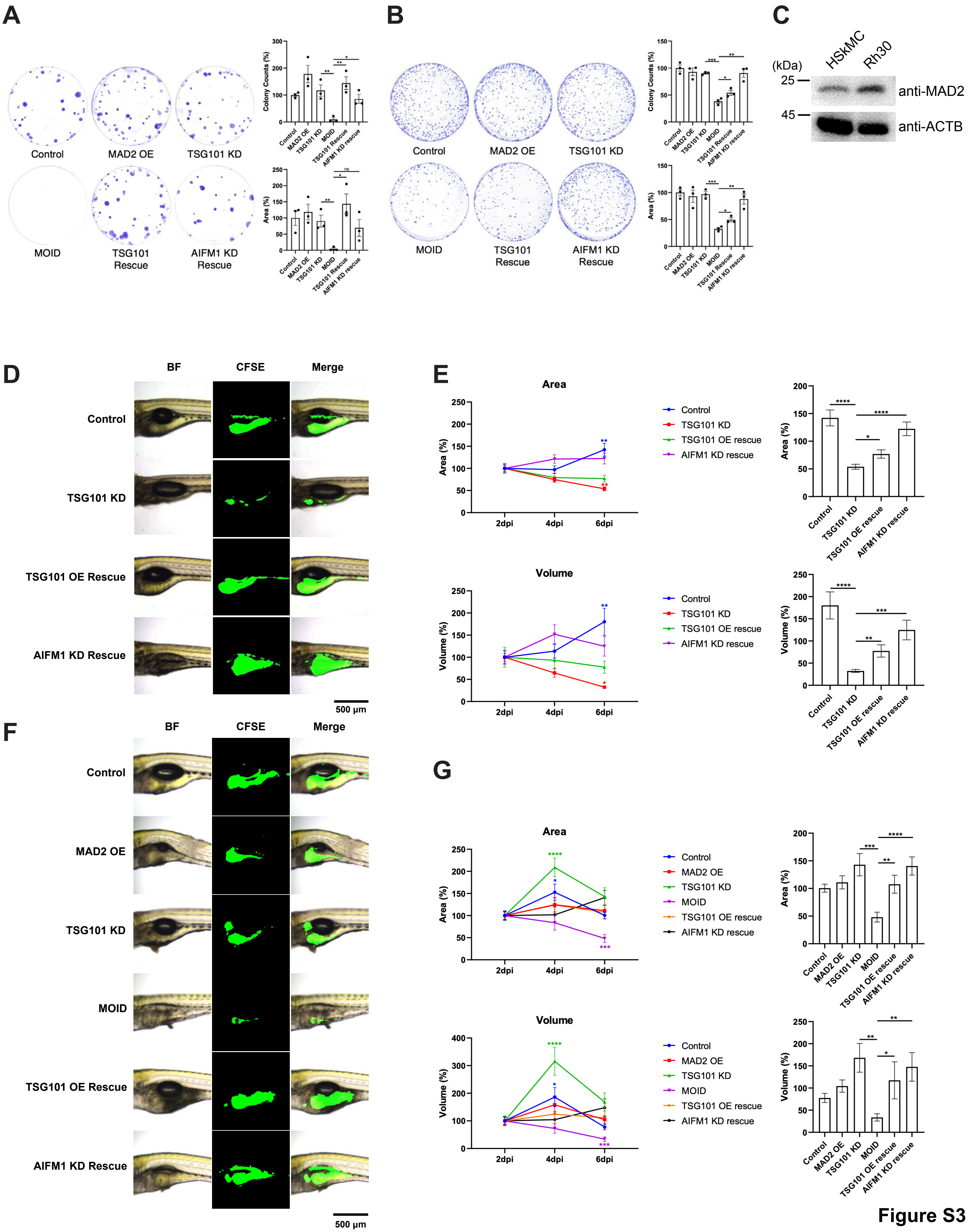
A
Control
MAD2 OE
TSG101 KD
MOID
TSG101
Rescue
AIFM1 KD
Rescue
Colony Counts (%)
Area (%)
Control
MAD2 OE
TSG101 KD
MOID
TSG101 Rescue
AIFM1 KD rescue
B
Control
MAD2 OE
TSG101 KD
MOID
TSG101
Rescue
AIFM1 KD
Rescue
Colony Counts (%)
Area (%)
C
HSkMC
Rh30
(kDa)
25
45
anti-MAD2
anti-ACTB
D
BF
CFSE
Merge
Control
TSG101 KD
TSG101 OE Rescue
AIFM1 KD Rescue
500 μm
E
Area
Area (%)
2dpi
4dpi
6dpi
Control
TSG101 KD
TSG101 OE rescue
AIFM1 KD rescue
Volume
Volume (%)
F
BF
CFSE
Merge
Control
MAD2 OE
TSG101 KD
MOID
TSG101 OE Rescue
AIFM1 KD Rescue
500 μm
G
Area
Area (%)
2dpi
4dpi
6dpi
Control
MAD2 OE
TSG101 KD
MOID
TSG101 OE rescue
AIFM1 KD rescue
Volume
Volume (%)

Figure S3

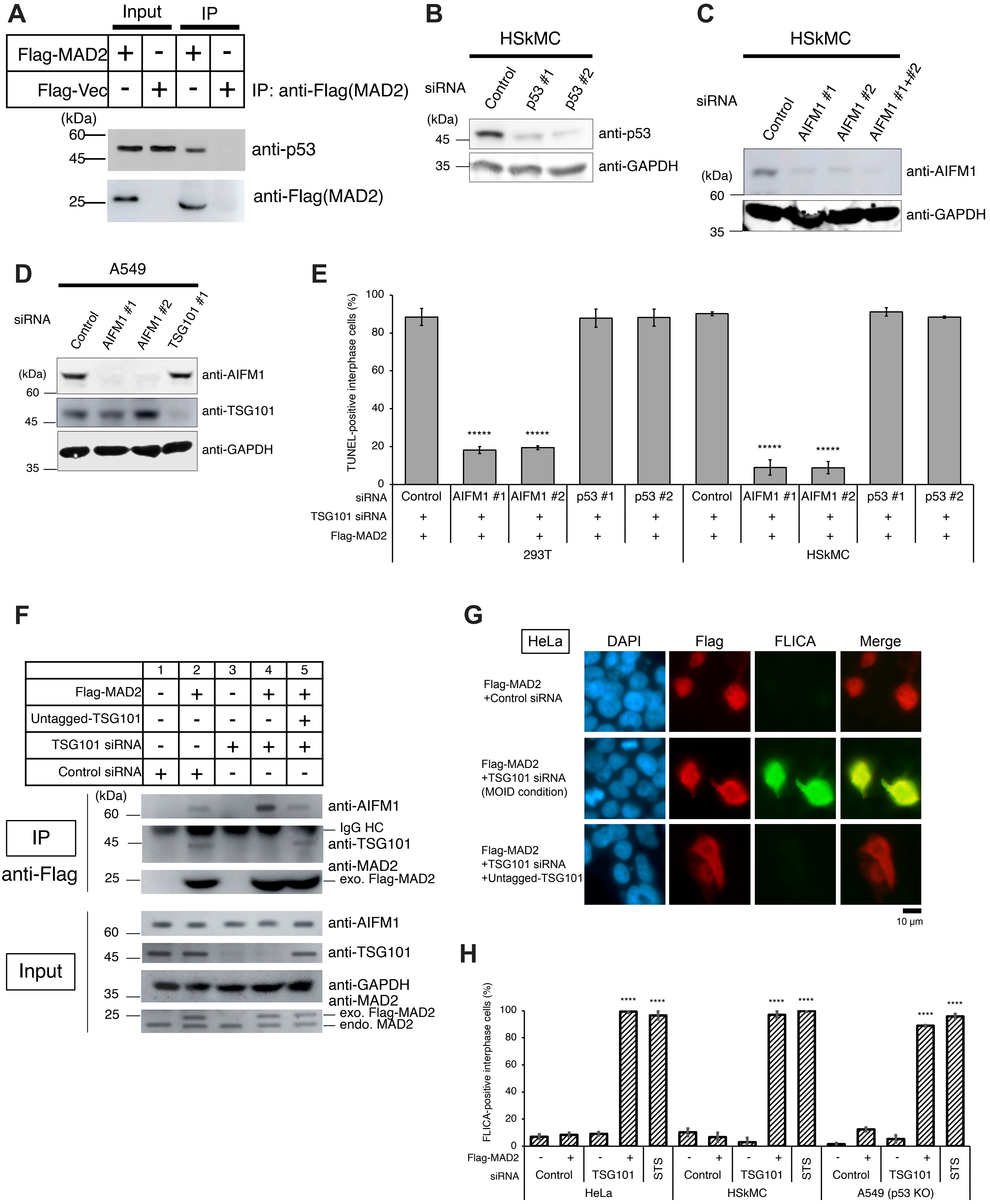
A
Input
IP
Flag-MAD2
Flag-Vec
IP: anti-Flag(MAD2)
(kDa)
60
45
25
anti-p53
anti-Flag(MAD2)
B
HSkMC
siRNA
Control
p53 #1
p53 #2
anti-p53
anti-GAPDH
C
HSkMC
siRNA
Control
AIFM1 #1
AIFM1 #2
AIFM1 #1+#2
anti-AIFM1
anti-GAPDH
D
A549
siRNA
Control
AIFM1 #1
AIFM1 #2
TSG101 #1
anti-AIFM1
anti-TSG101
anti-GAPDH
E
TUNEL-positive interphase cells (%)
siRNA
TSG101 siRNA
Flag-MAD2
293T
HSkMC
F
Flag-MAD2
Untagged-TSG101
TSG101 siRNA
Control siRNA
IP
anti-Flag
IgG HC
anti-TSG101
anti-MAD2
exo. Flag-MAD2
Input
anti-AIFM1
anti-GAPDH
endo. MAD2
G
HeLa
DAPI
Flag
FLICA
Merge
Flag-MAD2
+Control siRNA
Flag-MAD2
+TSG101 siRNA
(MOID condition)
Flag-MAD2
+TSG101 siRNA
+Untagged-TSG101
10 μm
H
FLICA-positive interphase cells (%)
Flag-MAD2
siRNA
Control
TSG101
STS
HeLa
HSkMC
A549 (p53 KO)

Figure S4

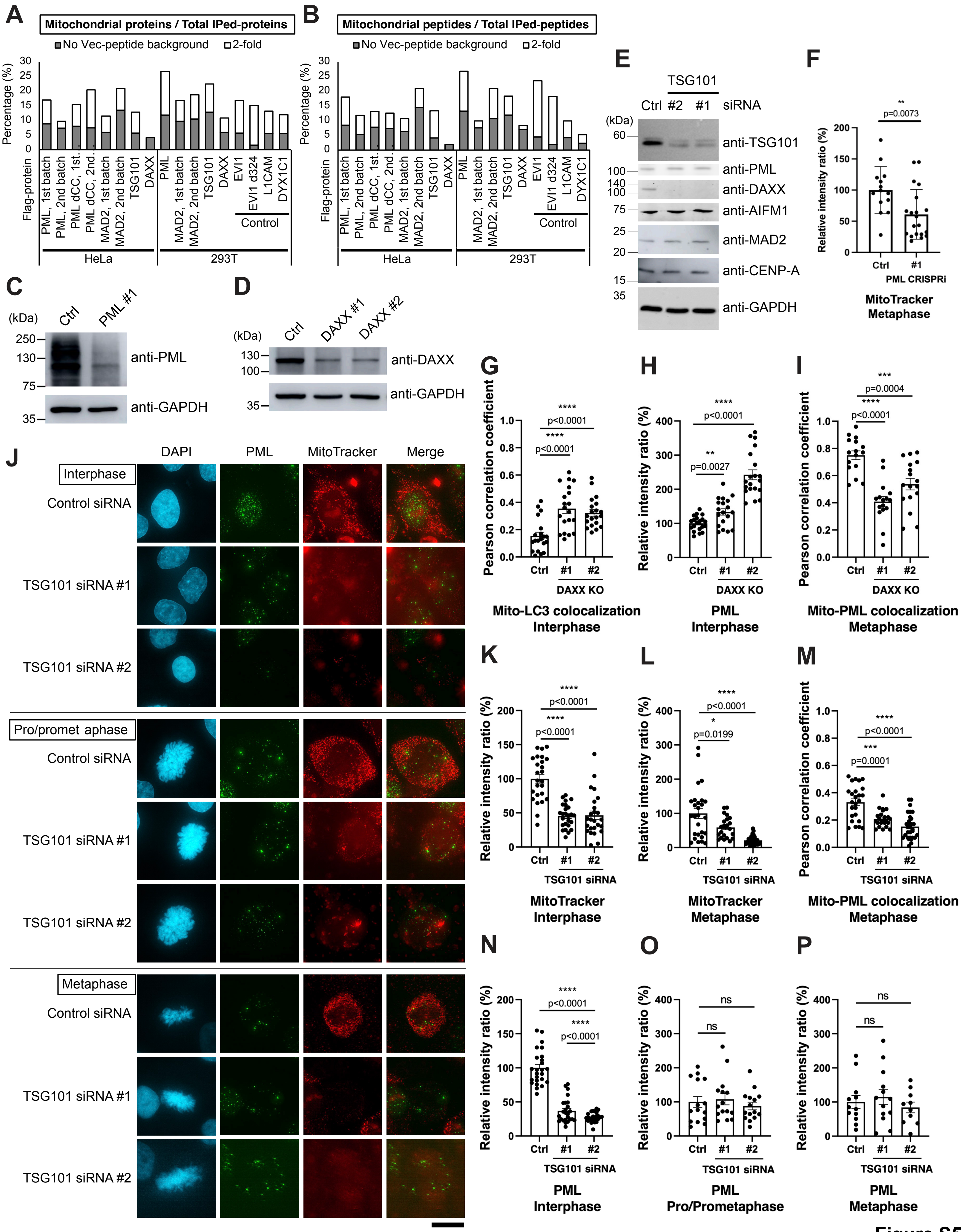

A
Mitochondrial proteins / Total IPed-proteins
No Vec-peptide background
2-fold
Percentage (%)
Flag-protein
HeLa
293T
Control
B
Mitochondrial peptides / Total IPed-peptides
C
anti-PML
anti-GAPDH
D
anti-DAXX
E
TSG101
siRNA
anti-TSG101
anti-PML
anti-DAXX
anti-AIFM1
anti-MAD2
anti-CENP-A
F
p=0.0073
PML CRISPRi
MitoTracker Metaphase
G
Mito-LC3 colocalization Interphase
H
PML Interphase
I
Mito-PML colocalization Metaphase
J
Interphase
Pro/promet aphase
Metaphase
DAPI
PML
MitoTracker
Merge
Control siRNA
TSG101 siRNA #1
TSG101 siRNA #2
10 µm
K
MitoTracker Interphase
L
MitoTracker Metaphase
M
Mito-PML colocalization Metaphase
N
PML Interphase
O
PML Pro/Prometaphase
P
PML Metaphase

Figure S5

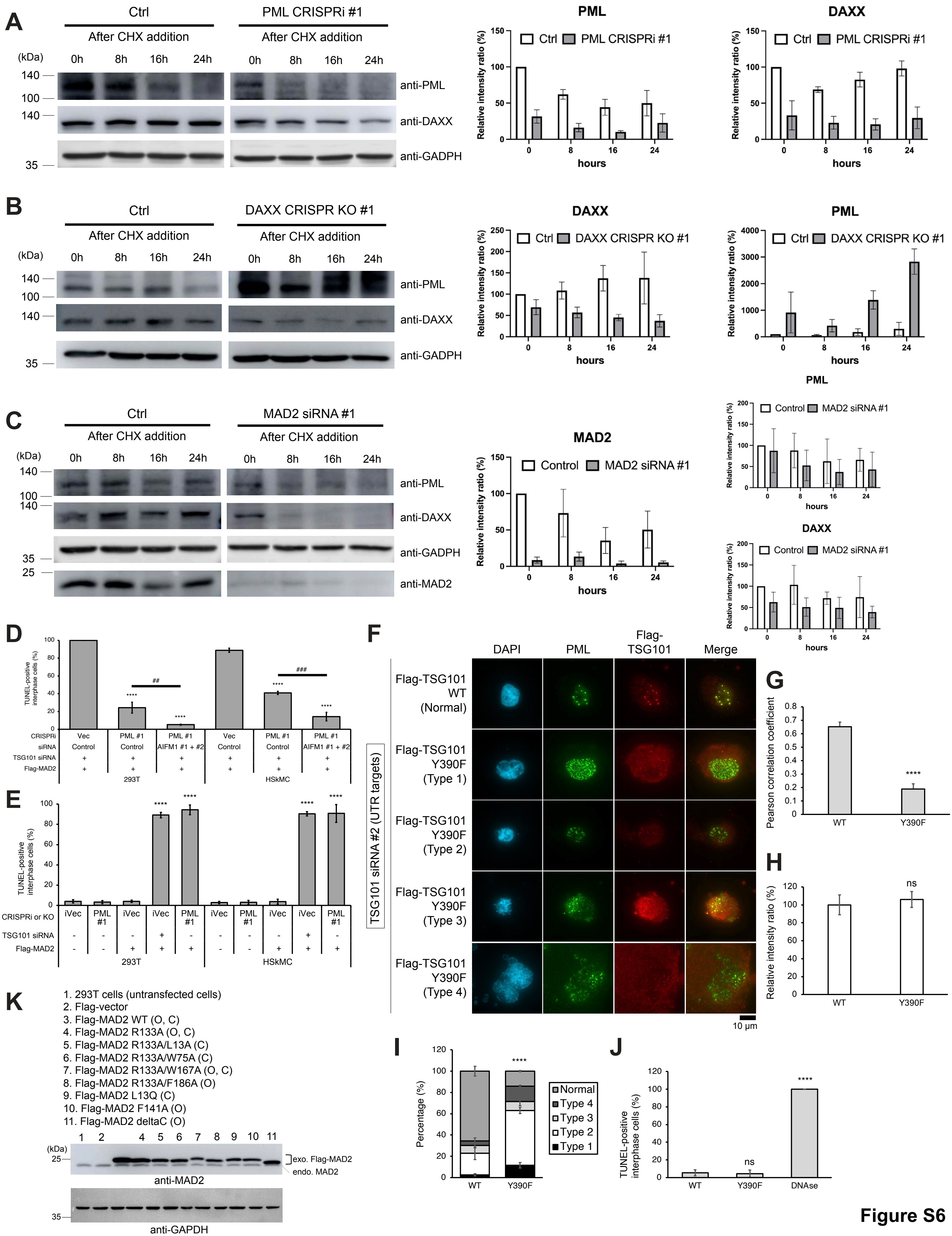
A
Ctrl
PML CRISPRi #1
After CHX addition
(kDa)
0h 8h 16h 24h
140
100
140
35
anti-PML
anti-DAXX
anti-GADPH
PML
DAXX
Ctrl
PML CRISPRi #1
Relative intensity ratio (%)
hours
B
DAXX CRISPR KO #1
C
MAD2 siRNA #1
Control
anti-MAD2
25
MAD2
D
TUNEL-positive interphase cells (%)
CRISPRi
siRNA
TSG101 siRNA
Flag-MAD2
Vec
PML #1
Control
AIFM1 #1 + #2
293T
HSkMC
##
###
****
E
CRISPRi or KO
iVec
F
DAPI
PML
Flag-TSG101
Merge
Flag-TSG101 WT (Normal)
Flag-TSG101 Y390F (Type 1)
Flag-TSG101 Y390F (Type 2)
Flag-TSG101 Y390F (Type 3)
Flag-TSG101 Y390F (Type 4)
TSG101 siRNA #2 (UTR targets)
10 µm
G
Pearson correlation coefficient
WT
Y390F
H
ns
I
Percentage (%)
Normal
Type 4
Type 3
Type 2
Type 1
J
DNAse
K
1. 293T cells (untransfected cells)
2. Flag-vector
3. Flag-MAD2 WT (O, C)
4. Flag-MAD2 R133A (O, C)
5. Flag-MAD2 R133A/L13A (C)
6. Flag-MAD2 R133A/W75A (C)
7. Flag-MAD2 R133A/W167A (O, C)
8. Flag-MAD2 R133A/F186A (O)
9. Flag-MAD2 L13Q (C)
10. Flag-MAD2 F141A (O)
11. Flag-MAD2 deltaC (O)
exo. Flag-MAD2
endo. MAD2
anti-MAD2
anti-GAPDH

Figure S6

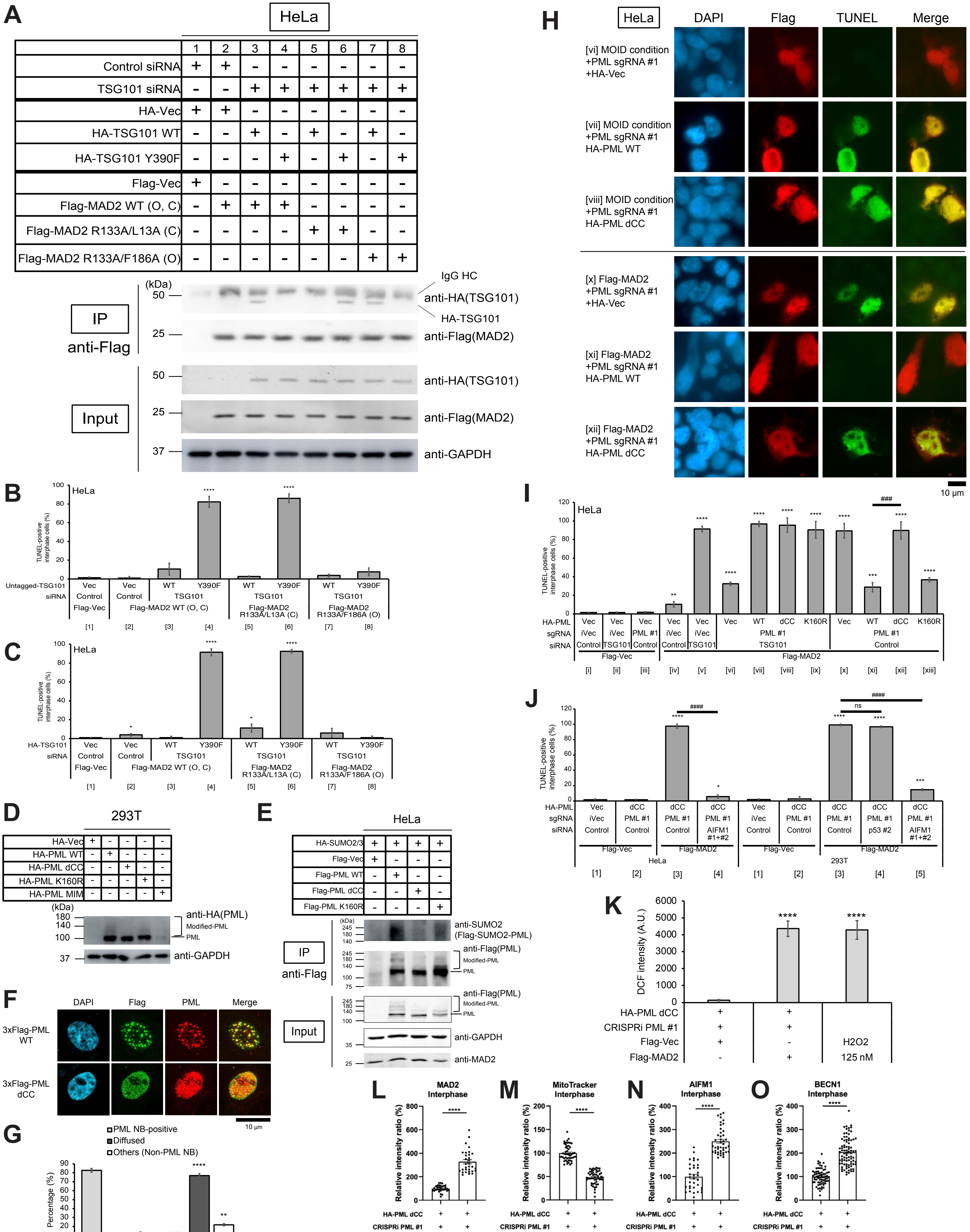

A
HeLa
Control siRNA
TSG101 siRNA
HA-Vec
HA-TSG101 WT
HA-TSG101 Y390F
Flag-Vec
Flag-MAD2 WT (O, C)
Flag-MAD2 R133A/L13A (C)
Flag-MAD2 R133A/F186A (O)
(kDa)
IgG HC
anti-HA(TSG101)
HA-TSG101
anti-Flag(MAD2)
anti-GAPDH
IP
anti-Flag
Input
B
HeLa
TUNEL-positive interphase cells (%)
Untagged-TSG101
siRNA
Vec
Control
Flag-Vec
WT
Y390F
TSG101
Flag-MAD2 WT (O, C)
Flag-MAD2 R133A/L13A (C)
Flag-MAD2 R133A/F186A (O)
C
HA-TSG101
D
293T
HA-Vec
HA-PML WT
HA-PML dCC
HA-PML K160R
HA-PML MIM
anti-HA(PML)
Modified-PML
PML
anti-GAPDH
E
HeLa
HA-SUMO2/3
Flag-Vec
Flag-PML WT
Flag-PML dCC
Flag-PML K160R
anti-SUMO2
(Flag-SUMO2-PML)
anti-Flag(PML)
Modified-PML
PML
anti-GAPDH
anti-MAD2
IP
anti-Flag
Input
F
DAPI
Flag
PML
Merge
3xFlag-PML WT
3xFlag-PML dCC
10 µm
G
PML NB-positive
Diffused
Others (Non-PML NB)
Percentage (%)
3xFlag-PML WT
3xFlag-PML dCC
H
HeLa
DAPI
Flag
TUNEL
Merge
[vi] MOID condition +PML sgRNA #1 +HA-Vec
[vii] MOID condition +PML sgRNA #1 HA-PML WT
[viii] MOID condition +PML sgRNA #1 HA-PML dCC
[x] Flag-MAD2 +PML sgRNA #1 +HA-Vec
[xi] Flag-MAD2 +PML sgRNA #1 HA-PML WT
[xii] Flag-MAD2 +PML sgRNA #1 HA-PML dCC
10 µm
I
HeLa
TUNEL-positive interphase cells (%)
HA-PML
sgRNA
siRNA
Vec
WT
dCC
K160R
iVec
PML #1
Control
TSG101
Flag-Vec
Flag-MAD2
J
TUNEL-positive interphase cells (%)
HA-PML
sgRNA
siRNA
AIFM1 #1+#2
p53 #2
HeLa
293T
K
DCF intensity (A.U.)
HA-PML dCC
CRISPRi PML #1
Flag-Vec
Flag-MAD2
H2O2
125 nM
L
MAD2 Interphase
M
MitoTracker Interphase
N
AIFM1 Interphase
O
BECN1 Interphase
Relative intensity ratio (%)
HA-PML dCC
CRISPRi PML #1
Flag-Vec
Flag-MAD2

Figure S7

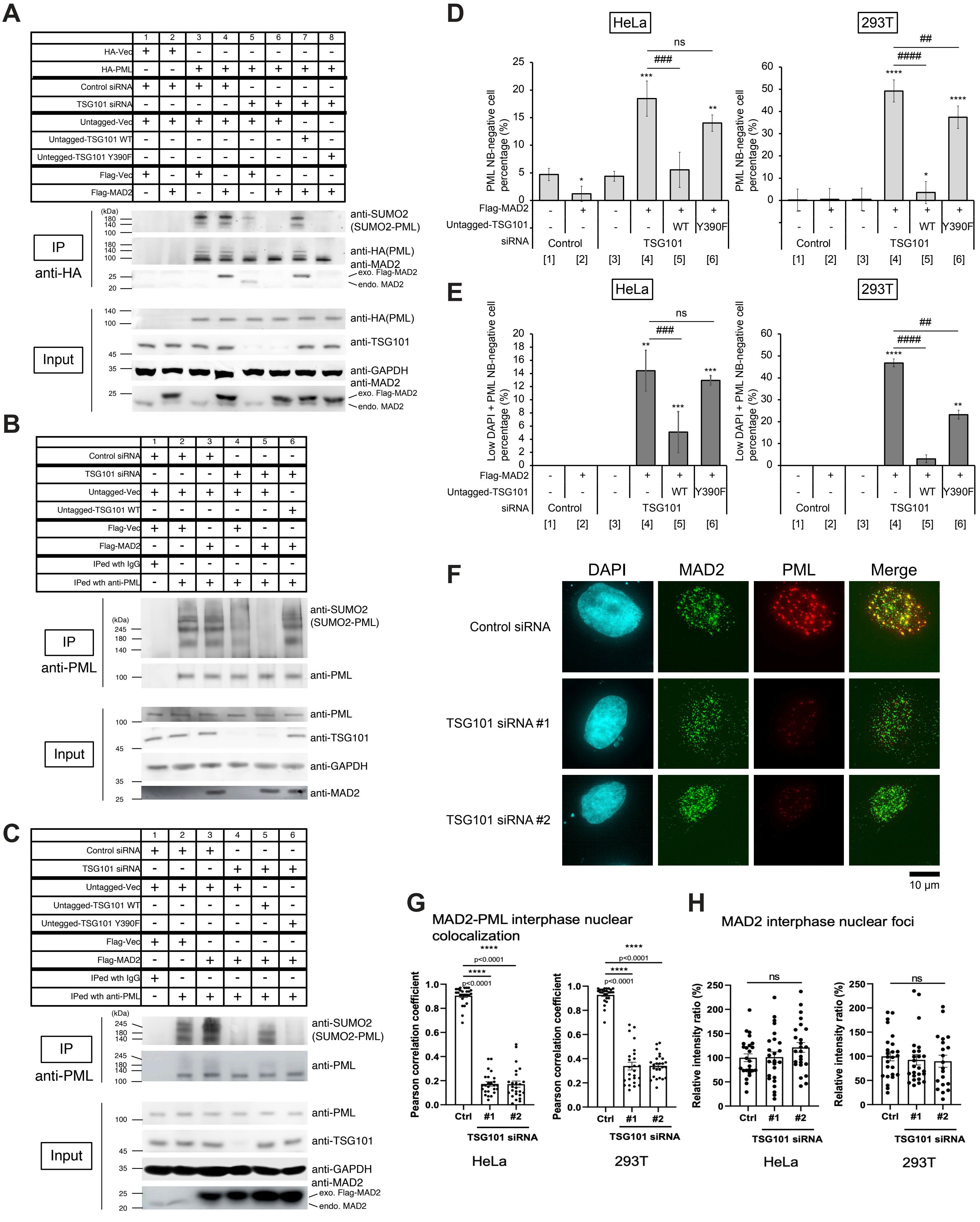
A
1 2 3 4 5 6 7 8
HA-Vec
HA-PML
Control siRNA
TSG101 siRNA
Untagged-Vec
Untagged-TSG101 WT
Untegged-TSG101 Y390F
Flag-Vec
Flag-MAD2
IP
anti-HA
(kDa)
anti-SUMO2
(SUMO2-PML)
anti-HA(PML)
anti-MAD2
exo. Flag-MAD2
endo. MAD2
Input
anti-HA(PML)
anti-TSG101
anti-GAPDH
anti-MAD2
exo. Flag-MAD2
endo. MAD2
B
1 2 3 4 5 6
Control siRNA
TSG101 siRNA
Untagged-Vec
Untagged-TSG101 WT
Flag-Vec
Flag-MAD2
IPed wth IgG
IPed wth anti-PML
IP
anti-PML
(kDa)
anti-SUMO2
(SUMO2-PML)
anti-PML
Input
anti-PML
anti-TSG101
anti-GAPDH
anti-MAD2
C
1 2 3 4 5 6
Control siRNA
TSG101 siRNA
Untagged-Vec
Untagged-TSG101 WT
Untegged-TSG101 Y390F
Flag-Vec
Flag-MAD2
IPed wth IgG
IPed wth anti-PML
IP
anti-PML
(kDa)
anti-SUMO2
(SUMO2-PML)
anti-PML
Input
anti-PML
anti-TSG101
anti-GAPDH
anti-MAD2
exo. Flag-MAD2
endo. MAD2
D
HeLa
293T
PML NB-negative cell percentage (%)
ns
###
####
##
Flag-MAD2
Untagged-TSG101
siRNA
Control
TSG101
WT
Y390F
[1] [2] [3] [4] [5] [6]
E
HeLa
293T
Low DAPI + PML NB-negative cell percentage (%)
ns
###
####
##
Flag-MAD2
Untagged-TSG101
siRNA
Control
TSG101
WT
Y390F
[1] [2] [3] [4] [5] [6]
F
DAPI
MAD2
PML
Merge
Control siRNA
TSG101 siRNA #1
TSG101 siRNA #2
10 μm
G
MAD2-PML interphase nuclear colocalization
Pearson correlation coefficient
p<0.0001
Ctrl
#1
#2
TSG101 siRNA
HeLa
293T
H
MAD2 interphase nuclear foci
Relative intensity ratio (%)
ns
Ctrl
#1
#2
TSG101 siRNA
HeLa
293T

Figure S8

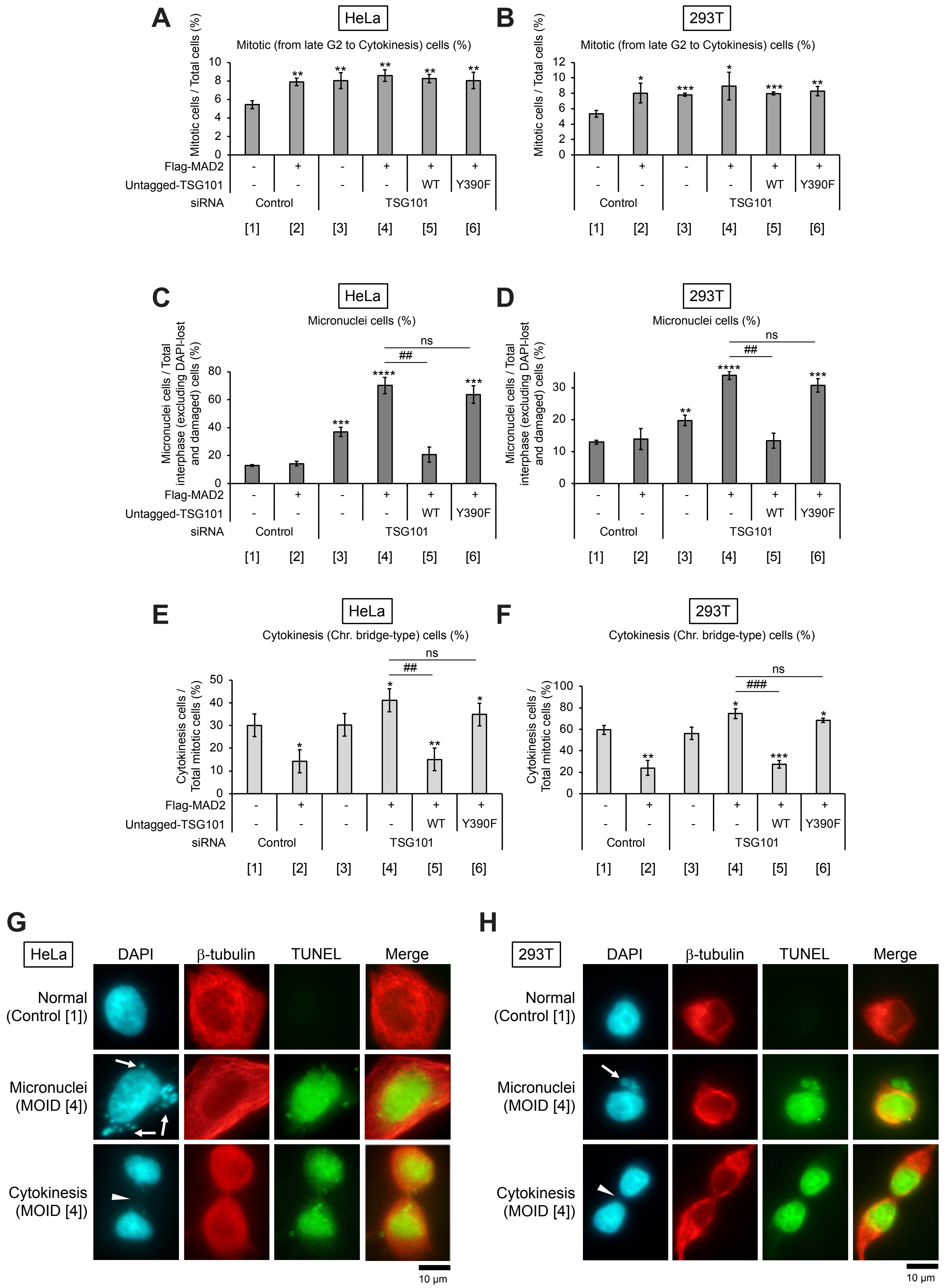
A
HeLa
Mitotic (from late G2 to Cytokinesis) cells (%)
Mitotic cells / Total cells (%)
Flag-MAD2
Untagged-TSG101
siRNA
Control
TSG101
WT
Y390F
[1] [2] [3] [4] [5] [6]
B
293T
Mitotic (from late G2 to Cytokinesis) cells (%)
Mitotic cells / Total cells (%)
C
HeLa
Micronuclei cells (%)
Micronuclei cells / Total interphase (excluding DAPI-lost and damaged) cells (%)
ns
##
D
293T
Micronuclei cells (%)
E
HeLa
Cytokinesis (Chr. bridge-type) cells (%)
Cytokinesis cells / Total mitotic cells (%)
F
293T
Cytokinesis (Chr. bridge-type) cells (%)
###
G
HeLa
DAPI
β-tubulin
TUNEL
Merge
Normal (Control [1])
Micronuclei (MOID [4])
Cytokinesis (MOID [4])
10 μm
H
293T

Figure S9

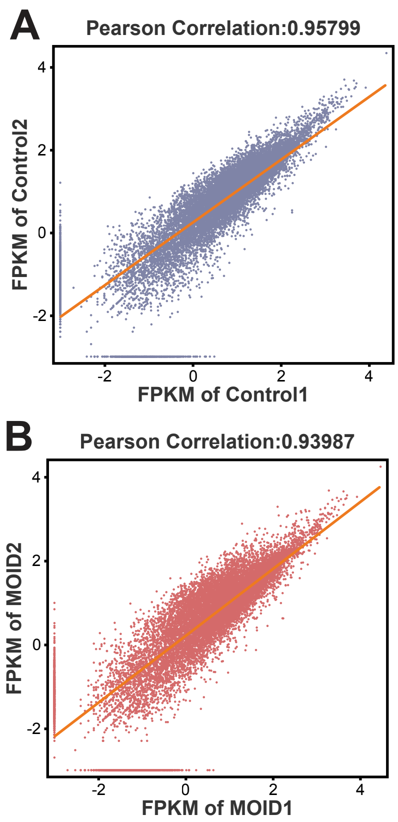

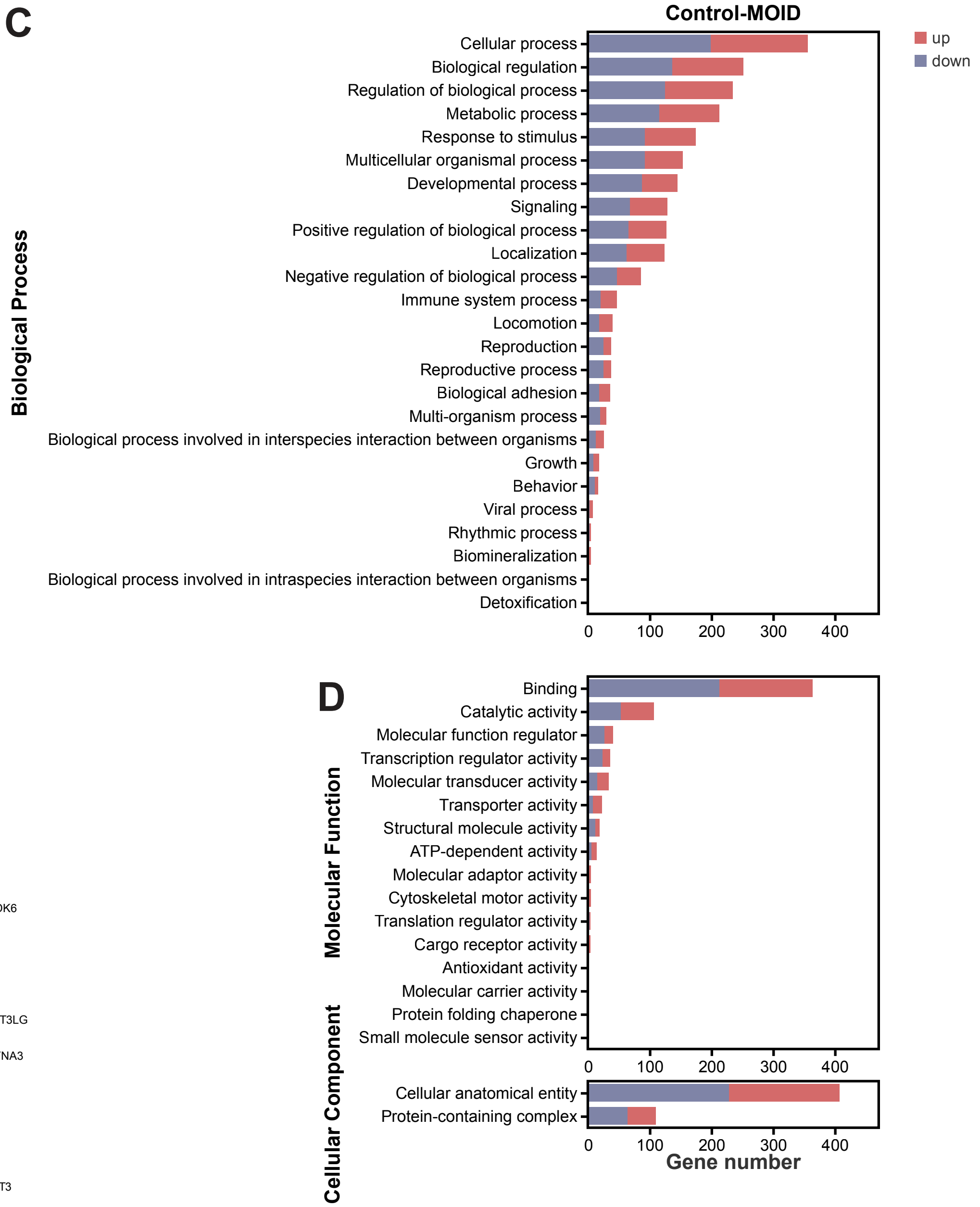

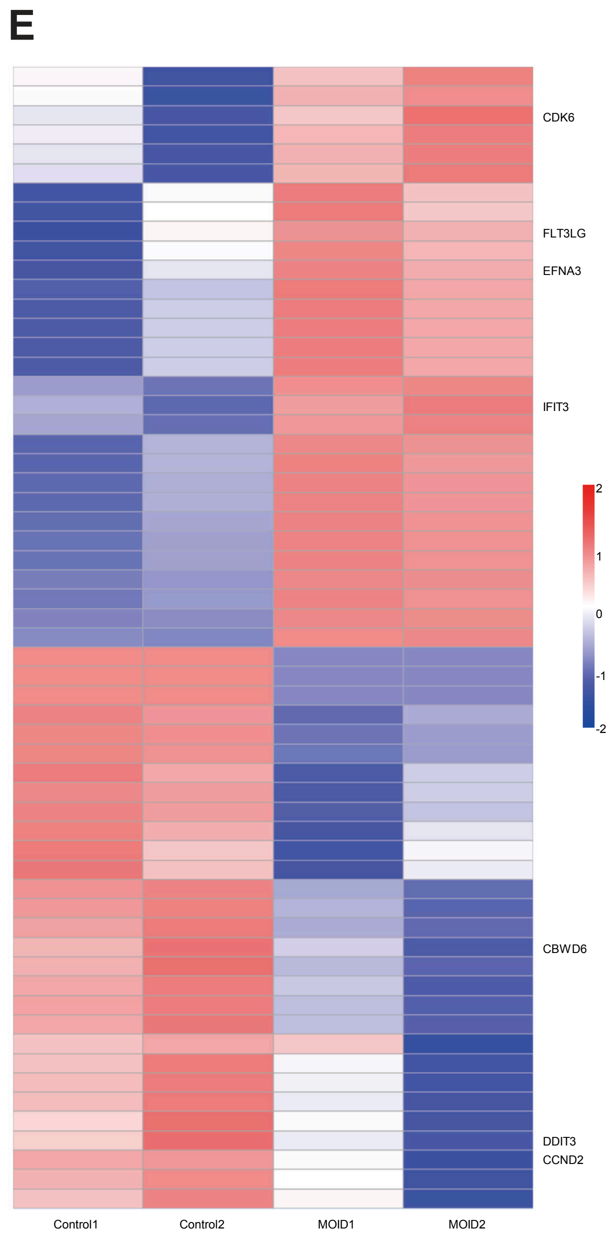

F

Gene symbol	Expression	Description
CCND2	Down	Cyclin D2, CDK kinase
DDIT3	Down	DNA damage inducible transcript 3
CBWD6	Down	ZNG1F, Zn regulated GTPase metalloprotein activator 1F
FLT3LG	Up	Fms related receptor tyrosine kinase 3 ligand
EFNA3	Up	Ephrin A3, anchored to the membrane
IFIT3	Up	Interferon induced protein with tetratricopeptide repeats 3
CDK6	Up	Cyclin dependent kinase 6

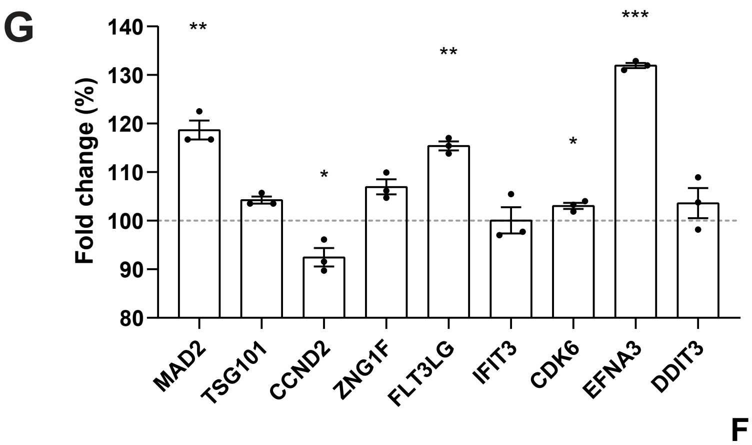

Figure S10
