# Supplementary material for: TSG101 depletion dysregulates mitochondria and PML NBs, triggering MAD2-overexpressing interphase cell death (MOID) through AIFM1-PML-DAXX pathway
Source: Cell Death Dis. 2024 Nov 17;15(11):838. doi: 10.1038/s41419-024-07229-w (PMC11570632; doi:10.1038/s41419-024-07229-w)
Supplement: Supplementary file 2 — Original western blots in Main Figures [file 41419_2024_7229_MOESM2_ESM.pdf]

Figure 1B

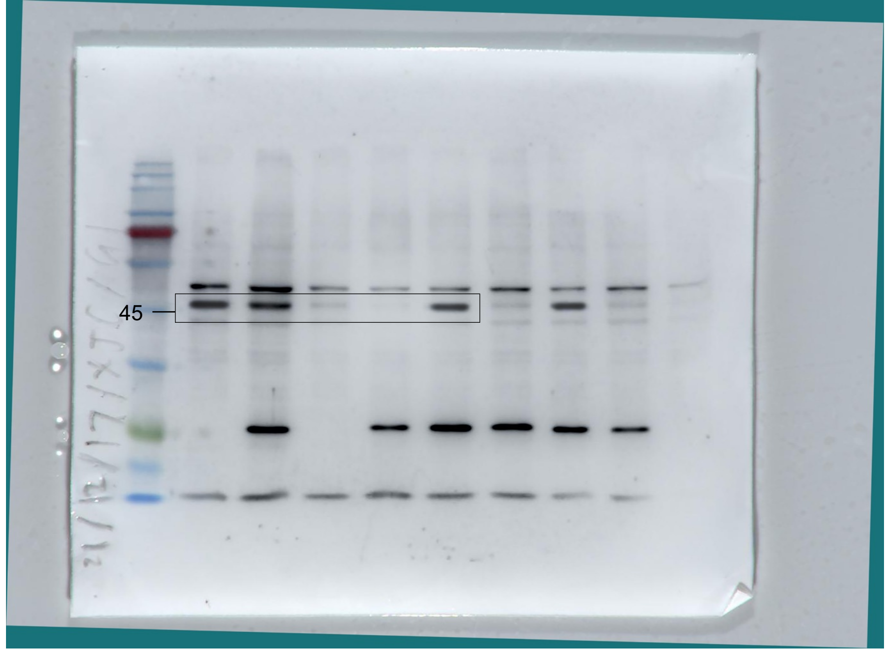

anti-TSG101

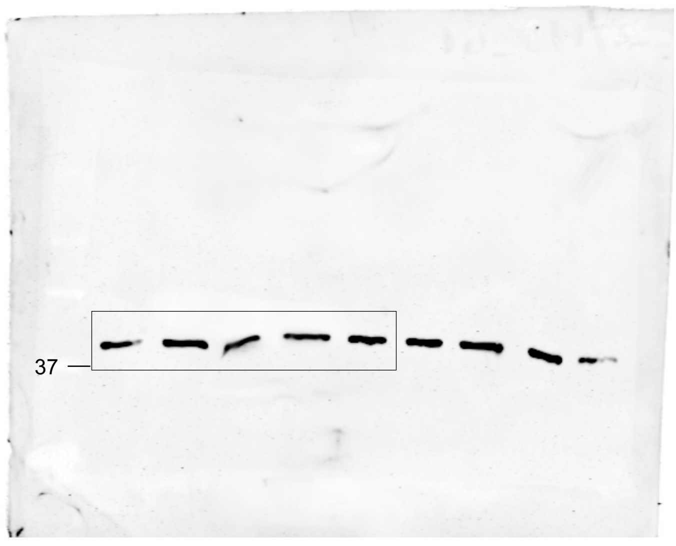

anti-GAPDH

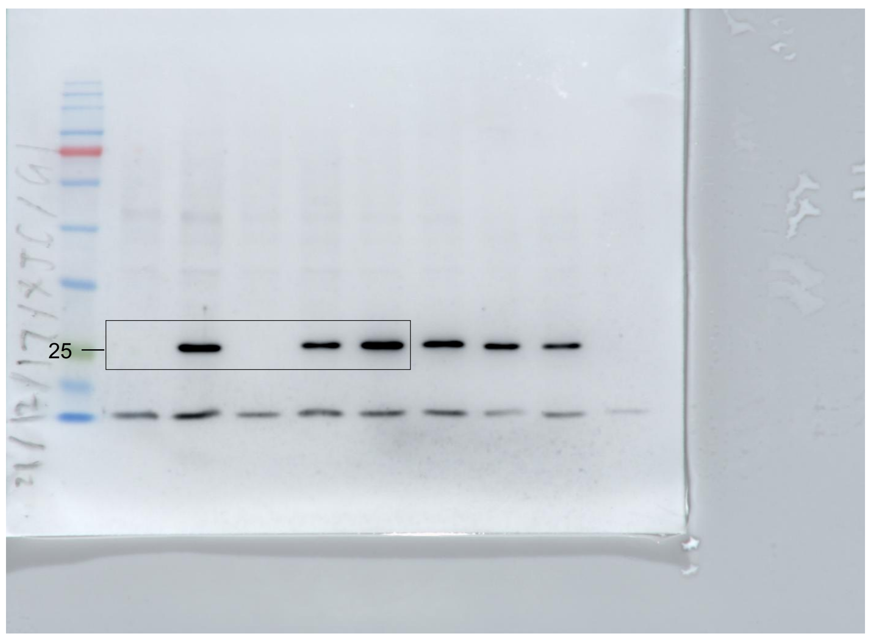

anti-Flag(MAD2)

Figure 2I
HeLa
Mc

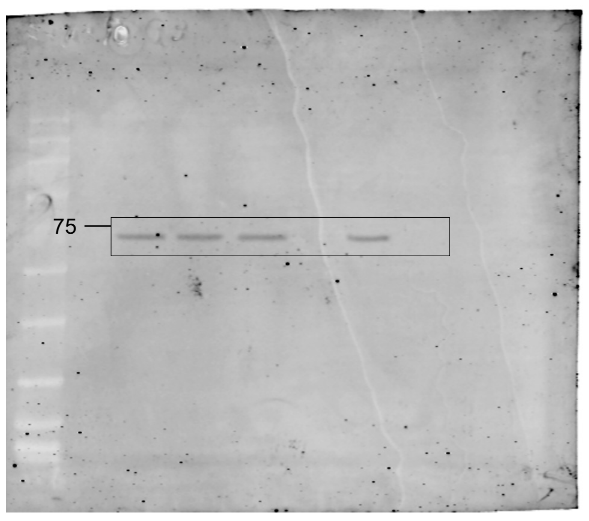

anti-AIFM1

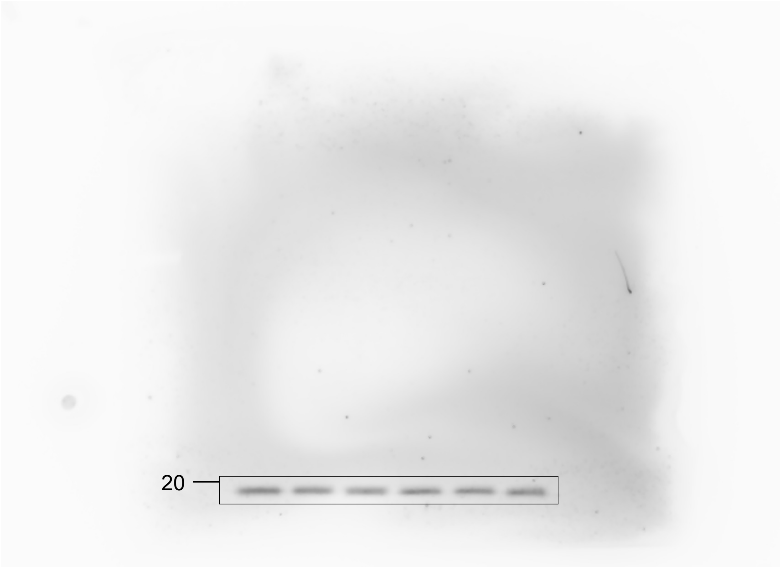

anti-TOM20

Figure 2I
HeLa
N

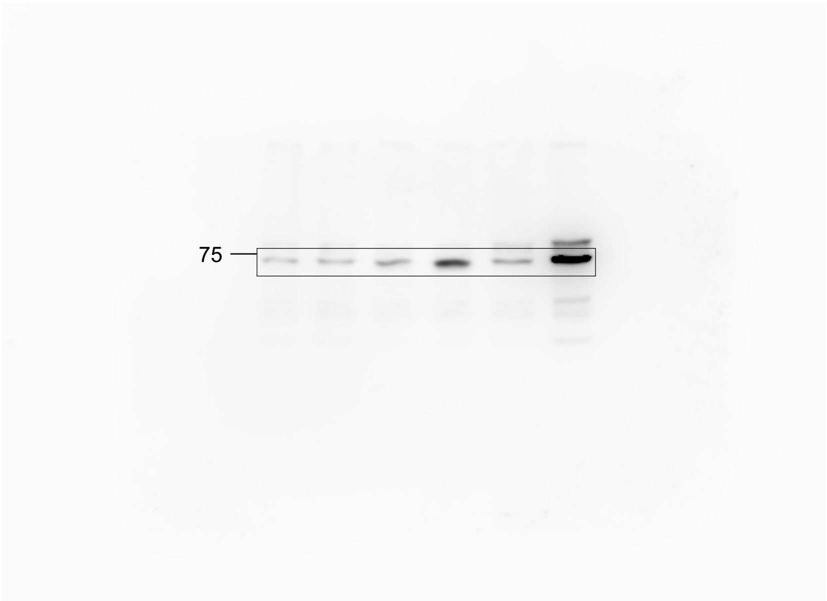

anti-AIFM1

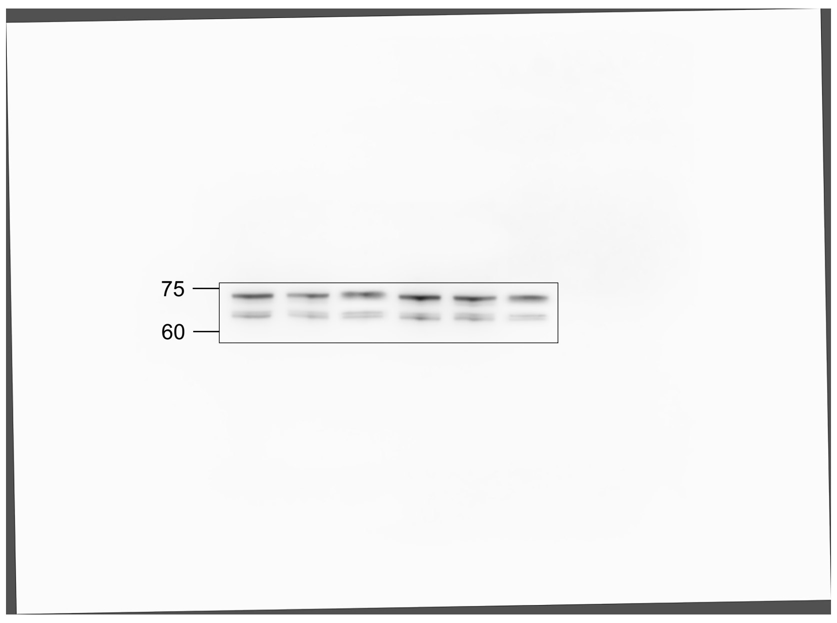

anti-Lamin A/C

Figure 2I
293T
Mc

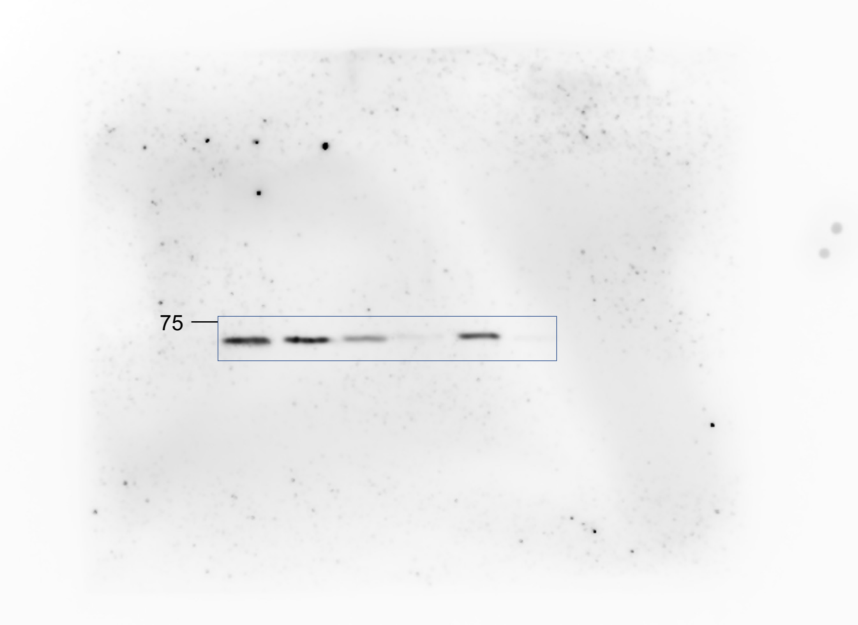

anti-AIFM1

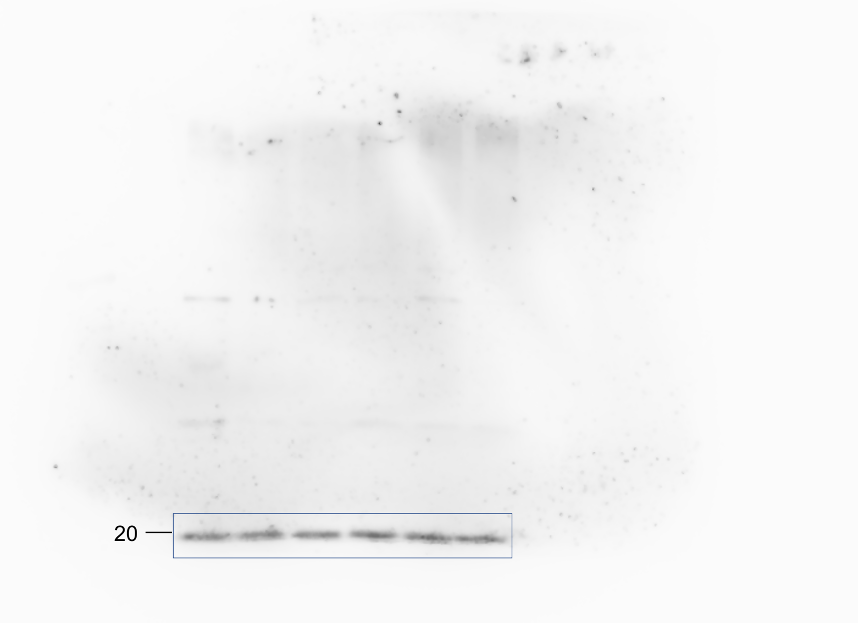

anti-TOM20

Figure 2I
293T
N

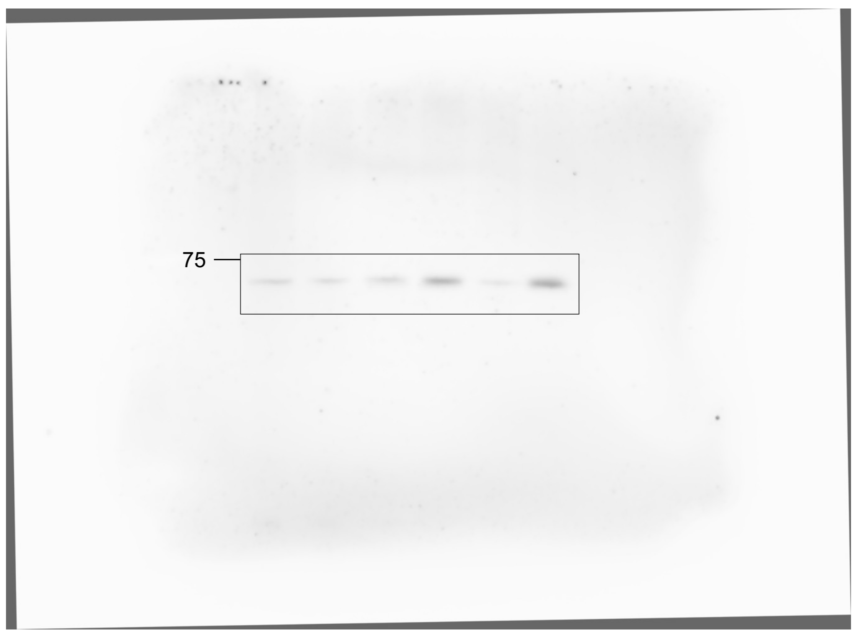

anti-AIFM1

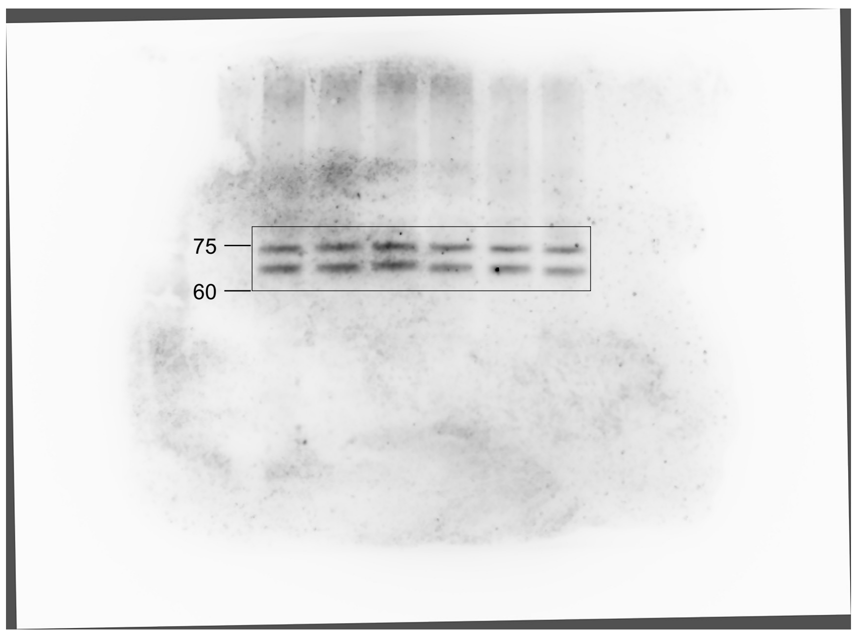

anti-Lamin A/C

Figure 3B

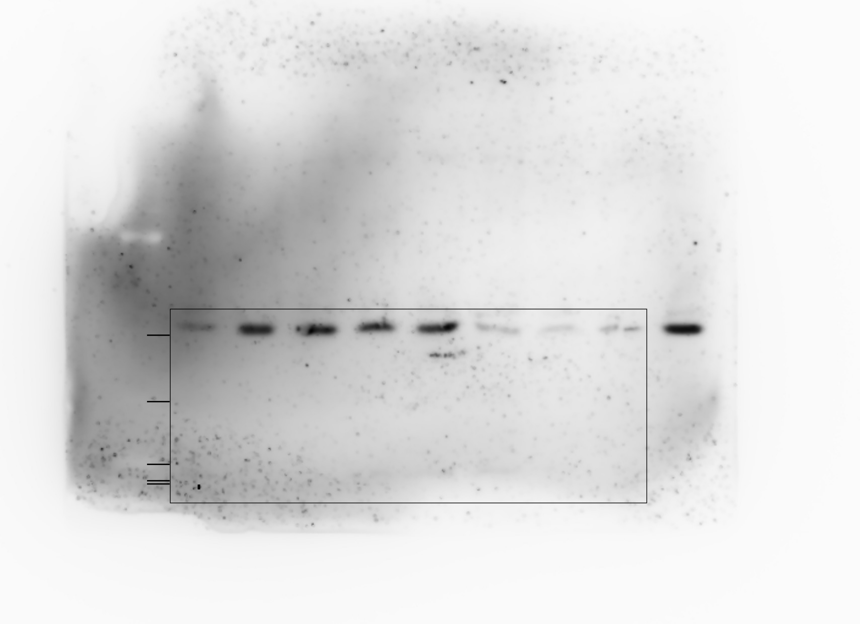

anti-TSG101

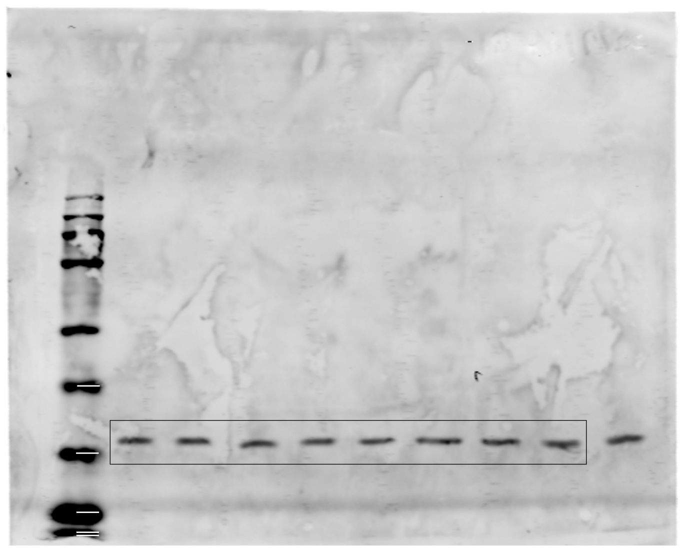

anti-GAPDH

Figure 3G
IP

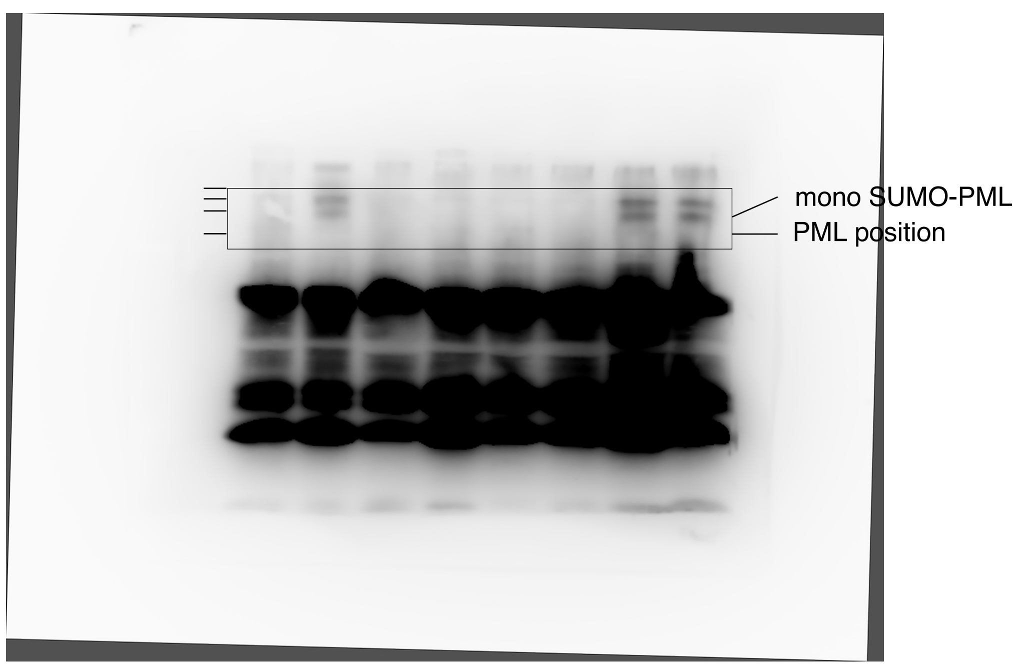

anti-SUMO2

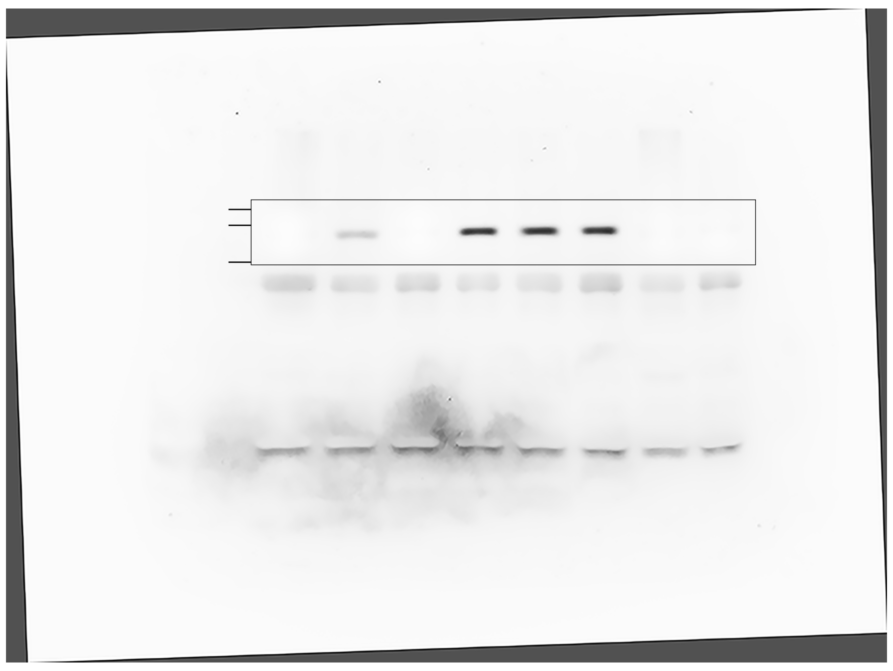

anti-AIFM1

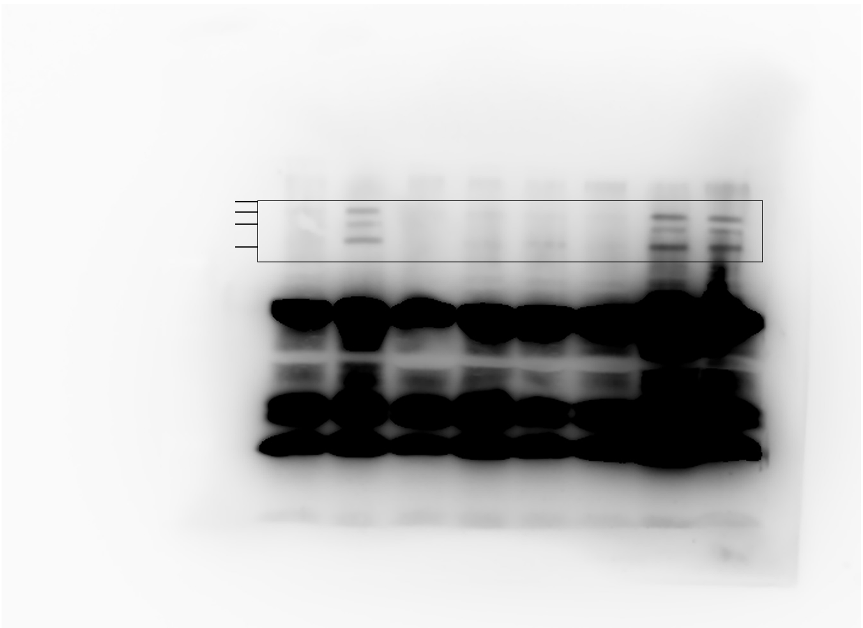

anti-PML

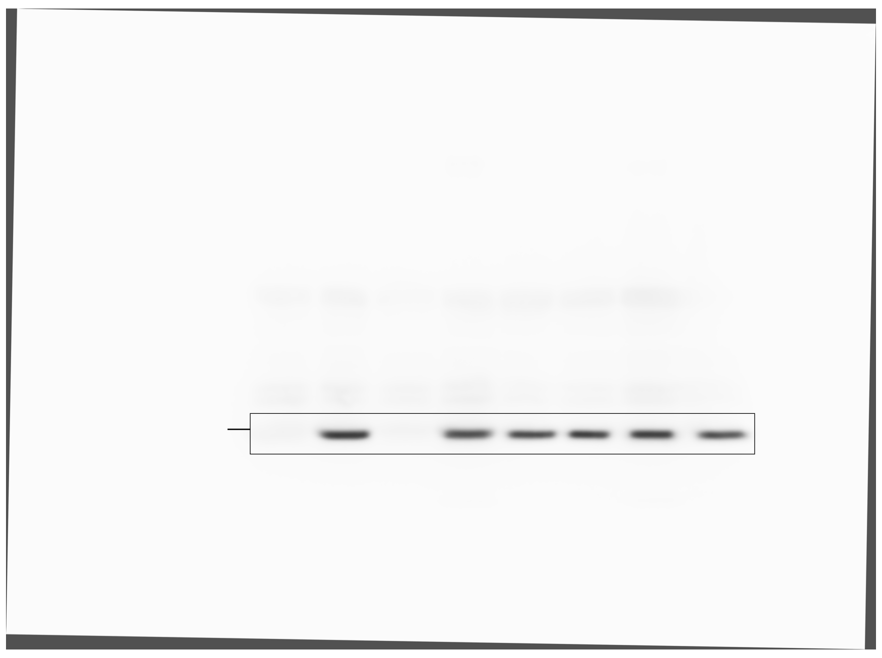

anti-MAD2

Figure 3G
Input

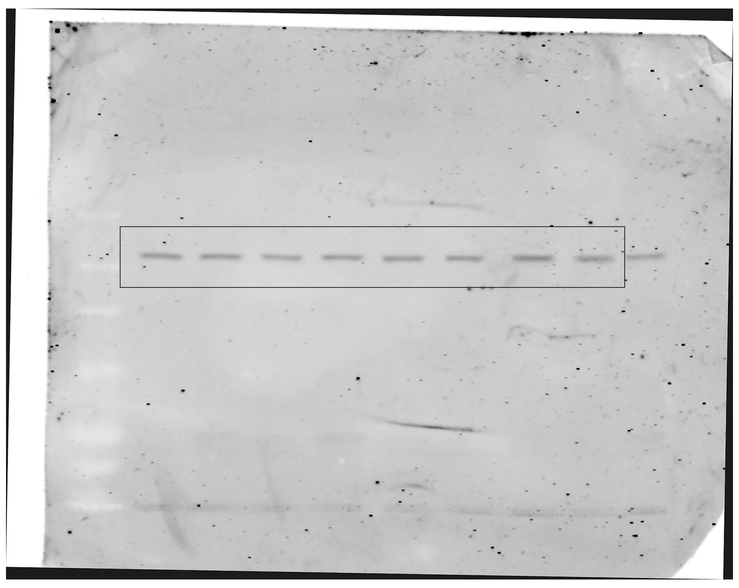

anti-AIFM1

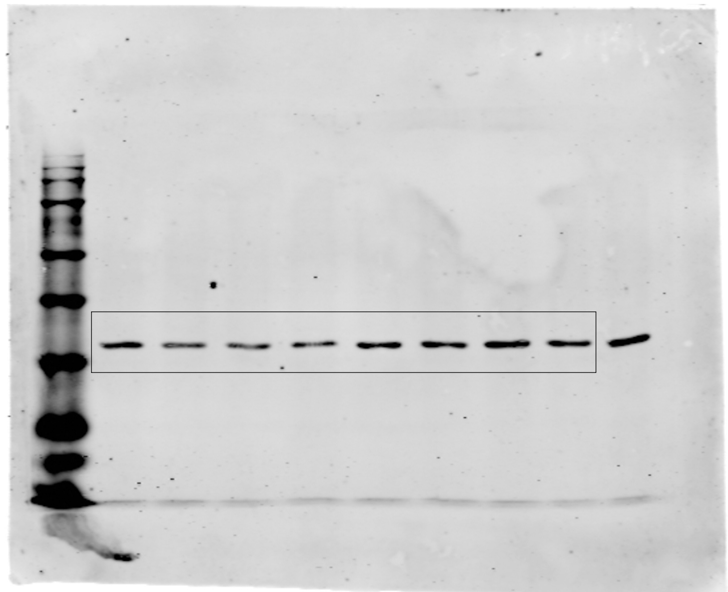

anti-GAPDH

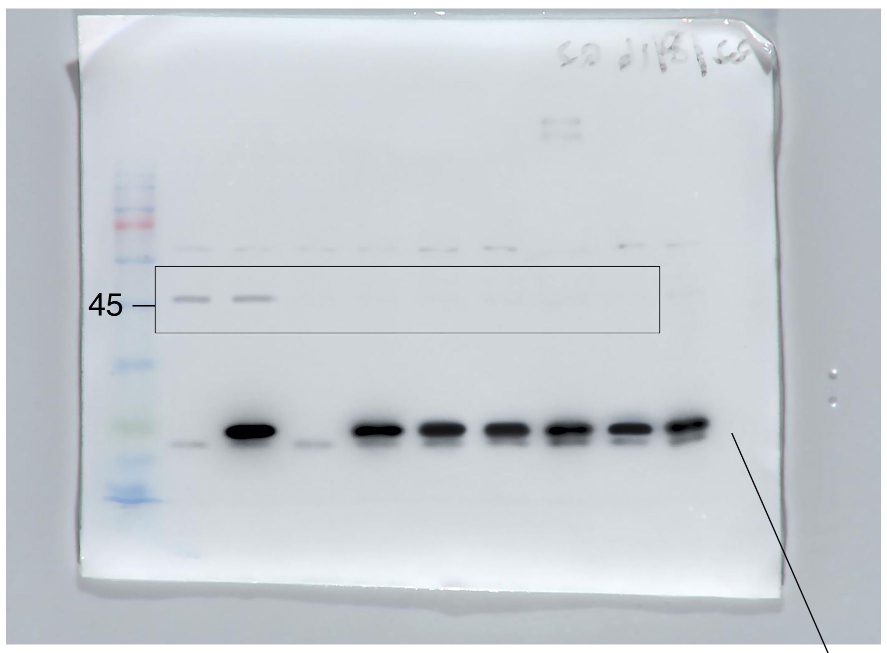

anti-TSG101

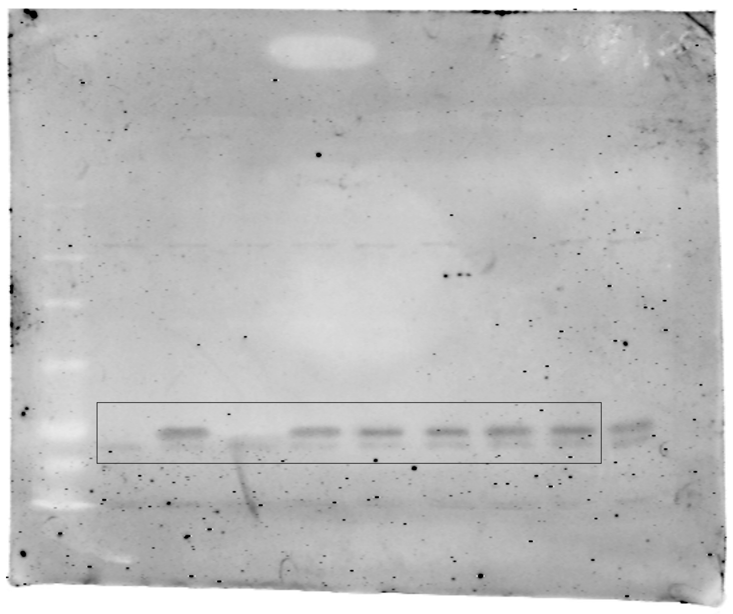

anti-MAD2

Previously blotted anti-MAD2 band

Figure 3H
IP

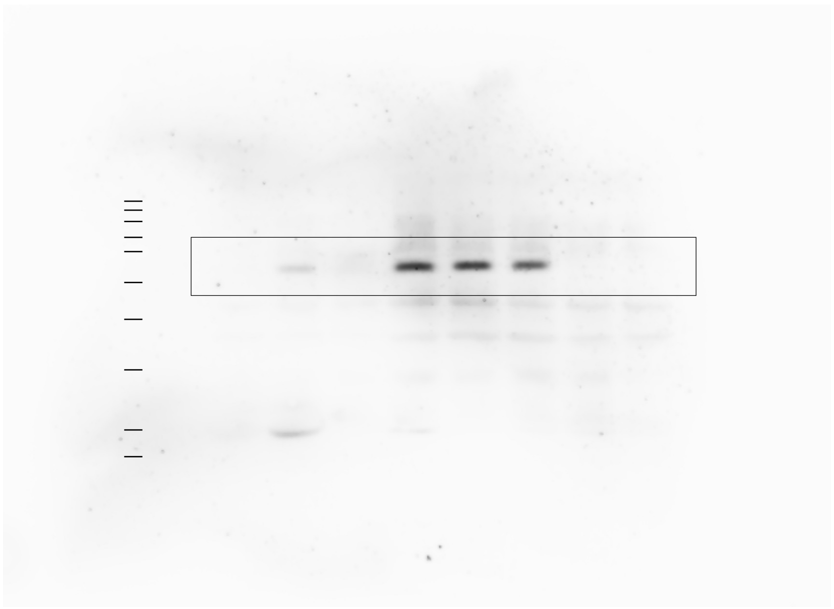

anti-AIFM1

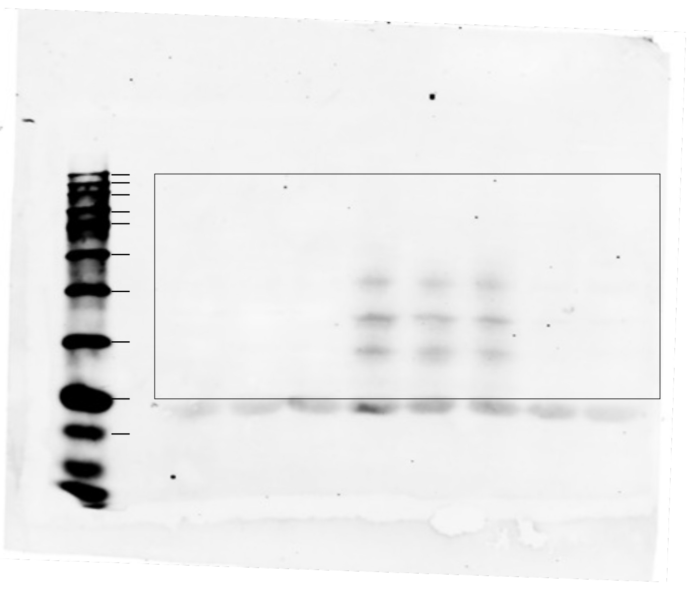

anti-SUMO2

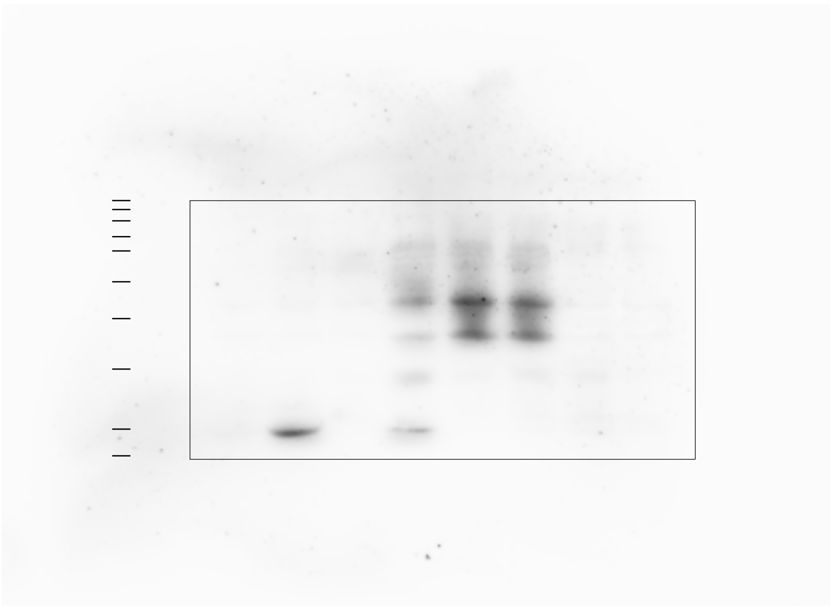

anti-MAD2

Figure 3H
Input

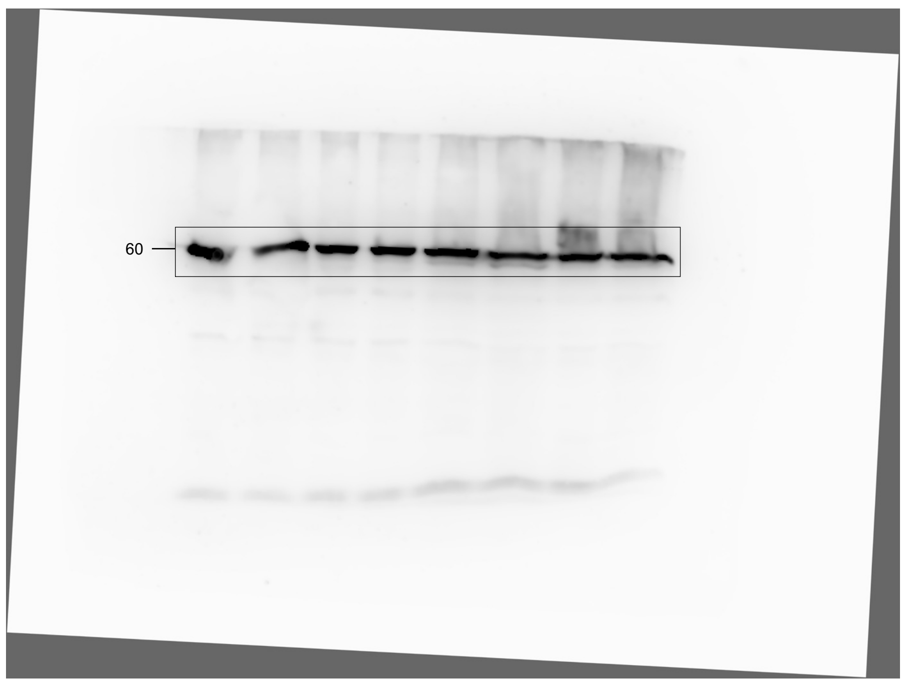

anti-AIFM1

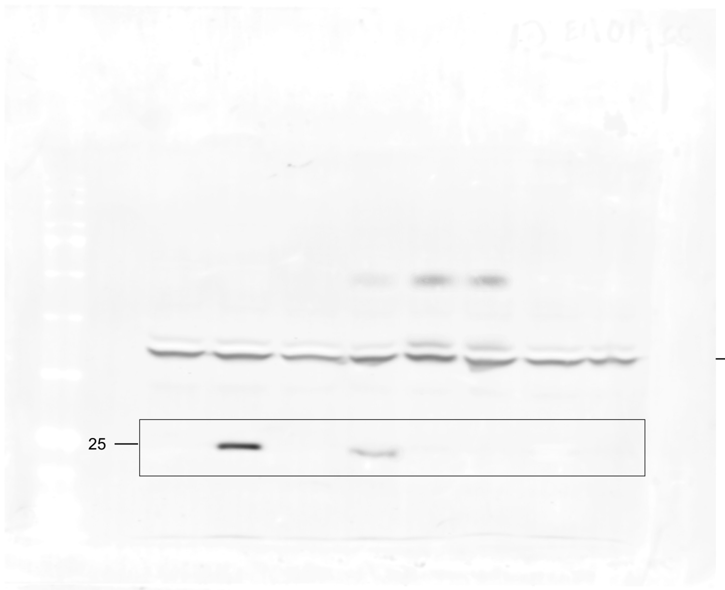

Previously blotted anti-GAPDH band

anti-Flag (A89)

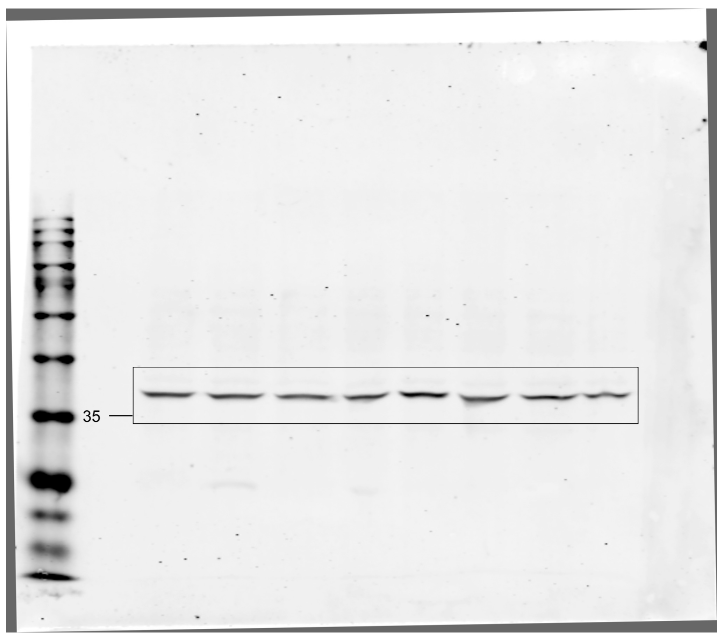

anti-GAPDH

Figure 3I
IP

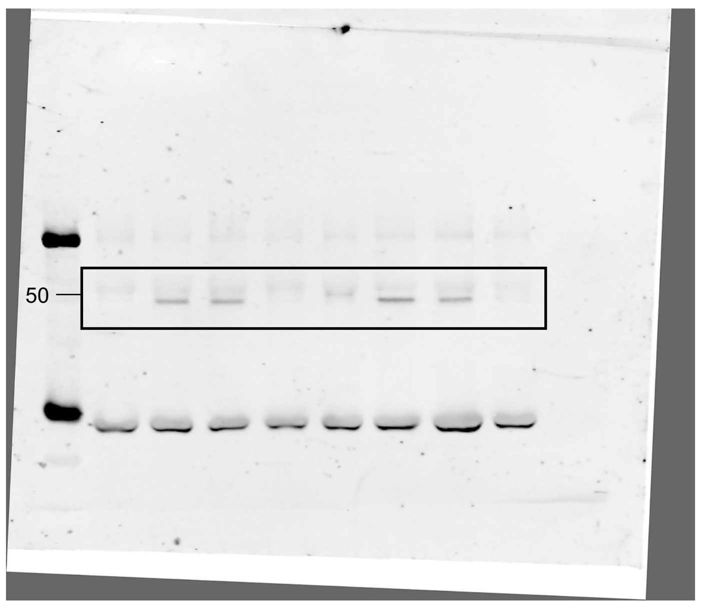

anti-TSG101

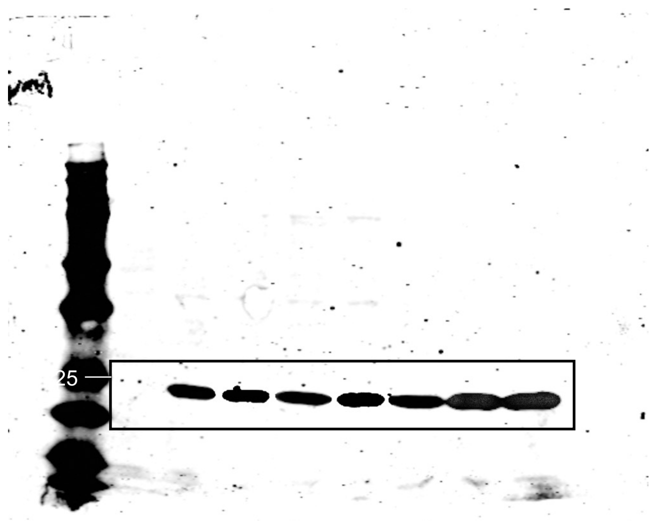

anti-Flag(MAD2)

Figure 3I
Input

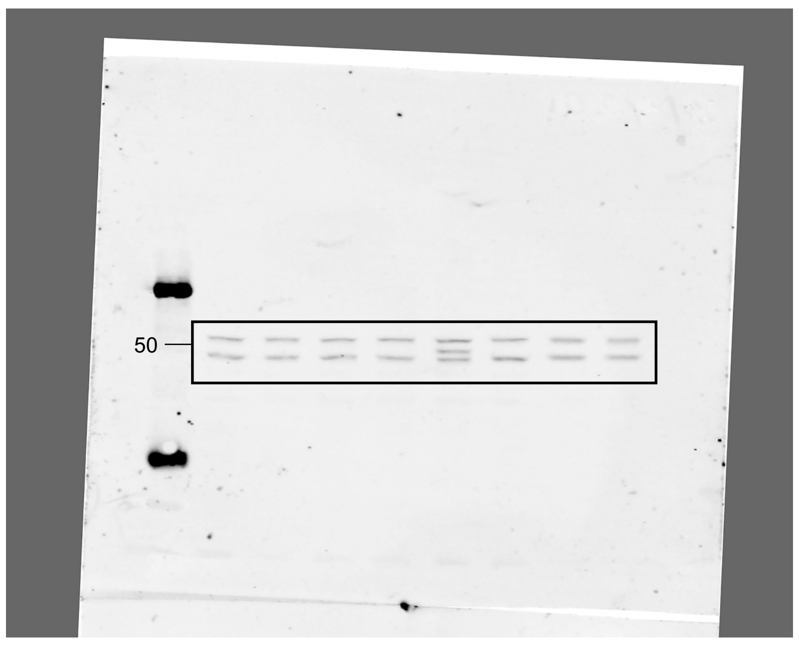

anti-TSG101

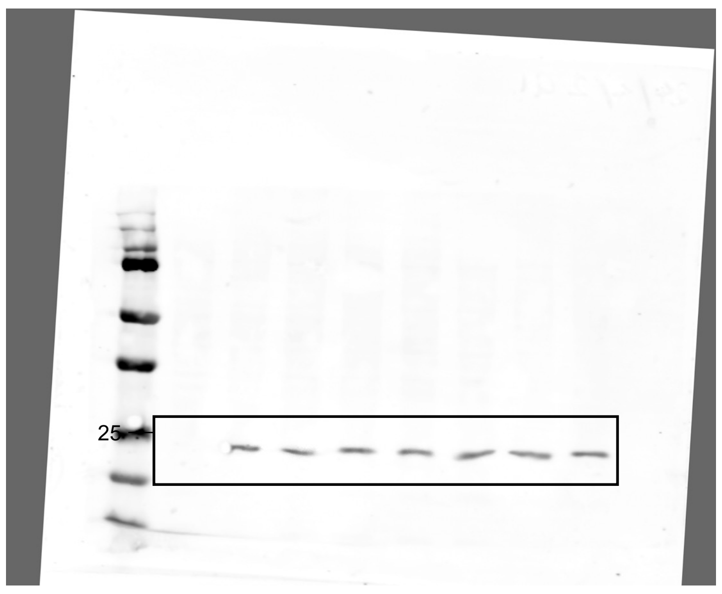

anti-Flag(MAD2)

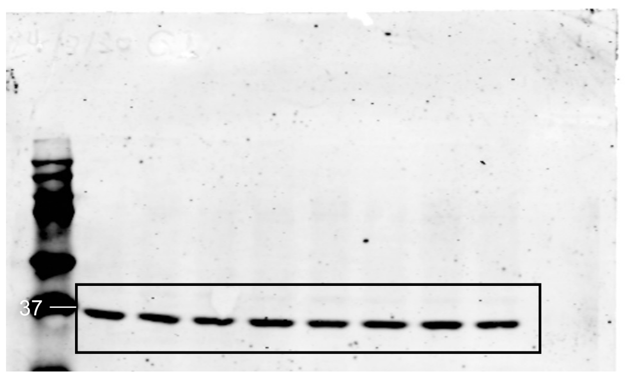

anti-GAPDH

Figure 3K
IP

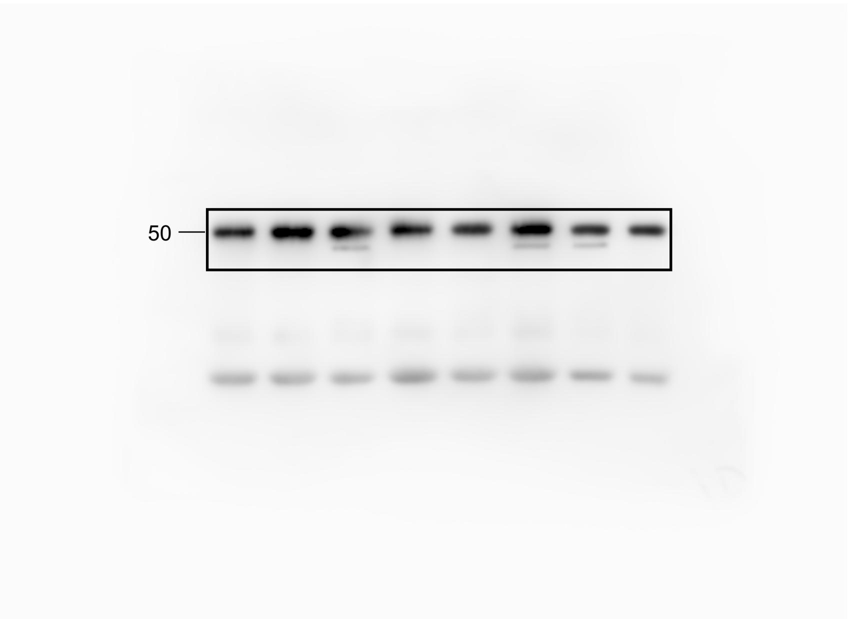

anti-HA(TSG101)

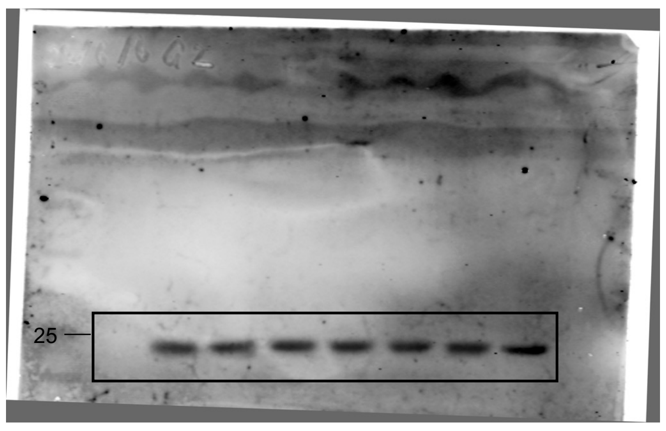

anti-Flag(MAD2)

Figure 3K
Input

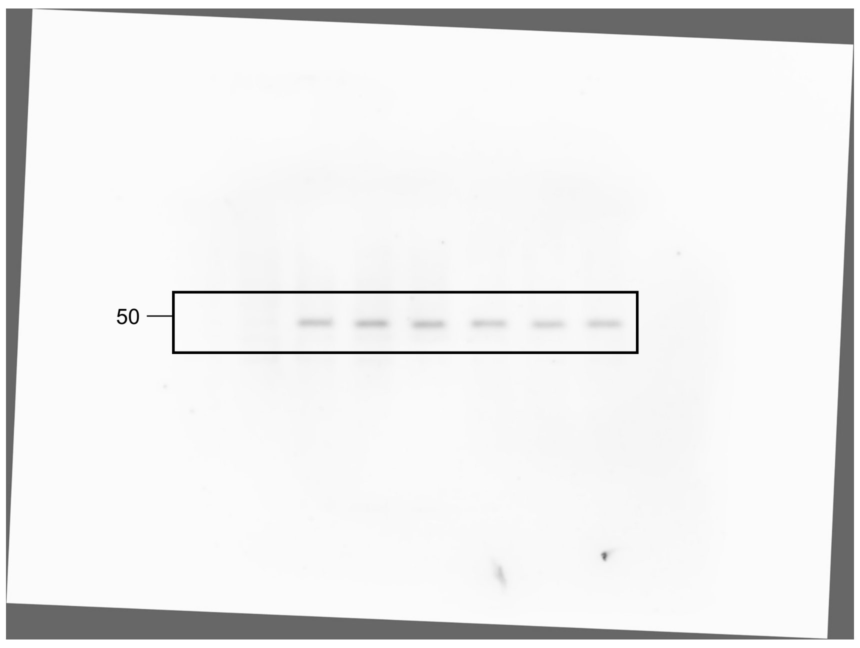

anti-HA(TSG101)

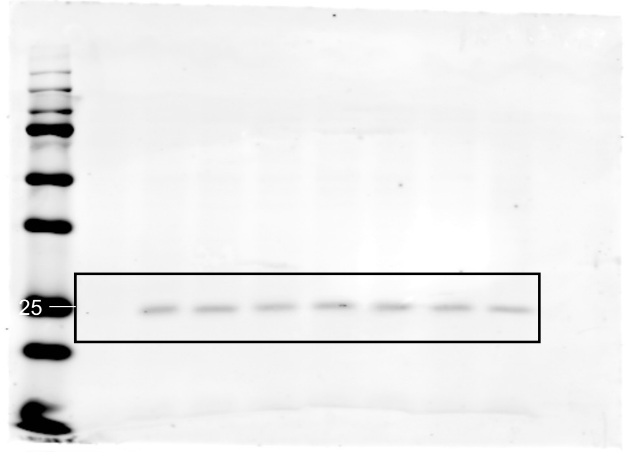

anti-MAD2

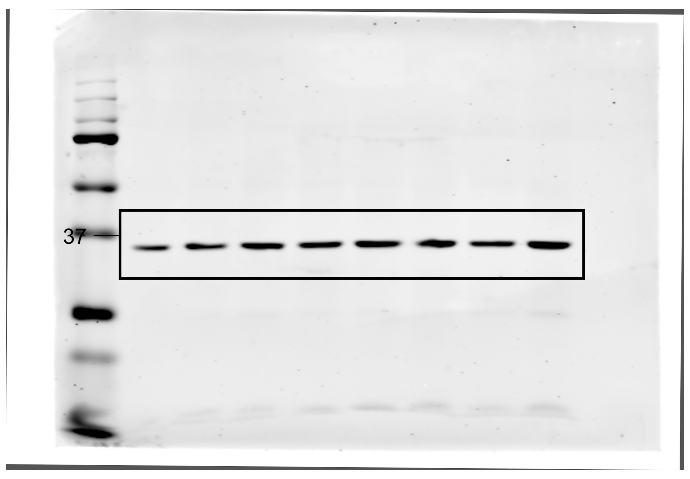

anti-GAPDH

Figure 4B

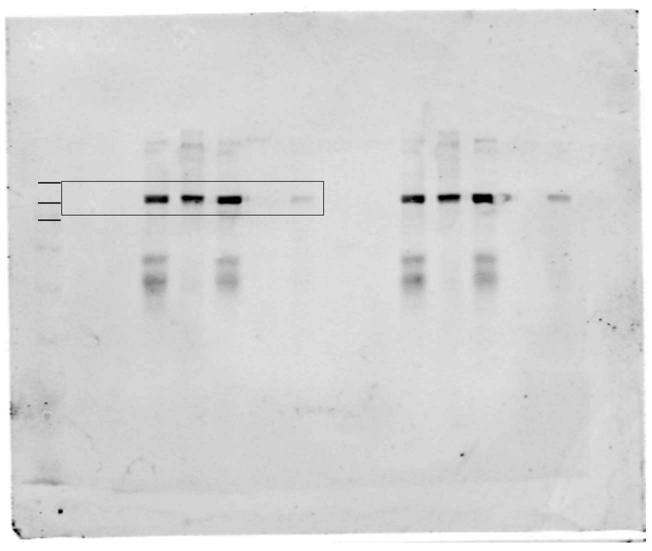

anti-HA(PML)

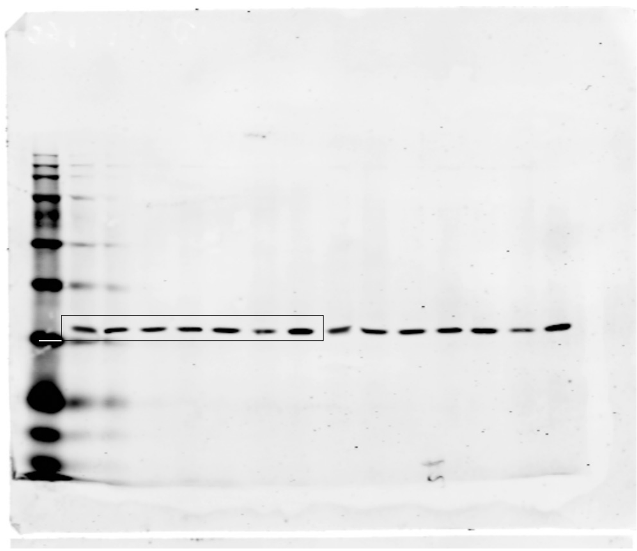

anti-GAPDH

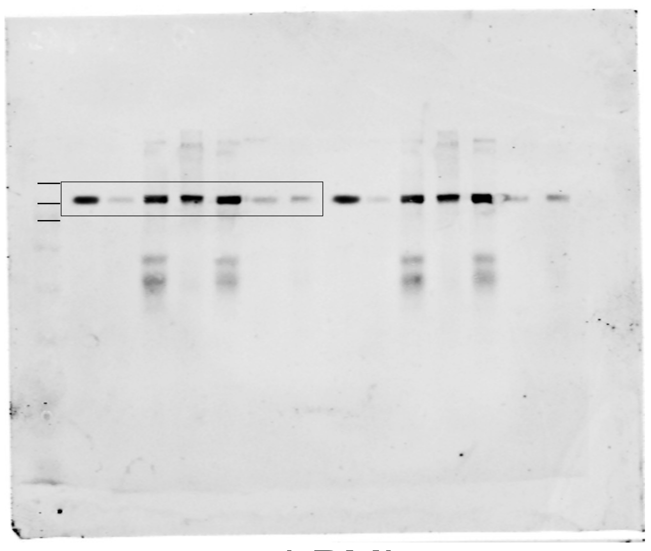

anti-PML

Figure 4C
IP and Input

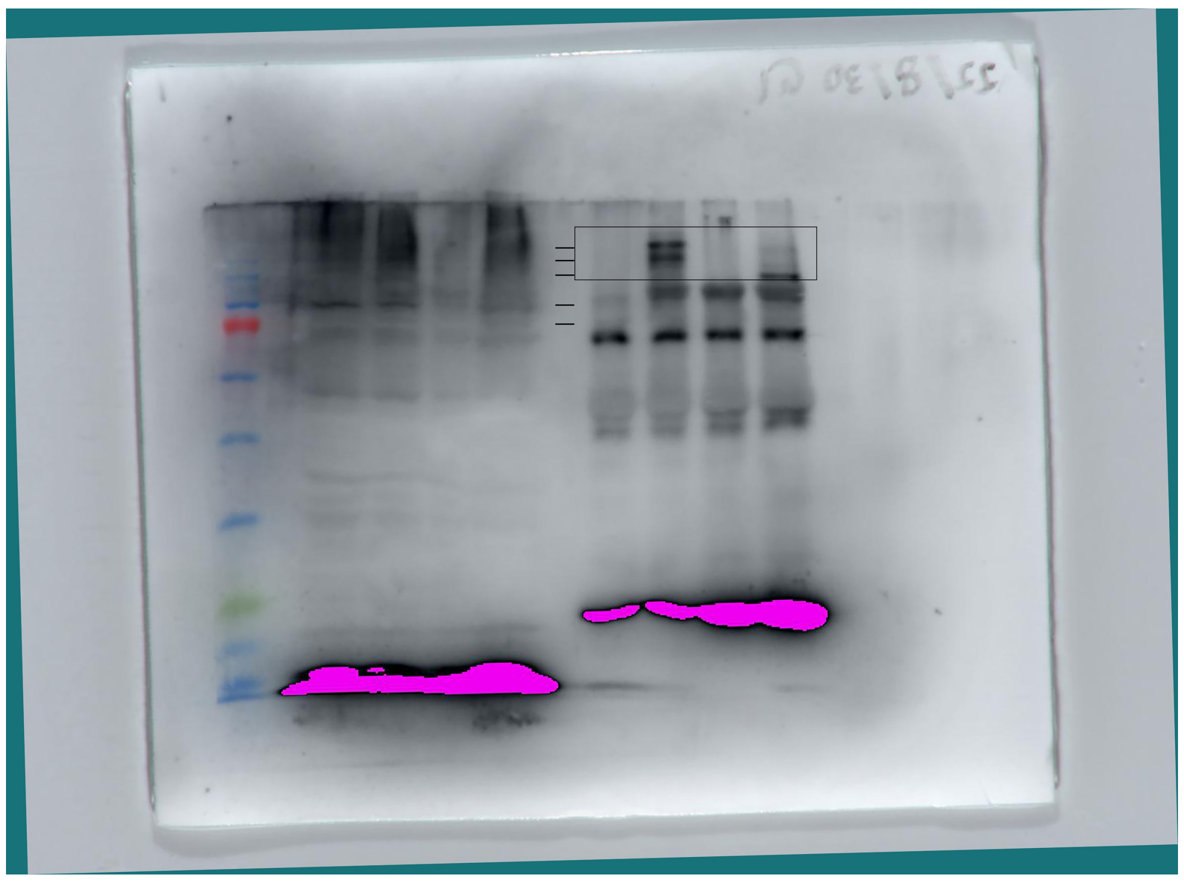

anti-SUMO2
(IP)

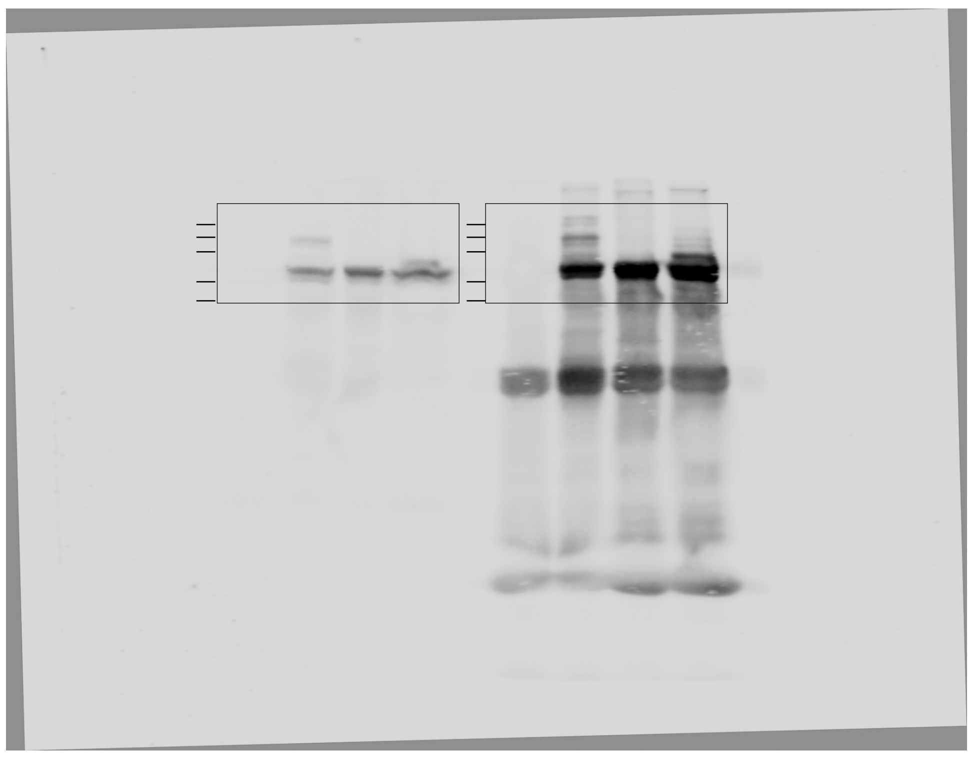

anti-Flag(PML
(Input)

anti-Flag(PML
(IP)

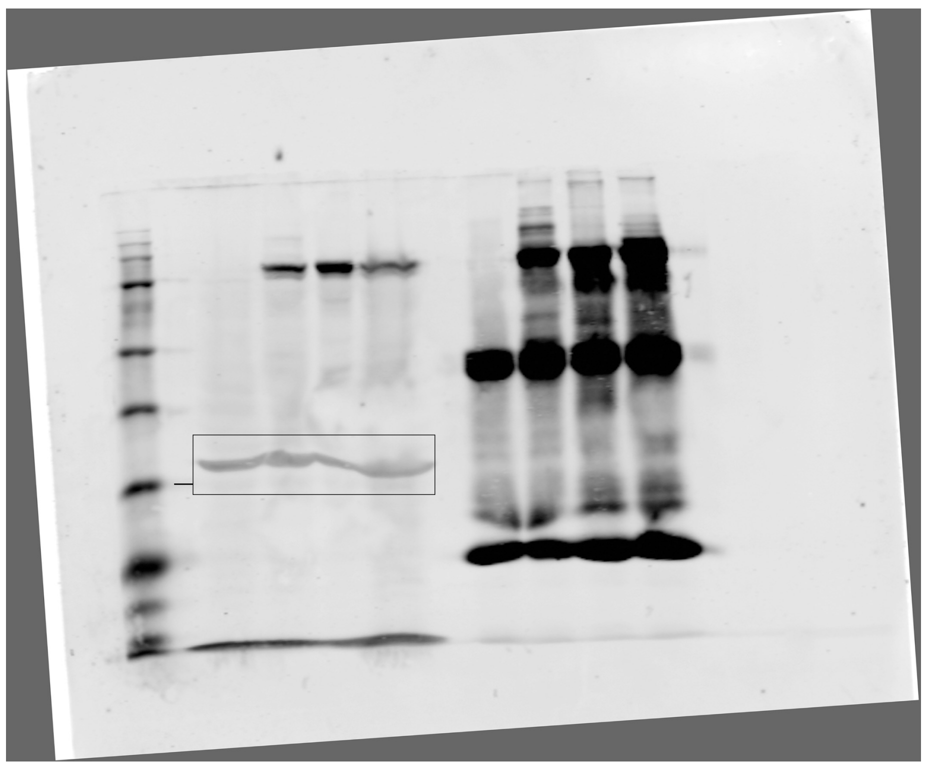

anti-GAPDH
(Input)

Figure 4F
IP

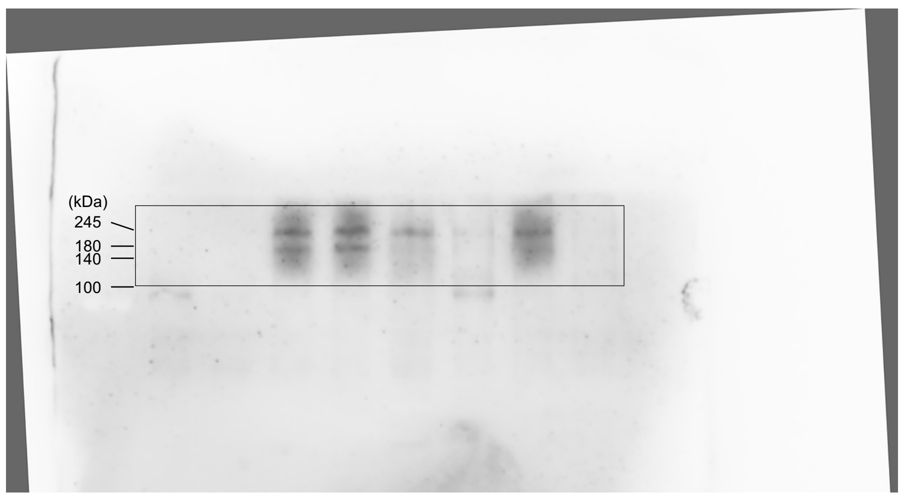

anti-SUMO2

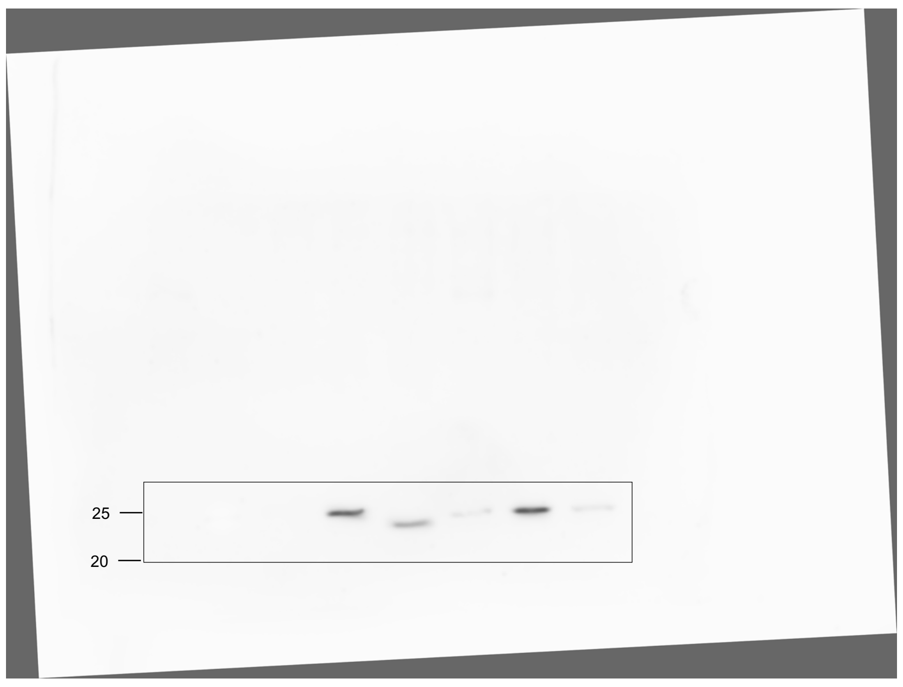

anti-MAD2

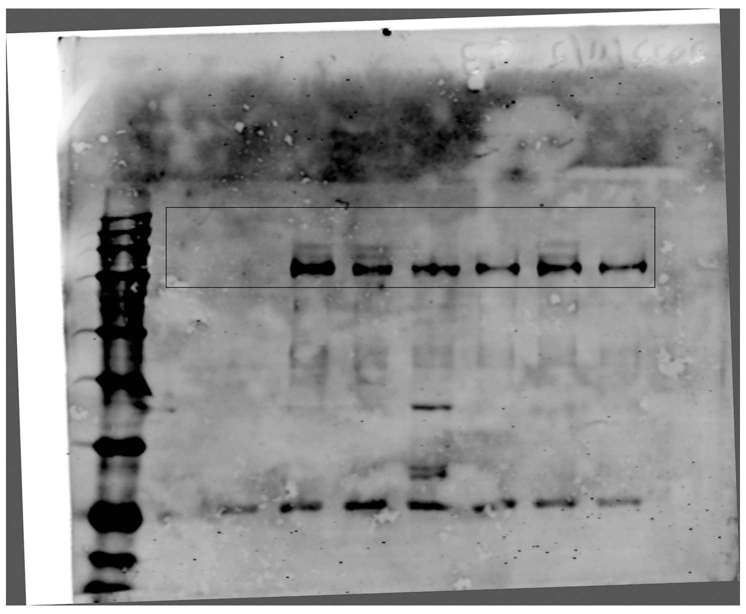

anti-HA (PML)

Figure 4F
Input

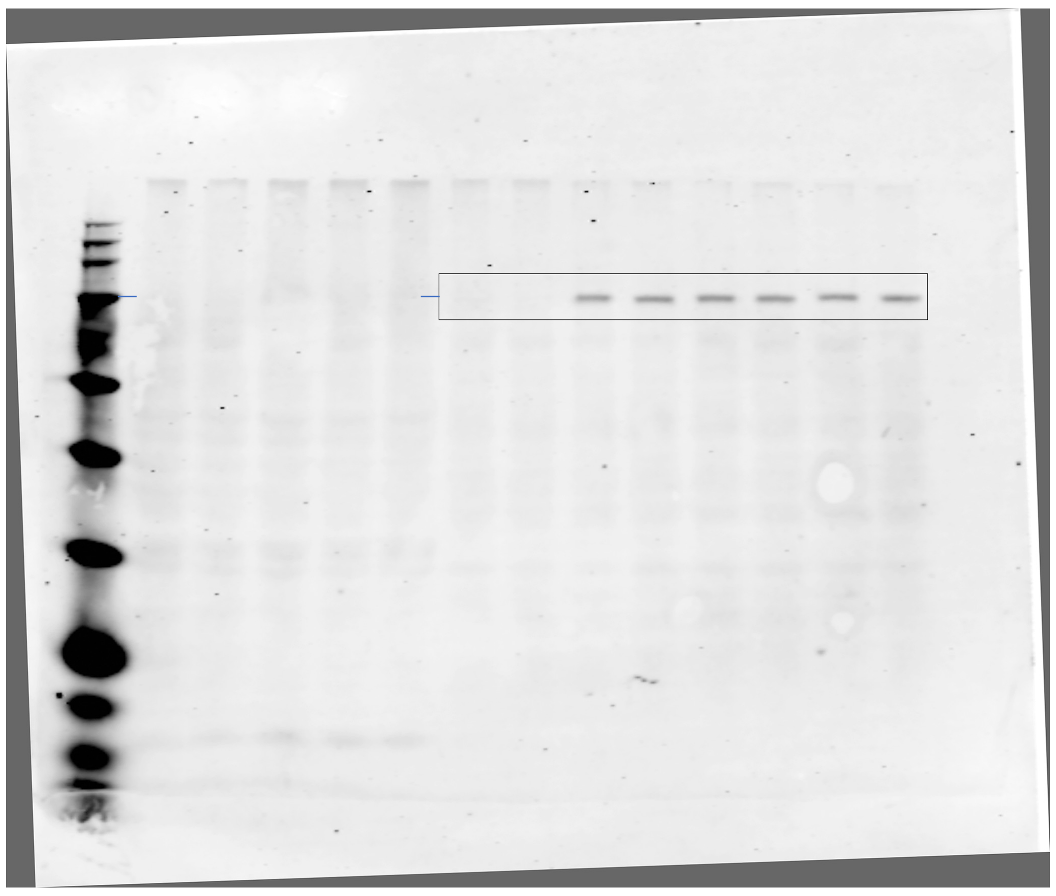

anti-HA(PML)

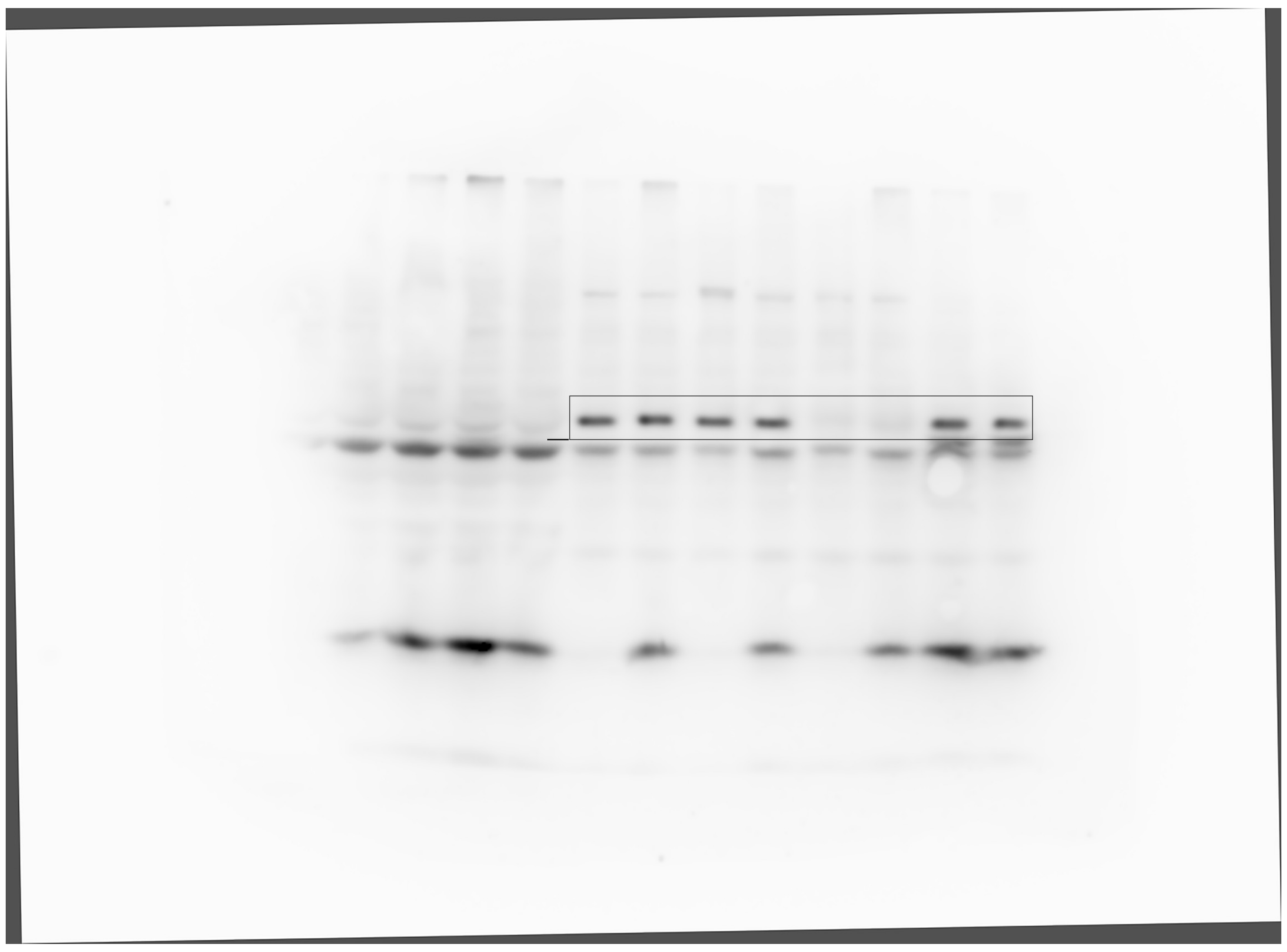

anti-TSG101

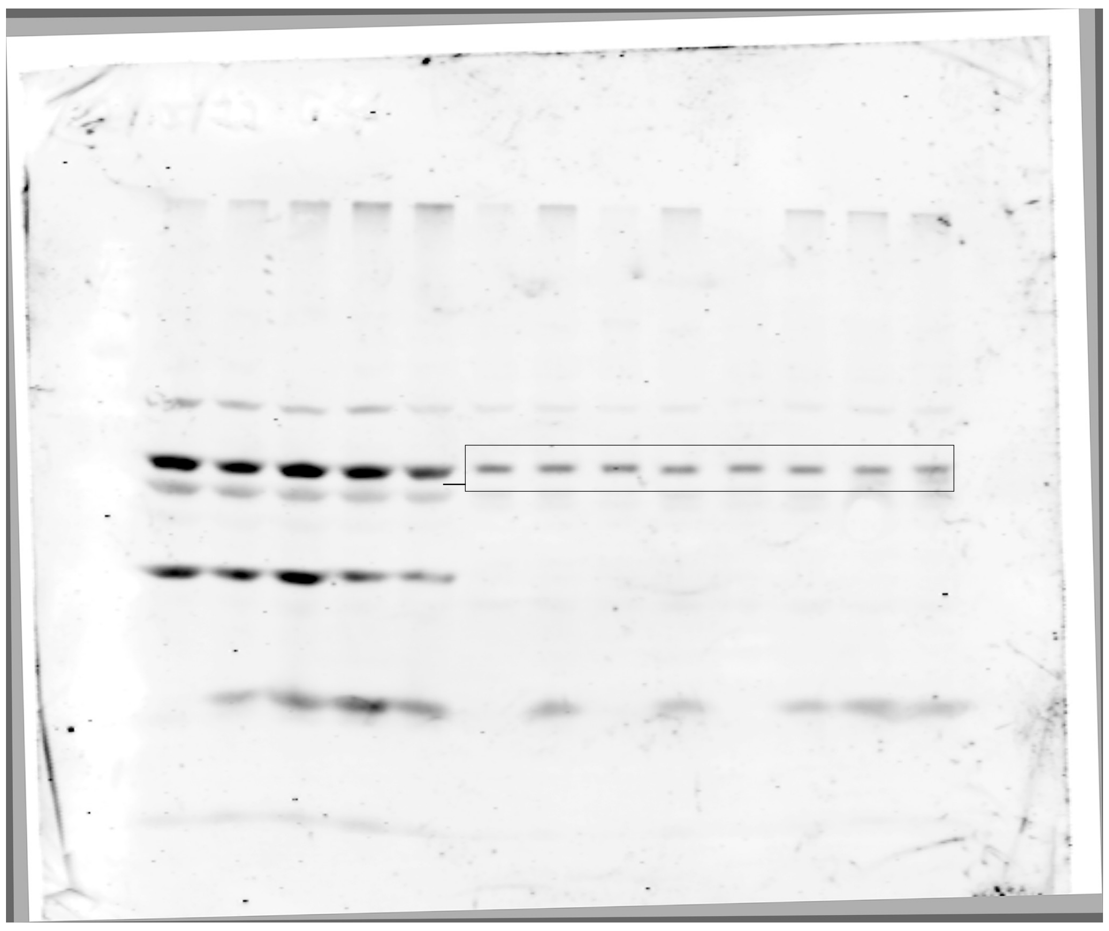

anti-β-tubulin

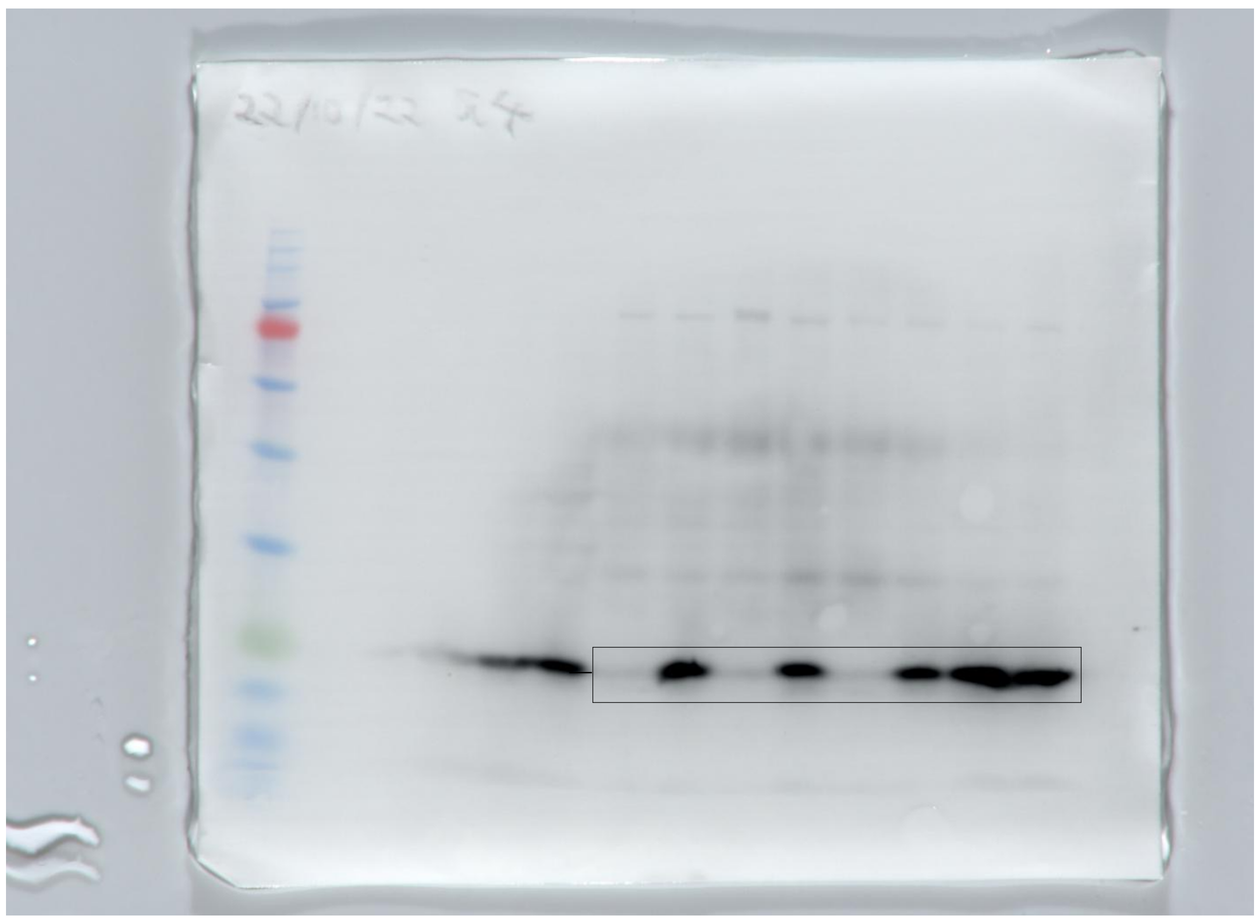

anti-MAD2

Figure 4G
IP

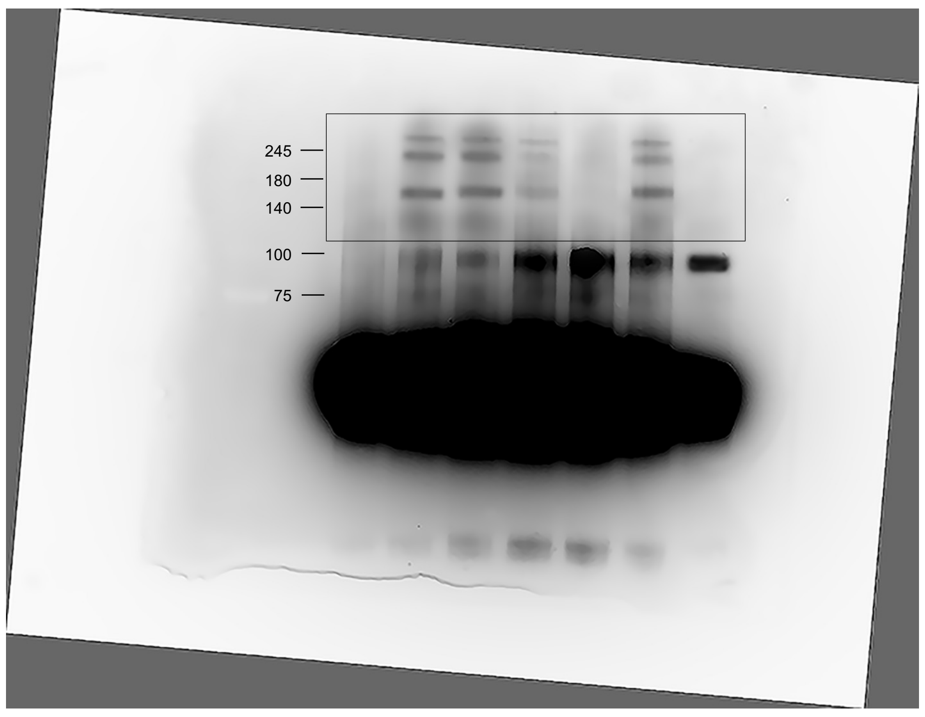

Anti-SUMO2

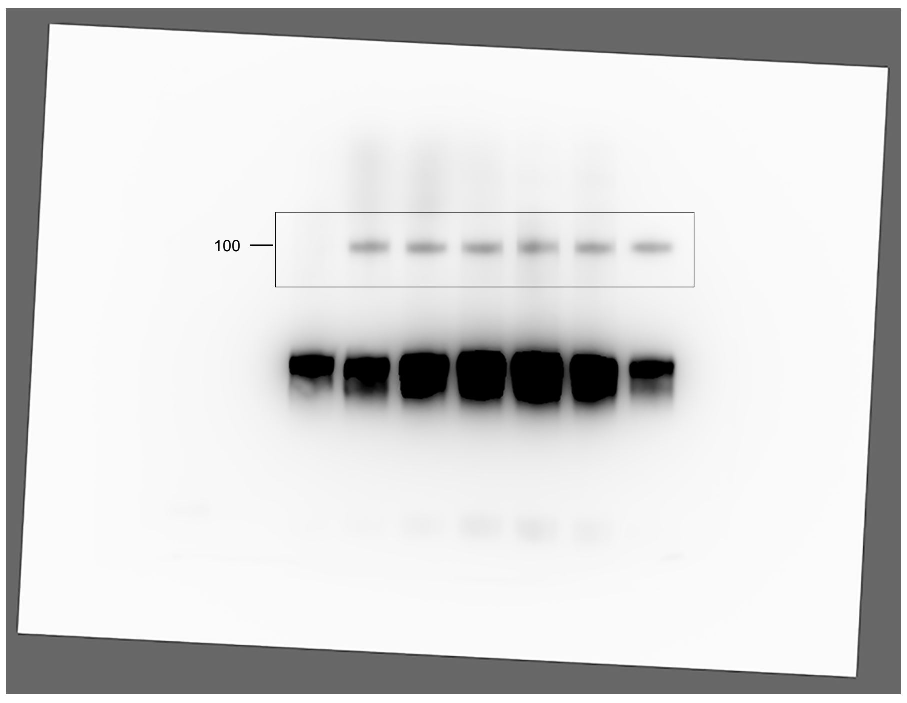

anti-PML

Figure 4G
Input

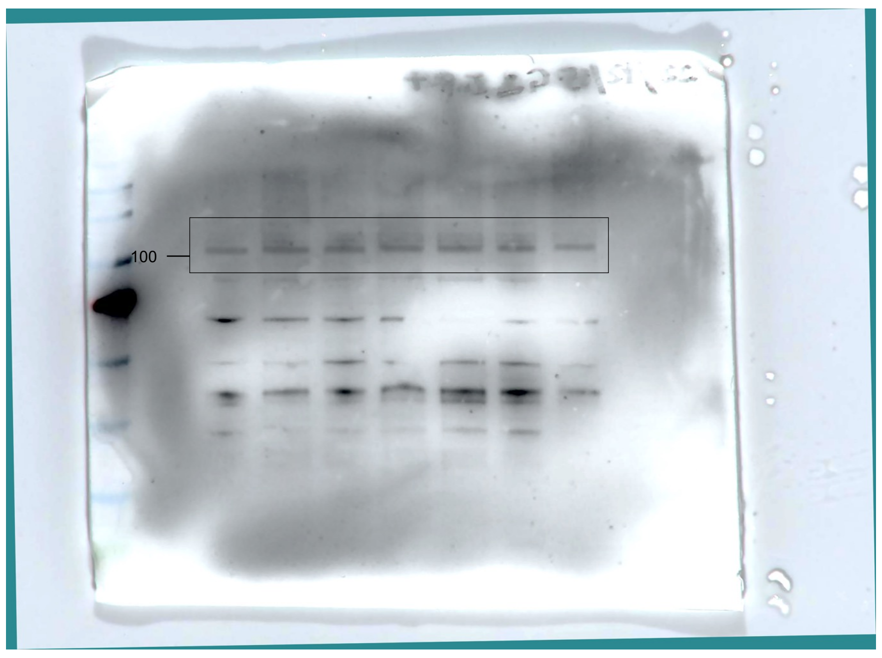

Anti-PML

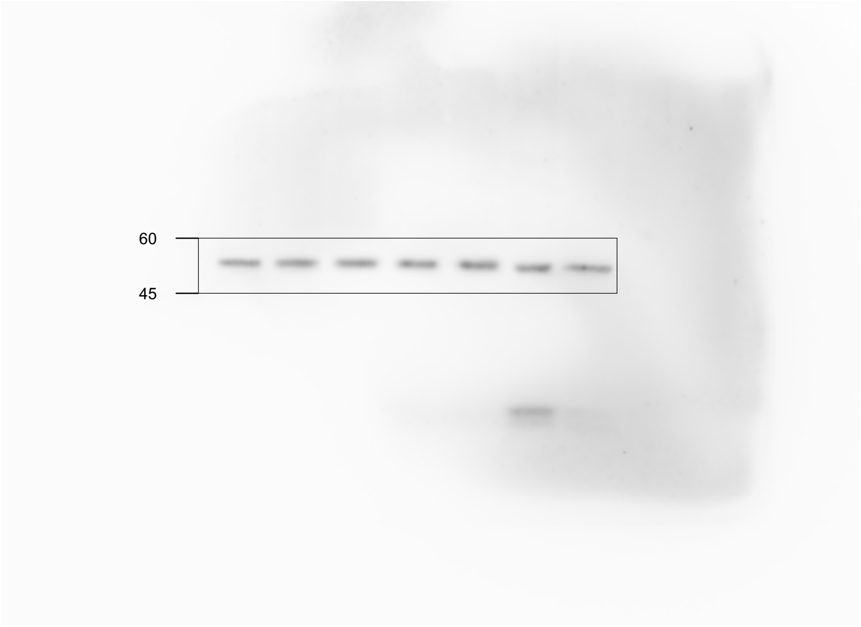

anti-β-tubulin

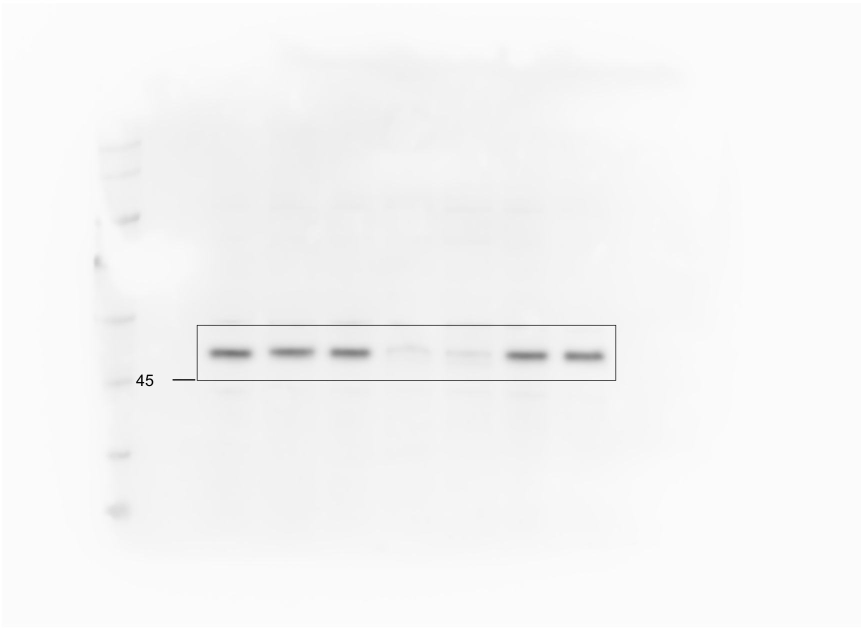

anti-TSG101

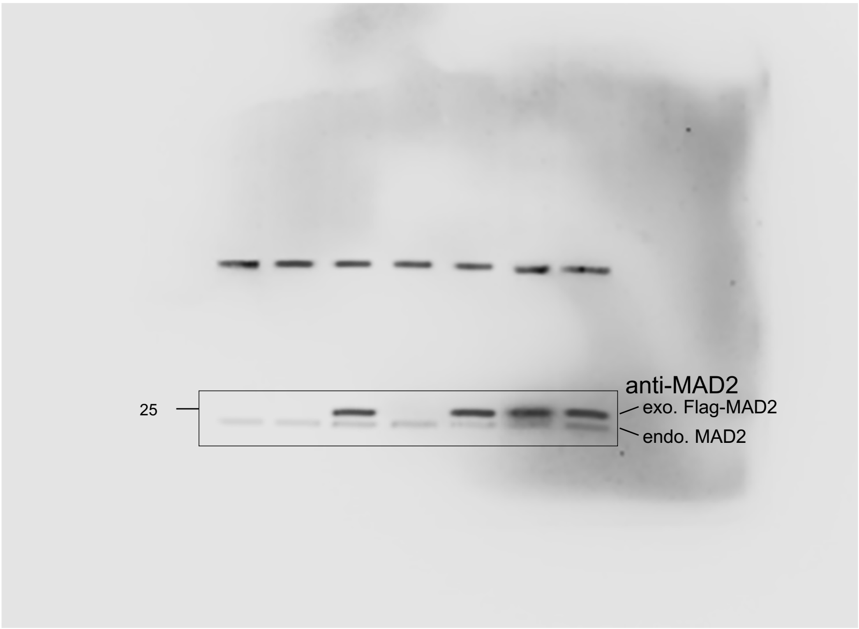

anti-MAD2
