# Supplementary material for: TSG101 depletion dysregulates mitochondria and PML NBs, triggering MAD2-overexpressing interphase cell death (MOID) through AIFM1-PML-DAXX pathway
Source: Cell Death Dis. 2024 Nov 17;15(11):838. doi: 10.1038/s41419-024-07229-w (PMC11570632; doi:10.1038/s41419-024-07229-w)
Supplement: Supplementary file 3 — Original western blots in Supplemental Figures [file 41419_2024_7229_MOESM3_ESM.pdf]

Figure S1B

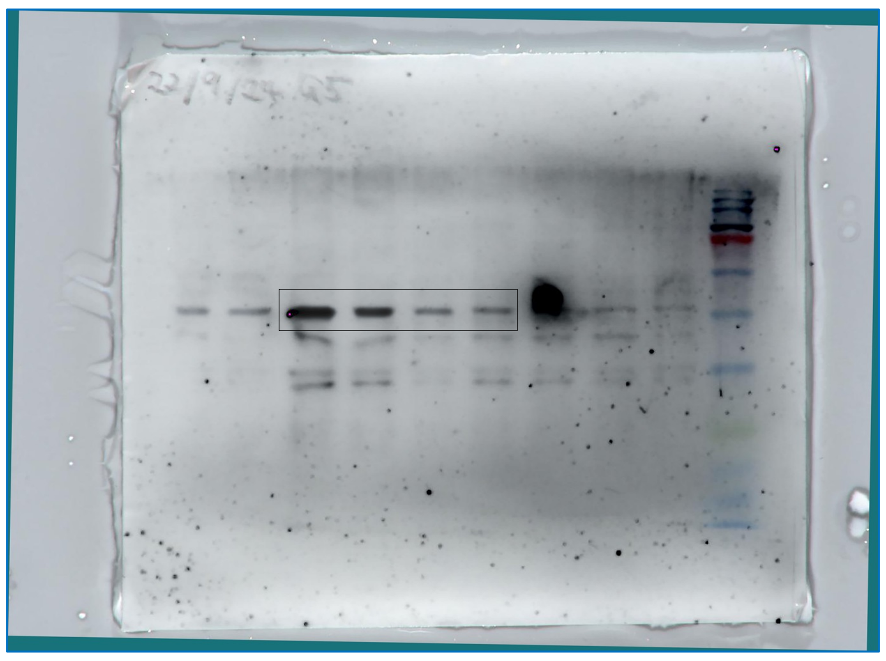

anti-TSG101

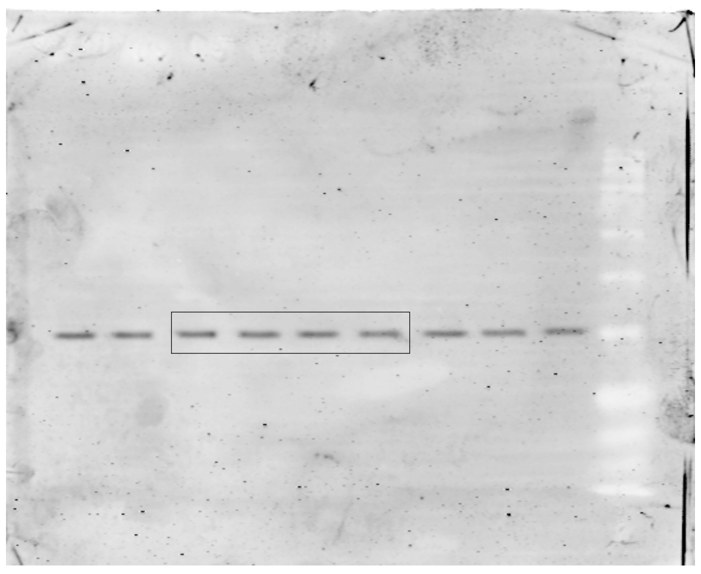

anti-GAPDH

Figure S2B

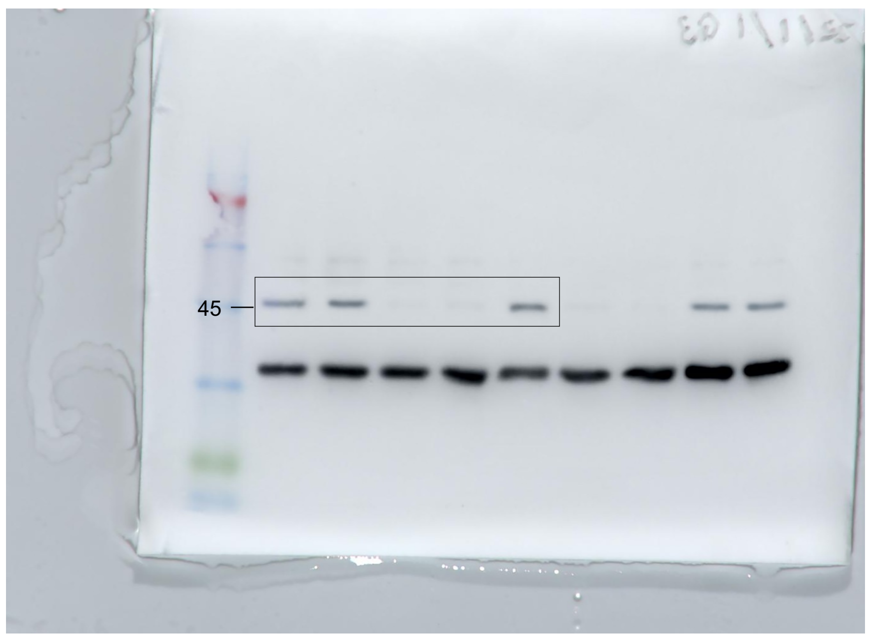

anti-TSG101

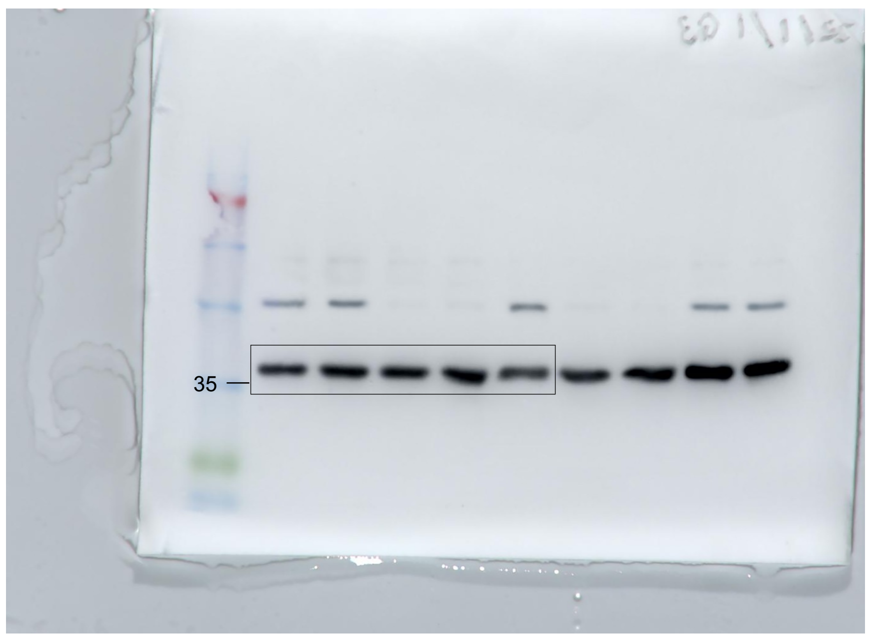

anti-GAPDH

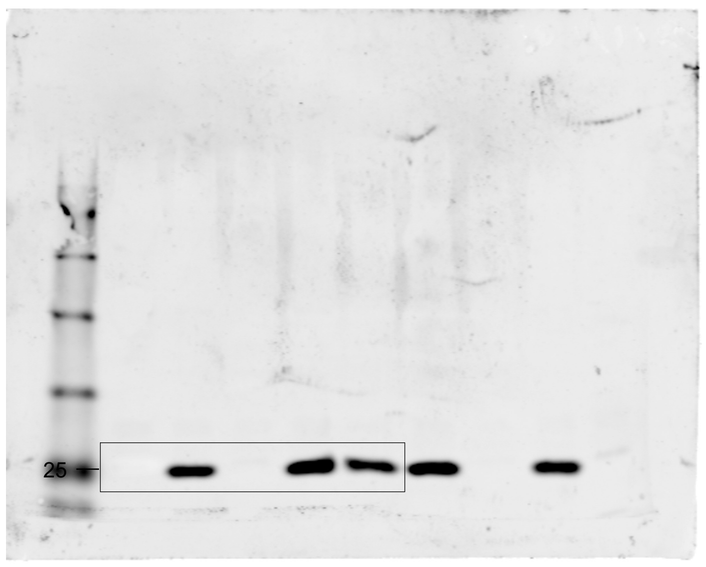

anti-Flag(MAD2)

Figure S2D Left

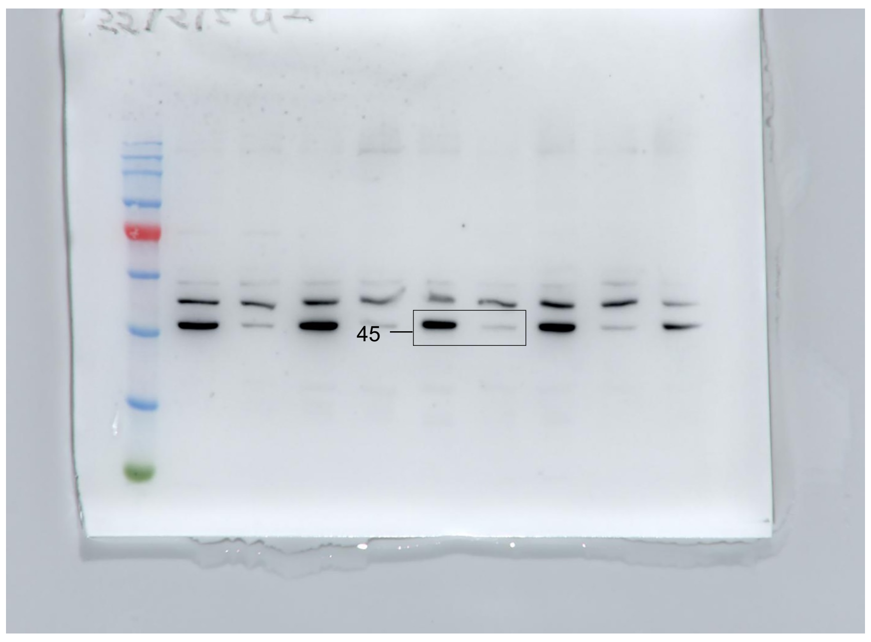

anti-TSG101

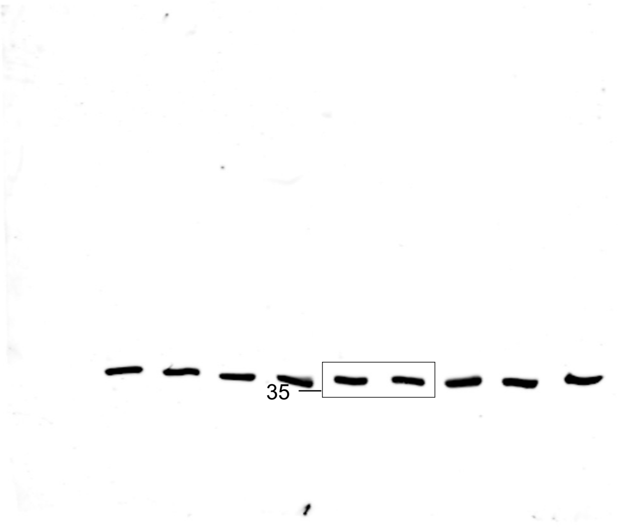

anti-GAPDH

Figure S2D Right

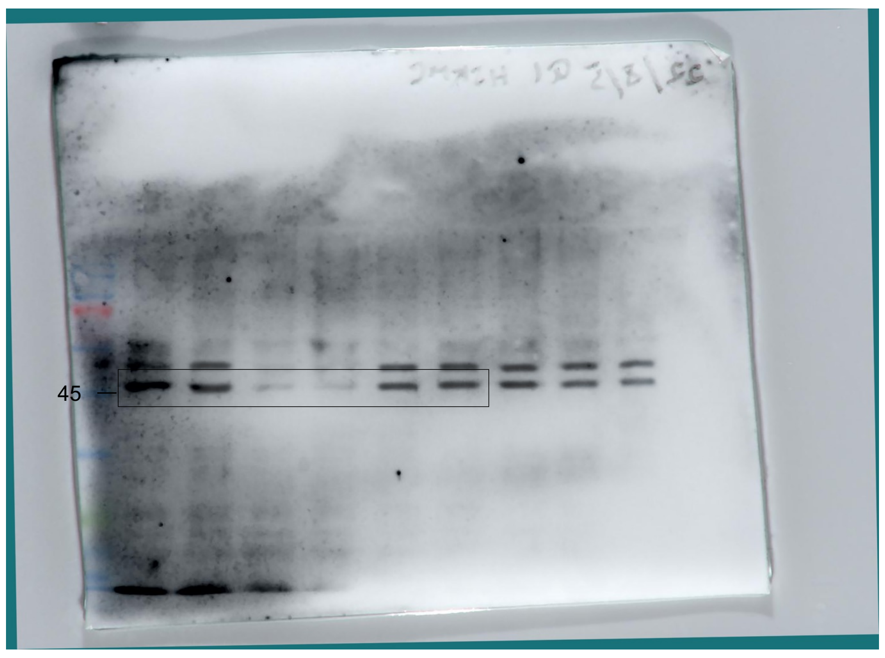

anti-TSG101

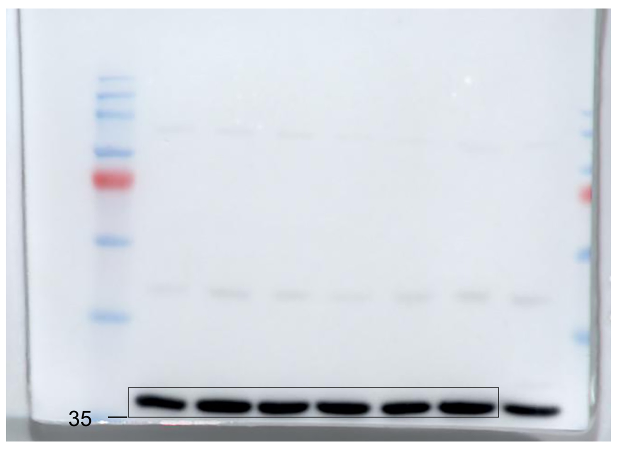

anti-GAPDH

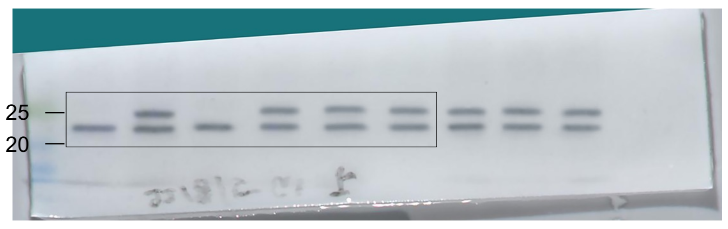

anti-MAD2

Figure S2G

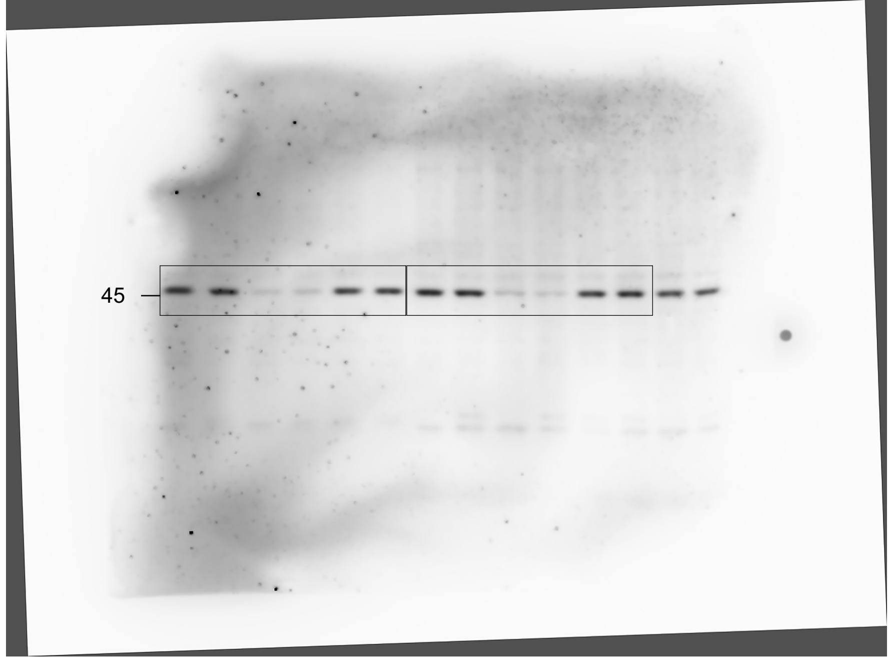

anti-TSG01
A549 (p53 KO)

anti-TSG01
A549

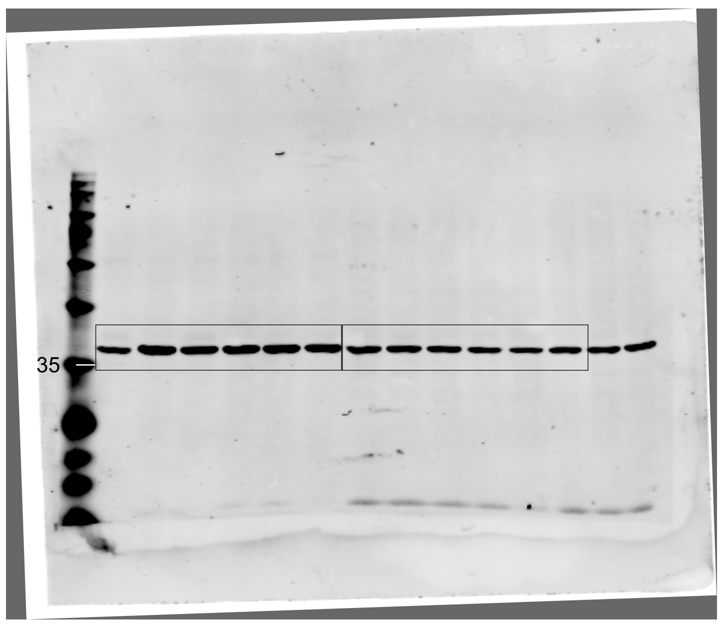

anti-GAPDH
A549 (p53 KO)

anti-GAPDH
A549

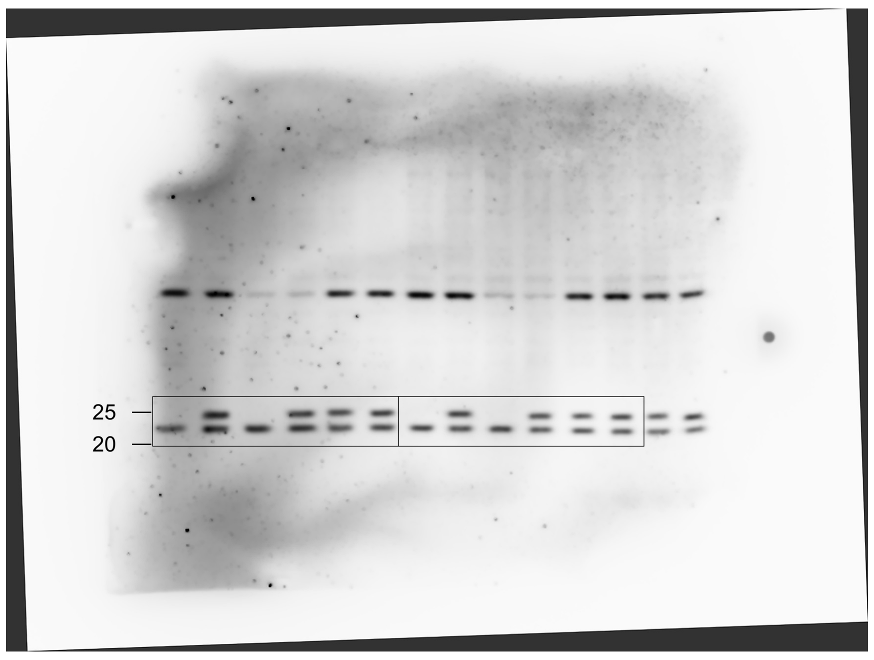

anti-MAD2
A549 (p53 KO)

anti-MAD2
A549

Note: Left-Right box is reverted in the publication format
to be consistent with the order of immunofluorescence images.

Figure S3C

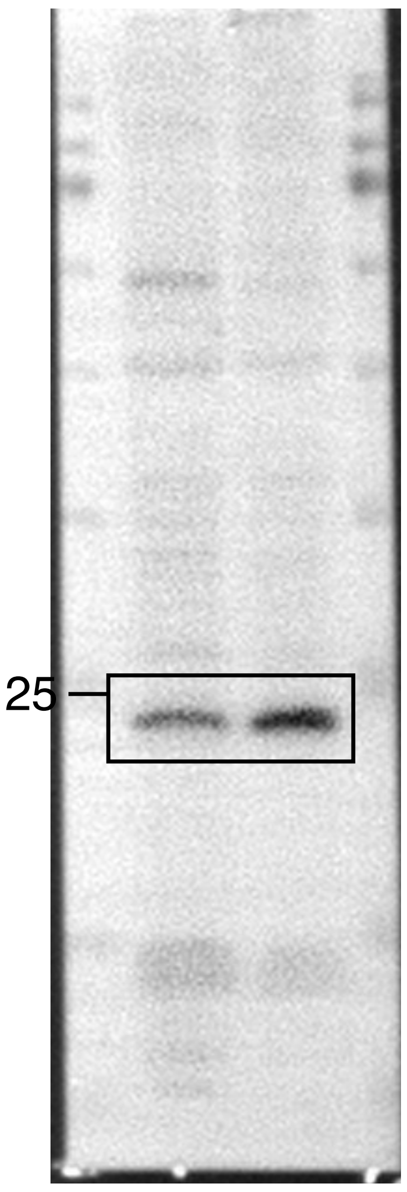

anti-MAD2

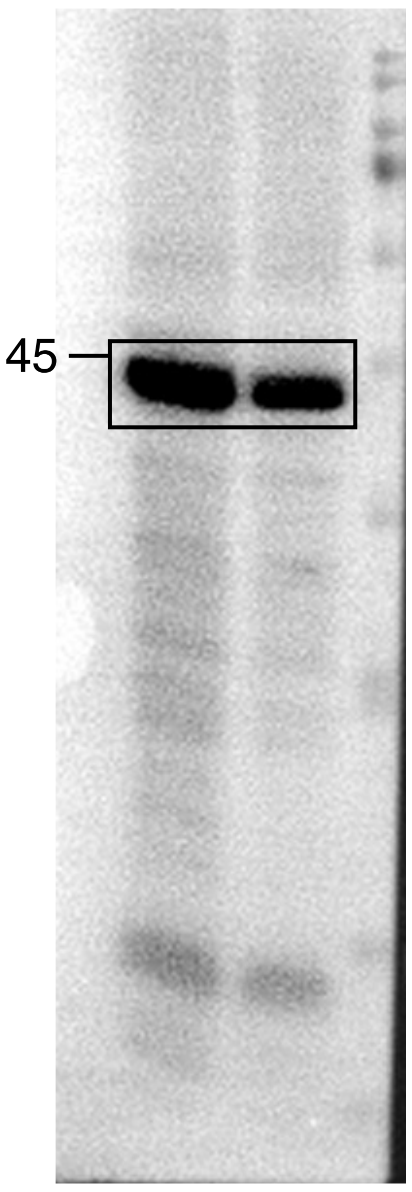

anti-ACTB

Figure S4A

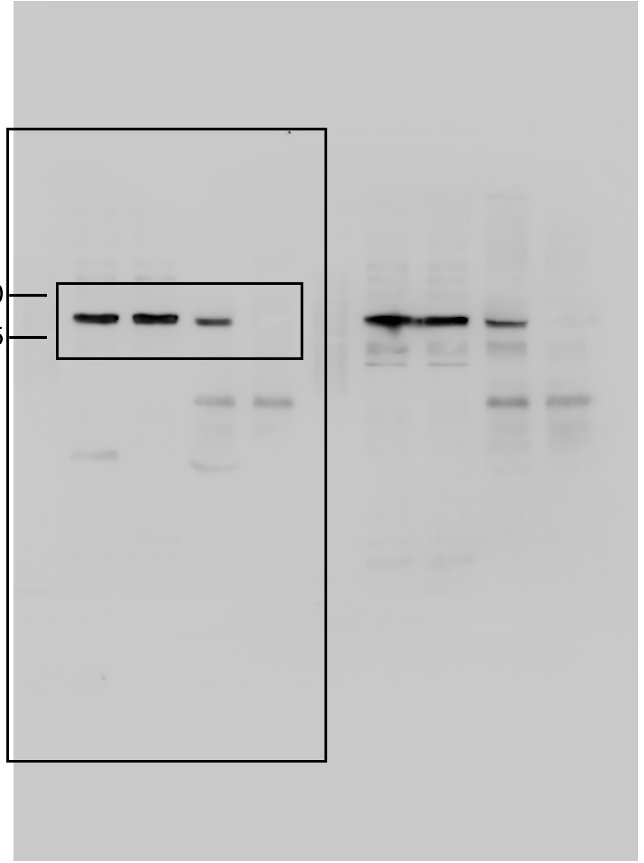

anti-p53

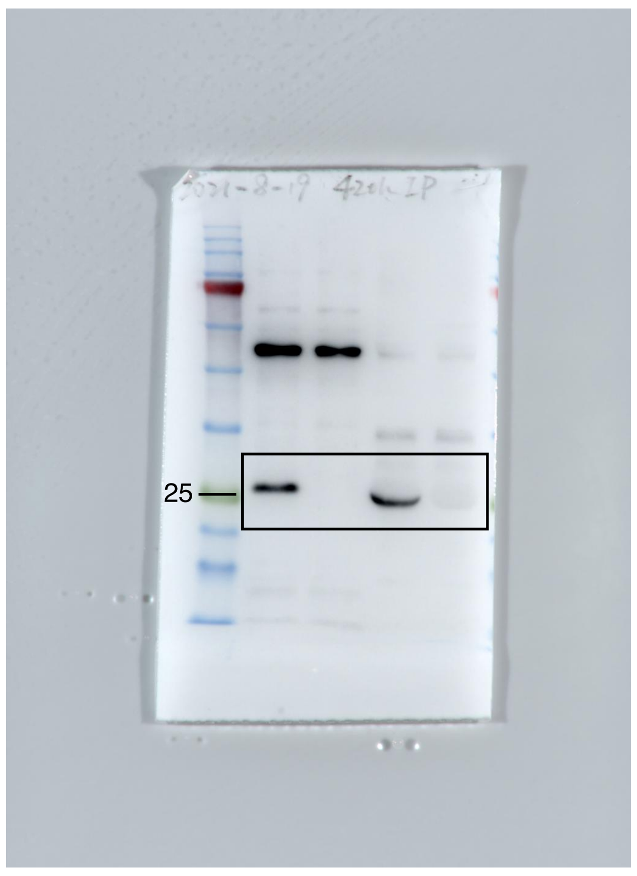

anti-Flag(MAD2)

Figure S4B

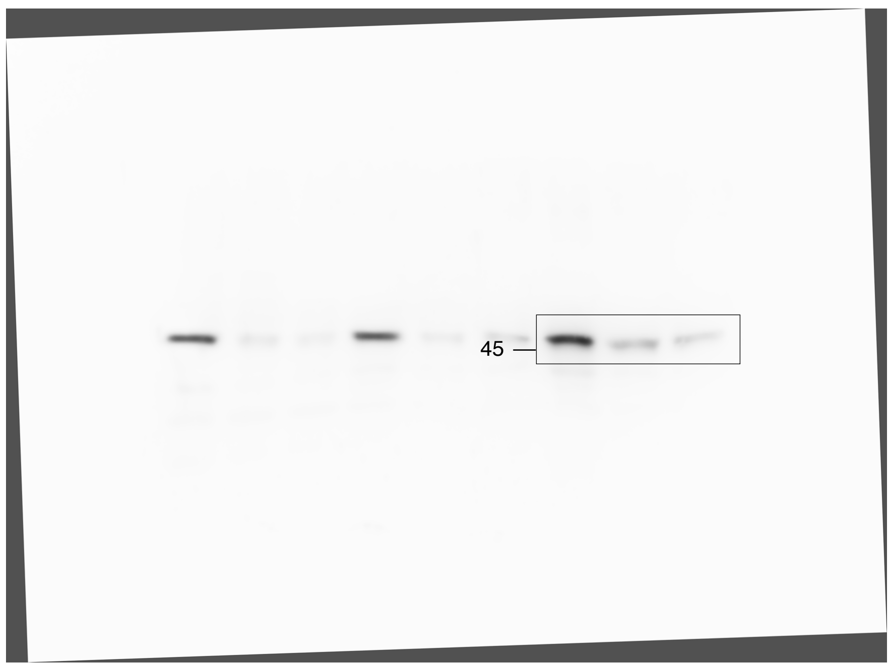

anti-p53

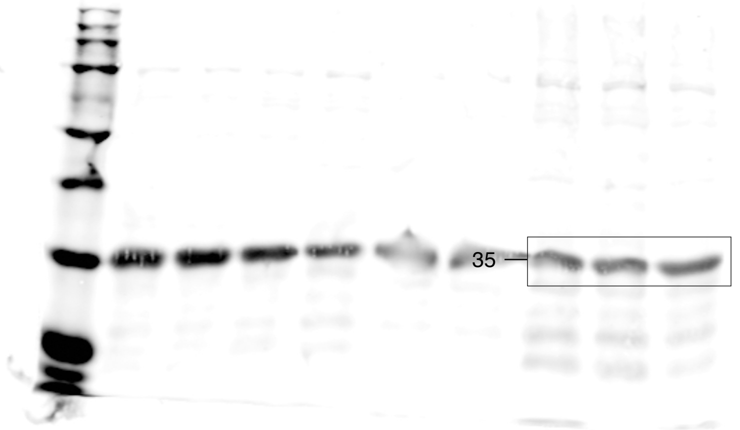

anti-GAPDH

Figure S4C

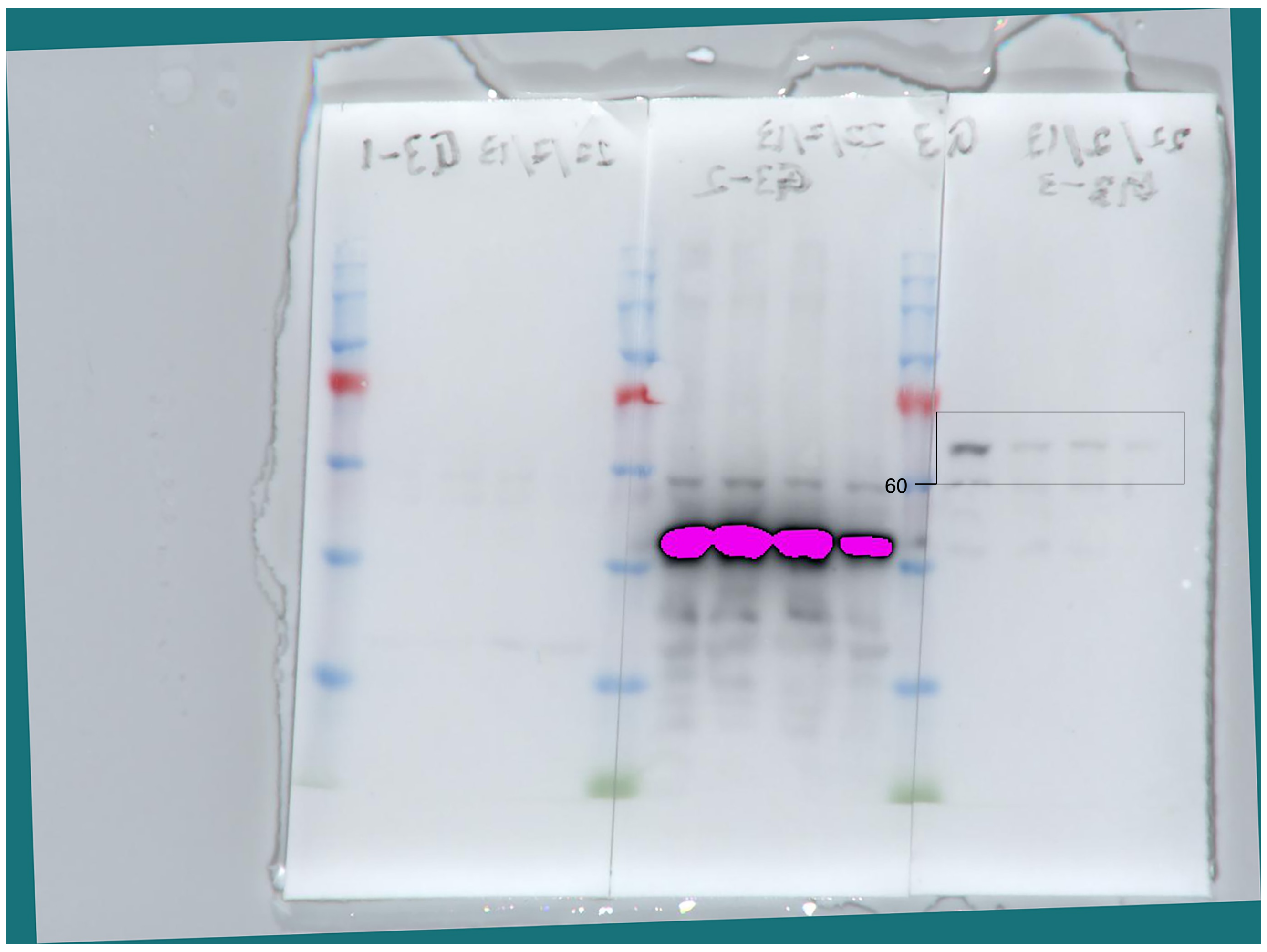

anti-AIFM1

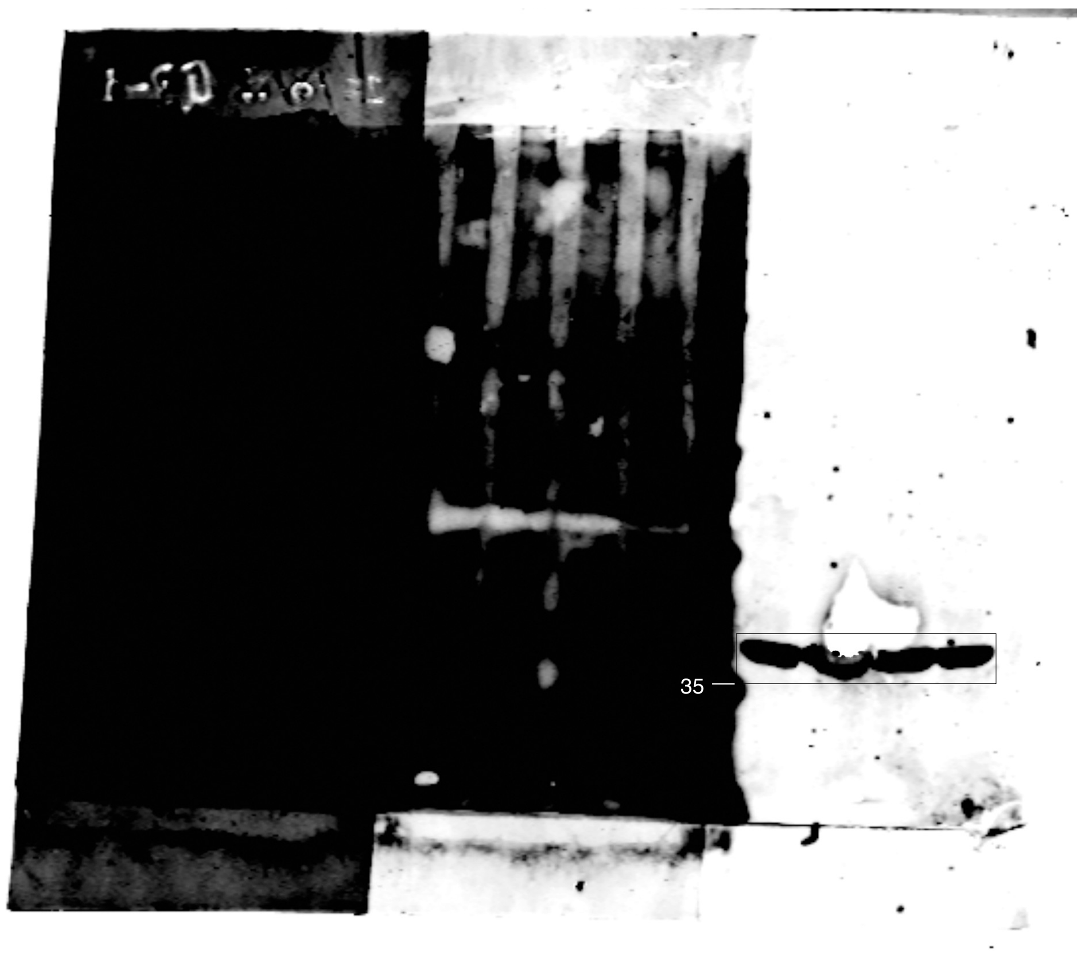

anti-GAPDH

Figure S4D

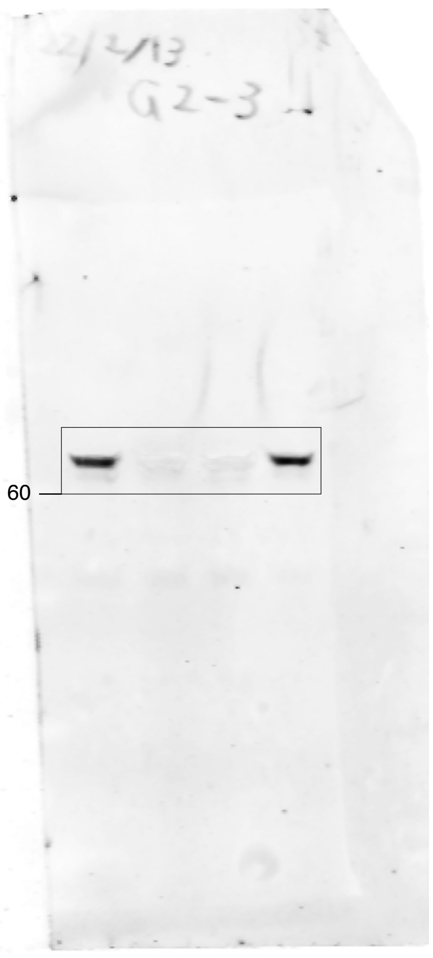

anti-AIFM1

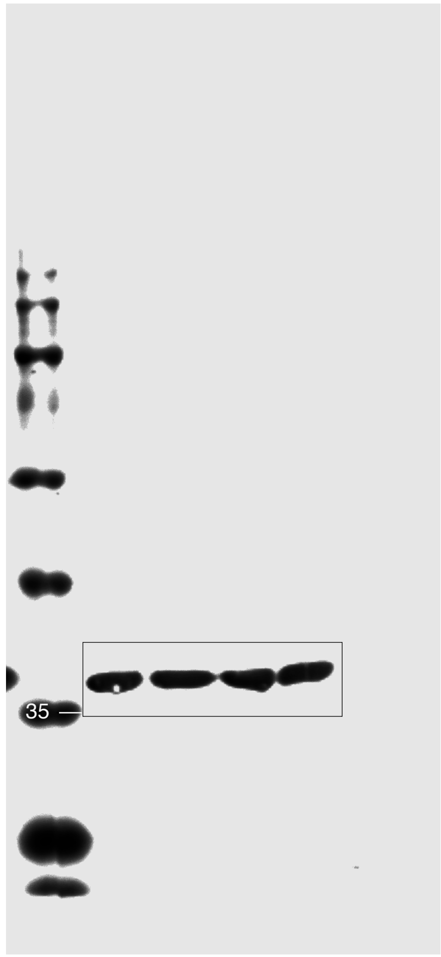

anti-GAPDH

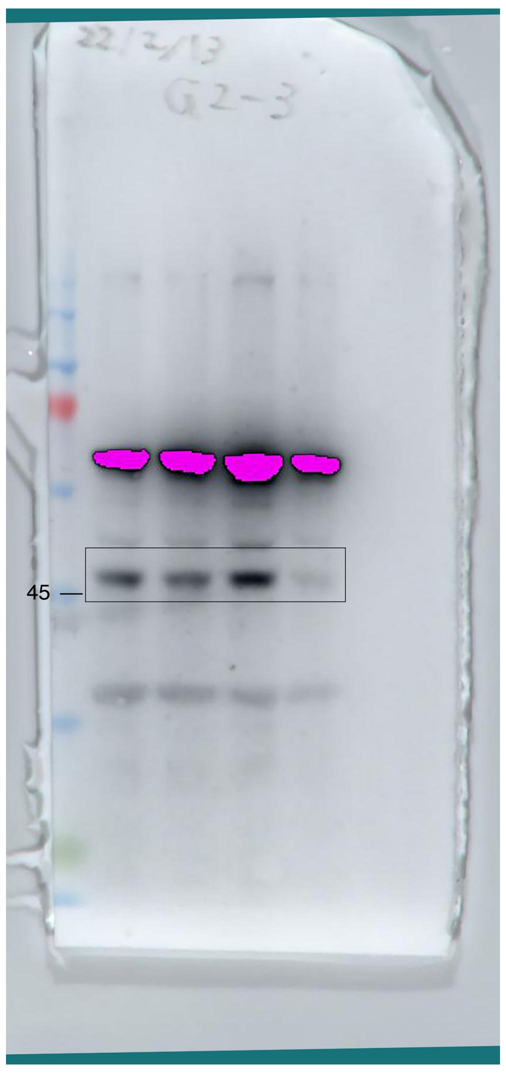

anti-TSG101

Figure S4F
Input

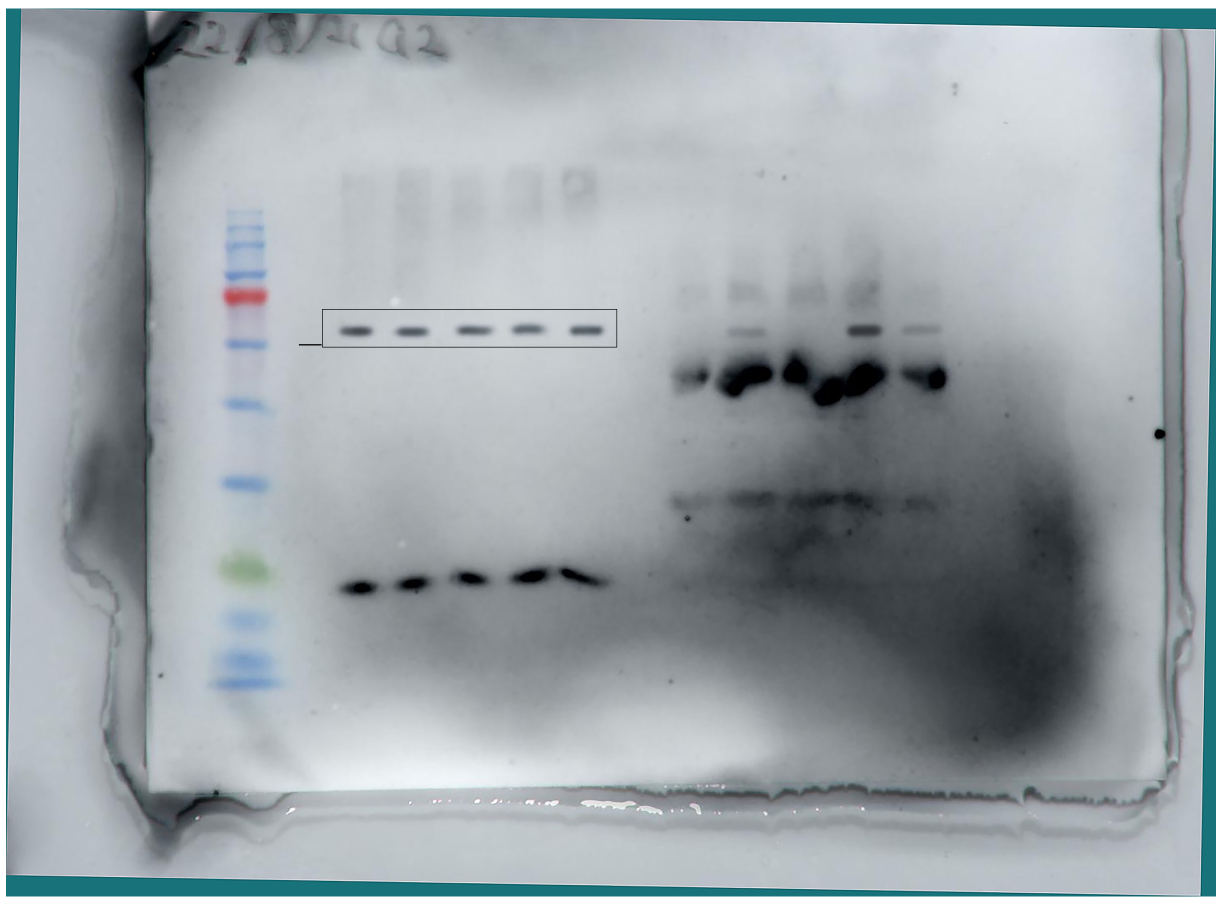

anti-AIFM1

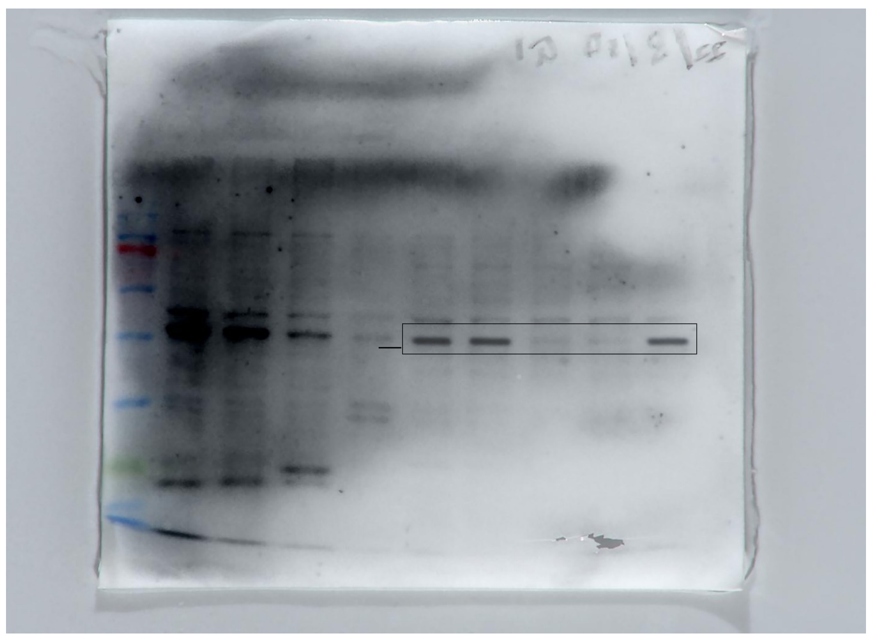

anti-TSG101

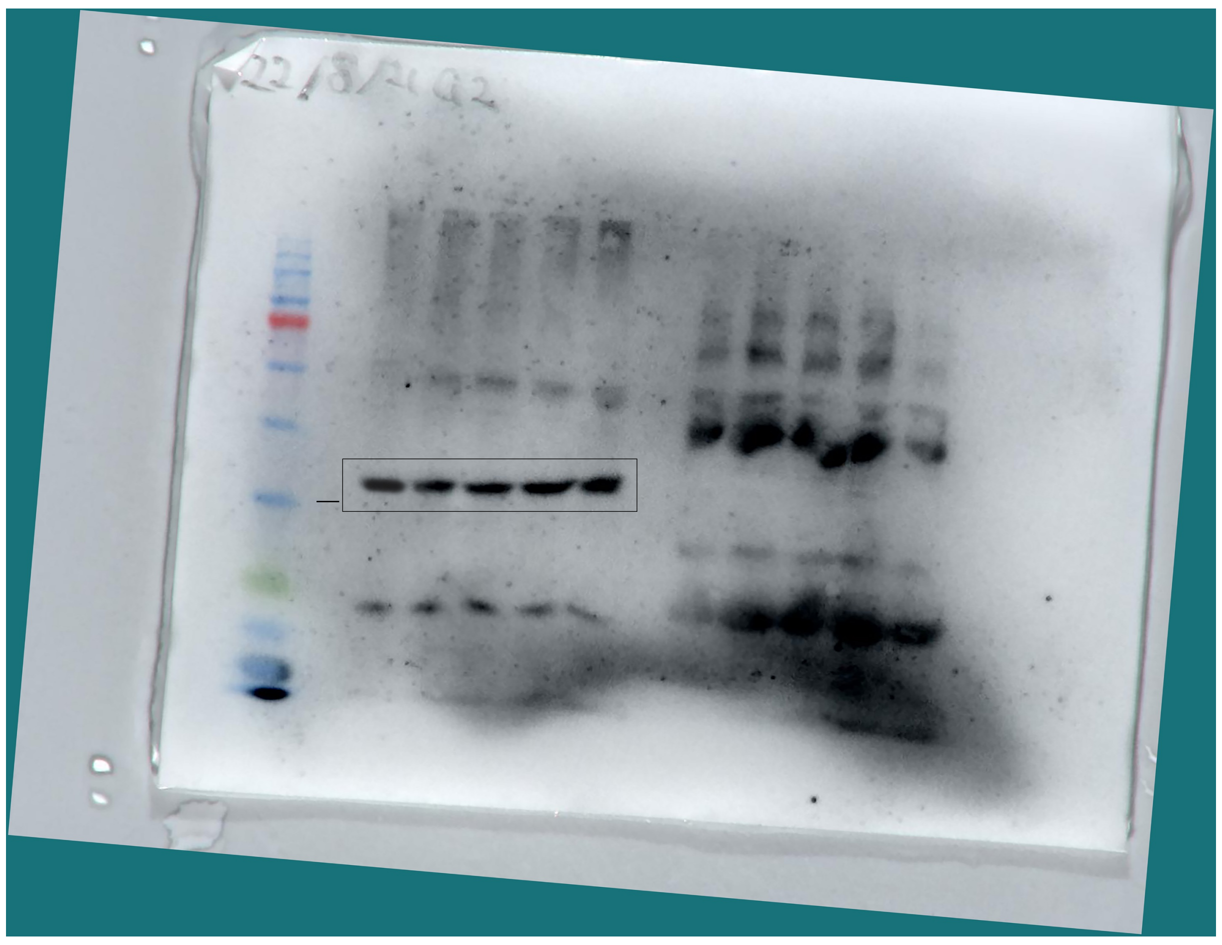

anti-GAPDH

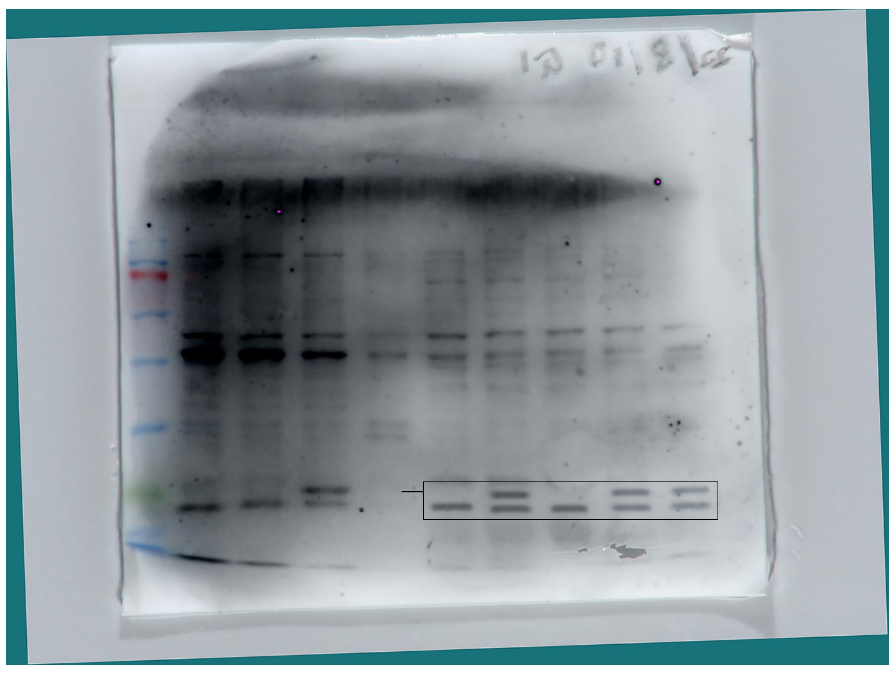

anti-MAD2

Figure S4F
IP

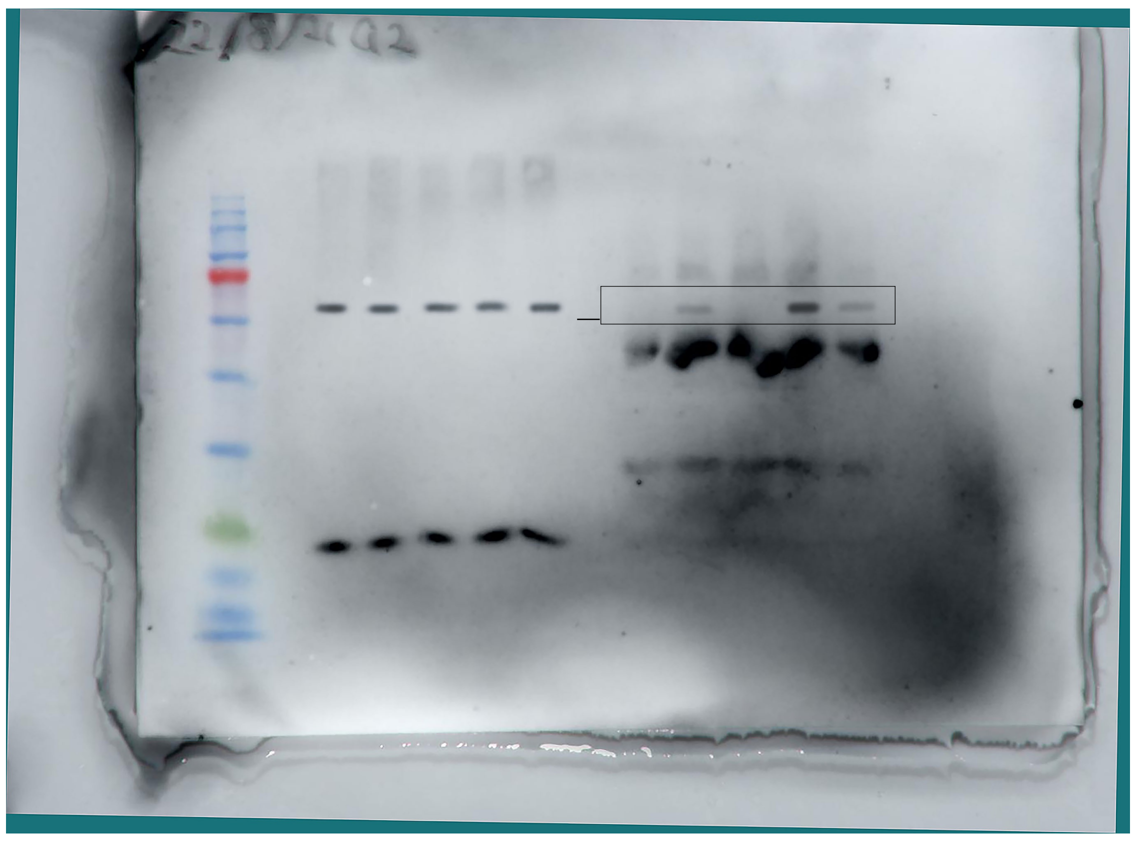

anti-AIFM1

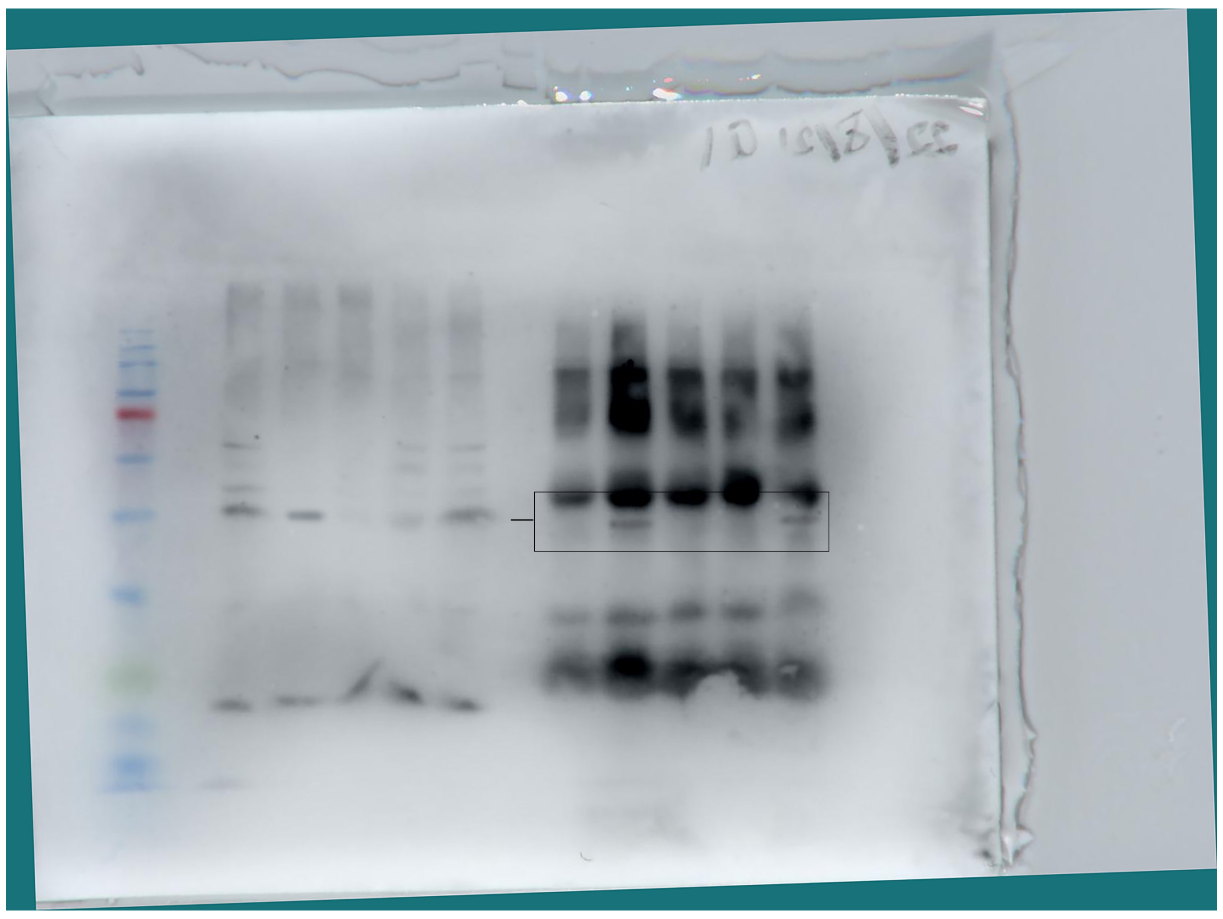

anti-TSG101

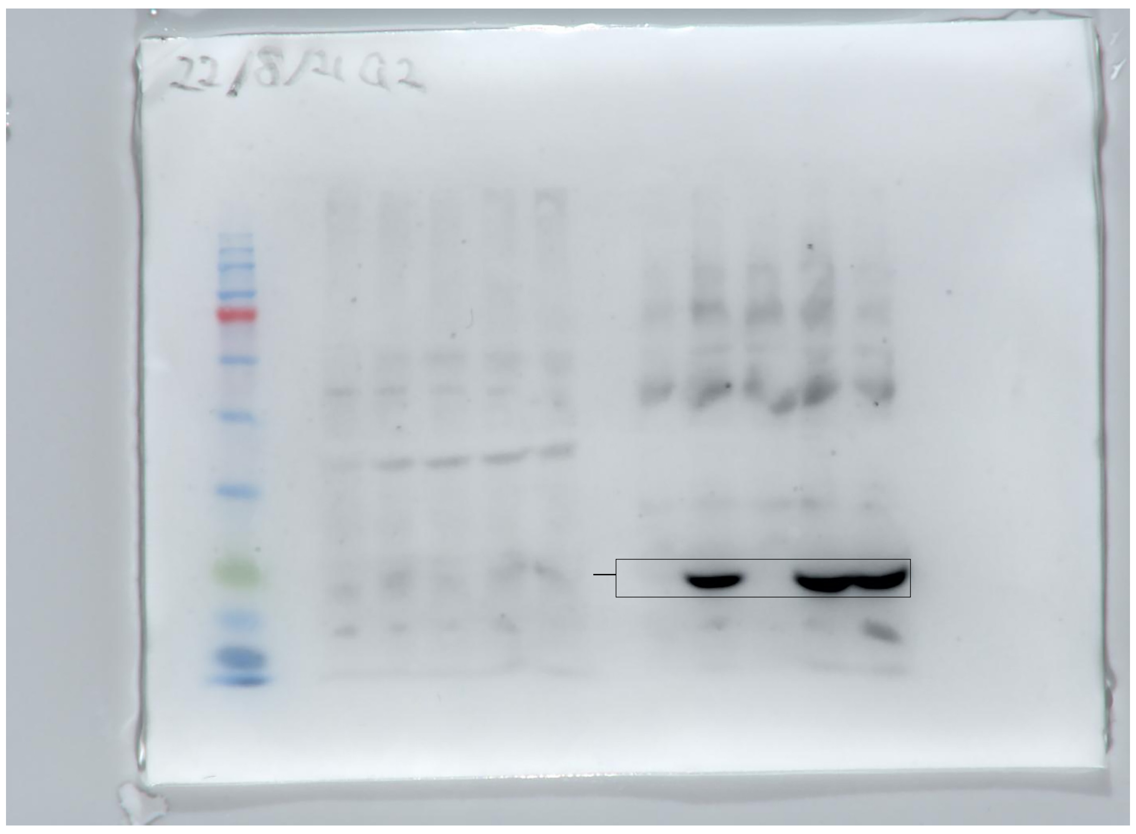

anti-MAD2

Figure S5A

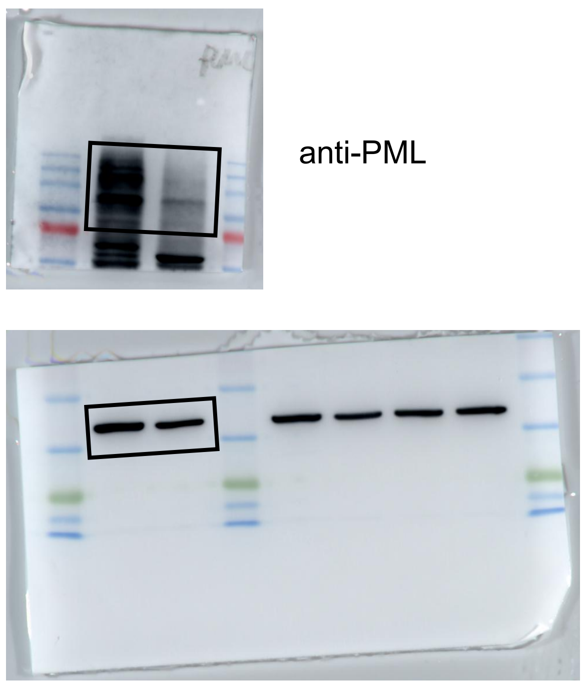

anti-PML

anti-GAPDH

Figure S5B

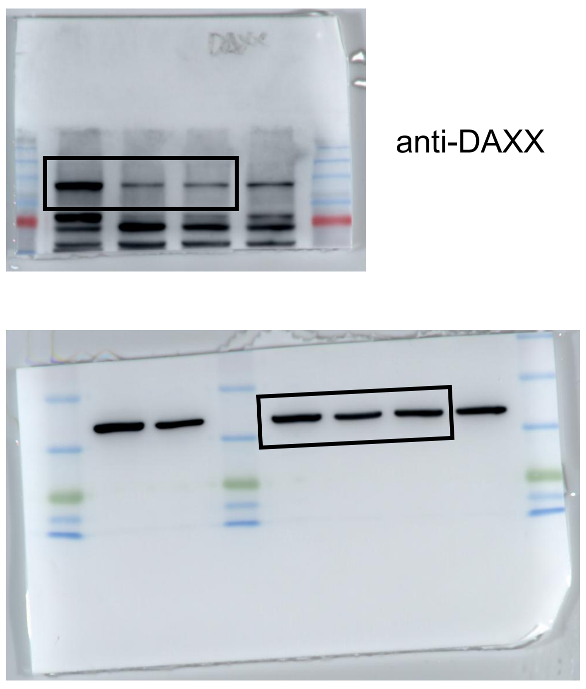

anti-DAXX

anti-GAPDH

Figure S5C

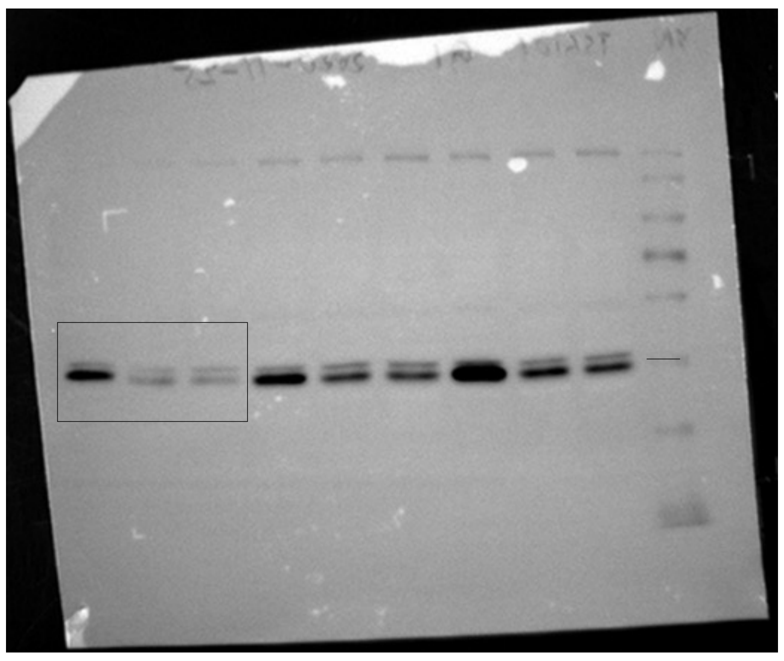

anti-TSG101

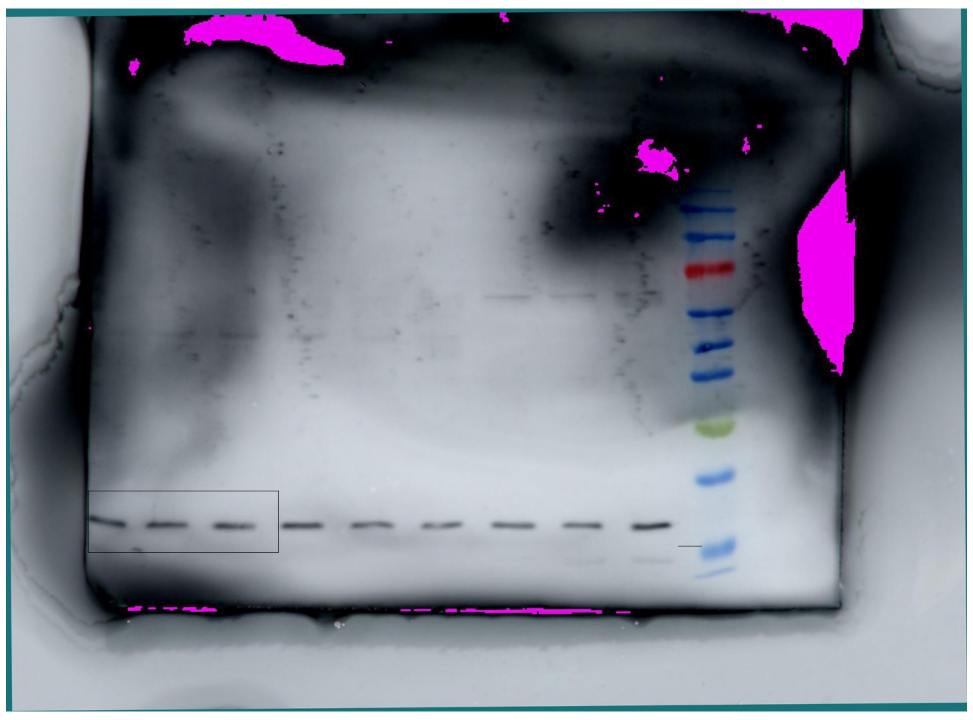

anti-CENP-A

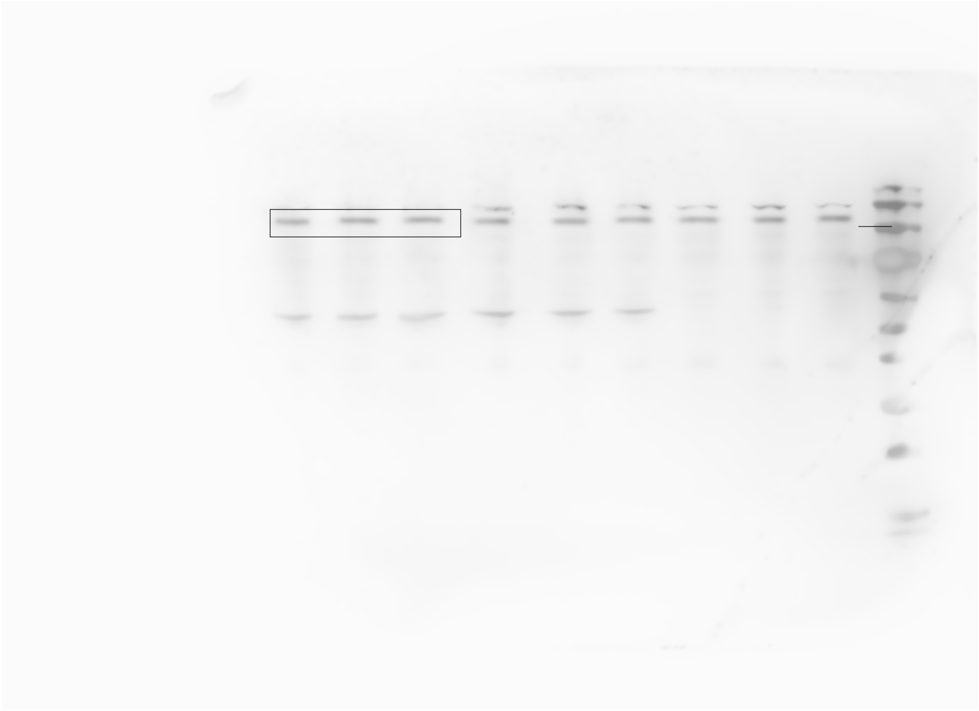

anti-PML

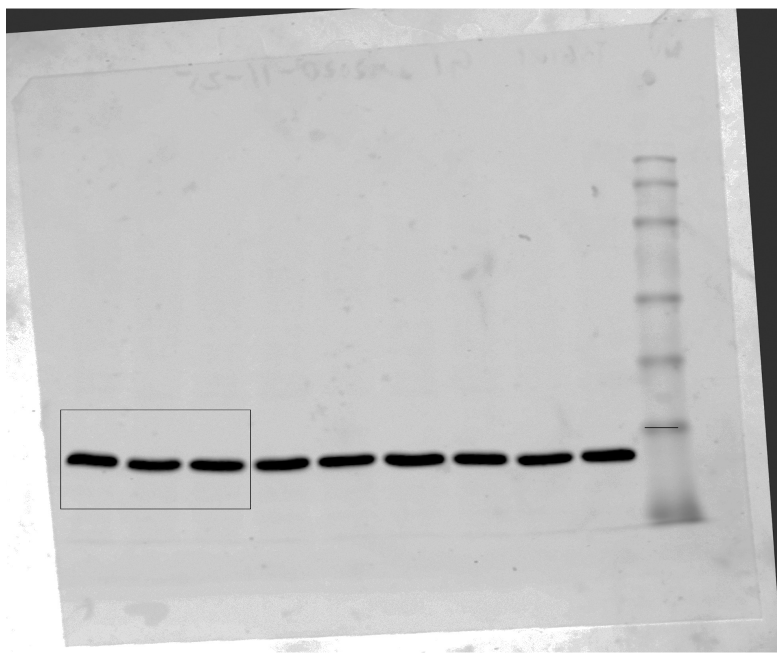

anti-GAPDH

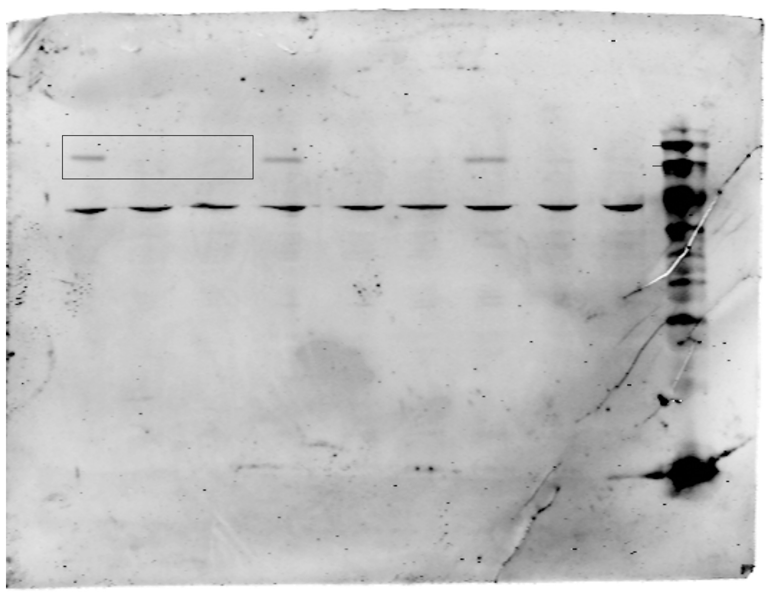

anti-DAXX

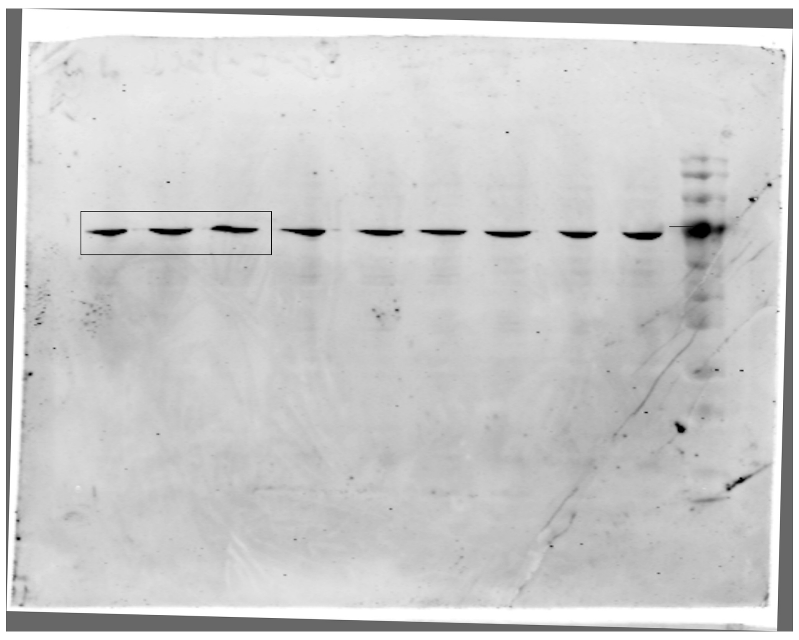

anti-AIF

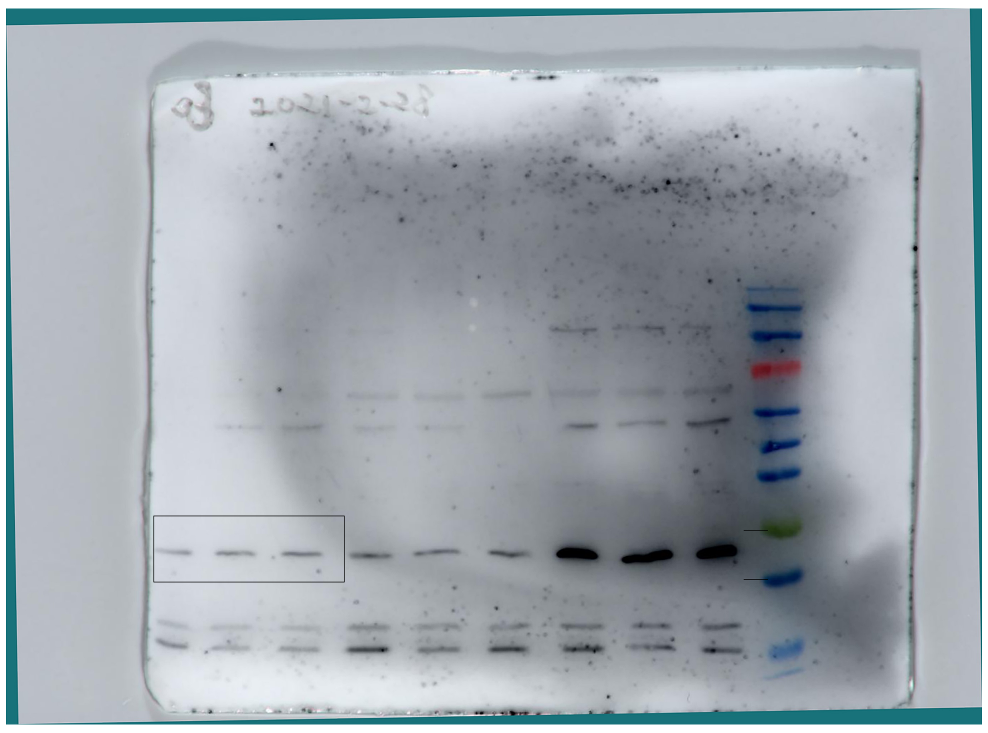

anti-MAD2

Figure S6A
Left

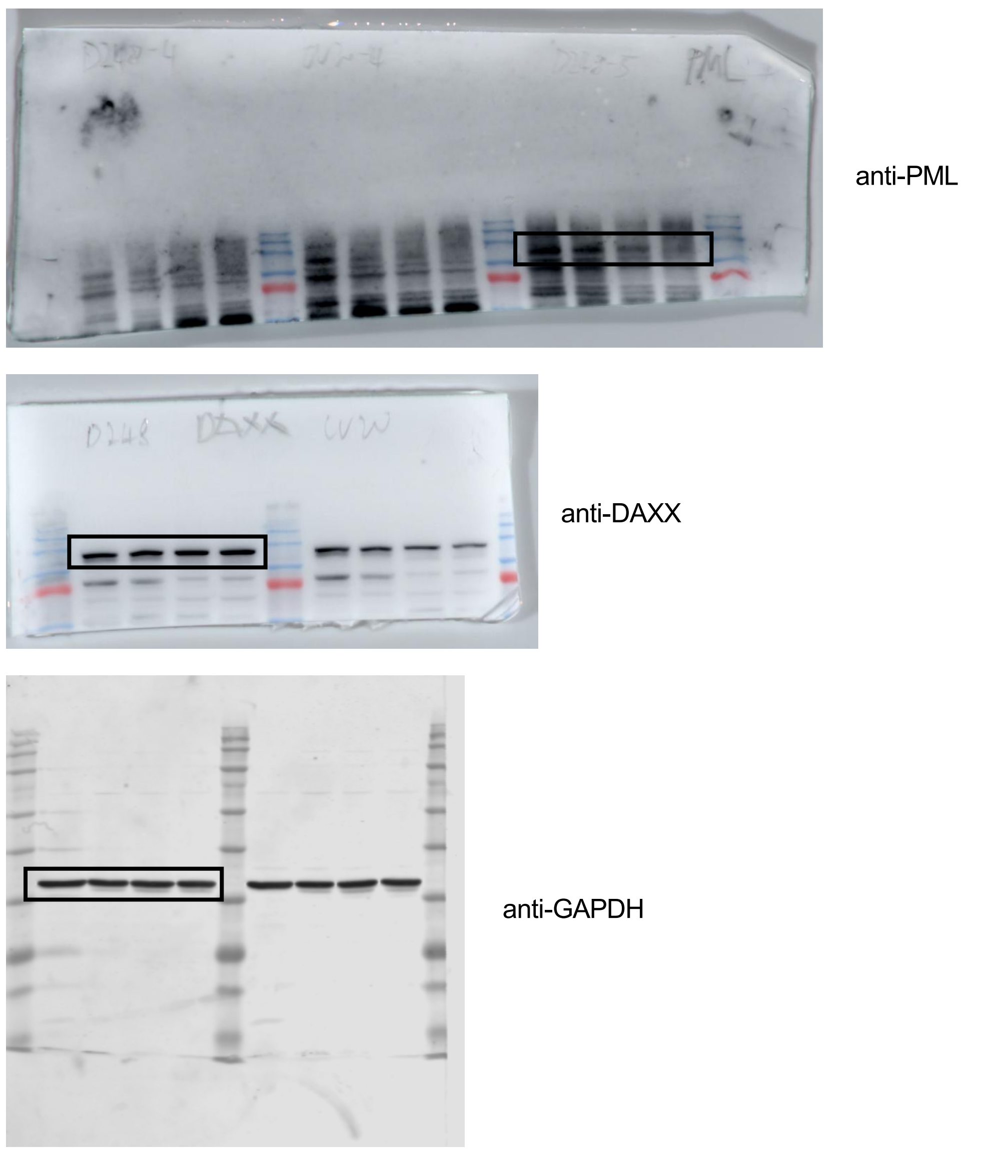

Figure S6A
Right

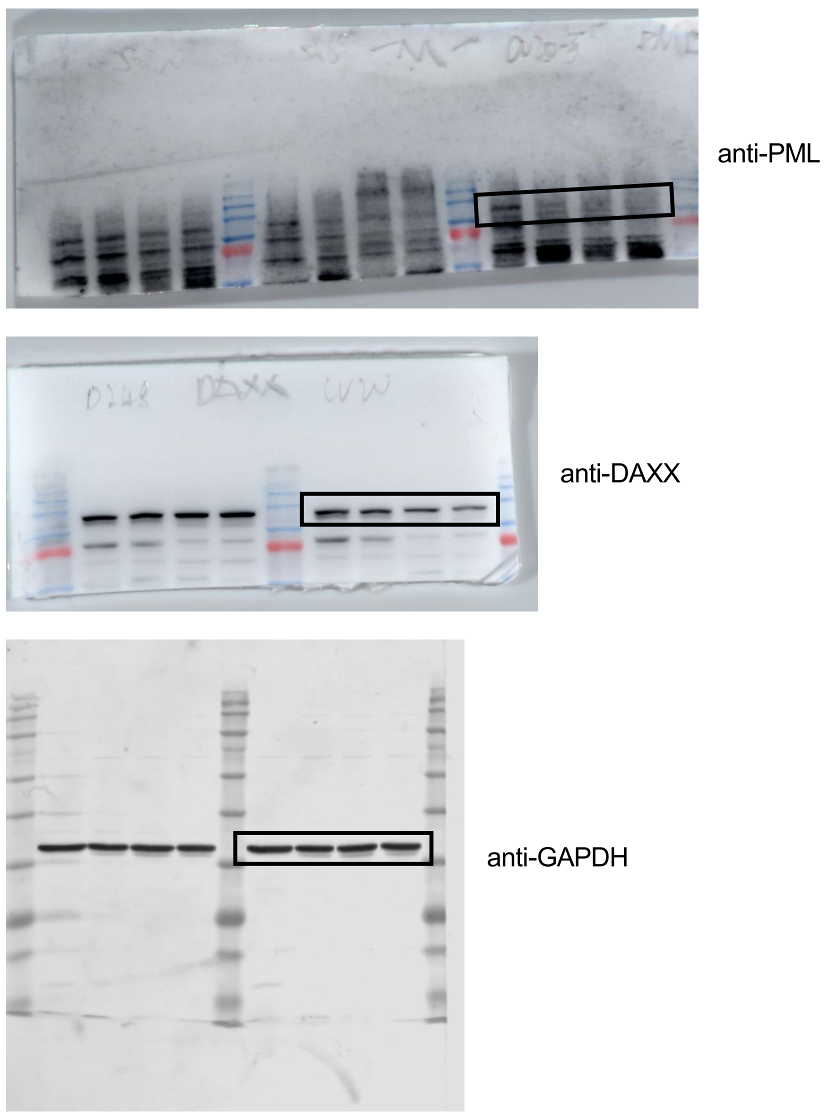

Figure S6AB
Left

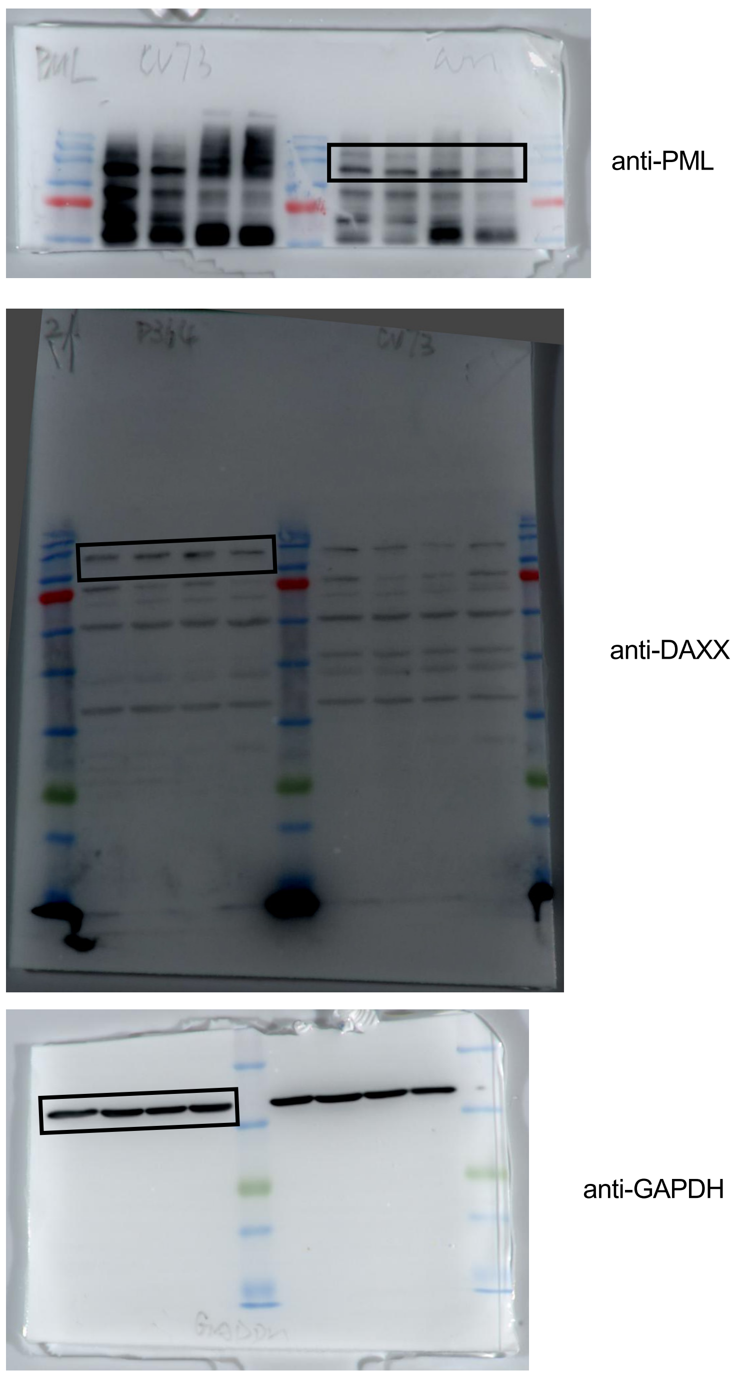

Figure S6B
Right

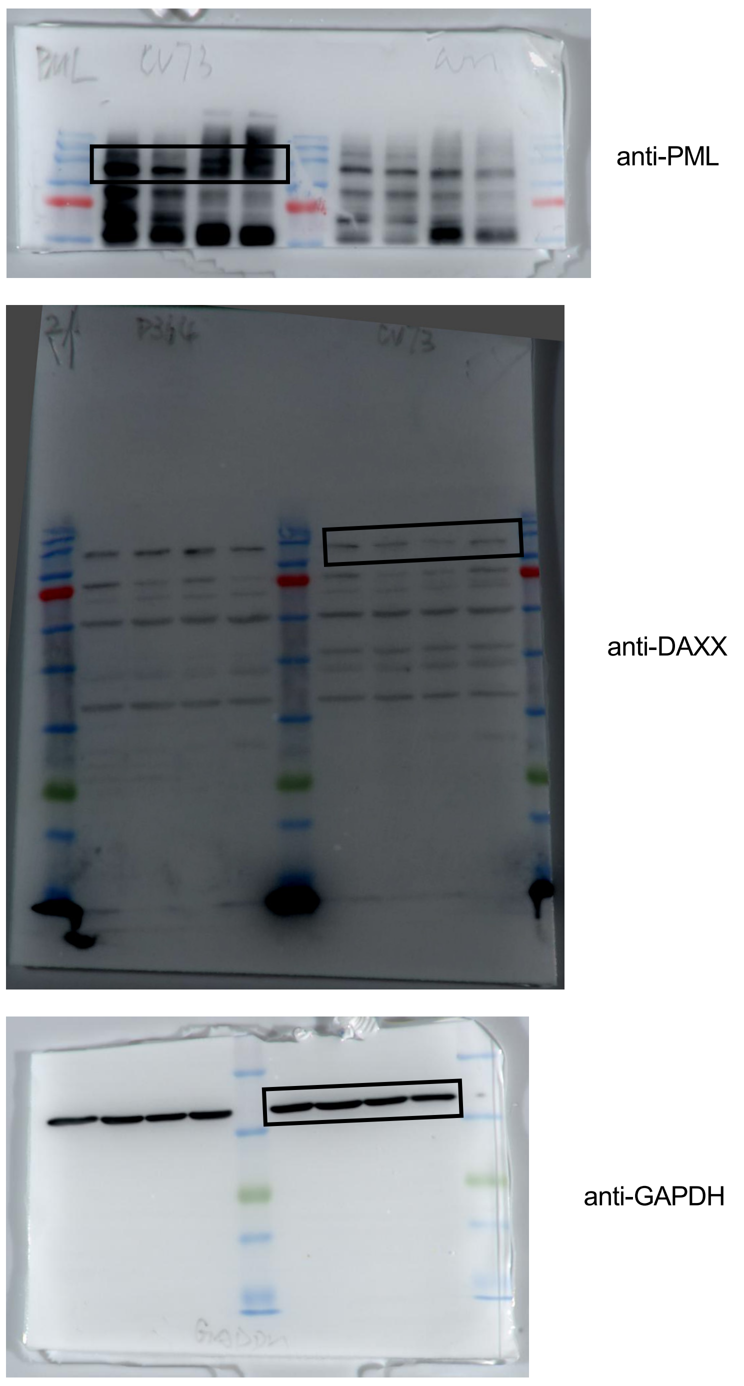

Figure S6C Left

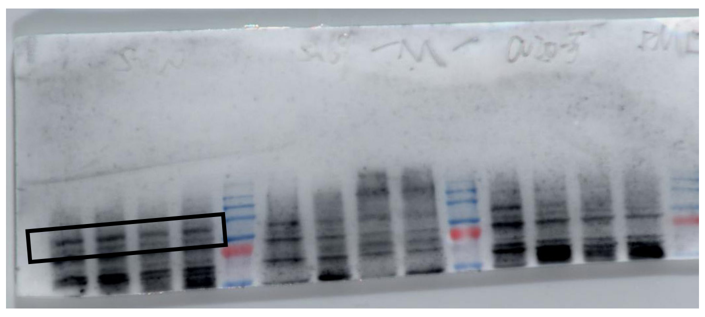

anti-PML

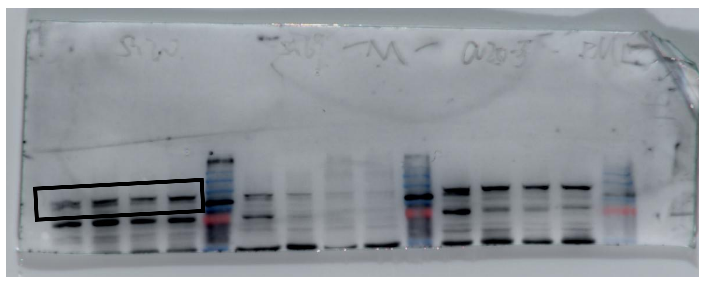

anti-DAXX

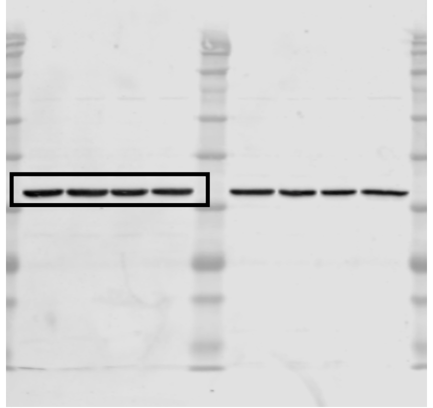

anti-GAPDH

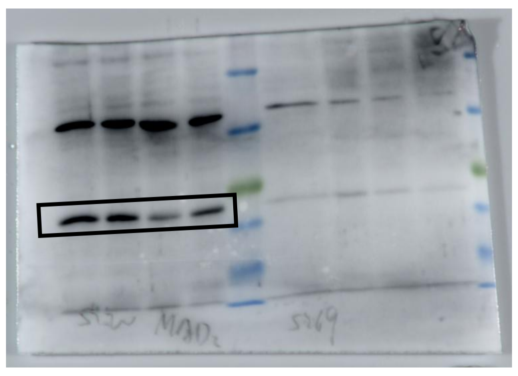

anti-MAD2

Figure S6C Right

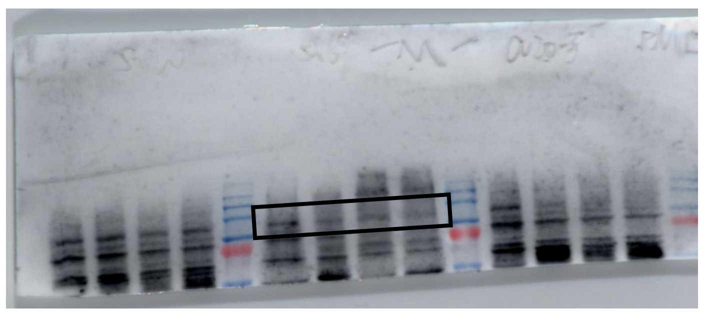

anti-PML

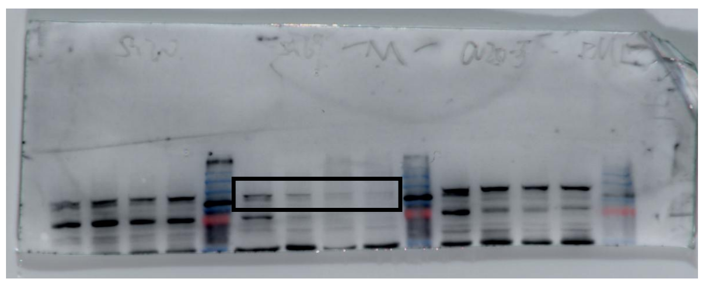

anti-DAXX

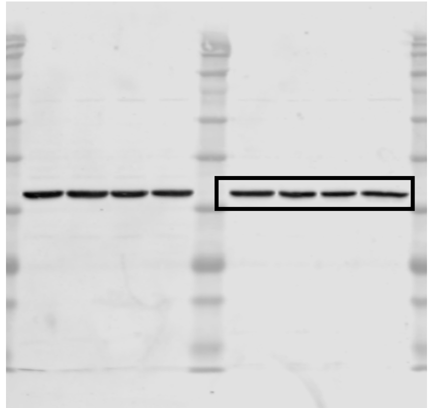

anti-GAPDH

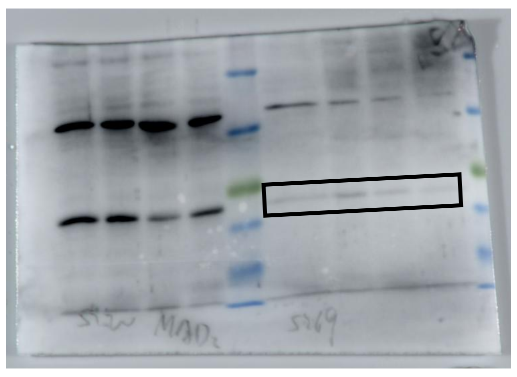

anti-MAD2

Figure S7A

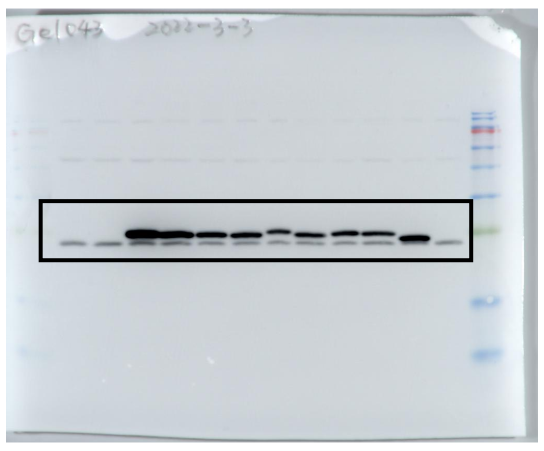

anti-MAD2

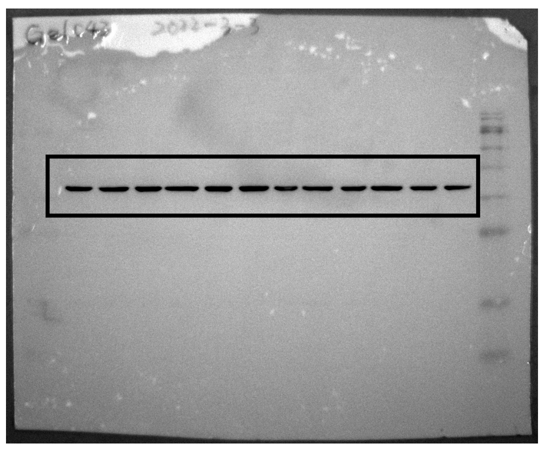

anti-GAPDH

Figure S7G
IP

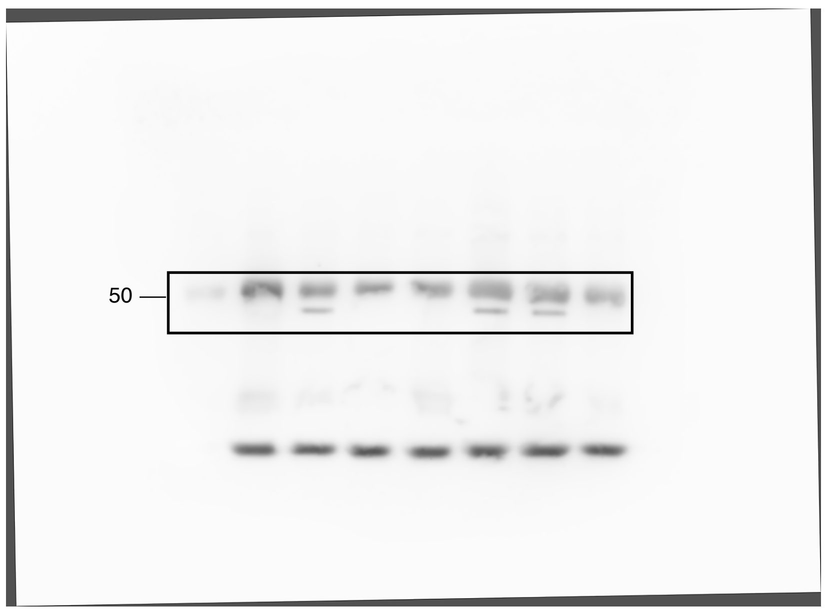

anti-HA(TSG101)

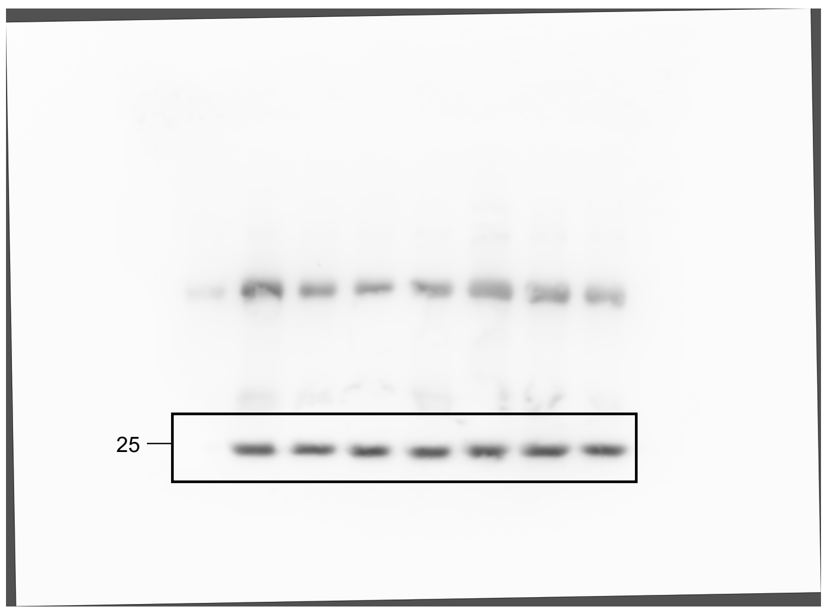

anti-Flag(MAD2)

Figure S7G
Input

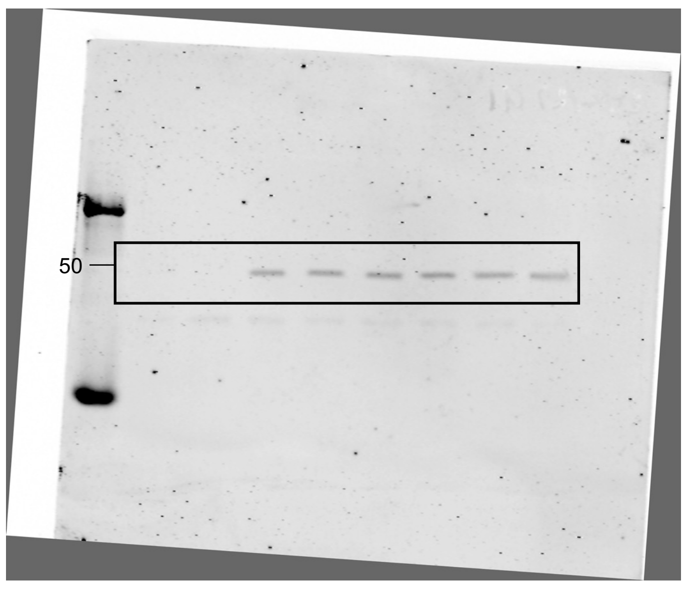

anti-HA(TSG101)

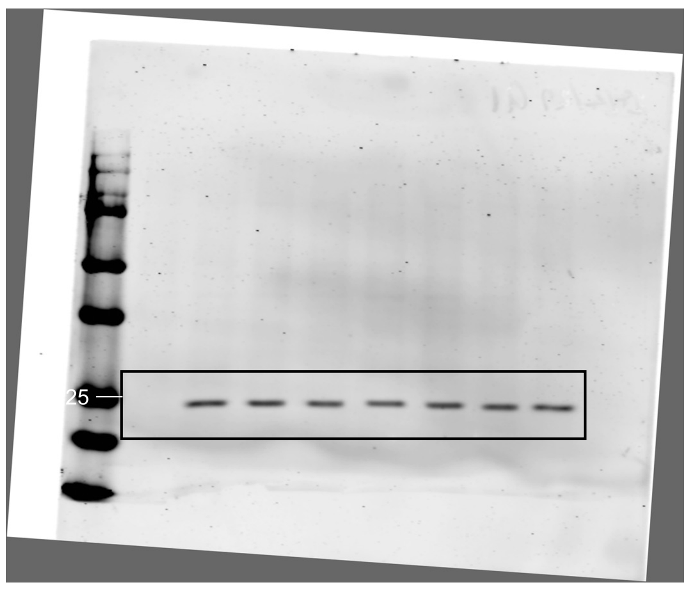

anti-Flag(MAD2)

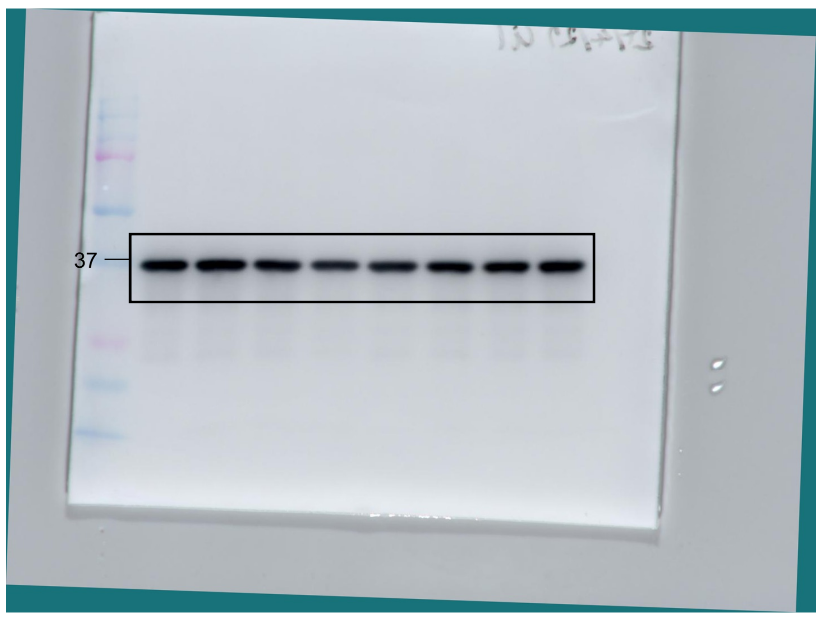

anti-GAPDH

Figure S7J

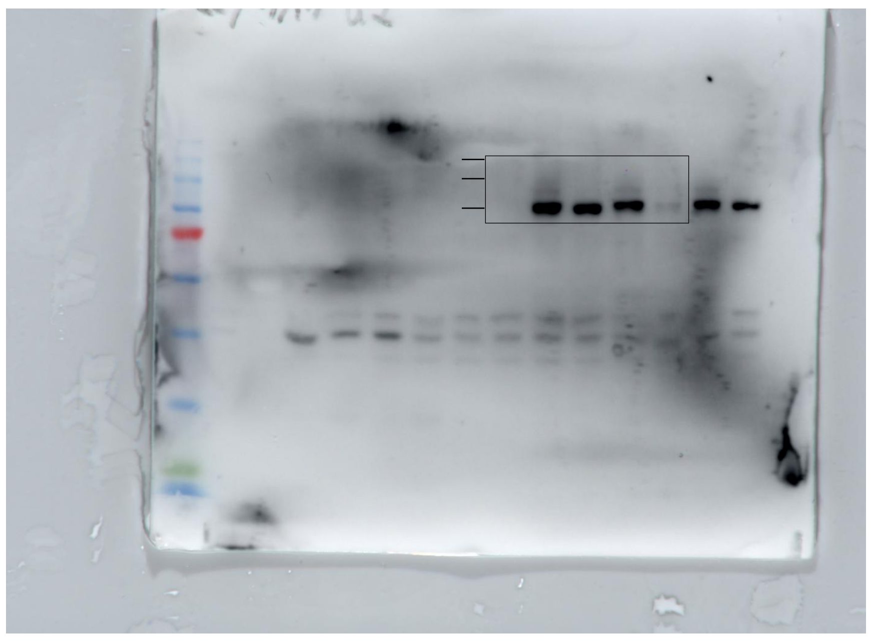

anti-HA(PML)

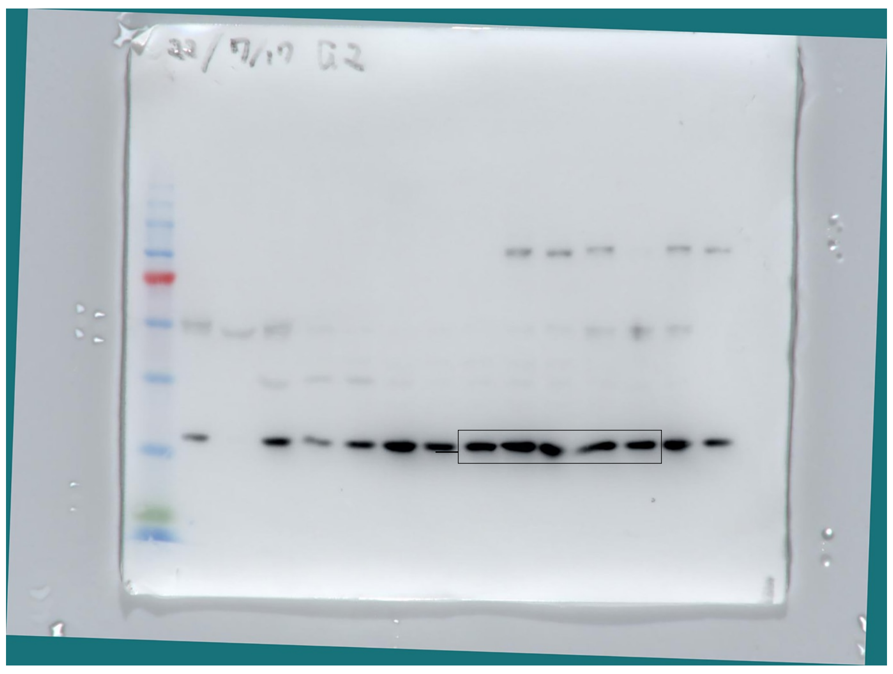

Anti-GAPDH

Figure S7K
IP

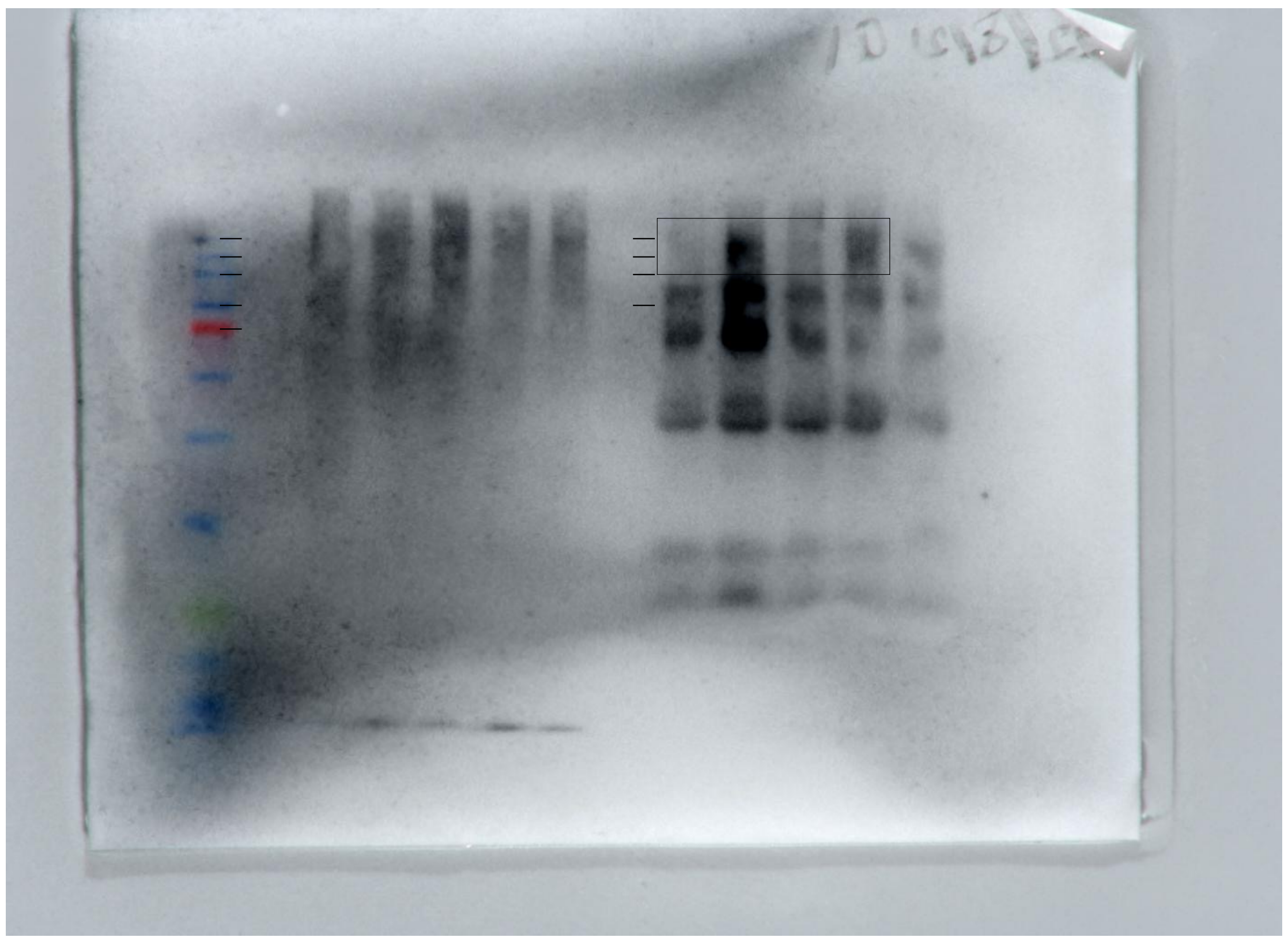

anti-SUMO2

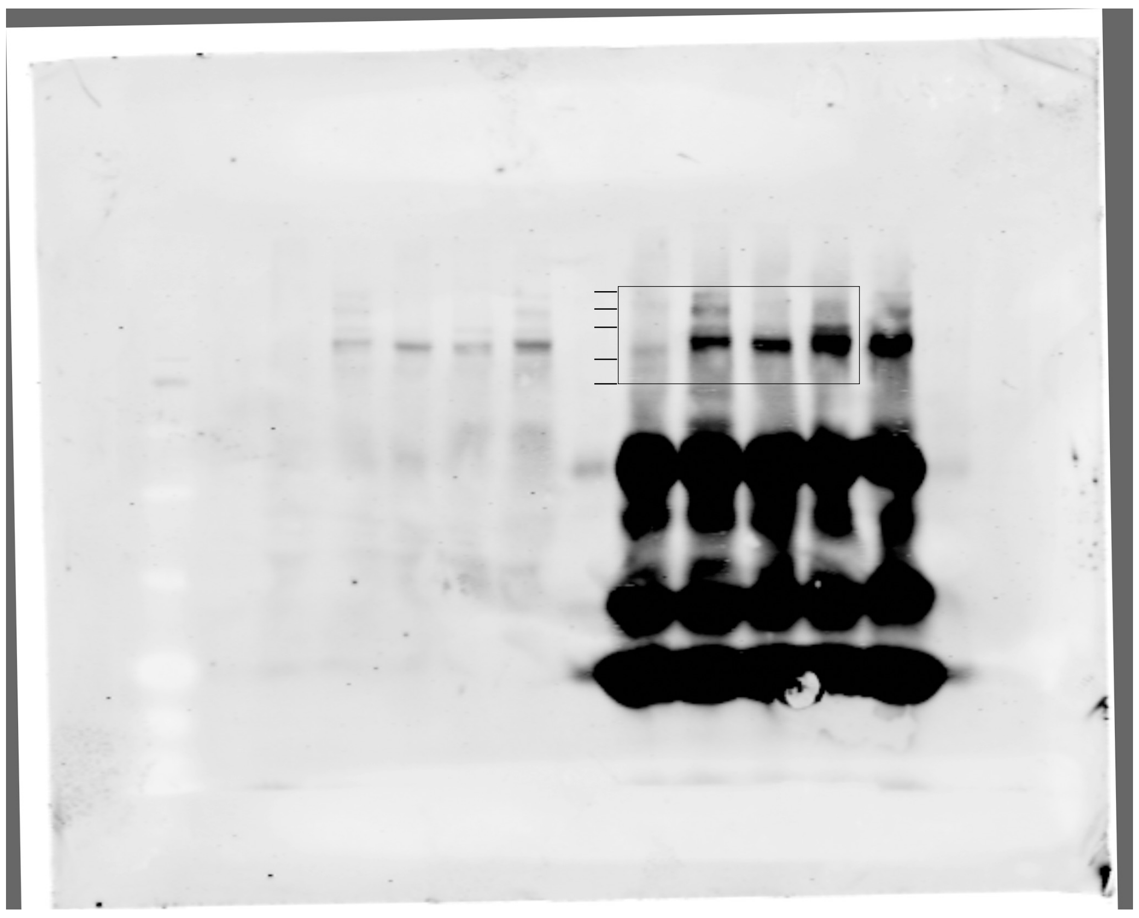

anti-Flag(PML)

Figure S7K
Input

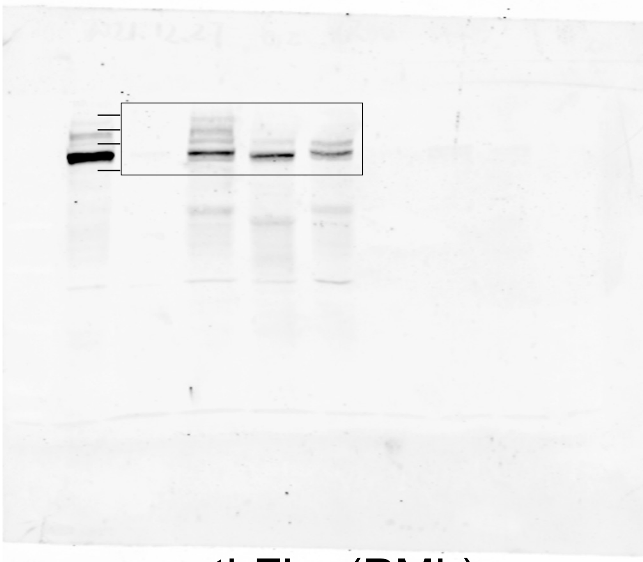

anti-Flag(PML)

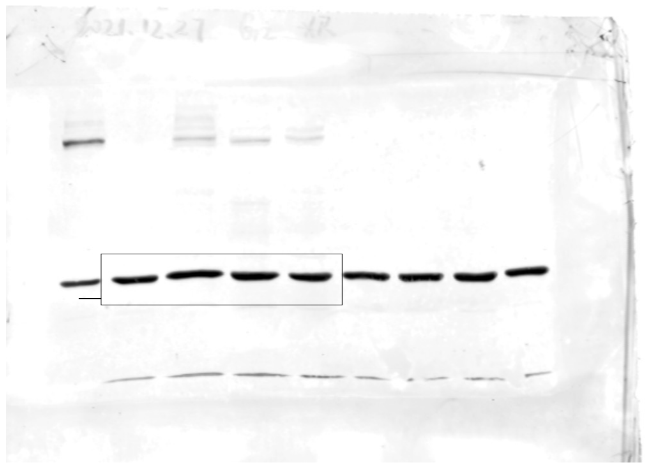

anti-GAPDH

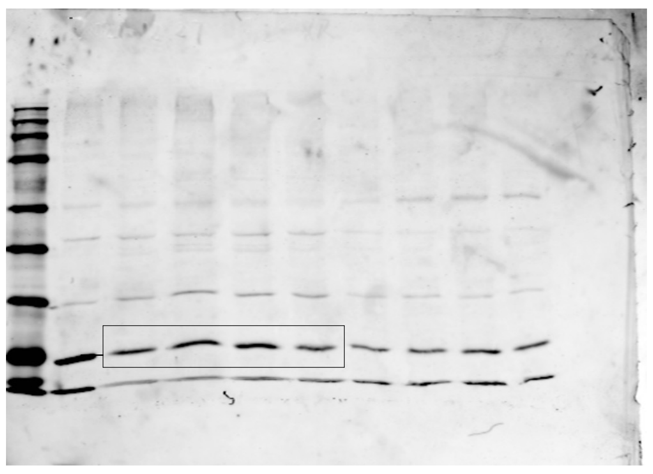

anti-MAD2

Figure S8A
IP

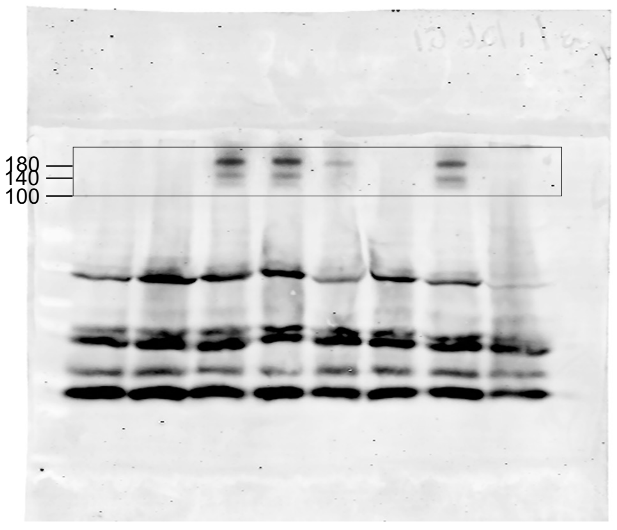

anti-SUMO2

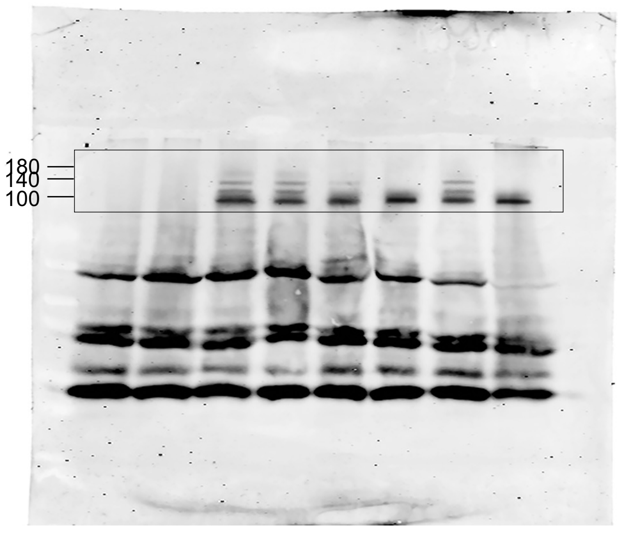

anti-HA(PML)

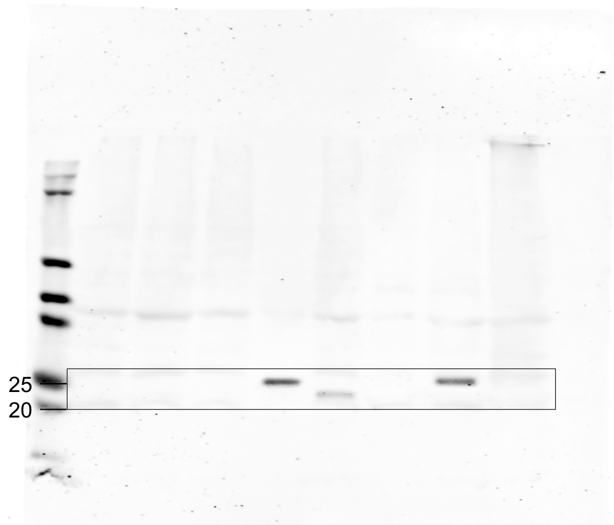

anti-MAD2

Figure S8A
Input

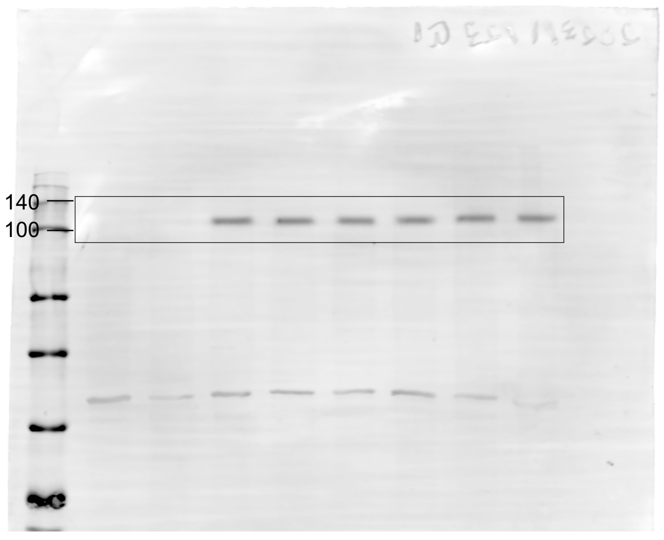

anti-HA(PML)

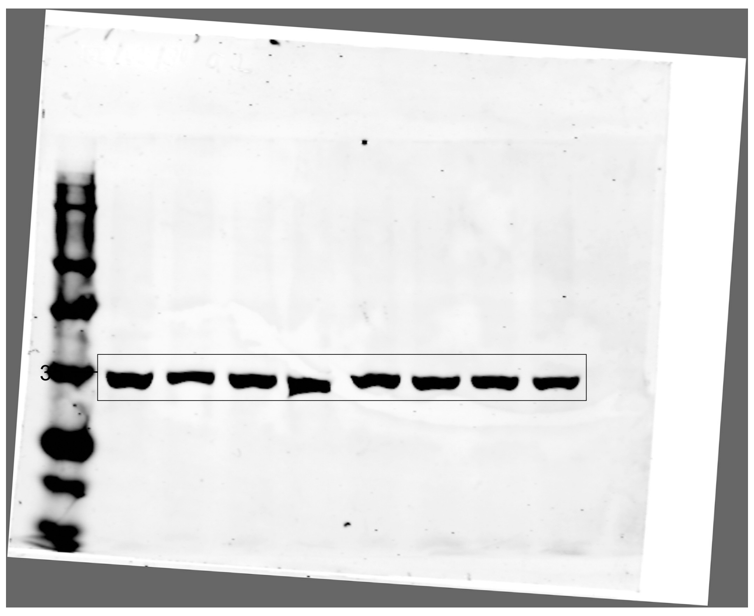

anti-GAPDH

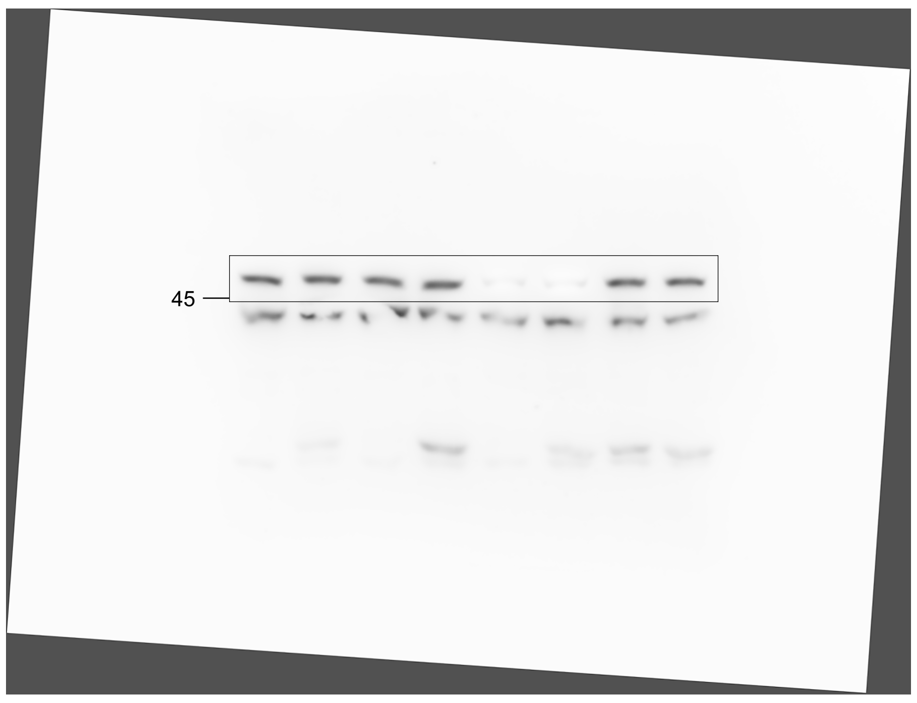

anti-TSG101

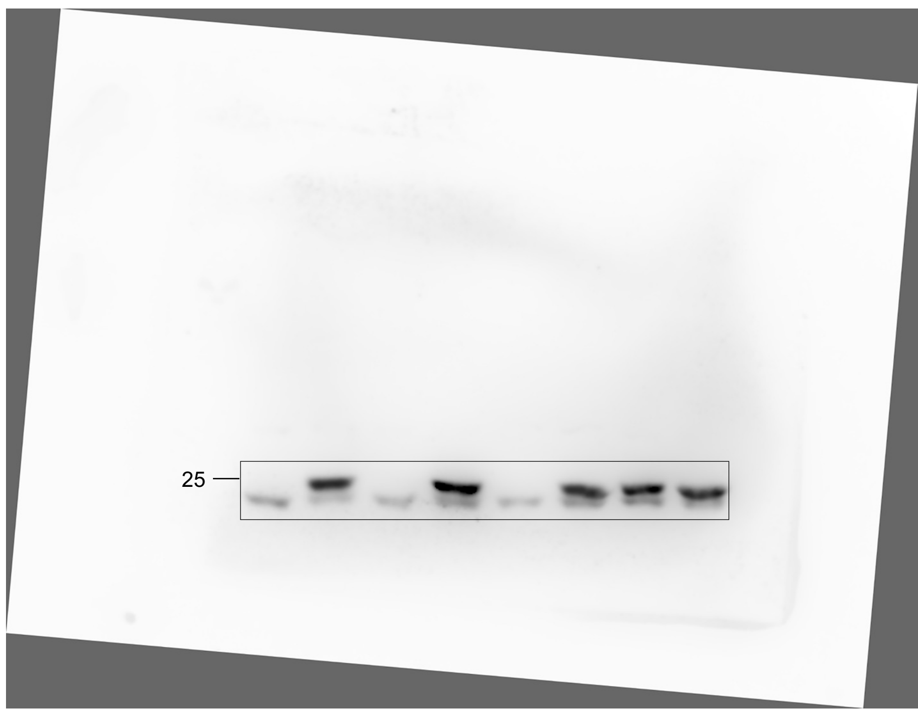

anti-MAD2

Figure S8B
IP

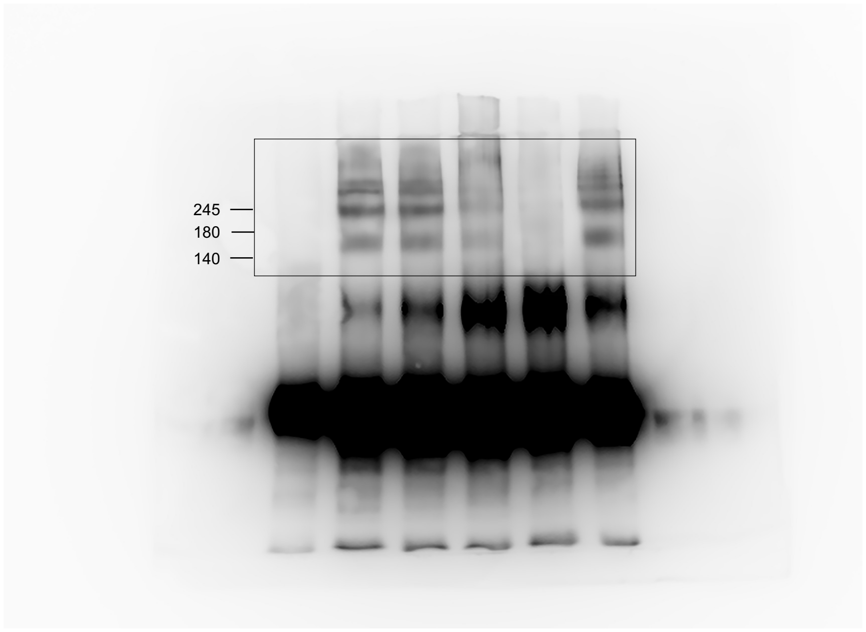

anti-SUMO2

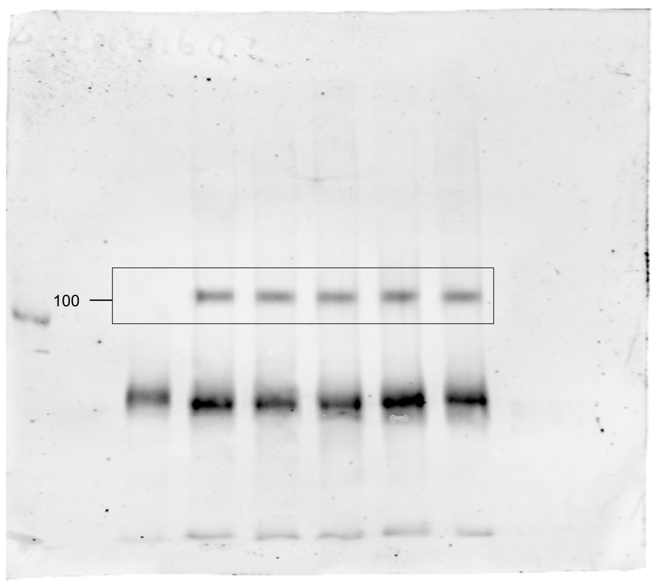

anti-PML

Figure S8B
Input

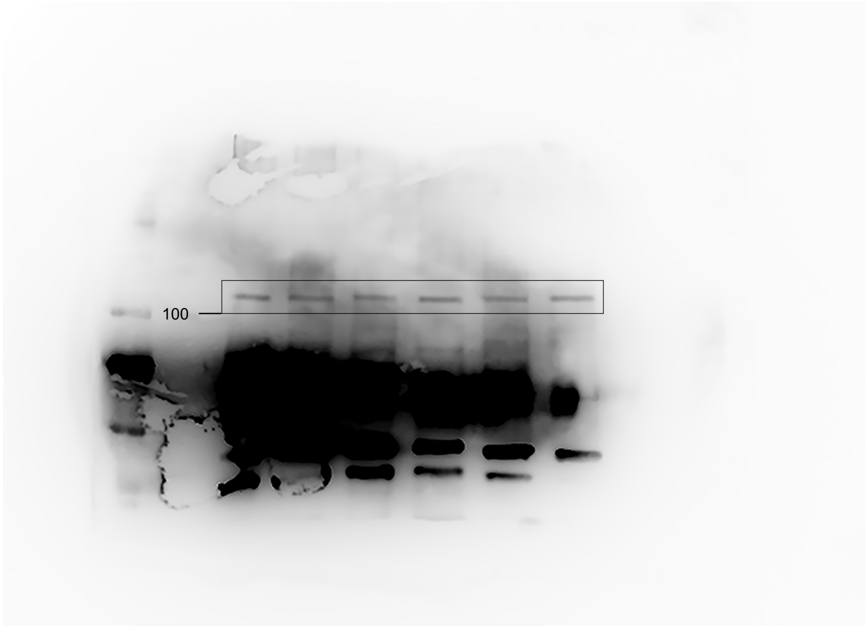

anti-PML

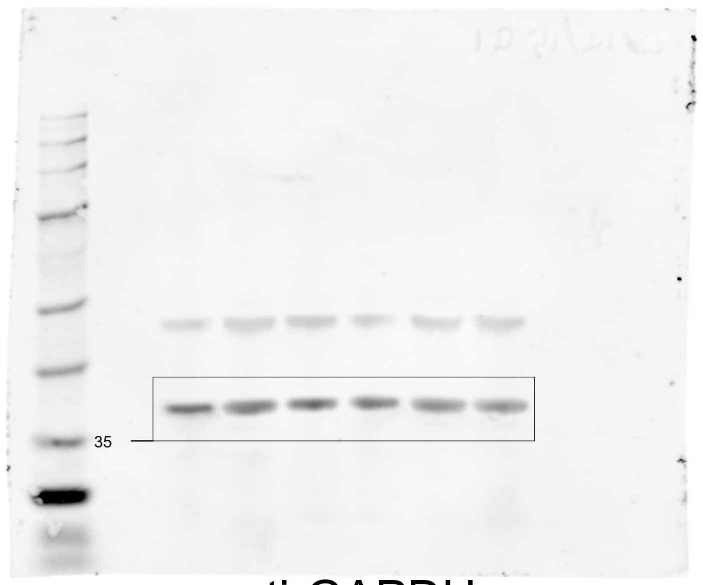

anti-GAPDH

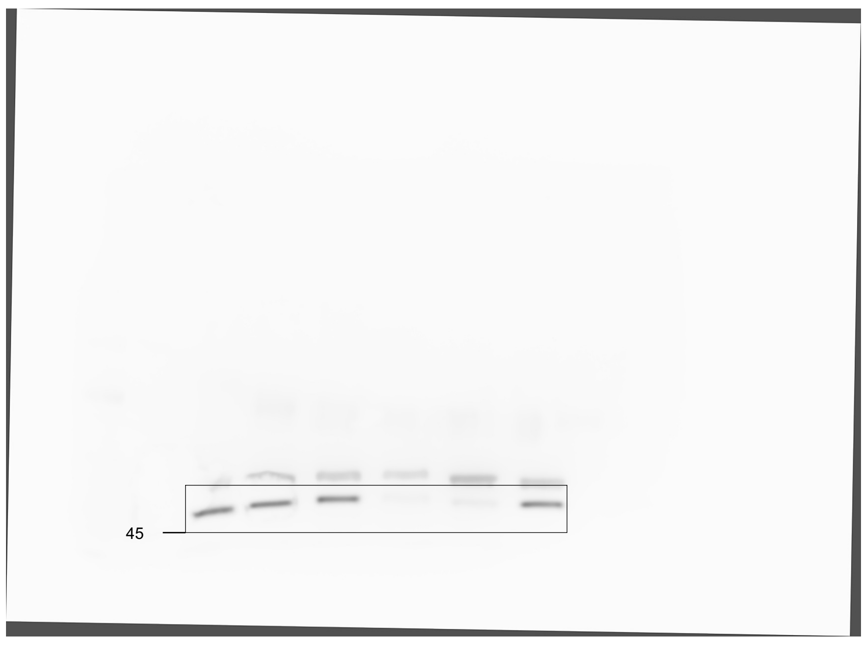

anti-TSG101

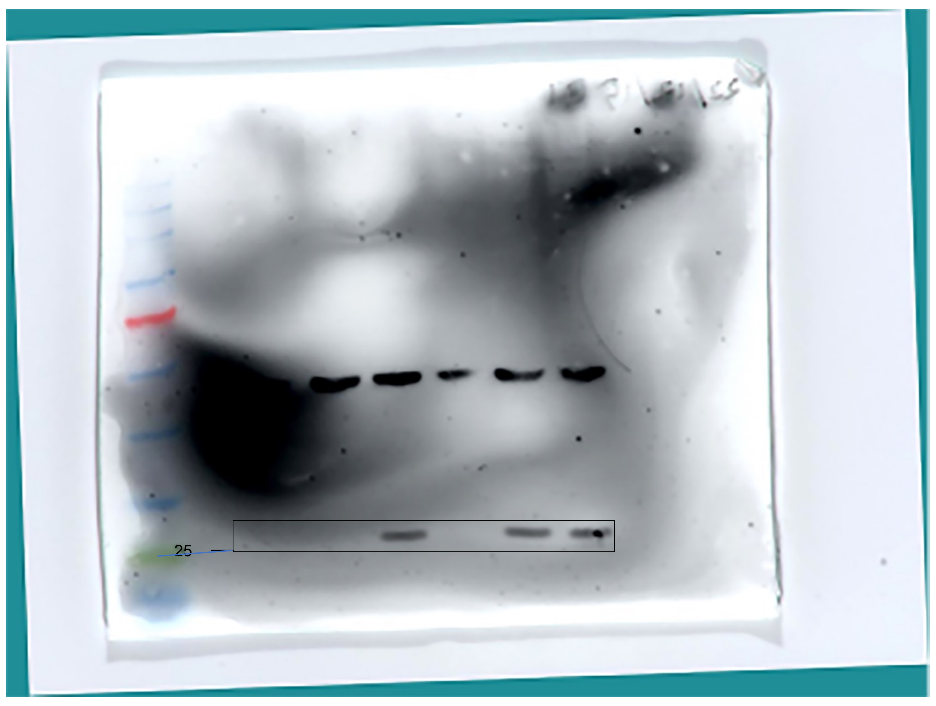

anti-MAD2

Figure S8C
IP

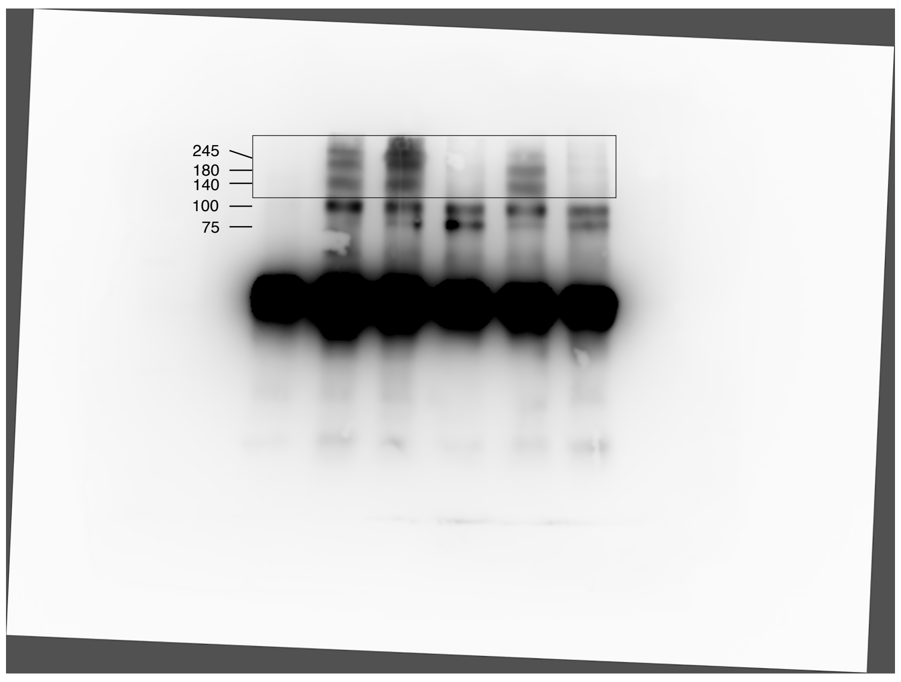

anti-SUMO2

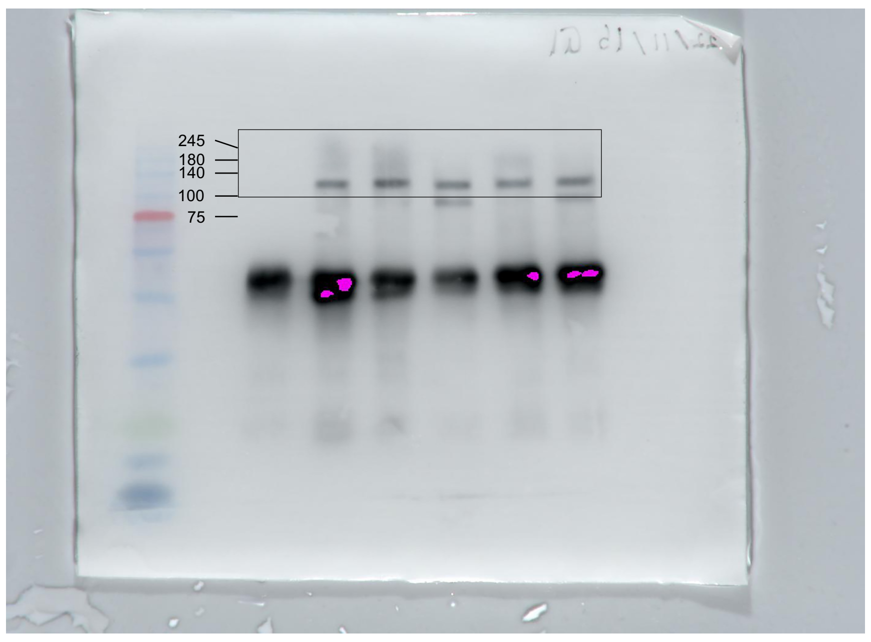

anti-PML

Figure S8C
Input

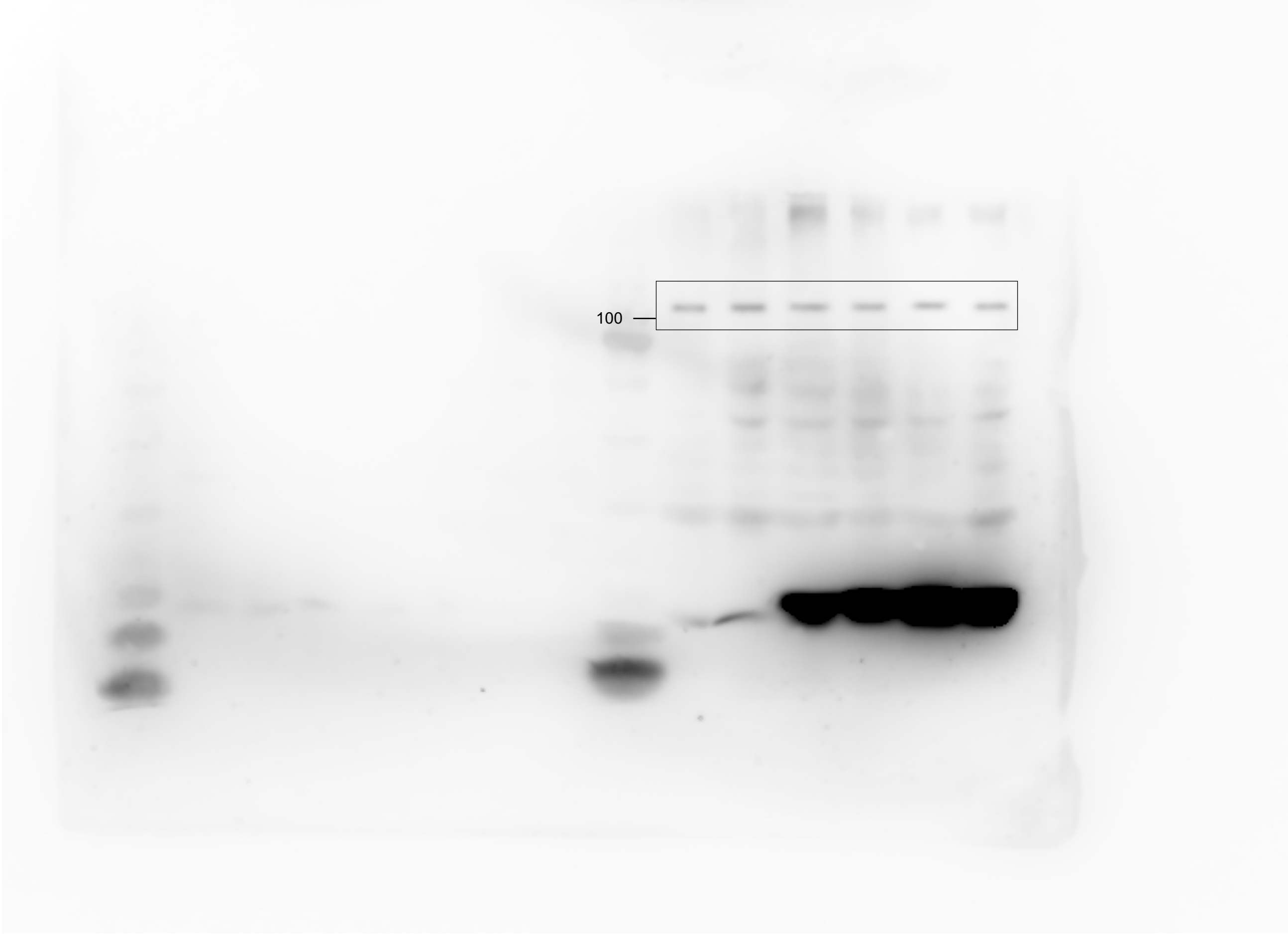

anti-PML

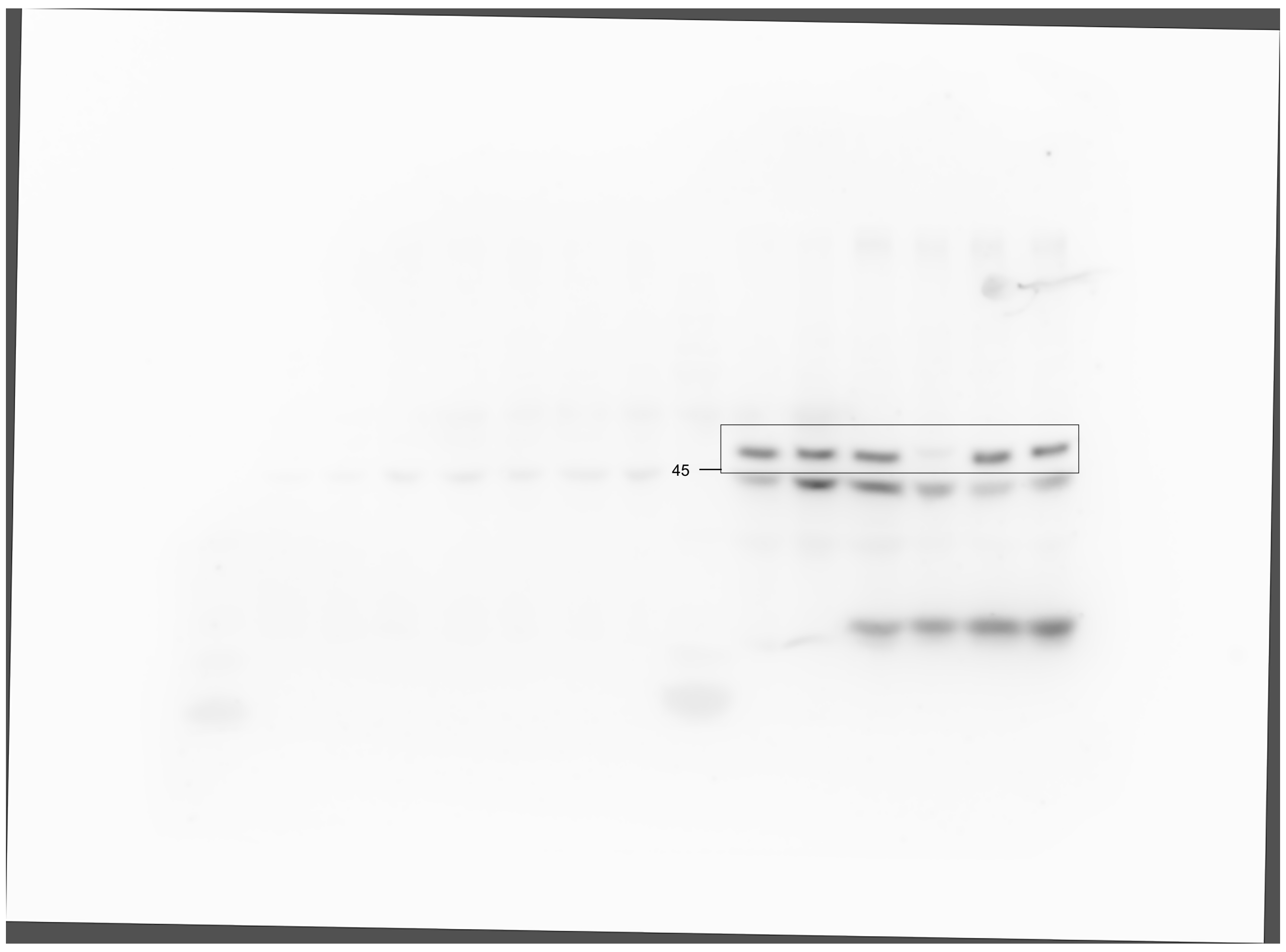

anti-TSG101

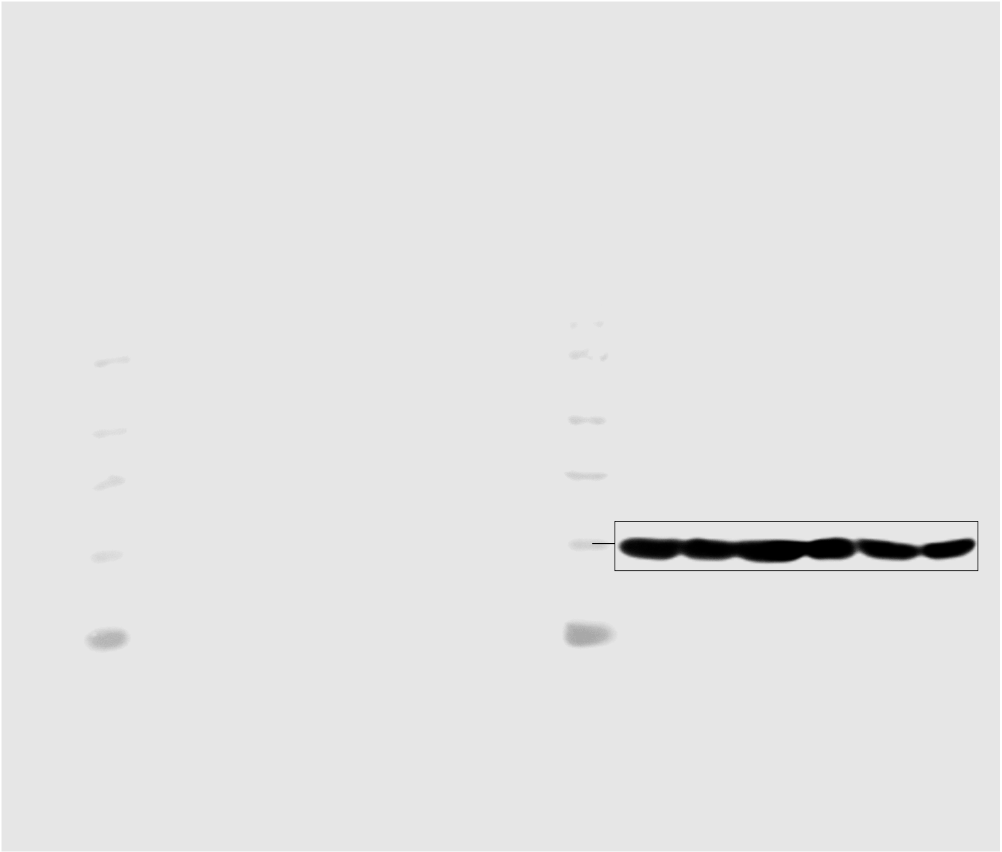

anti-GAPDH

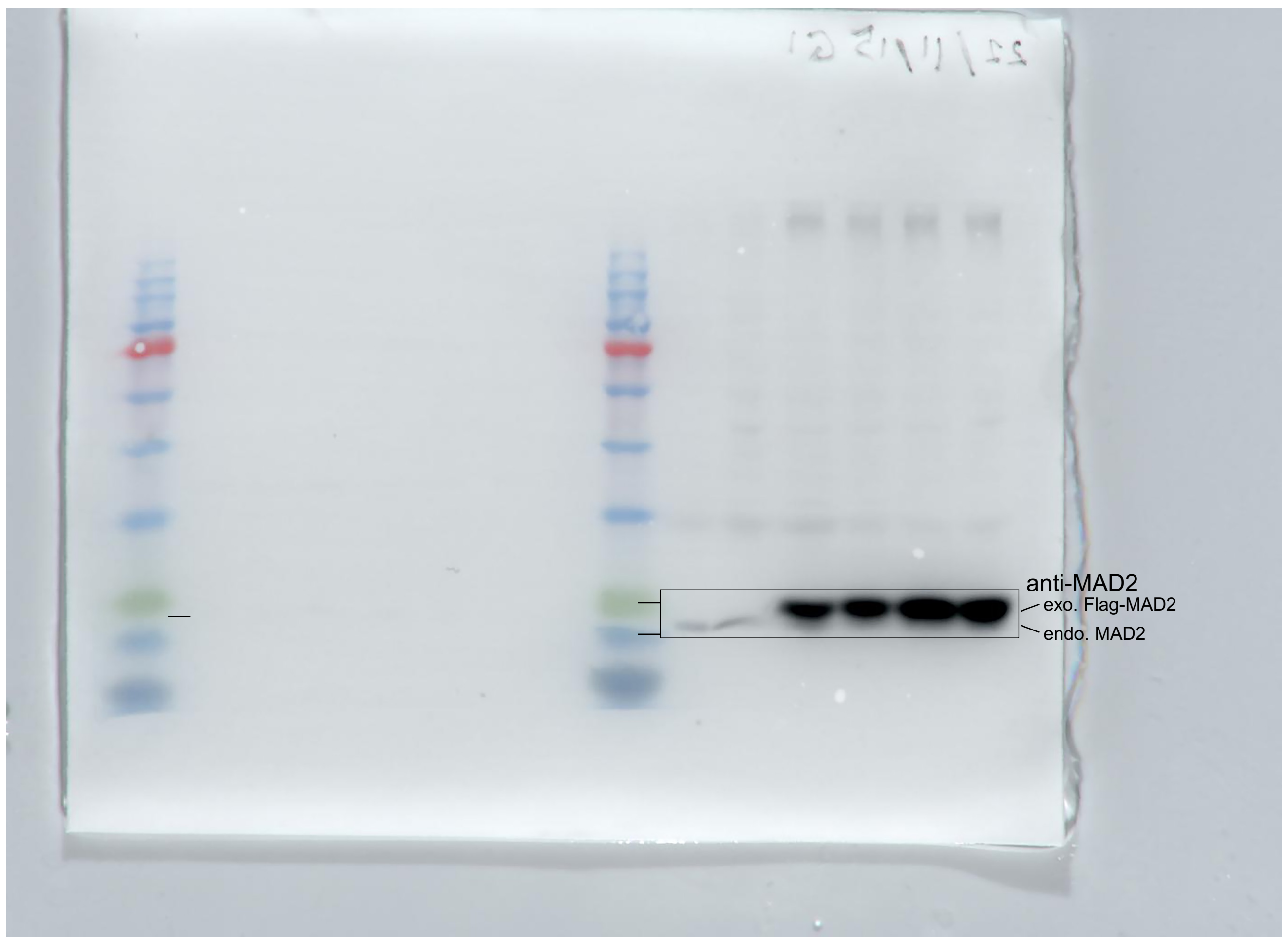

anti-MAD2
